# Phosphirenium Ions as Masked Phosphenium Catalysts: Mechanistic Evaluation and Application in Synthesis

**DOI:** 10.1021/acscatal.1c01133

**Published:** 2021-04-20

**Authors:** Danila Gasperini, Samuel E. Neale, Mary F. Mahon, Stuart A. Macgregor, Ruth L. Webster

**Affiliations:** † Department of Chemistry, University of Bath, Bath BA2 7AY, U.K.; ‡ Institute of Chemical Sciences, Heriot-Watt University, Edinburgh EH14 4AS, U.K.

**Keywords:** phosphenium ions, phosphirenium ions, organocatalysis, reaction mechanism studies, density functional theory, hydrosilylation

## Abstract

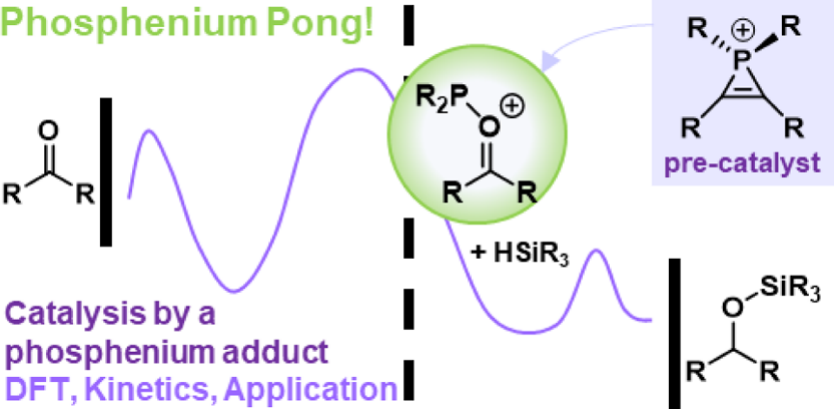

The utilization of phosphirenium ions is presented; optimized and broadened three-membered ring construction is described together with the use of these ions as efficient pre-catalysts for metal-free carbonyl reduction with silanes. Full characterization of the phosphirenium ions is presented, and initial experimental and computational mechanistic studies indicate that these act as a “masked phosphenium” source that is accessed via ring opening. Catalysis proceeds via associative transfer of {Ph_2_P^+^} to a carbonyl nucleophile, H–SiR_3_ bond addition over the C=O group, and associative displacement of the product by a further equivalent of the carbonyl substrate, which completes the catalytic cycle. A competing off-cycle process leading to vinyl phosphine formation is detailed for the hydrosilylation of benzophenone for which an inverse order in [silane] is observed. Experimentally, the formation of side products, including off-cycle vinyl phosphine, is favored by electron-donating substituents on the phosphirenium cation, while catalytic hydrosilylation is promoted by electron-withdrawing substituents. These observations are rationalized in parallel computational studies.

## 1 Introduction

**Scheme 1:**
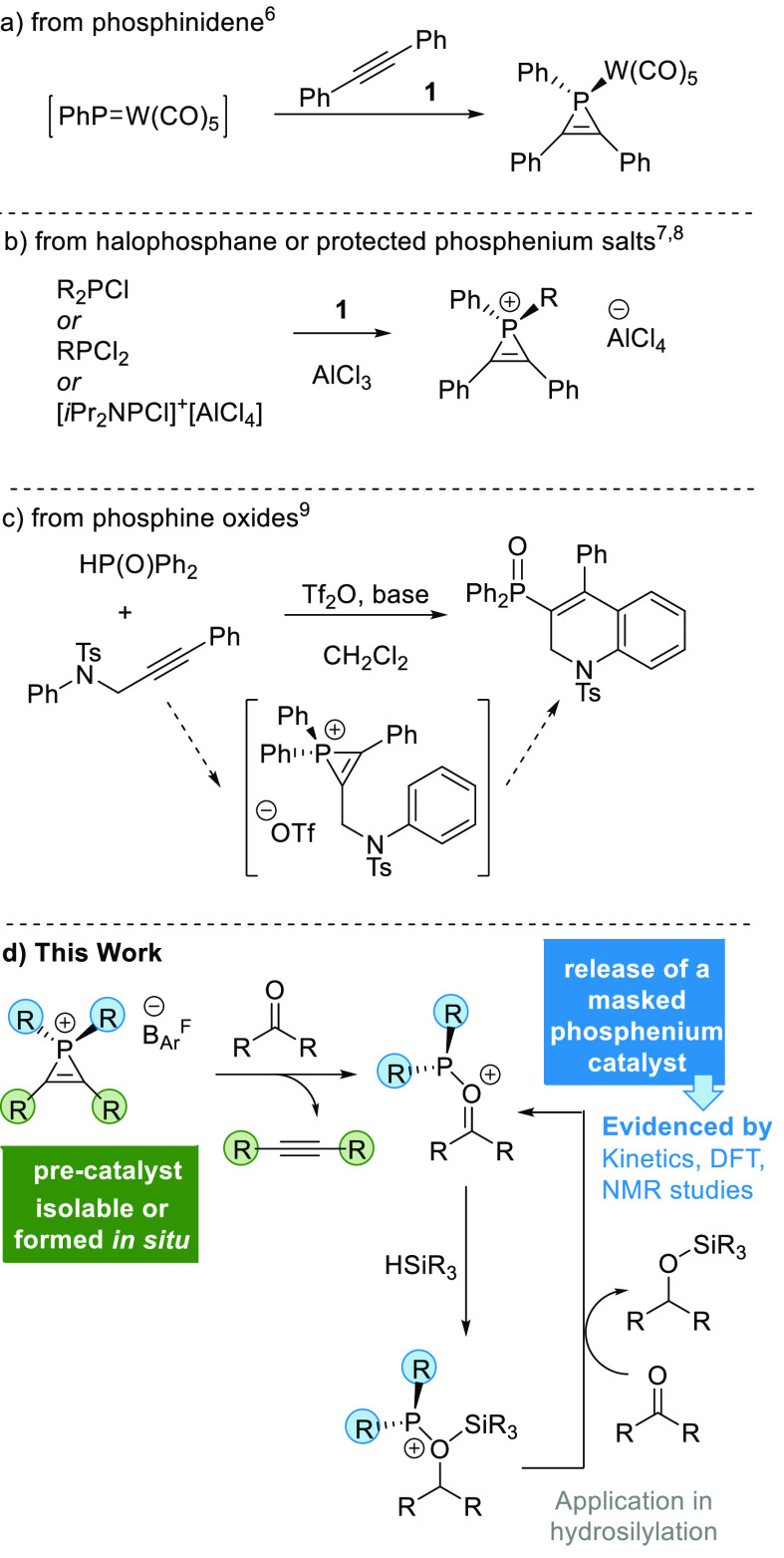
(a,b) Different Approaches to the Synthesis of Phosphirenium Ions; (c) Functionalization via a Phosphirenium Ion; (d) This Work, where Mechanistic Investigations Indicate that a Phosphirenium Pre-catalyst Acts as a Masked Phosphenium Ion, Which is Itself the Active Catalyst

The chemistry of the three-membered phosphacycle, the phosphirenium ion, first appeared in the literature at the beginning of the 1980s,^1^ along with a number of theoretical studies that considered their stabilization by σ*-aromaticity.^2^ The chemistry to date is dominated by the use of these species as reagents in synthesis; exploitation of their catalytic Lewis acidic properties has yet to be reported. The first example of a stabilized three-membered phosphirene–tungsten ring was reported by Marinetti and Mathey in 1982^3^ (Scheme 1a). A year later, Hogeveen and Breslow reported and characterized phosphirenium salts by formal addition of halophosphines to alkynes,^4^ a method that was further improved using amine-phosphenium ions [*i*Pr_2_NPCl]^+^[AlCl_4_]^−^ (Scheme 1b).^5^ Other routes for their synthesis or methods for structural elaboration have been developed,^6−8^ but it was only in 2017, when Hirano and Miura reported the synthesis of highly functionalized organophosphines postulated to proceed via a phosphirenium intermediate, that the application in practical organic synthesis was reported (Scheme 1c).^9^ They have since expanded their methodology to the synthesis of dibenzophospholes,^10,11^ while other groups have reported functionalization of phosphine oxides in the presence of Tf_2_O.^12^

Phosphirenium ions pose an interesting mechanistic challenge: they possess both electrophilic phosphorus and a low-lying π*-acceptor centered on the highly activated carbon–carbon double bond in the ring. Both could behave as Lewis acidic sites for the activation of organic substrates and so possess exciting opportunities for selective or divergent reactivity. However, we herein report on the unexpected ability of phosphirenium ions to undergo ring opening and subsequent alkyne displacement via nucleophilic trapping of a phosphenium ion {Ph_2_P^+^} by carbonyl reagents (Scheme 1d). Onward reaction with a silane and a further equivalent of carbonyl releases the reduced carbonyl product and regenerates the highly active phosphenium adduct, which is the active catalyst. Divalent phosphenium ions are highly elusive species, relying on lone pair stabilization of the empty phosphorus p-orbital by adjacent heteroatoms (e.g., N- or P-donors) without which they are challenging to detect and characterize.^13^ It is worth noting that divalent phosphenium species, in the form of N-heterocyclic phosphenium ions, have recently started to gather interest,^14^ not least due to their catalytic capabilities, which are as yet unproven with a simple {Ar_2_P^+^} catalyst. Here, our synergistic experimental and computational approach strongly supports our assertion of easy-to-prepare and -handle phosphirenium ions acting as a masked phosphenium catalyst of the form {Ph_2_P^+^}, stabilized by a Lewis basic carbonyl oxygen atom. An advantage of the work reported here is the ease of synthesis, with a range of phosphirenium pre-catalysts being readily prepared or formed in situ for use in catalysis.

## 2 Results and Discussion

### 2.1 Synthesis and Characterization of Phosphirenium Ions

Due to the practicality of the reaction, Hirano and Miura’s procedure (where the phosphirenium ion was detected but not isolated)^9,11,15^ was employed for the synthesis of phosphirenium ion **3·OTf** (Table 1, entry 1). Formation of **3·OTf** is observed by following the reaction via ^31^P{^1^H} NMR spectroscopy, where a characteristic peak at −108.3 ppm is obtained.^9^ To optimize the protocol, different activating agents were reacted with diphenyl acetylene **1** and secondary phosphine oxide (SPO) **2** in CDCl_3_. **3·OTf** does not form when using triflate salts, such as silver or iron triflates (entries 2 and 3), or by Brønsted acid activation with triflic acid^16^ (entry 4) or activated triflic acid (entry 5). Acetic anhydride does activate **2**, with the formation of Ph_2_P(OAc)^17^ and Ph_2_P(O)PPh_2_.^18^ However, these species do not react further with **1** (entry 6). Di-*tert*-butyl dicarbonate (Boc_2_O) is not effective for this transformation, and even at higher temperatures, **1** and **2** are recovered (entry 7). Following a brief solvent and temperature screening,^19^ the optimal conditions were determined to be CH_3_CN at 60 °C for 30 min, which give full conversion to **3·OTf** (entry 8).

**Table 1:**
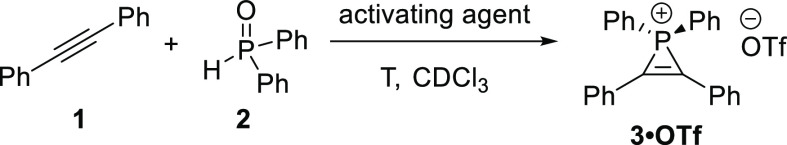
Optimization of the Synthesis of Phosphirenium Ions^a^

entry	activating agent	*T* (°C)	conversion to **3·OTf**
1	Tf_2_O	80	60%
2	Fe(OTf)_2_	80	0
3	AgOTf	80	0
4	HOTf	80	0
5	HOTf/PhSiH_3_	80	Ph_2_PH (major)
6	Ac_2_O	80	several products^b^
7	O(CO_2_*t*Bu)_2_	80	0
**8**	**Tf**_**2**_**O**	**60**	>99%^c^ (98%^d^)

a Reaction conditions **1** (0.2 mmol), **2** (0.2 mmol), activating agent (0.2 mmol), and CDCl_3_ (0.4 mM).

b See the Supporting Information.

c CD_3_CN.

d Spectroscopic yield analyzed by inverse-gated ^31^P{^1^H} NMR spectroscopy with PPh_3_ as an internal standard.

**Figure 1:**
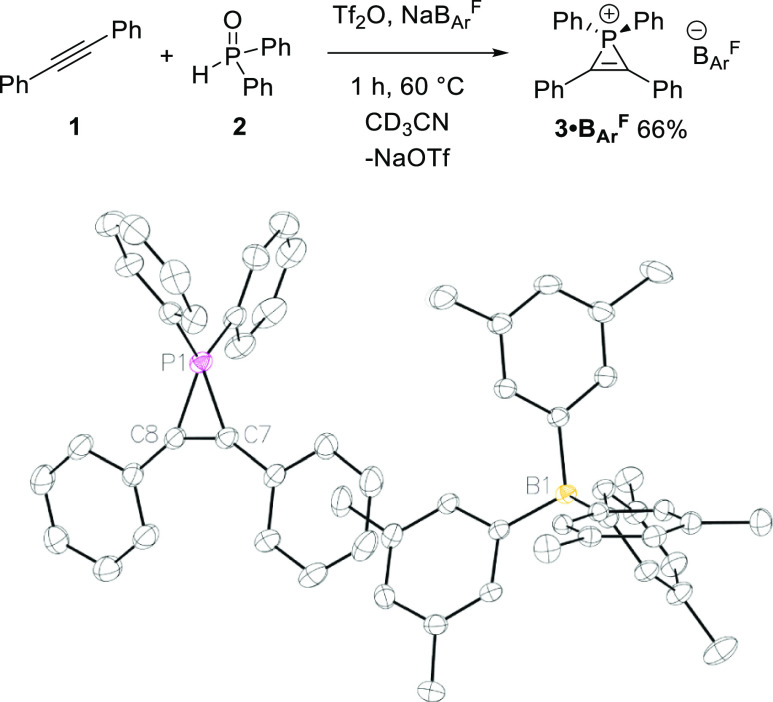
Counterion exchange at the phosphirenium ion. Isolated yield presented. ORTEP view of **3·B**_**Ar**_^**F**^ showing a thermal ellipsoid at 30% probability levels. H, F, and minor components of disordered anion C atoms were omitted for clarity.^23^

Adding 1 equiv of NaB_Ar_^F^ to the optimized reaction mixture of **1**, **2**, and Tf_2_O in CD_3_CN forms **3·B**_**Ar**_^**F**^, which is isolated in 66% yield (Figure 1). A small downfield shift is observed in the ^31^P{^1^H} NMR spectrum from −108.3 ppm for **3·OTf** to −109.1 ppm for **3·B**_**Ar**_^**F**^; the disappearance of the triflate peak at −79.3 ppm is observed by ^19^F NMR spectroscopy and substituted by a ^–^B_Ar_^F^ counterion at −62.5 ppm. Counterion exchange is not observed when using sodium or silver salts, such as NaBF_4_ or AgNTf_2,_ and **3·OTf** is recovered; with AgSbF_6_ and AgBF_4_, decomposition of the starting material is observed via ^31^P{^1^H}, ^19^F, and ^1^H NMR spectroscopy.^20^ The structure of **3·B**_**Ar**_^**F**^ was confirmed by single-crystal X-ray diffraction analysis. The bond metrics associated with this structure are in line with other examples of phosphirenium cations, for example, the ring P–C distances are 1.748(2) and 1.746(3) Å, and the C=C bond distance is 1.340(4) Å; both are similar to reports from Mathey, Wild, and Cowley.^3,6,7,21^ The alternating single and double bonds show that the structure depicted is the dominant resonance form of the phosphirenium ion in the solid state. This species represents the first isolated phosphirenium ion with a borate counterion.^22^

**Figure 2:**
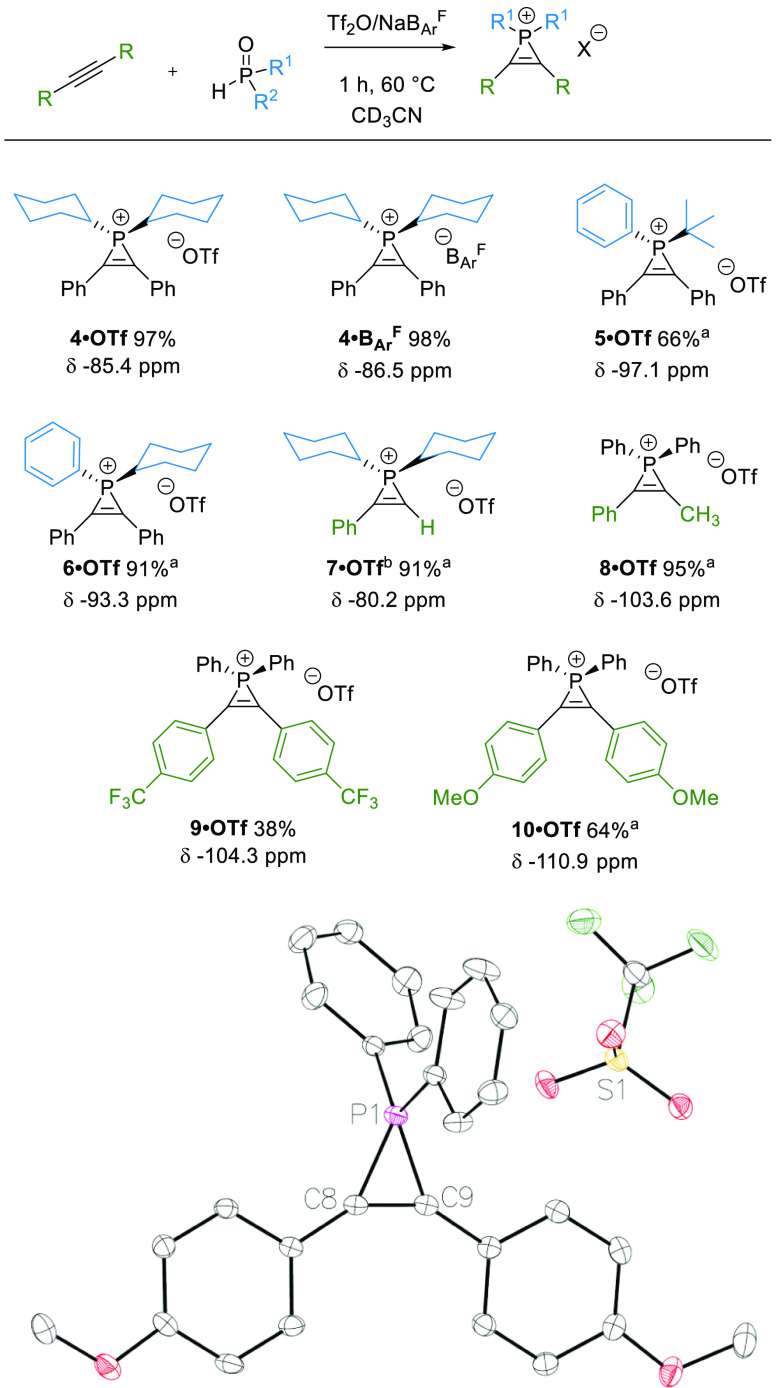
Synthesis of phosphirenium ions; alkyne (0.2–0.5 mmol), phosphine oxide (0.2–0.5 mmol), Tf_2_O (0.2–0.5 mmol), NaB_Ar_^F^ (0.2–0.5 mmol), CD_3_CN, 60 °C. Isolated yield presented; ^a^spectroscopic yield analyzed by inverse-gated ^31^P{^1^H} NMR spectroscopy with PPh_3_ as an internal standard; ^b^CD_3_Cl. ORTEP view of **10·OTf** showing a thermal ellipsoid at 30% probability levels. H atoms were omitted for clarity.^27^

With the optimized conditions in hand, the generality of the methodology was explored (Figure 2). Some of the phosphirenium ions were very sensitive and could only be characterized in situ (**5·OTf**, **6·OTf**, **7·OTf**, **8·OTf**, and **10·OTf** proved particularly challenging; see the Supporting Information for spectral data). The use of different substituted SPOs, bearing bearing aryl or alkyl groups, is well tolerated. For example, using Cy_2_P(O)H, **4·OTf** and **4·B**_**Ar**_^**F**^ are isolated in high yields (97 and 98%, respectively). These species display downfield ^31^P{^1^H} NMR signals compared to **3**, with a singlet at −85.4 and −86.5 ppm, respectively, due to the increased electron donation of the alkyl substituents. There is only a very modest difference in the ^31^P chemical shift observed between the ions bearing different anions, **3·OTf** versus **3·B**_**Ar**_^**F**^ Δδ = 0.8 and **4·OTf** versus **4·B**_**Ar**_^**F**^ Δδ = 1.1 ppm, which is as expected given the non-coordinating nature of the counterions.

Mixed SPOs bearing alkyl and phenyl groups, such as *t*BuPhP(O)H and CyPhP(O)H, are also well tolerated with full conversion after 1 h to form **5·OTf**(24) and **6·OTf**, with ^31^P{^1^H} NMR signals at −97.1 and −93.3 ppm, respectively. Varying the alkyne was also explored; phenyl acetylene and Cy_2_P(O)H lead to full conversion to **7·OTf** with a phosphorus signal at −80.2 ppm and a downfield signal for the terminal C–H at 8.4 ppm. For all phosphirenium species presented, there are deshielded signals in the proton and carbon NMR spectra, which can be explained by the resonance structure of the phosphirenium ion.^25,26^ 1-Phenyl-1-propyne reacts well with **2** to form **8·OTf** with a ^31^P signal at −103.6 ppm. Further functionalization of the alkyne is tolerated with **9·OTf** (38%) and **10·OTf** (64%) isolated using the optimized conditions. The phosphorus signals for **9·OTf** and **10·OTf** appear at δ −104.3 and −110.9 ppm, respectively, versus −108.3 ppm for **3·OTf**; thus, by this measure, the alkyne substituents only have a minor impact on P^+^ electronics, but as we show below, the same substituents have a dramatic effect on catalytic reactivity. Crystallization is not limited to B_Ar_^F^ counterions, and X-ray single-crystal analysis of **10·OTf** confirms the three-membered structure of this phosphirenium species (Figure 2, bottom), with similar metrics as observed for **3**. Short contacts between the counterion and the phosphirenium substituents are observed, although no direct P···O interaction was found, probably due to the steric effect of the two phenyl rings compared to, for example, the mixed Ph and Me system reported by Wild.^7^

### 2.2 Hydrosilylation Optimization

With these phosphirenium species in hand, we explored the use of phosphirenium ions as pre-catalysts for the hydrosilylation of aldehydes and ketones. We opted for this transformation because it would allow comprehensive mechanistic investigations using a range of experimental techniques.

Initial studies were conducted with 10 mol % phosphirenium species **4·B**_**Ar**_^**F**^, prepared and activated in situ prior to the addition of the substrates, benzophenone **11** and dimethylphenyl silane **12**, in an equimolar ratio. The reaction was monitored by ^1^H NMR spectroscopy and examined after 18 h of reaction at r.t. (Table 2). Initial results were promising with 44% total conversion of **11** (entry 1) as 28% **13a** and 16% **14a**. By increasing the temperature to 80 °C (entry 2), full conversion of the starting material is observed with 84% formation of **13a**, while 16% **14a** persists. Using 10 mol % isolated **4·B**_**Ar**_^**F**^, lower conversions (73%) are observed after 18 h (entry 3), with 58% **13a**. Isolated pre-catalyst **3·B**_**Ar**_^**F**^ shows higher reactivity, with increased conversion to **14a** versus **13a** at 10 mol % loading (entry 5). When phosphirenium triflate species are used, such as **3·OTf** or **4·OTf**, only deoxygenation occurs with full conversion into **14a**; this is due to hydrogen release from silane reacting with co-catalytic TfOH (formed from Tf_2_O).^28^ Thus, in line with Miura’s recent studies on phosphirenium-derived heterocycles,^9,11^ we also note that the use of a base is crucial to quench TfOH released during pre-catalyst formation and therefore increase the selectivity toward **13a**. See Table S2 in the Supporting Information where comprehensive benchmarking and optimization were undertaken demonstrating that not only does TfOH lead exclusively to the deoxygenation product **14a** but also that superior hydrosilylation catalysis takes place using a phosphirenium pre-catalyst compared to the equivalent reaction using TfOPPh_2_. A total of 5 mol % 2,6-lutidine was added to 5 mol % in situ-prepared pre-catalyst **4·B**_**Ar**_^**F**^ prior to addition of substrates **11** and **12**, and the selective formation of **13a** is observed (5 h, 80 °C, CD_3_CN, entry 5). Changing to **3·B**_**Ar**_^**F**^, with 5 mol % 2,6-lutidine, gives good reactivity at lower temperature (entry 6), and further improvement is seen with the electron-poor phosphirenium species **9·B**_**Ar**_^**F**^, for which reaction with 5 mol % 2,6-lutidine leads to full conversion into **13a** at 60 °C after only 1 h (entry 7). In contrast, electron-rich **10·B**_**Ar**_^**F**^ reacted at a slower rate with full conversion to the hydrosilylation product requiring 58 h at 80 °C (entry 8). Clearly the electronics of the alkyne used to prepare the phosphirenium pre-catalyst play a substantial role in catalytic competency. Screening of the base with 10 mol % **3·B**_**Ar**_^**F**^ as the catalyst of choice shows lower conversion to **13a** when using NEt_3_ and pyridine (entry 9 and 10) with 59 and 52%, respectively, and recovery of the starting materials with K_2_CO_3_ (entry 11) after 18 h at 80 °C. These results show that a slightly bulkier, aromatic organic base like 2,6-lutidine is most effective.^29^

**Table 2:**
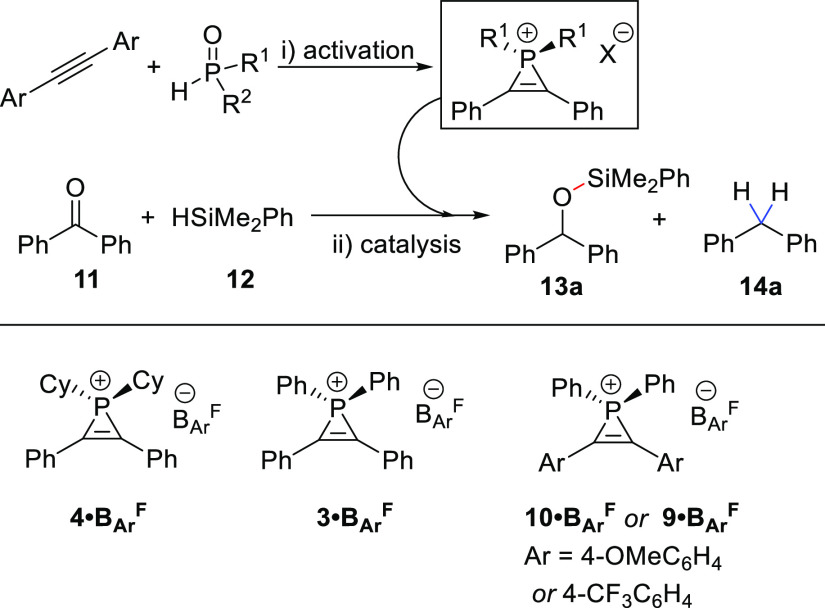
Optimization Reduction of Carbonyls with Silanes^a^

entry	catalyst (mol %)	temp.	**13a**^b^	**14a**^b^
1	**4·B**_**Ar**_^**F**^ (10)	r.t.	28	16
2	**4·B**_**Ar**_^**F**^ (10)	80 °C	84	16
3^b^	**4·B**_**Ar**_^**F**^ (10)	80 °C	58	15
4	**3·B**_**Ar**_^**F**^ (10)	80 °C	21	75
5^b^^,^^c^	**3·B**_**Ar**_^**F**^ (10)	80 °C	>99	
5^c^	**4·B**_**Ar**_^**F**^ (5) + 2,6-lutidine (5)	80 °C	>99	
6^c^	**3·B**_**Ar**_^**F**^ (5) + 2,6-lutidine (5)	60 °C	>99	
7^d^	**9·B**_**Ar**_^**F**^ (5) + 2,6-lutidine (5)	60 °C	>99	
8^e^	**10·B**_**Ar**_^**F**^ (5) + 2,6-lutidine (5)	80 °C		
9	**3·B**_**Ar**_^**F**^ (10) + NEt_3_ (10)	80 °C	59	
10	**3·B**_**Ar**_^**F**^ (10) + pyridine (10)	80 °C	52	
11	**3·B**_**Ar**_^**F**^ (10) + K_2_CO_3_ (10)	80 °C		

a Reaction condition: (i) activation step R_2_P(O)H (5–10 mol %), Tf_2_O (5–10 mol %), NaB_Ar_^F^ (5–10 mol %), **1** (5–10 mol %), CD_3_CN (0.41 M), 60 °C, and 30 min; (ii) benzophenone **11** (0.25 mmol), dimethylphenyl silane **12** (0.25 mmol), 18 h, and conversion.

b Isolated pre-catalyst.

c 5 h.

d 1 h.

e >99% after 58 h.

### 2.3 Mechanistic Studies

**Scheme 2:**
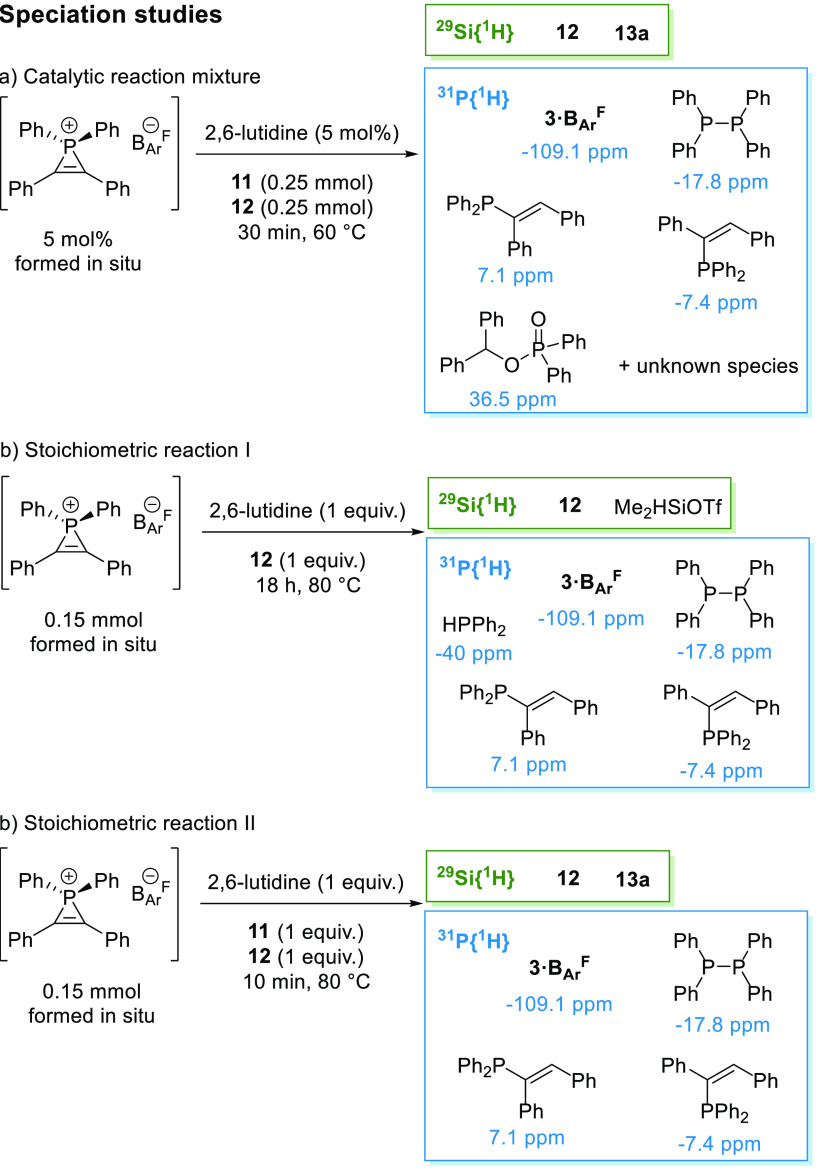
^29^Si{^1^H} and ^31^P{^1^H} NMR Spectroscopy Speciation Studies of (a) Standard Catalytic Reaction of **3·B**_**Ar**_^**F**^, 2,6-Lutidine, **11**, and **12**; (b) Stoichiometric Reaction of **3·B**_**Ar**_^**F**^, 2,6-Lutidine and **12**; and (c) Stoichiometric Reaction of **3·B**_**Ar**_^**F**^, 2,6-Lutidine, **11**, and **12**

We initiated our mechanistic investigations by analyzing the catalytic reaction mixture, using in situ-prepared **3·B**_**Ar**_^**F**^, by NMR spectroscopy (Scheme 2a). After 30 min, our standard reaction in CD_3_CN only shows the starting material (H_2_SiMePh **12**, −16.9 ppm) and product (**13a**, 8.3 ppm) in the ^29^Si{^1^H} NMR spectrum. However, a plethora of species are observed in the ^31^P{^1^H} NMR spectrum, with the major species being **3·B**_**Ar**_^**F**^ at −108.5 ppm, along with P_2_Ph_4_ at −17.8 ppm, (*E*)-(1,2-diphenylvinyl)diphenylphosphine at 7.1 ppm,^9^ smaller amounts of (*Z*)-(1,2-diphenylvinyl)diphenylphosphine at −7.4 ppm,^30^ and benzhydryl diphenylphosphinate at 36.5 ppm.^31^ While phosphines are known to facilitate hydrofunctionalization of carbonyls,^32^ noteworthy is the lack of catalytic activity observed using 10 mol % HPPh_2_ or P_2_Ph_4_.

A stoichiometric reaction to form **3·B**_**Ar**_^**F**^ with subsequent addition of 2,6-lutidine and **12** results in two new species by ^29^Si{^1^H} NMR spectroscopy. The major species is identified as Me_2_SiHOTf at 23 ppm, formed by the facile cleavage of the silane phenyl group.^33^ Analysis of the mixture by ^31^P{^1^H} NMR spectroscopy shows **3·B**_**Ar**_^**F**^, P_2_Ph_4_, and vinyl phosphine as per the catalytic reaction, along with HPPh_2_ with its characteristic peak at −40 ppm (Scheme 2b). Finally, when performing the same stoichiometric study in the presence of benzophenone **11** and analyzing the mixture after 10 min by ^29^Si{^1^H} NMR spectroscopy, we observe **13a** at 8.3 ppm but no traces of silyl triflate (Me_2_SiHOTf or Me_2_PhSiOTf).^33^ The latter mixture was also analyzed by ^31^P{^1^H} NMR spectroscopy, which shows the presence of **3·B**_**Ar**_^**F**^, vinyl phosphine, and P_2_Ph_4_, but no traces of HPPh_2_ are visible at any point of the analysis (Scheme 2c).

When performing hydrosilylation to form **13a** using 10 mol % **9·B**_**Ar**_^**F**^ pre-catalyst and 2,6-lutidine, we also observe the formation of substituted vinyl phosphine [i.e., (*E*)-(1,2-bis(4-(trifluoromethyl)phenyl)vinyl)diphenylphosphine] in situ. Hydrosilylation to form **13a** using 10 mol % **10·B**_**Ar**_^**F**^ pre-catalyst and 2,6-lutidine gives a complex array of peaks in the ^31^P NMR spectrum, making identification difficult although likely including vinyl phosphines.

The use of 20 mol % (*E*)-(1,2-diphenylvinyl)diphenylphosphine and 20 mol % 2,6-lutidine in a catalytic reaction of **11** and **12** does not give an appreciable quantity of **13a** after 18 h at 80 C, indicating that the vinyl phosphine is not an active catalyst in the hydrosilylation reaction, and this is further supported by computational studies.^34^

**Figure 3:**
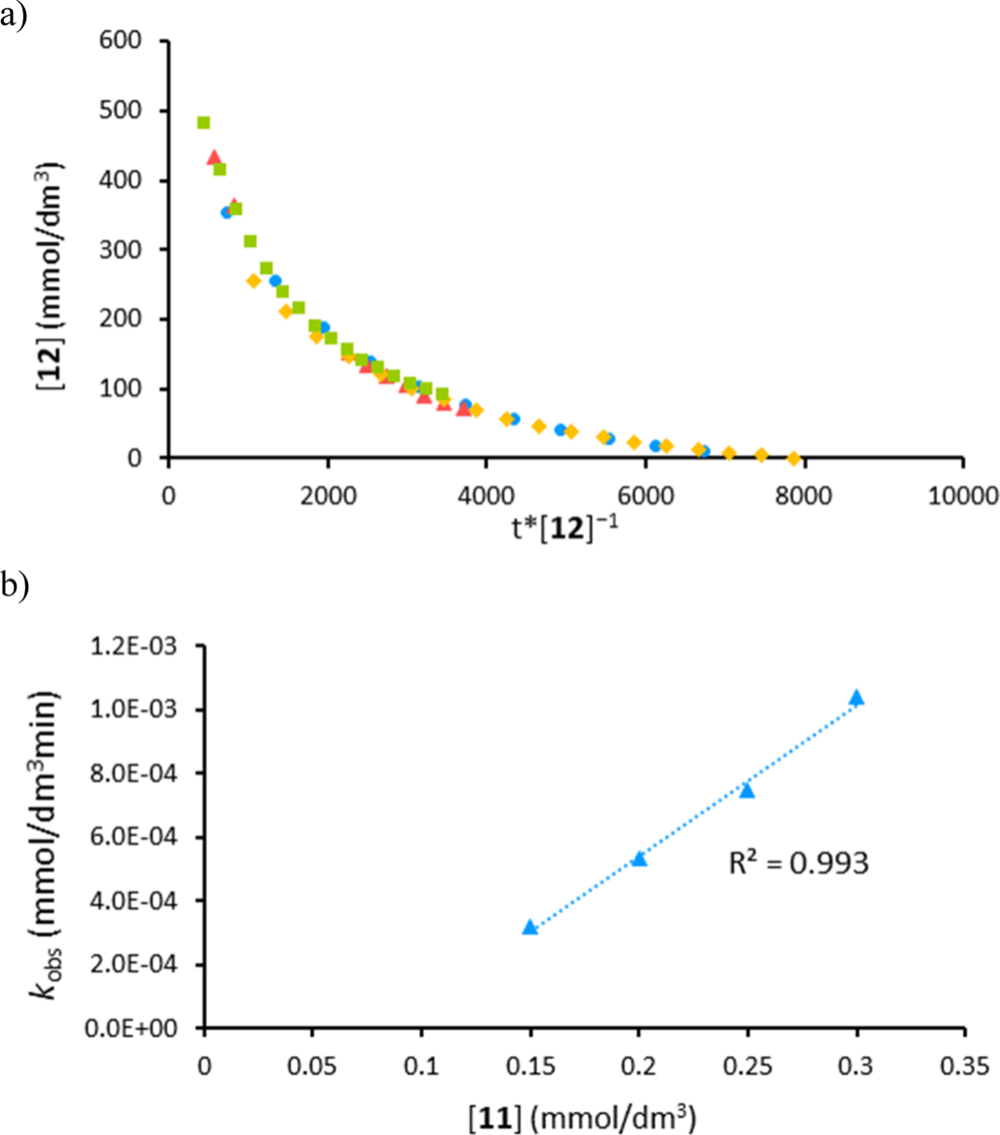
a) Graphical analysis showing inverse order relationship for the formation of **13a** at different phenyldimethylsilane **12** loadings (yellow ◆ 0.75, blue ● 1, red ◆ 1.25, and green ■ 1.5 equiv); (b) plot showing first-order dependence for the formation of **13a** at different benzophenone **11** loadings (0.75, 1, 1.25, and 1.5 equiv); and reaction conditions: (i) activation step Ph_2_P(O)H **2** (10 mol %), Tf_2_O (10 mol %), NaB_Ar_^F^ (10 mol %), diphenylacetylene **1** (10 mol %), CD_3_CN (0.4 M), 60 °C, and 30 min and (ii) 2,6-lutidine (10 mol %), ketone or aldehyde (0.20 mmol), silane (0.20 mmol), 80 °C, and PhMe used as an internal standard.

Next, we undertook kinetic studies using NMR spectroscopic monitoring. By pre-forming the catalyst in situ, we have analyzed the order in substrates **11** and **12** by varying the amount of the starting material using 10 mol % **3·B**_**Ar**_^**F**^. The data obtained suggest first-order dependence in **11**, while an inverse order is observed for **12**(35) (Figure 3). Product inhibition is not observed, with similar reaction rates obtained when adding 0.5 equiv of product **13a** to the catalytic reaction of **11** and **12** in a 1:1 mixture (5.32 × 10^–4^ vs 5.63 × 10^–4^ mmol/dm^3^ min). Therefore, the negative order in silane can be attributed to silane involvement in side-product formation or catalyst degradation. Comparable data are obtained when using isolated pre-catalyst **3·B**_**Ar**_^**F**^ in the absence of 2,6-lutidine, further supporting the assertion that the role of 2,6-lutidine is simply to mop up TfOH released on formation of **3·B**_**Ar**_^**F**^ from reaction of Tf_2_O with alkyne, SPO, and NaB_Ar_^F^.^26^ It is important to note that the kinetic data found for this transformation are in contrast with Piers’ hydrosilylation-type activation via Lewis acids [e.g., B(C_6_F_5_)_3_],^36^ where the increase in ketone concentration slows down the reaction. Indeed, interrogating the ^11^B and ^19^F NMR spectra from catalytic reactions also shows that only borate is present in solution; no borane signals are observed, and so we feel that borane-mediated hydrosilylation is unlikely in this case.

At this stage, we have three distinct pieces of experimental data that necessitate further investigation:

(i) Observation of vinyl phosphine both during catalysis and in stoichiometric studies indicates that phosphirenium ring opening could be involved, either as a catalyst deactivation process or on-cycle ring-opening/ring-closure sequence;
(ii) The inverse order in silane indicates that there is a catalytically inactive pathway dominating at higher silane loadings.
(iii) Phosphirenium ions with different electronic substituents have very different capabilities in hydrosilylation (**9·B**_**Ar**_^**F**^ > **3·B**_**Ar**_^**F**^ ≫ **10·B**_**Ar**_^**F**^), but these changes in electronics only have a modest impact on the phosphorus center (e.g., compare **3·OTf**, **9·OTf**, and **10·OTf**^31^P NMR chemical shift). We therefore believe that the role of the phosphirenium pre-catalyst is not merely to act as a simple Lewis acid.^37^

**Figure 4:**
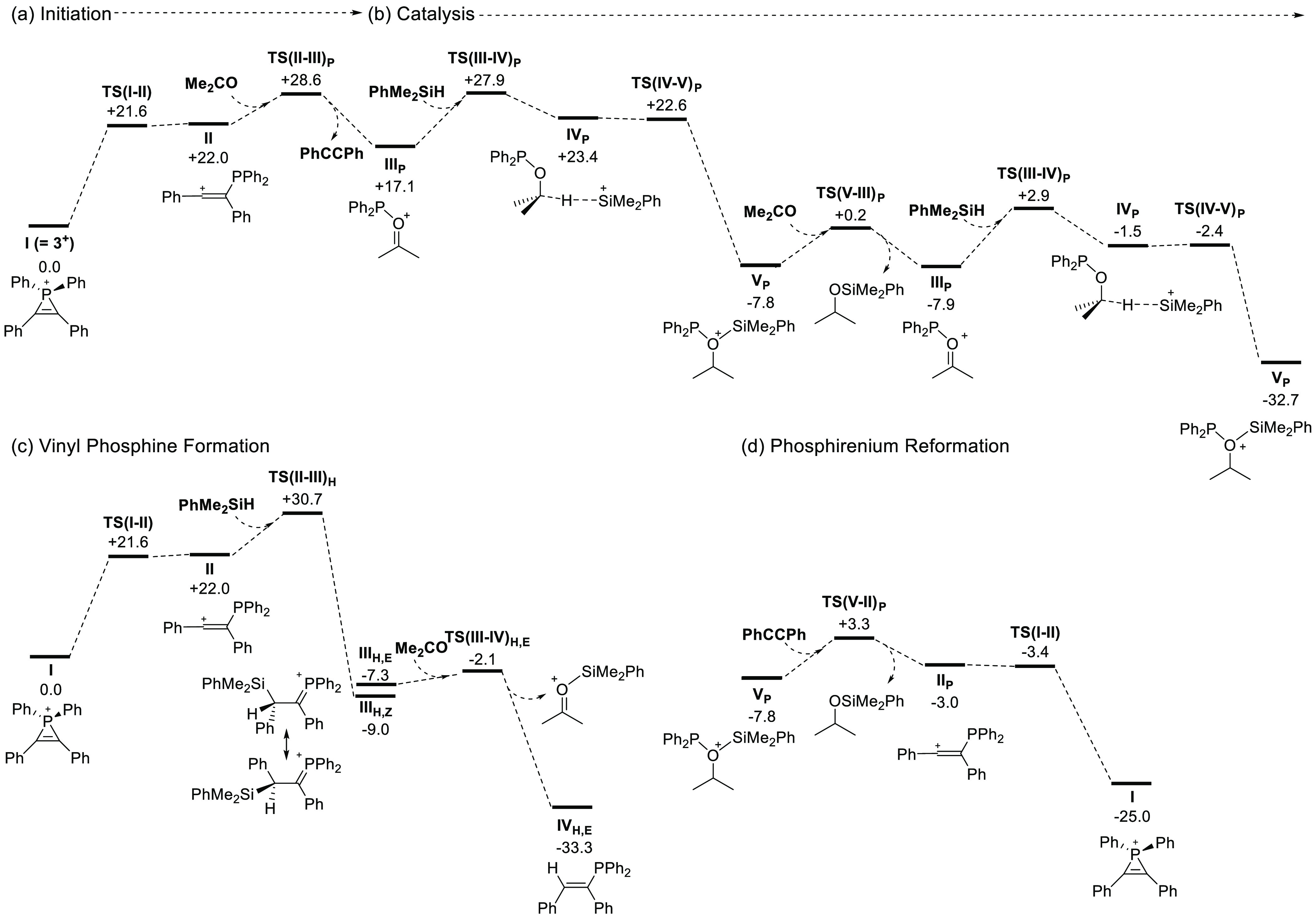
Free energy reaction profiles (kcal/mol) for the reactions of Me_2_PhSiH with acetone mediated by the phosphirenium cation **3**^**+**^ computed at the M052X-D3(CH_3_CN)/def2-TZVP//TPSS/def2-SVP level of theory. (a) Initiation via ketone attack at P in the ring-opened ethenylium cation, **II**; (b) catalytic cycle based on intermediate **III**_**P**_; (c) competing vinyl phosphine formation; and (d) competing phosphirenium reformation.

With these experimental results in hand, we undertook a computational study of the reactions of the phosphirenium cation, **3**^**+**^ (denoted as **I** in the computational study) with acetone and Me_2_PhSiH. Initial studies considered the direct nucleophilic attack of either the ketone or silane at the phosphirenium ring;^29^ however, the characterization of these processes indicated that a ring-opening step occurs first to form an ethenylium intermediate **II** at +22.0 kcal/mol. From **II**, catalysis is initiated via the nucleophilic attack of the ketone at the P center (see Figure 4a). This proceeds through **TS(II–III)**_**P**_ at +28.6 kcal/mol and results in both P–O bond formation and displacement of the alkyne to form a phosphenium-ketone adduct **III**_**P**_. Catalysis now proceeds (Figure 4b) via Si–H addition across the C=O moiety [**TS(III–IV)**_**P**_, +27.9 kcal/mol] to form **IV**_**P**_, in which partial hydride transfer has occurred (C···H = 1.29 Å; H···Si = 1.71 Å). Silyl group migration then readily occurs to form **V**_**P**_ at −7.8 kcal/mol, featuring a silylalkyloxonium adduct of the {Ph_2_P} moiety. Ketone attack at **V**_**P**_ then displaces the hydrosilylation product and generates **III**_**P**_ that can then enter a second cycle with further reaction with silane.^38^

Overall, the calculations imply a relatively slow initiation process via **TS(II–III)**_**P**_ at 28.6 kcal/mol to form **III**_**P**_, from which catalysis can then proceed with an energy span of only 10.8 kcal/mol. Catalysis from **III**_**P**_ should therefore be very efficient; however, the situation is complicated by at least two possible competing reactions. The first of these is vinyl phosphine formation (Figure 4c). This involves silane attack at the C1 position of intermediate **II** and proceeds via **TS(II–III)**_**H**_ at +30.7 kcal/mol with net insertion into the Si–H bond to give silylalkylphosphonium ion **III**_**H,Z**_ at −9.0 kcal/mol, featuring a short P–C bond (1.76 Å). Assuming facile rotation about the C–C bond leads to **III**_**H,E**_ (−7.3 kcal/mol), which can react with ketone with displacement of a silylether cation to form *E*-vinyl phosphine **IV**_**H,E**_ (−33.3 kcal/mol).^39^ In principle, **IV**_**H,E**_ could re-enter catalysis via hydride transfer to the silylether cation, generating the hydrosilylation product and intermediate **II**. However, this process has a computed barrier of 48.3 kcal/mol, and so the formation of vinyl phosphine represents an unproductive off-cycle process. The second competing process (Figure 4d) involves the reaction of **V**_**P**_ with alkyne via **TS(V–II)**_**P**_ at +3.3 kcal/mol with the release of the hydrosilylation product and formation of **II**. **II** can then rapidly undergo ring closure to reform **I**.

The significance of these two competing processes is captured in the free energy differences (ΔΔG) between the rate-limiting transition states. For vinyl phosphine formation, these are **TS(II–III)**_**H**_ and **TS(II–III)**_**P**_, where the latter is more stable and ΔΔG_1_ = +2.1 kcal/mol. The productive onward reaction to form **III**_**P**_ should therefore dominate; however, the small energy difference suggests that vinyl phosphine formation may be significant at higher silane concentrations, and this is consistent with the experimental identification of an inverse first-order reaction with respect to silane. For the competing reactions of **V**_**P**_ with either the ketone or the alkyne, the key transition states are **TS(III–IV)**_**P**_ and **TS(V–II)**_**P**_, respectively. In this case, **TS(V–II)**_**P**_ is only marginally disfavored with ΔΔG_2_ = +0.4 kcal/mol, and this small energy difference suggests reaction with alkyne and therefore the reformation of **I** will be competitive. However, unlike the vinyl phosphine **IV**_**E**_, **I** remains a competent catalyst precursor, albeit one that reacts with a relatively high overall barrier of 28.6 kcal/mol. There is therefore an ensemble of processes at play, in which efficient catalysis via **III**_**P**_ is balanced by catalyst loss through vinyl phosphine formation and a less efficient cycle based on phosphirenium precursor **I**. The net effect of these various processes is qualitatively consistent with a reaction that requires several hours at 60 °C to reach full conversion.

#### 2.3.1 Substituent Effects

**Figure 5:**
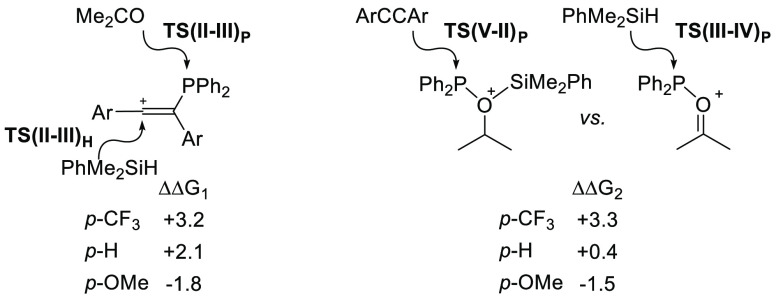
Comparison of key transition states (free energies, kcal/mol) for the competing processes outlined in Figure 4. ΔΔG_1_ = **TS(II–III)**_**H**_ – **TS(II–III)**_**P**_ and ΔΔG_2_ = **TS(V–II)**_**P**_ – **TS(III–IV)**_**P**_.

Reaction profiles computed for cations **9**^**+**^ and **10**^**+**^ were similar to those for the parent species **3**^**+**^ (see the Supporting Information). However, subtle changes in the relative energies of the key competing transition states are computed that have a significant impact on the behavior of these systems (see Figure 5). For **9**^**+**^, the electron-withdrawing *p*-CF_3_ substituents cause an increase in both ΔΔG_1_ and ΔΔG_2_. As a result, catalysis becomes more efficient both in terms of a reduced likelihood of vinyl phosphine formation and a lower propensity to reform the phosphirenium cation. This is reflected in the short (ca. 1 h) times to full conversion seen experimentally with **9**^**+**^. In contrast, the electron-donating *p*-OMe substituents switch the preference toward vinyl phosphine formation from **II** (ΔΔG_1_ = −1.8 kcal/mol) and favoring the reaction of **V**_**P**_ with alkyne that leads to the formation of **I** over the more direct catalytic reaction with ketone. Both factors should render catalysis less efficient, consistent with the very sluggish catalytic reaction that is observed with **10**^**+**^ (58 h to full conversion). The calculations indicate that while the *p*-OMe substituents in **10**^**+**^ promote ring opening [ΔG_**I**→**II**_ = +17.6 kcal/mol (*p*-OMe), +22.0 kcal/mol (*p*-H), and +24.1 kcal/mol (*p*-CF_3_)], they also disfavor the subsequent {Ph_2_P^+^} transfer via **TS(II–III)**_**P**_, and this results in hydride transfer via **TS(II–III)**_**H**_ dominating.

These combined experimental and computational studies allow us to propose a reaction mechanism for the hydrosilylation of carbonyls catalyzed by phosphirenium cations. These species act as a masked phosphenium source that is accessed via ring opening. Productive turnover then involves associative phosphenium transfer to the ketone, Si–H addition over the C=O bond, and associative displacement of the hydrosilylation product with further ketone. Spectroscopic and computational insights show that the formation of vinyl phosphine is an irreversible off-cycle process that will sequester the {Ph_2_P^+^} moiety through reaction with silane, in accordance with inverse order kinetics. Para-substituents on the phosphirenium aryl groups can significantly impact the behavior; electron-donating *p*-OMe substituents undermine catalysis by favoring the formation of side products.

Experimentally, the observation of HPPh_2_ and P_2_Ph_4_ by in situ reaction monitoring also supports the presence of the ring-opened form in the catalytic reaction. Given the capricious nature of unstabilized phosphenium ions, we do not observe free {Ph_2_P^+^} in the catalytic reaction. Calculations indicate that loss of {Ph_2_P^+^} from the parent phosphirenium is uphill by 34 kcal/mol; thus, observation of the free species is unlikely, lending further support to it being transferred directly between nucleophiles during the cycle. Catalytic reactions do show the presence of a phosphinate peak in the ^31^P NMR spectrum (−36.5 ppm, corresponding to benzhydryl diphenyl phosphinate^31^), which gives tentative support for the presence of phosphenium adducts.

### 2.4 Hydrosilylation Substrate Scope

**Figure 6:**
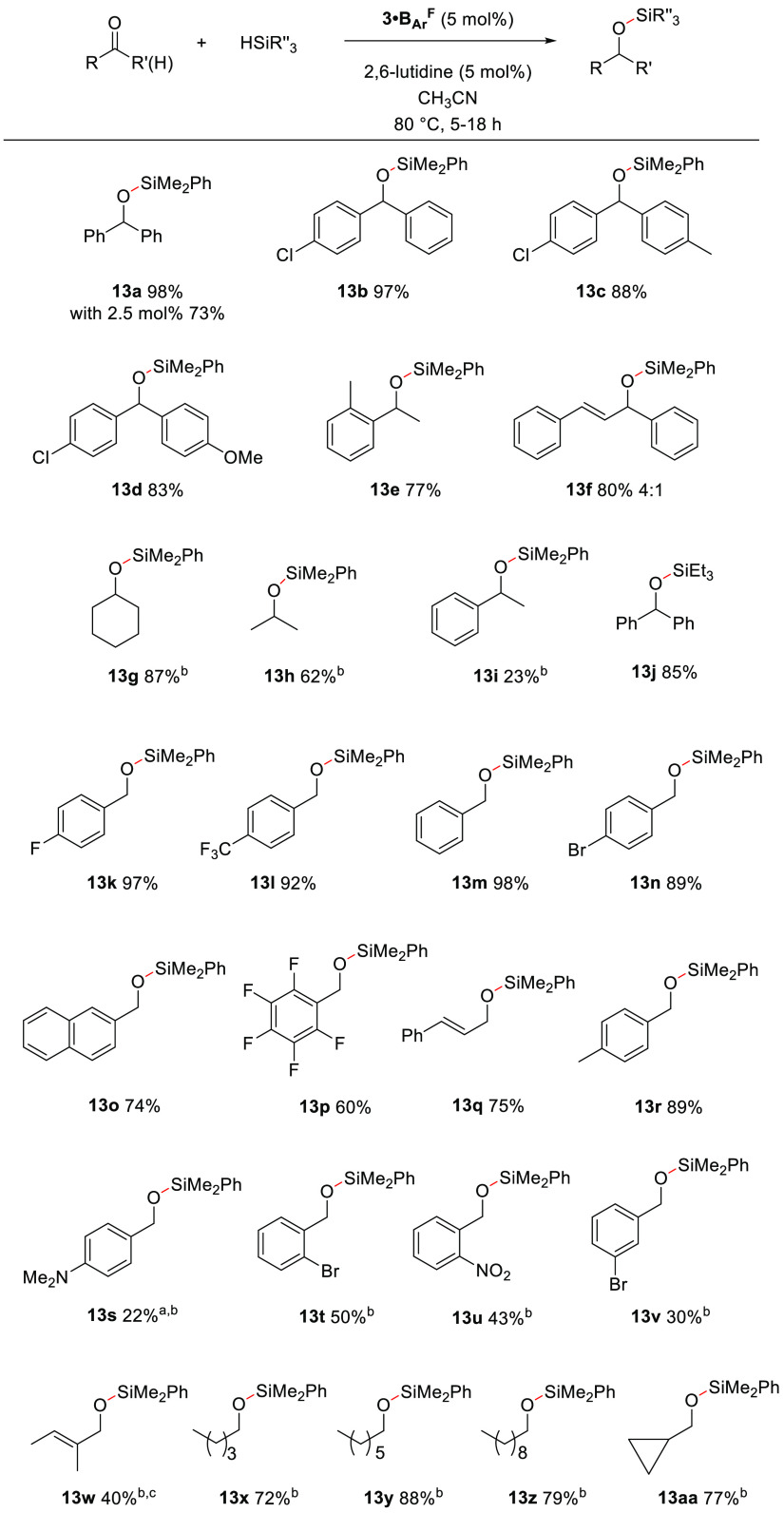
Reaction conditions: (i) activation step Ph_2_P(O)H **2** (5 mol %), Tf_2_O (5 mol %), NaB_Ar_^F^ (5 mol %), diphenylacetylene **1** (5 mol %), CD_3_CN (0.41 M), 60 °C, and 30 min and (ii) 2,6-lutidine (5 mol %), ketone or aldehyde (0.25 mmol), silane (0.25 mmol), 80 °C, and 5 h, reported as an average of two runs; ^a^conversion; ^b^10 mol %, 18 h; ^c^spectroscopic yield.

With the optimal conditions for catalytic hydrosilylation and choosing to use commercially available diphenyl acetylene for in situ pre-catalyst formation (Table 2, entry 6), we explored the generality of the reaction. Compound **13a** can be isolated in 73% yield, after 5 h at 60 °C using 2.5 mol % **3·B**_**Ar**_^**F**^ (Figure 6). Aryl-substituted ketones react well under the reaction conditions, giving **13b**-**13d** in excellent 83–97% isolated yields. Electron-withdrawing and electron-donating groups in the para-position are well tolerated. These results are interesting because diaryl ketones have been reported to typically react to yield fully deoxygenated products when subjected to higher temperatures or longer reaction times.^40^

In this procedure, the higher temperature does not affect the reaction outcome with hydrosilylation of diaryl ketones occurring selectively in 5 h. Unsymmetrical ketones with an aryl substituent and C_sp^3^_ and C_sp^2^_ substituents yield **13e** and **13f** in full conversion (77 and 80% isolated yields, respectively). Alkyne-containing carbonyl substrates are not suitable for this transformation, possibly due to alkyne scrambling of the propargyl group.^41^ Cyclohexanone and acetone react well using 10 mol % **3·B**_**Ar**_^**F**^, giving **13g** and **13h** in 87 and 62% yields, respectively, while acetophenone reacts sluggishly, and even with 10 mol % catalyst loading, only 23% of **13i** is obtained. The use of other tertiary silanes was found to be suitable, and **13j** is obtained in full conversion and high yield (85%). However, triethoxy silane and triphenyl silane do not convert, but the phosphirenium pre-catalyst is unaffected and is still observed in the reaction mixture. Secondary or primary silanes do not react with benzophenone **11** under the reaction conditions, and decomposition of the phosphirenium is observed by NMR spectroscopy.

Several aldehydes were subjected to the optimized reaction conditions, and the results are good to excellent when using electron-poor aryl aldehydes, with 60–98% isolated yields for **13k–13p**. Cinnamaldehyde reacts smoothly to yield **13q** in 75% yield. The reaction also works well with 4-methylbenzaldehyde, furnishing **13r** in 89% isolated yield, but is quite limited with other electron-rich aldehydes. 4-Dimethylamino benzaldehyde shows low conversion into **13s** (22%) after 18h with 10 mol % **3·B**_**Ar**_^**F**^; 4-methoxybenzaldehyde does not show any conversion after 24 h, which suggests a potential deactivation of the carbonyl or catalyst poisoning. Electron-withdrawing groups in the *ortho-* and *meta*-position are detrimental, giving low conversion and yield of **13t**, **13u**, and **13v**. Tiglic aldehyde reacts using 10 mol % catalyst at 80 °C with full conversion of silane to **13w** in only 40% spectroscopic yield, with traces of the product derived from the reduction of the double bond observed in situ. Alkyl aldehydes such as pentanal, heptanal, and dodecanal give **13x**, **13y**, and **13z** in 72–88% isolated yields, respectively, and cyclopropane aldehyde reacts to give **13aa** in 77% isolated yield with no ring opening observed, highlighting that radicals are not involved in the reaction.

## 3 Conclusions

The ease of synthesis and the newly discovered reactivity of phosphirenium ions allow us to conclude that they are effective organocatalysts for the hydrosilylation of ketones and aldehydes; to the best of our knowledge, this is the first report of a phosphirenium ion being used as a pre-catalyst. Full characterization of the phosphirenium ions bearing triflate or borate counterions is reported, and further details on their synthesis were explored. The practical pre-catalyst synthesis from typically cheap and readily available starting materials such as phosphine oxides and alkynes allows a straightforward catalytic procedure that is easily scalable. Our mechanistic investigations give insights into the principles governing their reactivity; this is underpinned by DFT studies and experimentally determined kinetics and stoichiometric studies. The ability of these phosphirenium ions to undergo ring opening paves the way for several reaction pathways, with the dominant and most favorable pathway being the release of the active catalyst, which is a phosphenium ion adduct. Increasing the electron-donating ability of the alkyne used to prepare the pre-catalyst slows catalysis, allowing an off-cycle deactivation process to dominate. These elusive phosphenium species are highly reactive and challenging to isolate and characterize; therefore, by disguising a phosphenium catalyst as a more stable and manageable phosphirenium adduct, we open up a multitude of opportunities in organic synthesis. Work is still ongoing in our laboratory to shed light on diverse reactivity and the activation modes of phosphirenium rings and the broader applicability of masked phosphenium catalysts.

## 4 Experimental Section

### 4.1 General Procedure for Synthesis of Phosphirenium Ions

In a J-Young NMR tube or a Schlenk tube, alkyne (0.25 mmol), SPO (0.25 mmol), triflic anhydride (1 equiv.), and NaB_Ar_^F^ (1 equiv.) were stirred in CD_3_CN or CDCl_3_ at 60 °C for 30 min. The reaction was monitored by ^31^P{^1^H} NMR spectroscopy until complete disappearance of the phosphine oxide peak. After the reaction was completed, the mixture was concentrated under vacuum. The product was washed with pentane and recrystallized at −20 °C.

### 4.2 General Procedure for the Hydrosilylation of Ketones and Aldehydes

To a J-Young NMR tube, dicyclohexyl phosphine oxide (5 mol %, 12.5 × 10^–3^ mmol, 2.7 mg) or diphenyl phosphine oxide (5 mol %, 12.5 × 10^–3^ or 25 × 10^–3^ mmol, 2.5 mg), diphenylacetylene (5 mol %, 12.5 × 10^–3^ mmol, 2.3 mg), triflic anhydride (5 mol %, 12.5 × 10^–3^ mmol, 2.1 μL), and NaB_Ar_^F^ (5 mol %, 12.5 × 10^–3^ mmol, 11.1 mg) were stirred in CH_3_CN (3 mL) for 30 min at 60 °C. After formation of pre-catalyst **3·B**_**Ar**_^**F**^, the reaction mixture was cooled down, and 2,6-lutidine (5 mol %, 12.5 × 10^–3^ mmol, 1.5 μL), benzophenone (0.25 mmol, 45.5 mg), and dimethylphenylsilane (0.25 mmol, 38.3 μL) were added, and the reaction was heated at 80 or 60 °C for 18 and 5 h, respectively. After drying the volatiles, the pure product was obtained by purification by flash chromatography (9:1 petroleum ether/EtOAc) or vacuum distillation.

### 4.3 DFT Calculations

Gaussian 09 (Revision D.01) was used with geometry optimizations carried out at the TPSS/def2-SVP level and all stationary points characterized with analytical frequency calculations. Electronic energies were recomputed at the M052X-D3(CH_3_CN)/def2-TZVP level, which has been shown to be a reliable approach for main group kinetics, thermodynamics, and non-covalent interactions.^42^ These corrected energies were then combined with the thermodynamic corrections from the gas-phase calculations to give the free energies quoted in the text. Full details and references are supplied in the Supporting Information.

## Supporting Information Available

Analysis data and NMR spectra for all products and computational supporting information which contains all data relevant to the computational studies reported here, including methodology, references, benchmarking studies, energetics, and coordinates of characterized stationary points (CIF)
General considerations, synthesis and characterization of the starting material, synthesis of phosphirenium ions, substrate scope hydrosilylation, and optimization of the catalytic reaction (PDF)
Computed geometries for all stationary points (XYZ)

## Supplementary Material

cs1c01133_si_001.cif

cs1c01133_si_002.pdf

cs1c01133_si_003.xyz
