# Supplementary material for: Phosphirenium Ions as Masked Phosphenium Catalysts: Mechanistic Evaluation and Application in Synthesis
Source: ACS Catal. 2021 Apr 20;11(9):5452–62. doi: 10.1021/acscatal.1c01133 (PMC8495902; doi:10.1021/acscatal.1c01133)
Supplement: Supplementary file 2 — cs1c01133_si_002.pdf [file cs1c01133_si_002.pdf]

SUPPORTING INFORMATION

Phosphirenium Ions as Masked Phosphenium Catalysts: Mechanistic Evaluation and Application in Synthesis

Danila Gasperini,[a] Samuel E. Neale,[b] Mary F. Mahon,[a] Stuart A. Macgregor,[b]* Ruth L. Webster[a]*

[a]Department of Chemistry, University of Bath, Bath, UK, BA2 7AY.

[b]Institute of Chemical Sciences, Heriot-Watt University, Edinburgh, UK, EH14 4AS.

Contents

1. General considerations ... 2
2. Synthesis and characterisation of starting material ... 3
3. Synthesis of phosphirenium ions: optimisation table ... 5
4. General procedure for the synthesis of phosphirenium ions ... 7
5. Optimisation catalytic reaction ... 10
6. Substrate scope hydrosilylation ... 12
   6.1 Deoxygenation reaction ... 16
7. Mechanistic investigation ... 17
   7.1 Understanding the pre-catalyst activation step ... 17
   7.2 Stoichiometric studies ... 24
   7.3 Phosphirenium cation in catalysis ... 34
   7.4 Reaction kinetics ... 41
8. NMR, Mass and IR Spectra ... 57
9. Crystallographic details ... 91
10. Computational Details ... 101
   10.1 Free energy profiles ... 102
   10.2 Computed Cartesian coordinates (Å) and energies (au) for all species ... 104
11. References ... 131

1. General considerations

All solvents and reagents were used as purchased and all reactions were performed under an inert atmosphere using standard Schlenk and glovebox techniques, unless otherwise stated. Heating and anhydrous reactions were undertaken in Teflon-sealed J-Young reaction NMR tubes and Schlenk vessels.

Laboratory grade protiated acetonitrile was purchased from Fisher Scientific and dried over sodium/benzophenone and distilled prior use. Reagents such as terminal alkynes, diphenylacetylene and triflic anhydride were purchased from Merck or Acros and used as purchased in an argon filled glovebox. Silanes were dried over $MgSO_4$ and kept in an argon filled glovebox. $NaB_{Ar}{}^F$ was synthesized according to literature procedures.[1] Aldehyde were washed with NaOH, extracted and dried to get rid of traces of carboxylic acids. Home-made phosphines and alkynes were synthesised and stored dry in air. Room temperature (r.t.) refers to 298 K. Temperatures of 0 °C (273 K) and –78 °C (195 K) were obtained using ice/water and CO_2(s)/acetone baths respectively. -18 °C (255 K) refers to the average freezer temperature.

^1H, $^{13}C\{^1H\}$, $^{19}F\{^1H\}$, $^{29}Si\{^1H\}$ $^{31}P\{^1H\}$ NMR spectra were recorded on Bruker Avance or Agilent 500, 400 and 300 MHz NMR spectrometers. In CD_3CN, ^1H and $^{13}C\{^1H\}$ NMR chemical shifts are reported relative to CH_3CN at 1.94 ppm and 118.26 ppm, respectively; in $CDCl_3$, ^1H and $^{13}C\{1H\}$ NMR chemical shifts are reported relative to $CHCl_3$ at 7.26 ppm and 77.16 ppm, respectively; in CD_2Cl_2, ^1H and $^{13}C\{^1H\}$ NMR chemical shifts are reported relative to $CHCl_3$ at 5.32 ppm and 54.0 ppm, respectively For the assignment of the ^1H and $^{13}C\{1H\}$ NMR spectra 2D NMR (COSY, HSQC, HMBC) experiments were also performed. Coupling constants (J) are reported in Hertz (Hz). Multiplicities are indicated by: br s (broad singlet), s (singlet), d (doublet), t (triplet), q (quartet) and m (multiplet). HRMS analyses were performed using an Agilent QTOF 6545 with Jetstream ESI spray source coupled to an Agilent 1260 Infinity II Quat pump HPLC with 1260 autosampler, column oven compartment and variable wavelength detector (VWD). Single crystals were analysed on a Supernova and New Xcalibur, EosS2 diffractometer. HPLC analysis were performed using an Agilent Technologies 1260 Infinity.

2. Synthesis and characterisation of starting material

Substituted symmetrical alkynes were prepared following the described procedure.[2] A 100 mL Schlenk equipped with a magnetic stir bar was purged with dry argon and then charged with $PdCl_2(PPh_3)_2$ (0.3 mmol), CuI (1 mmol) and starting aryl iodide or bromide (10 mmol). Dry toluene (50 mL) was then added by syringe under a dry argon flow. Argon-sparged DBU (60 mmol) was then added by syringe followed by a purge of the reaction Schlenk with argon. (Trimethylsilyl)acetylene (5 mmol) was then added by syringe followed immediately by distilled water (4 mmol). The reaction Schlenk was covered with aluminium foil and left stirring at RT (X = I) or 60 °C (X = Br) for 18 h. The reaction mixture was then partitioned in diethyl ether and distilled water (50 mL each). The organic layer was washed with 10% HCl (3 × 75 mL), saturated aqueous NaCl (1 × 75 mL), dried over $MgSO_4$, gravity-filtered and the solvent removed in vacuo. The crude product was purified by silica gel column chromatography (Petroleum ether/EtOAc).

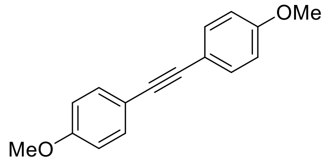

1,2-bis(4-methoxyphenyl)ethyne was synthesised using 4-bromotoluidine (5 mmol) and following the general procedure and obtained as a white solid (4.85 mmol, 1.15 g, 97% isolated yield) whose data are comparable to these found in the literature;[2] ^{1}H NMR (400 MHz, $CDCl_3$) δ (ppm) 7.45 (d, *J* = 8.8, 4H), 6.87 (d, *J* = 8.8, 4H), 3.82 (s, 6H); $^{13}C\{^1H\}$ NMR (126 MHz, $CDCl_3$) δ (ppm) 158.3, 131.8, 114.6, 112.9, 86.9, 54.2; m.p. (°C) = 141-144.

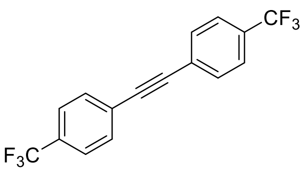

1,2-bis(4-(trifluoromethyl)phenyl)ethyne was synthesised using 4-1-iodo,4-trifluomethanebenzene (10 mmol) and following the general procedure and obtained as an orange solid (4.85 mmol, 1.15 g, 97% isolated yield) whose data are comparable to these found in the literature;[2] ^{1}H NMR (300 MHz,$CDCl_3$) δ (ppm) 7.66 (d, *J* = 9.5, 4H), 7.63 (d, *J* = 9.3, 4H); $^{13}C\{^1H\}$ NMR (126 MHz, $CDCl_3$) δ (ppm) 133.1, 130.9, 126.5, 124.4, 98.3, 90.1; $^{19}F\{^1H\}$ NMR (470 MHz, $CDCl_3$) δ (ppm) −62.9; m.p. (°C) = 101-104.

Asymmetric ketones were prepared following the described literature procedure.[3] *n*-Butyllithium (1.67 M solution in hexane, 1.32 mL, 2.2 mmol) was added dropwise to a solution of 1-bromo-4-methylbenzene (342 mg 2.0 mmol) in THF (3 mL) at −78 °C for 30 min. Then, substituted benzaldehyde (261 mg, 2.1 mmol) was added to the mixture at −78 °C and the obtained mixture was stirred at r.t. for 1 h. Then, after removal of the solvent, I_2 (812 mg, 3.2 mmol), K_2CO_3 (829 mg, 6.0 mmol), and *tert*-BuOH (3 mL) were added and the obtained mixture was stirred for 3 h at refluxing conditions. The reaction mixture was quenched with sat. aq. Na_2SO_3 (5 mL) and was extracted with $CHCl_3$ (3 × 20 mL). The organic layer was washed with brine and dried over $MgSO_4$ to provide products. The products were purified by a silica gel column chromatography (petroleum ether/EtOAc).

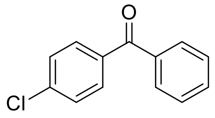

(4-chlorophenyl)(phenyl)methanone was synthesized according to the literature procedure and obtained as a white solid (411.6 mg, 1.9 mmol, 98% isolated yield) whose data are comparable to these found in the literature;[3] ^{1}H NMR (300 MHz,$CDCl_3$) δ (ppm) 7.42-7.51 (m, 4H), 7.59 (t, *J* = 7.4, 1H), 7.72-7.78 (m, 4H); $^{13}C\{^1H\}$ NMR (126 MHz, $CDCl_3$) δ (ppm) 195.4, 138.8, 137.2, 135.8, 132.6, 131.4, 129.9, 128.6, 128.3; m.p. (°C) = 74-76.

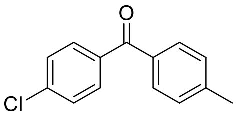

(4-chlorophenyl)(*p*-tolyl)methanone was synthesized according to the literature procedure and obtained as a white solid (1.7 mmol, 391.3 mg, 85% isolated yield) whose data are comparable to these found in the literature;[3] ^{1}H NMR (300 MHz,$CDCl_3$) δ (ppm) 7.75 (d, *J* = 8.8, 2H), 7.69 (d, *J* = 8.2, 2H), 7.45 (d, *J* = 8.1, 2H), 7.28 (d, *J* = 7.7, 2H), 2.44 (s, 3H); $^{13}C\{^1H\}$ NMR (126 MHz, $CDCl_3$) δ (ppm) 195.2, 143.5, 138.6, 136.2, 134.5, 131.3, 130.1, 129.1, 128.5, 21.6; m.p. 129-131 °C

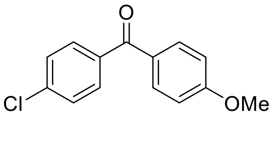

(4-chlorophenyl)(4-methoxyphenyl)methanone was synthesized according to the literature procedure and obtained as a white solid (458.8 mg, 1.85 mmol, 93% isolated yield) whose data are comparable to these found in the literature;[3] ^{1}H NMR (500 MHz, $CDCl_3$) δ (ppm) 7.80 (d, *J* = 8.9, 2H), 7.71 (d, *J* = 8.6, 2H), 7.45 (d, *J* = 8.6,

2H), 6.97 (d, *J* = 8.9, 2H), 3.89 (s, 3H); $^{13}C\{^1H\}$ NMR (126 MHz, $CDCl_3$) δ (ppm) 194.2, 163.4, 138.3, 136.5, 132.4, 131.1, 129.8, 128.5, 113.7, 55.5; m.p. (°C) = 128-132.

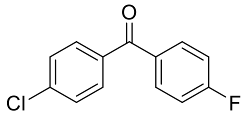

(4-chlorophenyl)(4-fluorophenyl)methanone was synthesized according to the literature procedure and obtained as a white solid (335.5 mg, 1.4 mmol, 70% isolated yield) whose data are comparable to these found in the literature;[3] ^1H NMR (500 MHz, $CDCl_3$) δ (ppm) 7.78-7.84 (m, 2H), 7.72 (d, *J* = 8.4, 2H), 7.46 (d, *J* = 8.4, 2H), 7.17 (t, *J* = 8.6, 2H); $^{13}C\{^1H\}$ NMR (100 MHz, $CDCl_3$) δ (ppm) 193.9, 165.4 (d, $J_{C\text{-}F}$ = 253.2), 138.9, 135.7, 133.4 (d, $J_{C\text{-}F}$ = 2.9), 132.5 (d, $J_{C\text{-}F}$ = 8.6), 131.2, 128.6, 115.6 (d, $J_{C\text{-}F}$ = 22.1); m.p. (°C) 104-107.

Asymmetric secondary phosphine oxides were synthesised following the literature procedure.[4] Alkyl magnesium bromide/chloride (1M in OEt_2, 22 mL) was cooled to 0 °C under N_2, then a solution of *P*,*P*-dichlorophenylphospine (20 mmol) in dry OEt_2 (5 mL) was dropwise added over 30 min. The mixture was then stirred at r.t overnight then quenched with sat. aq. NH_4Cl solution. Water (70 mL) was then added and the aqueous phase was then extracted with $CHCl_3$ (3 × 100 mL). The combined organic phases were dried over anhydrous $MgSO_4$, concentrated under vacuum, and the crude residue purified by silica gel column chromatography (Petroleum ether/EtOAc) to afford the desired secondary phosphine oxide (SPO).

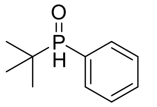

tert-butyl(phenyl)phosphine oxide was synthesized according to the literature procedure and obtained as a white solid (14.4 mmol, 2.6 g, 80% isolated yield) whose data are comparable to these found in the literature;[4] ^1H NMR (300 MHz,$CDCl_3$) δ (ppm) 7.73 (s, 1H), 7.58-7.65 (m, 2H), 7.41-7.52 (m, 3H), 1.06 (d, *J* = 16.6, 9H); $^{13}C\{^1H\}$ NMR (300 MHz, $CDCl_3$) δ (ppm) 132.1 (d, *J* = 2.8), 130.2 (d, *J* = 9.9), 129.3 (d, *J* = 90.0), 129.1 (d, *J* = 11.8), 32.4 (d, *J* = 69.2), 23.0 (d, *J* = 2.1); $^{31}P\{^1H\}$ NMR (300 MHz, $CDCl_3$) δ (ppm) 47.71 (ddq, *J* = 453.2, 30.3, 15.4, 14.9).

Further resolution of *tert*-butyl(phenyl)phosphine oxide was obtained following procedure reported in the literature.[5] Resolution by diastereomeric complex formation with (*S*)-mandelic acid (1 equiv.) and racemic *tert*-butyl(phenyl)phosphine oxide (954 mg, 5.2 mmol) were dissolved in OEt_2 and stirred at RT for 72 h. The mixture was heated to reflux for 2 h then left crystallising at −18°C. The diastereomeric mixture was separated by fractional crystallisation to yield the (−)-SPO-(+)-MA complex as colourless crystals [^1H NMR (500 MHz, $CDCl_3$) δ (ppm) 7.65 (dd, *J* = 12.2, 7.5, 2H), 7.59 (t, *J* = 7.5 Hz, 1H), 7.49 (dt, *J* = 8.3, 4.2, 2H), 7.45 – 7.42 (m, 2H), 7.37 – 7.29 (m, 3H), 5.18 (s, 1H), 1.13 (d, *J* = 16.8, 10H), $[\alpha]_D^{25}$ +7.3°(c = 0.5, $CHCl_3$); m.p. (°C) 138-141]. These were filtered off and dissolved in 1 M NaOH (10mL) and $CHCl_3$ (10mL). The layers were separated, and the aq. phase was extracted with $CHCl_3$ (5 × 5mL). The combined organic layers were dried, and the solvent removed under reduced pressure to give (*S*)-*tert*-butyl(phenyl)phosphine oxide (*S*)-*tert*-butyl(phenyl)phosphine oxide as a colourless solid (426.3 mg, 2.34 mmol, 46% yield) whose NMR data were as above; $[\alpha]_D^{25}$ −3.8°(c = 0.1, MeOH), m.p. (°C) 51-53.

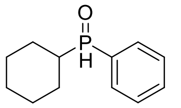

cyclohexyl(phenyl)phosphine oxide was synthesized according to the literature procedure and obtained as a white solid (14.4 mmol, 2.6 g, 80% isolated yield) whose data are comparable to these found in the literature;[6] ^1H NMR (400 MHz, $CDCl_3$) δ (ppm) 7.72 –7.62 (m, 2H), 7.59 – 7.54 (m, 1H), 7.53 –7.47 (m, 2H), 7.19 (dd, *J* = 456.4, 2.3, 1H), 1.97 –1.78 (m, 5H), 1.74 –1.65 (m, 1H), 1.40 –1.15 (m, 5H); $^{13}C\{^1H\}$ NMR (126 MHz, $CDCl_3$) δ (ppm) 132.5 (d, *J* = 2.8), 130.4 (d, *J* = 10.4), 130.0 (d, *J* = 92.9), 128.9 (d, *J* = 12.1), 38.8 (d, *J* = 69.8), 26.2 (d, *J* = 4.3), 26.0 (d, *J* = 3.8), 25.9 (d, *J* = 1.6), 25.4 (d, *J* = 1.7), 24.7 (d, *J* = 2.6); $^{31}P\{^1H\}$ NMR (162 MHz, $CDCl_3$) δ (ppm) 37.3.

3. Synthesis of phosphirenium ions: optimisation table

Table S1. Optimisation of the synthesis of phosphirenium salts

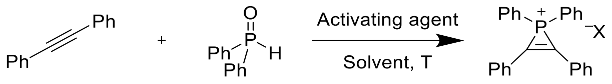

Entry	Activating agent	Solvent	T (°C)	Time	Conv (%)	X
1	HOTf	CD_2Cl_2	RT to 60	10 min to 18 h	/	/
2	AgOTf	CD_2Cl_2	RT to 60	10 min to 18 h	/	/
3	$NaB_{Ar}{}^F$	CD_2Cl_2	RT to 60	10 min to 18 h	/	/
4	$Fe(OTf)_2$	CD_2Cl_2	RT to 60	10 min to 18 h	/	/
6	$(OTf)_2$	$CDCl_3$	80	30 min	95[c]	OTf
7	$(OTf)_2$	C_6D_6	80	30 min	56[c]	OTf
8	$(OTf)_2$	CD_3CN	80	30 min	98[c]	OTf
9	$(OTf)_2/NaB_{Ar}{}^F$	$CDCl_3$	80	30 min	/	$B_{Ar}{}^F$
10	$(OTf)_2/NaB_{Ar}{}^F$	CD_3CN	80	30 min	>99	$B_{Ar}{}^F$
11	$(OTf)_2/NaBF_4$	$CDCl_3$	80	10 min to 18 h	>99	OTf
12	$(OTf)_2/AgNTf_2$	$CDCl_3$	80	30 min	>99	OTf
13	$(OTf)_2/AgSbF_6$	$CDCl_3$	RT to 60	10 min to 18 h	/[b]	/
14	$(OTf)_2/AgBF_4$	$CDCl_3$	RT to 60	10 min to 18 h	/[b]	/
15	$(OAc)_2$	$CDCl_3$	RT to 60	10 min to 18 h	Mixture of products[a]	/
16	$O(CO_2tBu)_2$	$CDCl_3$	RT to 60	10 min to 18 h	/	/
17	$HOTf/PhSiH_3$	CD_3CN	80	18 h	$PHPh_2$	/
18	$(OTf)_2$ (0.5 equiv.)	CD_3CN	80	18 h	32[c]	OTf
19	$(OTf)_2$ (2 equiv.)	CD_3CN	80	18 h	>99	OTf
20	$(OTf)_2$	CD_3CN	rt	30 min	47[c]	OTf

Reaction conditions; diphenylacetylene (0.25 mmol), diphenylphosphine oxide (0.25 mmol), activator (0.25 mmol unless otherwise stated), solvent (0.55 M), RT to 60 °C or 80 °C; [a] see description below; [b] potential formation of silver adducts.[7] [c] spec yield calculated using a capillary of PPh_3 in CD_3CN (0.1 M).

Chlorinated solvents were found to be suitable for the reaction, *e.g.* CH_2Cl_2 and $CDCl_3$, however with solubility issues of the salt at 0.55 M. Similarly, the reaction proceeds in C_6D_6, with solubility issues at 0.55 M. The salts are fully soluble in CD_3CN, which allows quantification and analysis of products thus solvent of choice.

2.1 Details of reaction of diphenylphosphine oxide and acetic anhydride

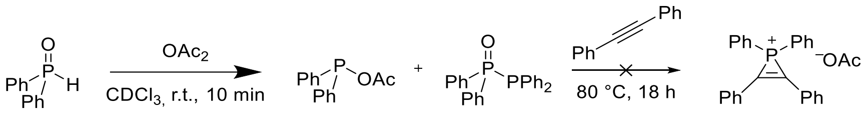

In a J-Young NMR tube or a Schlenk tube, diphenyl phosphine oxide (0.25 mmol) and acetic anhydride (1 equiv.), were stirred in $CDCl_3$ at RT. The reaction was monitored by $^{31}P\{^1H\}$ NMR. After 10 min the following signals were obtained; 81.91 ppm potentially Ph_2POAc,[8] 37.2 (d, J = 231.6 Hz) and -22.0 (d, J = 231.0 Hz) as $Ph_2P(O)PPh_2$,[9] and other peaks (singlets at 33.4, 29.4, 28.7, 22.3) were not identified. When adding diphenyl acetylene (0.25 mmol) and heating the reaction at 80 °C for 18 h, cycloaddition to form phosphirenium ion was not observed.

Figure S1 ^{1}H NMR(top) and $^{31}P\{1H\}$ NMR (bottom) for the reaction of diphenyl phosphine oxide and acetic anhydride

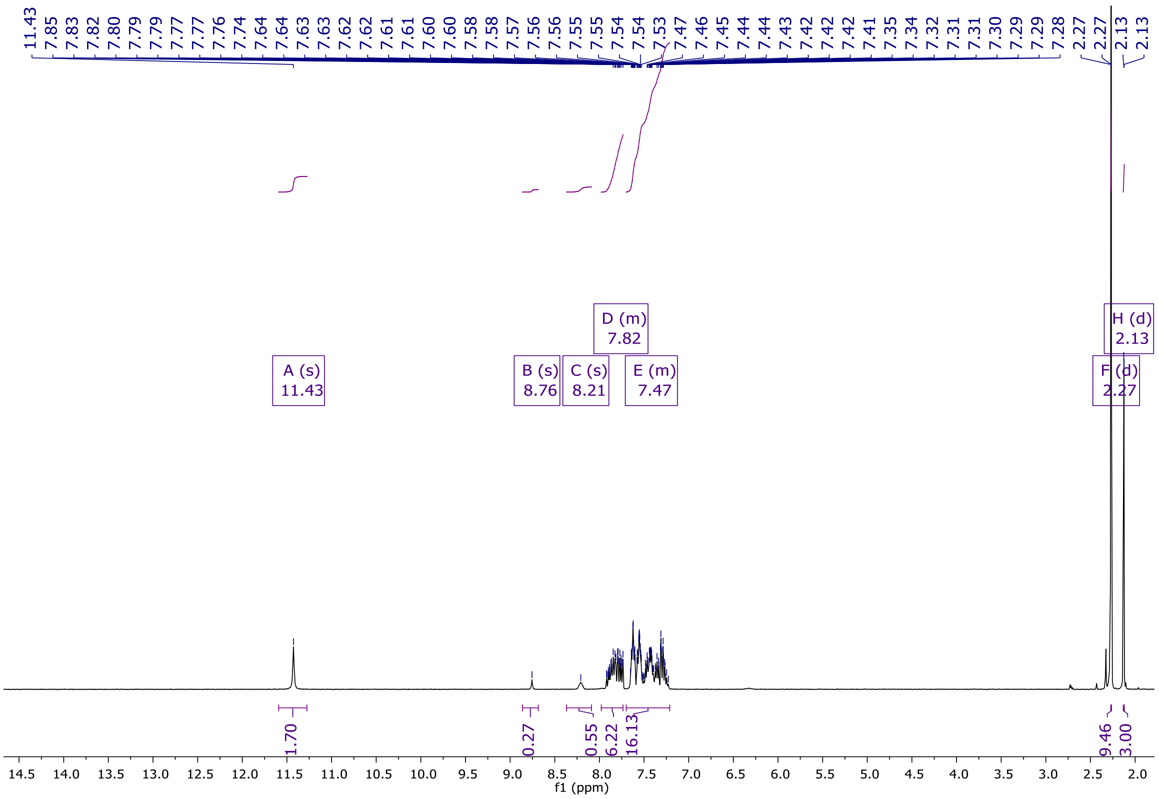

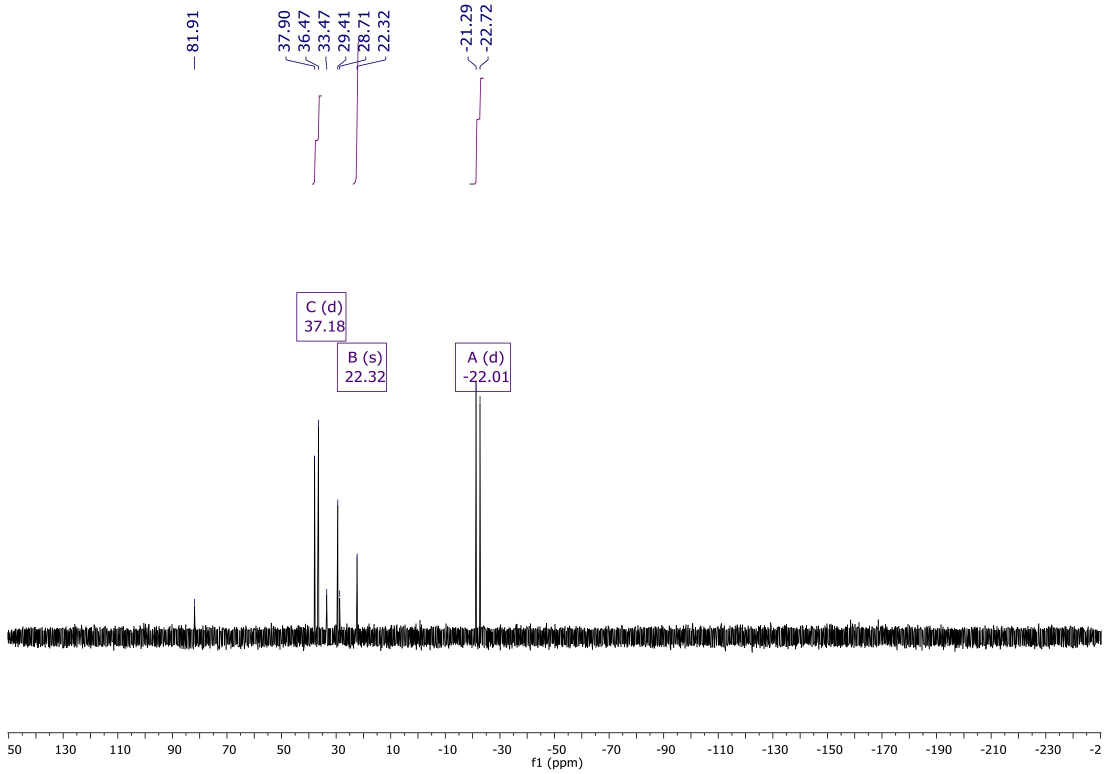

4. General procedure for the synthesis of phosphirenium ions

General procedure A: In a J-Young NMR tube or a Schlenk tube, alkyne (0.1-0.2 mmol), secondary phosphine oxide (0.1-0.2 mmol) and triflic anhydride (1 equiv.), were stirred in dichloromethane or acetonitrile at 60 °C for 1 h. The reaction was monitored by $^{31}P\{^1H\}$ NMR until complete disappearance of phosphine oxide peak. After the reaction was completed the mixture was concentrated under vacuum. The product was washed with pentane and recrystallized at -18 °C or r.t. in mixture of acetonitrile/pentane or dichloromethane/pentane. The products were kept in an inert atmosphere where if dry did not decompose even after 3 months of storage. The ions are sensitive to adventitious traces of water, but do not require extremely dry conditions as phosphines or alkynes kept dry in air have been used. If left in solution of CD_3CN decomposition was observed by $^{31}P\{^1H\}$ NMR after a week.

General procedure B: In a J-Young NMR tube or a Schlenk tube, alkyne (0.1-0.2 mmol), secondary phosphine oxide (0.1-0.2 mmol), triflic anhydride (1 equiv.) and NaB_{Ar}^F (1 equiv.), were stirred in dichloromethane or acetonitrile at 60 °C for 1 h. The reaction was monitored by $^{31}P\{^1H\}$ NMR until complete disappearance of phosphine oxide peak. After the reaction was completed the mixture was concentrated under vacuum. The product was washed with pentane and recrystallized at -20 °C or r.t. in mixture of acetonitrile/pentane or dichloromethane/pentane. The products were kept in an inert atmosphere where if dry did not decompose even after 3 months of storage. The ions are sensitive to adventitious traces of water, but do not require extremely inert conditions and phosphines or alkynes kept dry in air have been used. If left in solution of CD_3CN decomposition was observed by $^{31}P\{^1H\}$ NMR after a week.

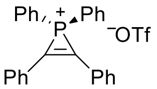

3•OTf

1,1,2,3-tetraphenyl-1H-phosphiren-1-ium trifluoromethanesulfonate **3·OTf** was synthesised through general procedure A with diphenylacetylene (0.2 mmol, 35.6 mg), diphexylphosphine oxide (0.2 mmol, 40.4 mg), triflic anhydride (0.2 mmol, 33.8 µL) in CD_3CN. The reaction was stirred at 60 °C for 1 h. The mixture was concentrated under vacuum and the solid obtained washed with pentane to yield **3·OTf** as a light-brown solid (89.2 mg, 0.17 mmol, 87%); ^1H NMR (300 MHz, $CDCl_3$) δ 8.41 – 7.01 (m, 20H); $^{13}C\{^1H\}$ NMR (126 MHz, CD_3CN) δ 136.7, 135.3 (d, *J* = 13.6), 134.5, 132.8 – 132.5 (m), 131.3 (d, *J* = 16.3), 130.9, 130.5 (d, *J* = 14.8), 129.6, 123.9 (d, *J* = 2.3), 117.0 (d, *J* = 93.4); $^{31}P\{^1H\}$ NMR (122 MHz, $CDCl_3$) δ −108.2; $^{19}F\{^1H\}$ NMR (470 MHz, $CDCl_3$) δ -78.4; HRMS (ESI): calcd for $C_{27}H_{20}F_3O_3PS^+$ $[M+H]^+$: 512.0823, found: 512.0830.

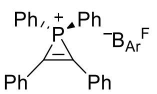

$3{\bullet}B_{Ar}{}^F$

1,1,2,3-tetraphenyl-1H-phosphiren-1-ium tetrakis[3,5-bis(trifluoromethyl)phenyl]borate **$3{\cdot}B_{Ar}{}^F$** was synthesised through general procedure B with diphenylacetylene (0.1 mmol, 17.8 mg), diphenylphosphine oxide (0.1 mmol, 20.2 mg) triflic anhydride (0.1 mmol, 16.9 µL) and Na $B_{Ar}{}^F$ (0.1 mmol, 88.6 mg) in CD_3CN. The reaction was stirred at 60 °C for 1 h. The mixture was concentrated under vacuum and the solid obtained washed with hexane to yield **$3{\cdot}B_{Ar}{}^F$** as a light-brown solid analysed *in situ* (75% spectroscopic yield); minor traces of NaOTf are visible by ^{19}F NMR, which was confirmed by spiking the solution with 10 mg of NaOTf. Suitable crystals were grown in CH_2Cl_2/pentane at −18 °C; ^1H NMR (500 MHz, CD_3CN) δ 8.11 – 7.76 (m, 3H), 7.75 – 7.70 (m, 12H), 7.67 (s, 6H), 7.67 – 7.48 (m, 3H), 7.49 – 7.30 (m, 6H), 7.25 (t, *J* = 8.1, 2H); $^{13}C\{^1H\}$ NMR (126 MHz, CD_3CN) δ 163.1 (d, *J* = 49.7), 162.3 (d, *J* = 49.9), 136.6 (d, *J* = 24.4), 135.8 – 135.7 (m), 133.7 (d, *J* = 13.1), 133.3 (d, *J* = 2.5), 132.7 (d, *J* = 11.9), 131.2 – 130.9 (m), 130.9 – 130.7 (m) 130.7 – 130.6 (m), 130.1 (dd, *J* = 5.8, 2.8), 129.9 (dd, *J* = 5.7, 2.9), 129.7 – 129.6 (m), 125.4 (q, *J* = 271.8, CF_3) 118.6 (dt, *J* = 7.8, 3.9); $^{31}P\{^1H\}$ NMR (122 MHz, CD_3CN) δ −109.1; $^{11}B\{^1H\}$ NMR (96 MHz, CD_3CN) δ −3.6; ^{19}F NMR (470 MHz, CD_3CN) δ -63.2; HRMS (ESI): calcd for $C_{26}H_{20}P^+$ $[M]^+$: 363.1302, found: 363.1303.

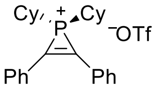

4•OTf

1,1-dicyclohexyl-2,3-diphenyl-1H-phosphiren-1-ium trifluoromethanesulfonate **4·OTf** was synthesised through general procedure B with diphenylacetylene (0.15 mmol, 31.9 mg), dicyclohexylphosphine oxide (0.15 mmol, 29.3 mg), triflic anhydride (0.15 mmol, 27.6 µL) in CD_2Cl_2. The reaction was stirred at 60 °C for 1 h. The mixture was concentrated under vacuum and the solid obtained washed with pentane to yield **4·OTf** as a light yellow solid (97%); ^1H NMR (300 MHz, CD_3CN) δ 8.09 – 7.97 (m, 5H), 7.73 – 7.64 (m, 7H), 3.21 – 3.05 (m, 2H), 2.14 – 1.99 (m, 4H), 1.74 (ddq, *J* = 9.6, 6.6, 3.0 Hz, 4H), 1.68 – 1.58 (m, 1H), 1.51 – 1.04 (m, 13H); $^{13}C\{^1H\}$ NMR (75 MHz, CD_3CN) δ 133.9, 132.4 (d, *J* = 7.5), 132.25, 130.7, 129.5, 125.1 (d, *J* = 3.8), 123.7 (d, *J* = 3.9), 33.9 (d, *J* = 30.1), 29.00 (d, *J* = 4.4), 26.42 (d, *J* = 16.2), 25.37 (d,

J = 2.1); $^{31}P\{^1H\}$ NMR (202 MHz, CD_2Cl_2) δ −85.4; $^{19}F\{^1H\}$ NMR (471 MHz, CD_2Cl_2) δ −78.5; HRMS (ESI): calcd for $C_{26}H_{32}F_3O_3PS^+$ [M]$^+$: 512.1766, found: 512.1762.

1,1-dicyclohexyl-2,3-diphenyl-1H-phosphiren-1-ium tetrakis[3,5-bis(trifluoromethyl)phenyl]borate **4·B$_{Ar}$^{F}**

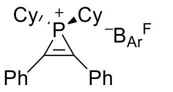

4•B$_{Ar}$^{F}

was synthesised through general procedure B with diphenylacetylene (0.15 mmol, 26.7 mg), dicyclohexylphosphine oxide (0.15 mmol, 31.2 mg), triflic anhydride (0.15 mmol, 25.2 µL) and NaB$_{Ar}$^{F} (0.15 mmol, 132.9 mg) in CD_3CN. The reaction was stirred at 60 °C for 1 h. The mixture was concentrated under vacuum and the solid obtained washed with pentane to yield **4·B$_{Ar}$^{F}** as a light yellow solid (120.7 mg, 0.09 mmol, 65%); ^1H NMR (500 MHz, CD_3CN) δ 8.06 – 7.98 (m, 4H), 7.88 – 7.84 (m, 3H), 7.76 (d, J = 1.7, 7H), 7.71 – 7.65 (m, 8H), 3.20 – 3.08 (m, 2H,C*H*), 2.16 – 2.04 (m, 5H, CH_2), 1.75 (ddt, J = 13.2, 6.7, 3.3, 5H, CH_2), 1.65 (dddt, J = 13.5, 5.2, 3.6, 1.8, 2H, CH_2), 1.48 – 1.28 (m, 11H, CH_2), 1.21 – 1.09 (m, 2H, CH_2). $^{13}C\{^1H\}$ NMR (126 MHz, CD_3CN) δ 135.4, 133.8, 132.3 (d, J = 7.5), 131.1, 130.8, 130.6, 129.9 – 128.0 (m, CF_3), 125.9, 125.0 (d, J = 3.8), 123.7 (d, J = 3.6), 121.2 – 121.0 (m), 33.9 (d, J = 30.0, *C*H Cy), 28.9 (d, J = 4.3, *C*H_2), 26.3 (d, J = 16.2, *C*H_2), 25.3 (d, J = 2.1, *C*H_2); $^{19}F\{^1H\}$ NMR (470 MHz, CD_3CN) δ −63.2; $^{31}P\{^1H\}$ NMR (202 MHz, CD_3CN) δ −86.5; m.p. (°C) 195-100; HRMS (ESI): calcd for $C_{26}H_{32}P^+$ [M]$^+$: 375.2242, found: 375.2215.

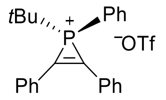

5•OTf

1-(*tert*-butyl)-1,2,3-triphenyl-1H-phosphiren-1-ium trifluoromethanesulfonate **5·OTf** was synthesised through general procedure A with diphenylacetylene (0.16 mmol, 29.3 mg), *tert*-butylphenyl phosphine oxide (0.16 mmol, 30.0 mg), triflic anhydride (0.1 mmol, 27.6 µL) in CD_3CN. The reaction was stirred at 60 °C for 1 h and the compound **5·OTf** characterised *in situ* (91% spectroscopic yield); ^1H NMR (500 MHz, CD_3CN) δ 8.16 – 7.93 (m, 5H), 7.86 (td, J = 7.9, 4.6, 3H), 7.76 (td, J = 5.0, 2.4, 1H), 7.62 – 7.53 (m, 5H), 7.50 – 7.38 (m, 11H), 7.37 – 7.24 (m, 4H), 2.86 – 2.78 (m, 1H), 2.55 (td, J = 7.1, 3.6, 1H), 1.61 (s, 1H), 1.54 (s, 6H), 1.49 (s, 6H); $^{13}C\{^1H\}$ NMR (126 MHz, CD_3CN) δ 138.1, 135.0 (d, J = 11.3), 134.5, 133.4 (d, J = 11.8), 132.7 (d, J = 8.2), 132.4, 131.6 (d, J = 14.0), 131.3, 131.1, 130.8, 130.5, 130.3, 130.1, 129.65, 129.6, 129.25, 123.9, 118.3, 90.1, 28.0 (d, J = 2.2) 24.2 (d, J = 5.4) 23.0 (d, J = 1.9); $^{31}P\{^1H\}$ NMR (202 MHz, CD_3CN) δ −93.3; $^{19}F\{^1H\}$ NMR (471 MHz, CD_3CN) δ −79.3.

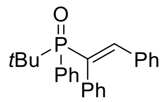

To further prove formation of the phosphirenium species, **5·OTf** was synthesised in situ through general procedure B with diphenylacetylene (0.27 mmol, 48.9 mg), *tert*-butylphenyl phosphine oxide (0.27 mmol, 49.19 mg), triflic anhydride (0.27 mmol, 46.1 µL) in CH_2Cl_2 (3 mL). After quenching with H_2O (3 mL), formation of *tert*-butyl(1,2-diphenylvinyl)(phenyl)phosphine oxide was observed and isolated (0.21 mmol, 76.6 mg, 79% yield); ^1H NMR (500 MHz, $CDCl_3$) δ (ppm) 7.71 (ddd, J = 9.6, 8.2, 1.4, 2H), 7.61 (d, J = 18.8, 1H), 7.53 – 7.47 (m, 1H), 7.40 (td, J = 7.7, 2.8, 2H), 7.34 – 7.25 (m, 3H), 7.16 – 7.05 (m, 5H), 6.94 – 6.90 (m, 2H), 1.15 (d, J = 14.9, 9H). $^{13}C\{^1H\}$ NMR (126 MHz, $CDCl_3$) δ (ppm) 144.1 (d, J = 8.3), 137.1 (d, J = 8.7), 133.3 (d, J = 7.5), 135.4 (d, J = 76.7), 131.5 (d, J = 2.7), 130.5 (d, J = 3.9), 130.3, 129.6 (d, J = 89.1), 128.8 (d, J = 1.3), 128.8, 128.7, 128.2, 127.9 (d, J = 1.9), 127.8 (d, J = 10.8), 35.1 (d, J = 69.8), 25.8. $^{31}P\{^1H\}$ NMR (202 MHz, $CDCl_3$) δ (ppm) 40.2. HRMS (ESI): calcd for $C_{24}H_{26}OP^+$ [M+H]$^+$: 361.1643, found: 361.1714.

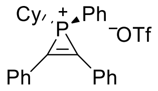

6•OTf

1-cyclohexyl-1,2,3-triphenyl-1H-phosphiren-1-ium trifluoromethanesulfonate **6·OTf** was synthesised through general procedure A with diphenylacetylene (0.1 mmol, 17.8 mg), phenyl,cyclohexyl phosphine oxide (0.1 mmol, 20.8 mg), triflic anhydride (0.1 mmol, 16.8 µL) in CD_3CN. The reaction was stirred at 60 °C for 1 h and **6·OTf** was characterised *in situ* (91% spectroscopic yield); ^1H NMR (500 MHz, CD_3CN) δ 8.10 – 8.03 (m, 4H), 7.96 – 7.85 (m, 2H), 7.77 – 7.61 (m, 10H), 3.76 (dddd, J = 11.8, 8.3, 6.9, 3.4, 1H), 2.84 – 2.76 (m, 1H), 2.28 – 2.15 (m, 3H), 1.88 – 1.75 (m, 3H), 1.75 – 1.66 (m, 2H), 1.62 – 1.39 (m, 6H), 1.37 – 1.11 (m, 2H). $^{13}C\{^1H\}$ NMR (126 MHz, CD_3CN) δ 136.2 (d, J = 3.8), 134.4, 134.3, 132.6 (d, J = 8.2), 132.4, 131.4, 131.3 (d, J = 14.8), 130.8, 130.7, 130.2, 129.6, 129.6, 129.5, 127.4, 124.6 (d, J = 8.3), 124.5 (d, J = 2.9), 123.9, 118.3, 90.0, 33.05 (d, J = 41.6), 29.1 (d, J = 4.1), 26.6 (d, J = 17.4), 25.6 (d, J = 2.2); $^{31}P\{^1H\}$ NMR (202 MHz, CD_3CN) δ −97.1; $^{19}F\{^1H\}$ NMR (470 MHz, CD_3CN) δ −79.3.

1,1-dicyclohexyl-2-phenyl-1H-phosphiren-1-ium trifluoromethanesulfonate **7·OTf** was synthesised through

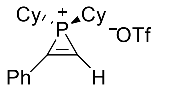

7•OTf

general procedure A with phenylacetylene (0.18 mmol, 20 µL), dicyclohexylphosphine oxide (0.15 mmol, 31.9 mg), triflic anhydride (0.15 mmol, 27.6 µL) in $CDCl_3$. The reaction was stirred at 60 °C for 1 h and the compound **7·OTf** characterised *in situ* (91% spectroscopic yield); ^1H NMR (300 MHz, CD_3CN) δ 8.29 (d, J = 17.0, 1H), 7.81 – 7.75 (m, 2H), 7.57 – 7.46 (m, 3H), 2.84

(tdt, J = 11.8, 5.9, 3.5, 2H, CHP), 1.83 (dtt, J = 9.7, 4.7, 3.0 Hz, 5H, CH_2), 1.68 – 1.49 (m, 5H, CH_2), 1.31 – 1.00 (m, 11H, CH_2). $^{13}C\{^1H\}$ NMR (75 MHz, CD_3CN) δ 136.6, 134.6, 132.4 (d, J = 7.1), 130.5, 124.2 (d, J = 4.0), 114.0 (d, J = 10.6 Hz, C=CH), 34.4, 33.2 (d, J = 32.8, CHP), 28.3 (dd, J = 48.9, 4.3), 26.36 (d, J = 3.2), 26.15 (d, J = 3.0), 25.9, 25.9, 25.4 (d, J = 2.2), 24.5 (d, J = 3.4). $^{31}P\{^1H\}$ NMR (122 MHz, CD_3CN) δ −81.71; $^{19}F\{^1H\}$ NMR (470 MHz, CD_3CN) δ −78.4; HRMS (ESI): calcd for $C_{21}H_{28}F_3O_3PS^+$ $[M]^+$: 448.1449, found: 448.1453.

1,1-dicyclohexyl-2-phenyl-1H-phosphiren-1-ium trifluoromethanesulfonate **8·OTf** was synthesised through general procedure A with phenylpropyne (0.25 mmol, 31.3 µL), dicyclohexylphosphine oxide (0.2 mmol, 40.4 mg), triflic anhydride (0.2 mmol, 33.6 µL) in $CDCl_3$. The reaction was stirred at 60 °C for 1 h and the compound **8·OTf** characterised *in situ* (95% spectroscopic yield); ^1H NMR (300 MHz, CD_3CN) δ 8.62 (dt, J = 15.4, 1.6, 1H), 7.92 – 7.81 (m, 4H, Ar), 7.79 – 7.61 (m, 15H, Ar), 7.41 – 7.33 (m, 6H, Ar), 4.39 (dd, J = 11.9, 1.6, 3H, CH_3); $^{13}C\{^1H\}$ NMR (75 MHz, CD_3CN) δ 144.1 (d, J = 6.3, PhC=CCH_3), 134.9 (d, J = 4.1), 133.8 (d, J = 13.5), 131.2 (d, J = 12.2), 129.5 (d, J = 16.5), 129.10 (d, J = 14.5), 128.7 (d, J = 7.5), 127.4, 121.2 (d, J = 16.4), 32.5 (CH_3); $^{31}P\{^1H\}$ NMR (202 MHz, CD_3CN) δ −104.0; $^{19}F\{^1H\}$ NMR (471 MHz, CD_3CN) δ −79.2; HRMS (ESI): calcd for $C_{21}H_{18}F_3O_3PS^+$ $[M+H]^+$: 451.0666, found: 451.0740.

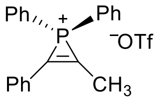

8•OTf

1,1-diphenyl-2,3-bis(4-(trifluoromethyl)phenyl)-1H-phosphiren-1-ium trifluoromethanesulfonate **9·OTf** was synthesised through general procedure A with 1,2-bis(4-(trifluoromethyl)phenyl)ethyne (0.2 mmol, 62.8 mg), diphenylphosphine oxide (0.2 mmol, 40.4 mg), triflic anhydride (0.2 mmol, 33.6 µL) in CD_3CN. The reaction was stirred at 60 °C for 1 h and the compound **9·OTf** was isolated as an orange solid (0.08 mmol, 49 mg) in 38% yield; suitable crystals were grown in CH_2Cl_2/pentane at r.t.; ^1H NMR (300 MHz, CD_3CN) δ 8.39 – 8.20 (m, 3H, Ar), 8.10 – 8.03 (m, 1H, Ar), 8.02 – 7.96 (m, 5H, Ar), 7.95 – 7.81 (m, 3H, Ar), 7.84 – 7.63 (m, 4H, Ar), 7.67 – 7.37 (m, 1H, Ar), 7.36 – 7.23 (m, 1H, Ar); $^{13}C\{^1H\}$ NMR (75 MHz, CD_3CN) δ 136.9 (d, J = 4.2), 135.5 (d, J = 13.7, Ar), 134.9 (d, J = 12.3, Ar), 134.46, 133.95 (d, J = 11.6 Hz, Ar), 133.34 (d, J = 8.7, Ar), 132.0, 131.2 (d, J = 16.5, Ar), 130.7 (d, J = 13.6, Ar), 127.8 – 127.3 (m, Ar), 124.54 (q, J = 272.2, CF_3), 116.4 (d, J = 94.0, Ar); $^{31}P\{^1H\}$ NMR (122 MHz, CD_3CN) δ −103.26 (d, J = 18.6). ^{19}F NMR (471 MHz, CD_3CN) δ −63.4 (CF_3), −79.2 (OTf); m.p. (°C) = 127-129; HRMS (ESI): calcd for $C_{29}H_{18}F_9O_3PS^+$ $[M]^+$: 648.0571, found: 648.0599.

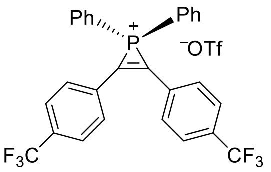

9•OTf

1,1-diphenyl-2,3-bis(4-(methoxy)phenyl)-1H-phosphiren-1-ium trifluoromethanesulfonate **10·OTf** was synthesised through general procedure A with 1,2-bis(4-(methoxy)phenyl)ethyne (0.2 mmol, 47.7 mg), diphenylphosphine oxide (0.2 mmol, 40.4 mg), triflic anhydride (0.2 mmol, 33.6 µL) in CD_3CN. The reaction was stirred at 60 °C for 1 h and the compound **10·OTf** was characterised *in situ* (64% spectroscopic yield); suitable crystals were grown in CH_2Cl_2/pentane at −18 °C. ^1H NMR (300 MHz, CD_3CN) δ 8.08 – 8.02 (m, 3H), 8.02 – 7.82 (m, 10H), 7.75 – 7.61 (m, 7H), 7.22 – 7.13 (m, 3H), 7.08 – 6.96 (m, 2H), 6.89 – 6.82 (m, 2H), 6.81 – 6.74 (m, 2H), 6.69 – 6.62 (m, 2H), 3.91 (s, 5H), 3.73 (s, 3H, OCH_3), 3.66 (s, 3H, OCH_3); $^{13}C\{^1H\}$ NMR (75 MHz, CD_3CN) δ 163.1, 160.0, 159.8, 135.5, 135.2, 133.9 (d, J = 13.5), 133.6, 133.3 (d, J = 9.9), 131.9 (d, J = 13.2), 131.2, 130.9 (d, J = 5.1), 130.3 (d, J = 13.0), 129.9 (d, J = 16.3), 129.5, 129.3, 115.0, 113.9, 113.2, 55.3, 54.6 (OCH_3); $^{31}P\{^1H\}$ NMR (202 MHz, CD_3CN) δ −110.9; $^{19}F\{^1H\}$ NMR (471 MHz, CD_3CN) δ −79.3; HRMS (ESI): calcd for $C_{29}H_{24}F_3O_5PS^+$ $[M]^+$: 572.0992, found: 572.1034.

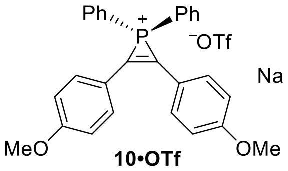

10•OTf

5. Optimisation catalytic reaction

Table S2 Optimisation of the catalytic reduction of carbonyls with silane

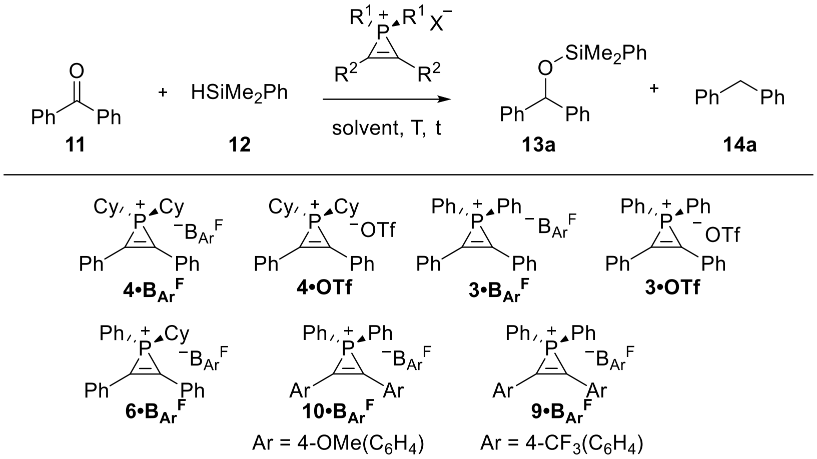

Entry	Catalyst[a]	Solvent	time	T (°C)	**13a** (%)	**14a** (%)	Conv of **12** (%)
			Catalyst optimisation				
1	**4 B_{Ar}^{F}** (10 mol%)	CD_3CN	18 h	r.t.	28	16	44
2	**4 B_{Ar}^{F}** (10 mol%)	CD_3CN	18 h	80	84	16	>99
3	**4 OTf** (10 mol%)	CD_3CN	18 h	80	/	82	82
4	**4·B_{Ar}^{F}** (10 mol%)[b]	CD_3CN	18 h	80	58	15	73
5	**3·B_{Ar}^{F}** (10 mol%)	CD_3CN	18 h	80	21	75	>99
6	**6·B_{Ar}^{F}** (10 mol%)	CD_3CN	18 h	80	11	89	>99
7	**10·B_{Ar}^{F}** (10 mol%)	CD_3CN	48 h	r.t. to 80	/	/	/
8	**10·OTf** (10 mol%)	CD_3CN	48 h	r.t. to 80	/	/	/
9	**9·B_{Ar}^{F}** (10 mol%)	CD_3CN	8 min	r.t.	/	>99	>99
10	**3·OTf** (10 mol%)	CD_3CN	5 min	r.t.	/	>99	>99
11	**3·OTf** (5 mol%)	CD_3CN	5 min	r.t.	/	>99	>99
			Blank reactions				
12	NaB_{Ar}^{F} (20 mol%)	CD_3CN	18 h	80	/	/	/
13	OTf_2 (20 mol%)	CD_3CN	18 h	80	/	/	/
14	$HP(O)Cy_2$ (20 mol%)	CD_3CN	18 h	80	/	/	/
15	$HP(O)Ph_2$ (20 mol%)	CD_3CN	18 h	80	/	/	/
16	NaB_{Ar}^{F}+OTf_2+$HP(O)Ph_2$ (10 mol%)	CD_3CN	18 h	80	/	/	/
17	NaB_{Ar}^{F}+OTf_2+$HP(O)Cy_2$+ diphenylacetylene (10 mol%)	CD_3CN	18 h	r.t. to 80	/	/	/
18	NaB_{Ar}^{F}+OTf_2+$HP(O)Ph_2$+ diphenylacetylene (10 mol%)	CD_3CN	18 h	r.t. to 80	/	/	/
19	NaOTf (20 mol%)	CD_3CN	36 h	r.t. to 80	/	/	/
20	OTf_2 +2,6-lut(10 mol%)	CD_3CN	18 h	80	/	/	/
21	TfOH +2,6-lut(10 mol%)	CD_3CN	18 h	80	/	/	/
22	TfOH (10 mol%)	CD_3CN	18 h	80	/	>99	>99
23	2,6-lutidine (10 mol%)	CD_3CN	18 h	80	/	/	/
24	OTf_2+2,6-lut+$Ph_2P(O)H$	CD_3CN	18 h	80 °C	/	/	/

	(10 mol%)						
25	$HPPh_2$ (10 mol%)	CD_3CN	18 h	80 °C	/	/	/
26	$HPPh_2$ + 2,6-lut + TfOH (10 mol%)	CD_3CN	18 h	80 °C	/	/	/
27	$HPPh_2$ + NaB$_{Ar}{}^{F}$ + diphenylacetylene (10 mol%)	CD_3CN	18 h	80 °C	/	/	/
27	$HPPh_2$ + OTf_2 + NaB$_{Ar}{}^{F}$ + diphenylacetylene (10 mol%)	CD_3CN	18 h	80 °C	73	/	73
28	Ph_4P_2 (10 mol%)	CD_3CN	18 h	80 °C	/	/	/
	Solvent optimisation						
30	**4 B**$_{Ar}{}^{F}$ (10 mol%)	THF	18 h	80	/	/	
31	**4·B**$_{Ar}{}^{F}$ (10 mol%)	C_6D_6	18 h	80	80	10	90[c]
32	**4·B**$_{Ar}{}^{F}$ (10 mol%)	$CDCl_3$	18 h	80	82	3	14[c]
33	**4·B**$_{Ar}{}^{F}$ (10 mol%)	CD_3CN	1 h	80	65	2	67
34	**4 B**$_{Ar}{}^{F}$ (10 mol%)	CD_3CN	3 h	80	85	3	88
	Temperature optimisation						
35	**4·B**$_{Ar}{}^{F}$ (10 mol%)	CD_3CN	18 h	40	/	/	/
36	**4 B**$_{Ar}{}^{F}$ (10 mol%) [b]	CD_3CN	18 h	60	35 (63 in 36 h)	13	48
37	**3 B**$_{Ar}{}^{F}$ (10 mol%) [isolated]	CD_3CN	5 h	80	>99	/	>99
38	**3 B**$_{Ar}{}^{F}$ (10 mol%) [isolated]	CD_3CN	5 h	r.t.	43	/	43
	Base optimisation and catalyst loading						
39	**4 B**$_{Ar}{}^{F}$ + 2,6-lut (5 mol%)	CD_3CN	5 h	80	>99	/	>99
40	**4·B**$_{Ar}{}^{F}$ (5 mol%)	CD_3CN	5 h	80	85	8	93
41	**4 B**$_{Ar}{}^{F}$ (2.5 mol%)	CD_3CN	5 h	80	73	/	73
42	**4·B**$_{Ar}{}^{F}$ + K_2CO_3(10 mol%)	CD_3CN	18 h	80	/	/	/
43	**3·B**$_{Ar}{}^{F}$ + K_2CO_3(10 mol%)	CD_3CN	18 h	80	/	/	/
44	**4·B**$_{Ar}{}^{F}$ + NEt_3 (10 mol%)	CD_3CN	18 h	80	/	/	/
45	**3·B**$_{Ar}{}^{F}$ + NEt_3 (10 mol%)	CD_3CN	18 h	80	59	/	59
46	**4 B**$_{Ar}{}^{F}$ + pyr (10 mol%)	CD_3CN	18 h	80	/	/	/
47	**3 B**$_{Ar}{}^{F}$ + pyr (10 mol%)	CD_3CN	18 h	80	52	/	52
48	**4 B**$_{Ar}{}^{F}$ + 2,6-lut (5 mol%)	CD_3CN	18 h	60	31	/	31
49	**3 B**$_{Ar}{}^{F}$ + 2,6-lut (5 mol%)	CD_3CN	5 h	60	>99	/	>99
50	**9 B**$_{Ar}{}^{F}$ + 2,6-lut (5 mol%)	CD_3CN	1 h	60	>99	/	>99
51	**10 B**$_{Ar}{}^{F}$ + 2,6-lut (5 mol%)	CD_3CN	18 h	80	/ (>99 in 58h)	/	/
	Optimisation of silane						
52	**4·B**$_{Ar}{}^{F}$ (10 mol%) + H_2SiPh_2 (0.25 mmol)	CD_3CN	36 h	r.t. to 80	/	/	/
53	**4·B**$_{Ar}{}^{F}$ (10 mol%) + H_3SiPh (0.25 mmol)	CD_3CN	36 h	r.t. to 80	/	/	/
54	**3·B**$_{Ar}{}^{F}$ +2,6-lut (10 mol%) $H_2SiPhMe$ (0.25 mmol)	CD_3CN	36 h	r.t. to 80	/	/	[d]
55	**3·B**$_{Ar}{}^{F}$ +2,6-lut (10 mol%) H_3SiPh (0.25 mmol)	CD_3CN	36 h	r.t. to 80	/	/	[d]

[a] Reaction condition: i) activation step $R_2P(O)H$ (10 mol%), OTf_2 (10 mol%), NaBAr^F (10 mol%), diphenylacetylene (10 mol%), CD_3CN (0.41 M), 60 °C, 30 min; ii) benzophenone **11** to dimethylphenyl silane **12** 1:1 (0.25 mmol), 80 °C, 18 h, unless otherwise stated, toluene used as internal standard (10 µL); [b] the volatiles, such as solvent and triflic acid, were removed prior addition of fresh solvent and starting materials; [c] pre-catalyst not fully soluble; [d] decomposition of phosphirenium observed with P-Si bond formation.

6. Substrate scope hydrosilylation

General procedure for the hydrosilylation of ketones and aldehydes

To a sealed Schlenk tube dicyclohexyl phosphine oxide (5 mol%, $12.5\cdot10^{-3}$ mmol, 2.7 mg) or diphenyl phosphine oxide (5 or 10 mol%, $12.5\cdot10^{-3}$ or $25\cdot10^{-3}$ mmol, 2.5 mg), diphenylacetylene (5 or 10 mol%,, $12.5\cdot10^{-3}$ mmol or $25\cdot10^{-3}$ mmol, 2.3 or 4.6 mg), triflic anhydride (5 or 10 mol%,, $12.5\cdot10^{-3}$ or $25\cdot10^{-3}$ mmol, 2.1 or 4.2 µL), $NaB_{Ar}{}^{F}$ (5 or 10 mol%, $12.5\cdot10^{-3}$ or $25\cdot10^{-3}$ mmol, 11.1 or 22.2 mg) were stirred in MeCN (3 mL, $8.3\cdot10^{-2}$ M) for 30 min at 60 °C in a J-Young NMR tube. After formation of pre-catalyst **3·$B_{Ar}{}^{F}$**, the reaction mixture was cooled to room temperature and 2,6-lutidine (5 or 10 mol%, $12.5\cdot10^{-3}$ or $25\cdot10^{-3}$ mmol, 1.5 or 3 µL), ketone (0.25 mmol), silane (0.25 mmol) were added and the reaction heated at 80 °C for 5 h, unless otherwise stated. After drying the volatiles pure product was obtained by purification by flash chromatography (9:1 Petroleum ether/EtOAc) or by trap-to-trap distillation. The reaction could not be performed in bigger vessels, e.g. Schlenk vials.

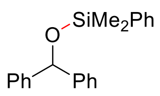

13a

(benzhydryloxy)dimethyl(phenyl)silane was synthesised following the general procedure with 5 mol% of **3·$B_{Ar}{}^{F}$**, with benzophenone (0.25 mmol, 45.5 mg), dimethylphenylsilane (0.25 mmol, 38.3 µL) after 5 h at 60 or 80 °C to yield **13a** as a colourless oil (0.245 mmol, 77.9 mg, 98% isolated yield) which data are in accordance to these reported in the literature.[10] **13a** was also synthesised with 2.5 mol% of **3·$B_{Ar}{}^{F}$** to yield the product in 73% after 5 h at 80 °C. ^{1}H NMR (400 MHz, $CDCl_3$) δ (ppm) 7.67-7.23 (m, 15H), 5.88 (s, 1H), 0.43 (s, 6H); ^{13}C{^{1}H} NMR (126 MHz, $CDCl_3$) δ (ppm) 145.6, 138.2, 134.1, 130.5, 128.7, 128.4, 127.6, 127.5, 77.4, –0.9.; ^{29}Si NMR (99 MHz, CD_3CN) δ 8.21.

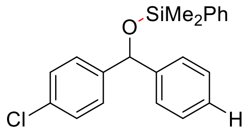

13b

((4-chlorophenyl)(phenyl)methoxy)dimethyl(phenyl)silane was synthesised following the general procedure with 5 mol% of **3·$B_{Ar}{}^{F}$**, with (4-chlorophenyl)(phenyl)methanone (0.25 mmol, 54.16 mg), dimethylphenylsilane (0.25 mmol, 38.3 µL) for 5 h at 80 °C to yield **13b** as a pale yellow solid (0.24 mmol, 85.6 mg, 97% isolated yield) which data are in accordance to these reported in the literature.[11] ^{1}H NMR (400 MHz, $CDCl_3$) δ (ppm) 7.49–7.51 (m, 2H), 7.31–7.41 (m, 3H), 7.19–7.28 (m, 9H), 5.69 (s, 1H), 0.29 (s, 6H); ^{13}C{^{1}H} NMR (126 MHz, $CDCl_3$) δ (ppm) 144.1, 143.2, 137.4, 133.5, 132.7, 129.7, 128.3, 128.3, 127.8, 127.8, 127.3, 126.4, 76.2, –1.1 (d, *J* = 16.8).

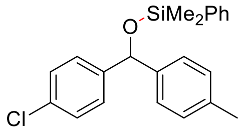

13c

((4-chlorophenyl)(p-tolyl)methoxy)dimethyl(phenyl)silane was synthesised following the general procedure with 5 mol% of **3·$B_{Ar}{}^{F}$**, with (4-chlorophenyl)(p-tolyl)methanone (0.25 mmol, 57.7 mg), dimethylphenylsilane (0.25 mmol, 38.3 µL) for 5 h at 80 °C to yield **13c** as a colourless solid (0.22 mmol, 80.7 mg, 88% isolated yield) which data are in accordance to these reported in the literature.[12] ^{1}H NMR (400 MHz, $CDCl_3$) δ (ppm) 7.49–7.51 (m, 2H), 7.31–7.41 (m, 3H), 7.22 (s, 4H), 7.14 (d, *J* = 8.0, 2H), 7.08 (d, *J* = 8.0, 2H), 5.66 (s, 1H), 2.30 (s, 3H), 0.28 (s, 6H); ^{13}C{^{1}H} NMR (126 MHz, $CDCl_3$) δ (ppm) 143.4, 141.2, 137.5, 136.9, 133.5, 132.6, 129.6, 129.0, 128.3, 127.8, 127.7, 126.4, 76.0, 21.1, −1.1 (d, *J* = 16.8).

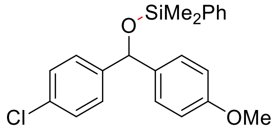

13d

((4-chlorophenyl)(4-methoxyphenyl)methoxy)dimethyl(phenyl)silane was synthesised following the general procedure with 5 mol% of **3·$B_{Ar}{}^{F}$**, with (4-chlorophenyl)(4-methoxyphenyl)methanone (0.25 mmol, 61.7 mg), dimethylphenylsilane (0.25 mmol, 38.3 µL) for 5 h at 80 °C to yield **13d** as a colourless solid (0.21 mmol, 79.3 mg, 83% isolated yield). Attempt to synthesise enantioenriched **13d** was made by utilising (*S*)-*tert*-butylphenyl phosphine oxide (5 mol%, $12.5\cdot10^{-3}$ mmol, 2.3 mg), 4-ethynyl-α,α,α-trifluorotoluene (5 mol%, $12.5\cdot10^{-3}$ mmol, 2 µL) at 60 °C for 48 h (76%); following the reaction by ^{31}P{^{1}H} NMR spectroscopy, we observe a peak at −93 ppm, in line with data obtained for other phosphirenium species (see section 4). However the reaction yields **13d** as a racemic product, as observed by chiral HPLC. ^{1}H NMR (500 MHz, $CDCl_3$) δ (ppm) 7.56 – 7.51 (m, 2H, *m*-C_6H_4Cl), 7.44 – 7.35 (m, 4H, SiPh), 7.26 (s, 3H, *o*-C_6H_4Cl, SiPh), 7.21 – 7.17 (m, 2H, *m*-C_6H_4OMe), 6.87 – 6.81 (m, 2H, *o*-C_6H_4OMe), 5.69 (s, 1H, CHSi), 3.81 (s, 3H, OMe), 0.32 (d, *J* = 1.5, 6H, $SiMe_2$). ^{13}C{^{1}H} NMR (126 MHz, $CDCl_3$) δ (ppm) 158.9 (*i*-C_6H_4OMe), 143.6 (*q*-C_6H_4Cl), 137.6 (SiPh), 136.6 (*q*-C_6H_4OMe), 133.7 (*m*-C_6H_4Cl), 133.1 (*i*-C_6H_4Cl), 132.7 (SiPh), 129.8 (SiPh), 128.4 (*o*-C_6H_4Cl), 127.9 (SiPh), 127.8 (*m*-C_6H_4OMe), 113.8 (*o*-C_6H_4OMe), 75.9 (<u>C</u>HSi), 55.3 (OMe), -1.0 (d, *J* = 16.8 Hz, $SiMe_2$). m.p.

= 68-71 °C; chiral HPLC analysis, Chiralcel IB 9(7:3 pentane: iPrOH, flow rate 0.5 mL min-1 220 nm) tr 40.8, 42.1 min: 49.7:50.1.

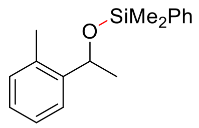

13e

dimethyl(phenyl)(1-(*o*-tolyl)ethoxy)silane was synthesised following the general procedure with 5 mol% of **3·B**$_{Ar}^{F}$, with 1-(*o*-tolyl)ethan-1-one (0.25 mmol, 32.7 µL), dimethylphenylsilane (0.25 mmol, 38.3 µL) for 5 h at 80 °C to yield **13e** as a colorless oil (0.19 mmol, 52.06 mg, 77% isolated yield) which data are in accordance to these reported in the literature.[13] ^{1}H NMR (400 MHz, $CDCl_3$) δ (ppm) 7.57–7.61 (m, 2H), 7.21–7.44 (m, 3H), 4.81 (q, *J* = 6.3, 1H), 2.10 (s, 3H), 1.41 (d, *J* = 6.3, 3H), 0.50 ppm (s, 3H); ^{13}C{^{1}H} NMR (126 MHz, $CDCl_3$) δ (ppm) 146.4, 138.3, 133.7, 129.6, 128.3, 127.9, 127.0, 125.5, 68.8, 25.9, 21.0, −2.2ppm.

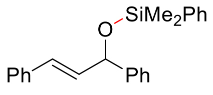

13f

(*E*)-((1,3-diphenylallyl)oxy)dimethyl(phenyl)silane was synthesised following general procedure with 5 mol% of **3·B**$_{Ar}^{F}$, with chalcone (0.25 mmol, 41.5 mg), dimethylphenylsilane (0.25 mmol, 38.3 µL) for 18 h at 80 °C to yield **13f** as a colourless oil (0.22 mmol, 50.9 mg, 87% isolated yield) which data are in accordance to these reported in the literature;[14] ^{1}H NMR (500 MHz, $CDCl_3$) δ 7.64 – 7.55 (m, 2H), 7.51 – 7.45 (m, 2H), 7.44 – 7.14 (m, 11H), 6.44 (d, *J* = 15.9, 1H), 6.18 (dd, *J* = 15.8, 6.6, 1H), 5.24 (d, *J* = 6.5, 1H) , 0.32 ($SiCH_3$), 0.28 ($SiCH_3$); ^{13}C NMR (126 MHz, $CDCl_3$) δ 143.5, 138.0, 136.9, 133.7, 132.7, 129.7, 129.6, 128.6, 128.4, 127.9, 127.7, 127.4, 126.7, 126.4, 76.1,–0.6, −0.8. HRMS (ESI): calcd for $C_{23}H_{24}OSi^+$ [M+H]$^+$: 345.1596, found: 345.1674

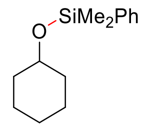

13g

(cyclohexyloxy)dimethyl(phenyl)silane was synthesised following general procedure with 5 mol% of **3·B**$_{Ar}^{F}$, with cyclohexanone (0.25 mmol, 25.9 µL), dimethylphenylsilane (0.25 mmol, 38.3 µL) for 18 h at 80 °C to yield **13g** as a colourless oil (0.22 mmol, 50.9 mg, 87% isolated yield) which data are in accordance to these reported in the literature.[15] ^{1}H NMR (400 MHz, $CDCl_3$) δ (ppm) 7.57-7.60 (m, 2H), 7.35-7.37 (m, 3H), 1.14-1.77 (m, 11H), 0.37 (s, 6H); ^{13}C{^{1}H} NMR (126 MHz, $CDCl_3$) δ (ppm) 138.9, 133.5, 129.4, 127.7, 71.4, 35.9, 25.6, 24.4, −0.8.

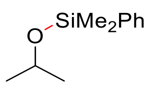

13h

isopropoxydimethyl(phenyl)silane was synthesised following the general procedure with 5 mol% of **3·B**$_{Ar}^{F}$, with acetone (0.25 mmol, 18.5 µL), dimethylphenylsilane (0.25 mmol, 38.3 µL) for 18 h at 80 °C to yield **13h** as a colourless oil (0.16 mmol, 31.1 mg, 62% isolated yield) which data are in accordance to these reported in the literature.[15] ^{1}H NMR (400 MHz, $CDCl_3$) δ (ppm) 7.58-7.59 (m, 2H), 7.33-7.34 (m, 3H), 3.95-4.01 (m, 1H), 1.12-1.13 (m, 6H), 0.37 (s, 6H); ^{13}C{^{1}H} NMR (126 MHz, $CDCl_3$) δ (ppm) 138.6, 133.5, 129.5, 127.8, 65.3, 25.7, −1.1.

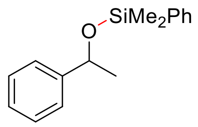

13i

dimethyl(phenyl)(1-phenylethoxy)silane was synthesised following the general procedure with 10 mol% of **3·B**$_{Ar}^{F}$, with acetophenone (0.25 mmol, 29.2 µL), dimethylphenylsilane (0.25 mmol, 38.3 µL) for 18 h at 80 °C to yield **13i** as a colourless oil ($6.5 \cdot 10^{-2}$ mmol, 16.8 mg, 23% isolated yield) which data are in accordance to these reported in the literature.[16] ^{1}H NMR (400 MHz, $CDCl_3$) δ (ppm) 7.57–7.61 (m, 2H), 7.21–7.44 (m, 3H), 4.79 (q, *J* = 6.3, 1H; CH), 1.38 (d, *J* = 6.3 Hz, 3H; CH_3), 0.31, 0.26 (s, 6H); ^{13}C{^{1}H} NMR (126 MHz, $CDCl_3$) δ (ppm) 146.4, 138.3, 133.7, 129.6, 128.3, 127.9, 127.0, 125.5, 71.5, 27.3, −1.2.

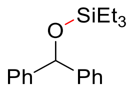

13j

(benzhydryloxy)triethylsilane was synthesised following the general procedure with 5 mol% of **4·B**$_{Ar}^{F}$, with benzophenone (0.25 mmol, 45.5 mg), triethylsilane (0.25 mmol, 39.9 µL) for 5 h at 80 °C to yield **13j** as a colourless oil (0.21 mmol, 63.4 mg, 85% isolated yield) which data are in accordance to these reported in the literature;[17] ^{1}H NMR (500 MHz, $CDCl_3$) δ (ppm) 7.36 (d, *J* = 7.2, 4H, Ar), 7.32 – 7.26 (m, 4H, Ar), 7.23 – 7.18 (m, 2H, Ar), 5.76 (s, 1H, CH), 0.88 (t, *J* = 8.0, 9H, $SiCH_2CH_3$), 0.57 (q, *J* = 7.9, 6H, $SiCH_2CH_3$). ^{13}C{^{1}H} NMR (126 MHz, $CDCl_3$) δ (ppm) 145.4 (*q*-C_6H_5), 128.3 (*m*-C_6H_5), 127.1 (*p*-C_6H_5), 126.5 (*o*-C_6H_5), 76.5 (CH), 6.9 ($SiCH_2CH_3$), 5.0 ($SiCH_2CH_3$).

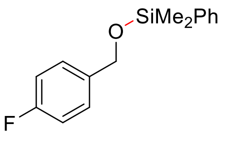

13k

((4-fluorobenzyl)oxy)dimethyl(phenyl)silane was synthesised following the general procedure with 5 mol% of **4·B**$_{\mathbf{Ar}}$$^{\mathbf{F}}$, with 4-fluorobenzaldehyde (0.25 mmol, 26.8 µL), dimethylphenylsilane (0.25 mmol, 38.3 µL) for 18 h at 80 °C to yield **13k** as a colourless oil (0.24 mmol, 63.1 mg, 97% isolated yield) which data are in accordance to these reported in the literature.[18] ^{1}H NMR (500 MHz, $CDCl_3$) δ (ppm) 7.65 –7.59 (m, 2H), 7.46 – 7.37 (m, 3H), 7.31 –7.25 (m, 2H), 7.06 –6.97 (m, 2H), 4.67 (2H), 0.45 (s, 6H); ^{13}C{^{1}H} NMR (126 MHz, $CDCl_3$) δ (ppm) 162.1 (d, *J* = 244.4, CF), 137.5, 136.5 (d, *J* = 2.9), 133.6, 129.9, 128.4 (d, *J* = 8.3), 128.1, 115.2 (d, *J* = 21.1), 64.5, −1.6.

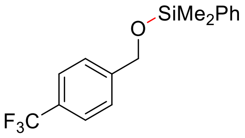

13l

dimethyl(phenyl)((4-(trifluoromethyl)benzyl)oxy)silane was synthesised following the general procedure with 5 mol% of **3·B**$_{\mathbf{Ar}}$$^{\mathbf{F}}$, with 4-(trifluoromethyl)benzaldehyde (0.25 mmol, 34.1 µL), dimethylphenylsilane (0.25 mmol, 38.3 µL) for 18 h at 80 °C to yield **13l** as a colourless oil (0.23 mmol, 71.4 mg, 92% isolated yield) which data are in accordance to these reported in the literature.[19] ^{1}H NMR (500 MHz, $CDCl_3$) δ (ppm) 7.80-7.78 (m, 2H), 7.64-7.52 (m, 7H), 4.76 (s, 2H), 0.57 (s, 6H); ^{13}C{^{1}H} NMR (126 MHz, $CDCl_3$) δ (ppm) 139.8, 138.2, 137.5, 133.8, 131.0, 130.3, 127.9, 127.5, 123.4, 68.6, −1.5; ^{19}F NMR (376 MHz, $CDCl_3$) δ (ppm) −62.6.

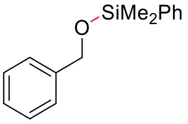

13m

(benzyloxy)dimethyl(phenyl)silane was synthesised following the general procedure with 5 mol% of **3·B**$_{\mathbf{Ar}}$$^{\mathbf{F}}$, with benzaldehyde (0.25 mmol, 25.5 µL), dimethylphenylsilane (0.25 mmol, 38.3 µL) for 18 h at 80 °C to yield **13m** as a colourless oil (0.245 mmol, 59.4 mg, 98% isolated yield) which data are in accordance to these reported in the literature.[15] ^{1}H NMR (400 MHz, $CDCl_3$) δ (ppm) 7.58-7.60 (m, 2H), 7.20-7.37 (m, 8H), 4.69 (s, 2H), 0.40 (s, 6H); ^{13}C{^{1}H} NMR (126 MHz, $CDCl_3$) δ (ppm) 140.7, 137.6, 133.5, 129.7, 128.2, 127.9, 127.1, 126.5, 65.0, −1.7.

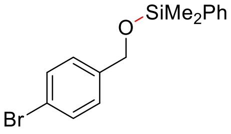

13n

((4-bromobenzyl)oxy)dimethyl(phenyl)silane was synthesised following the general procedure with 5 mol% of **3·B**$_{\mathbf{Ar}}$$^{\mathbf{F}}$, with 4-bromobenzaldehyde (0.25 mmol, 46.2 mg), dimethylphenylsilane (0.25 mmol, 38.3 µL) for 18 h at 80 °C to yield **13n** as a colourless solid (0.22 mmol, 71.5 mg, 89% isolated yield) which data are in accordance to these reported in the literature.[20] ^{1}H NMR (400 MHz, $CDCl_3$) δ (ppm) 7.61-7.57 (m, 2H), 7.48-7.38 (m, 5H), 7.17 (m, 2H), 4.64 (s, 2H), 0.42 (s, 6H); ^{13}C{^{1}H} NMR (126 MHz, $CDCl_3$) δ (ppm) 139.8, 137.3, 133.6, 131.38, 129.9, 128.3, 128.0, 120.9, 64.3, −1.7

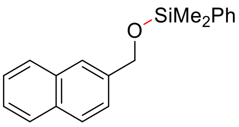

13o

dimethyl(naphthalen-2-ylmethoxy)(phenyl)silane was synthesised following the general procedure with 5 mol% of **3·B**$_{\mathbf{Ar}}$$^{\mathbf{F}}$, with naphthaldehyde (0.25 mmol, 39.0 mg), dimethylphenylsilane (0.25 mmol, 38.3 µL) for 18 h at 80 °C to yield **13o** as a colourless oil (0.18 mmol, 54.1 mg, 74% isolated yield) ^{1}H NMR (300 MHz, $CDCl_3$) δ 7.94 – 7.71 (m, 4H), 7.71 – 7.55 (m, 2H), 7.58 – 7.17 (m, 5H), 4.97 – 4.80 (m, 2H), 0.45 (s, 6H). ^{13}C{^{1}H} NMR (75 MHz, $CDCl_3$) δ 138.3, 137.7, 133.7, 133.5, 132.9, 129.9, 128.1, 127.9, 127.8, 126.1, 125.7, 125.1, 65.3, −1.5; HRMS (ESI): calcd for $C_{19}H_{20}OSi^{+}$ $[M+Na]^{+}$: 315.1181, found: 315.1158.

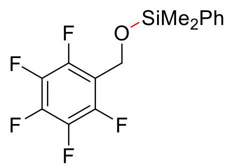

13p

dimethyl((perfluorophenyl)methoxy)(phenyl)silane was synthesised following the general procedure with 5 mol% of **3·B**$_{\mathbf{Ar}}$$^{\mathbf{F}}$, with pentafluorobenzaldehyde (0.25 mmol, 30.8 mg), dimethylphenylsilane (0.25 mmol, 38.3 µL) for 18 h at 80 °C to yield **13p** as a colourless oil (0.15 mmol, 49.85 mg, 60% isolated yield); ^{1}H NMR (400 MHz, $CDCl_3$) δ 7.67 – 7.48 (m, 2H), 7.41 – 7.31 (m, 3H), 4.70 (t, *J* = 1.8, 2H), 0.42 (s, 6H), traces of CD_3CN. ^{19}F{^{1}H} NMR (376 MHz, $CDCl_3$) δ −143.30 – −143.77 (m), −154.8 – −155.0 (m), −162.2 – −162.7 (m); ^{13}C{^{1}H} NMR (75 MHz, $CDCN_3$) δ 158.5 (quat C), 135.7 (Ar), 134.6 (d, *J* = 42.0 Hz, CF), 133.9 (Ar), 132.3 (Ar), 130.5 (d, *J* = 43.8 Hz, CF), 128.9 (Ar), 128.8 (Ar), −2.1 ($SiCH_3$); HRMS (ESI): calcd for $C_{15}H_{13}F_5OP^{+}$ $[M]^{+}$: 332.0656, found: 332.0635.

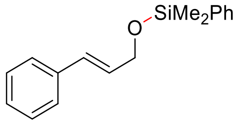

13q

(cinnamyloxy)dimethyl(phenyl)silane was synthesised following the general procedure with 5 mol% of $\mathbf{3\cdot B_{Ar}^{F}}$, with cinnamaldehyde (0.25 mmol, 31.5 µL), dimethylphenylsilane (0.25 mmol, 38.3 µL) for 18 h at 80 °C to yield **13q** as a colourless oil (0.19 mmol, 50.3 mg, 75% isolated yield) which data are in accordance to these reported in the literature.[21] ^{1}H NMR (400 MHz, $CDCl_3$) δ (ppm) 7.6-7.7(m, 2H), 7.1-7.5(m, 8H), 6.55(d, *J* = 16.5, 1H), 6.19 (dt, *J* = 16.5, 5.1, 1H), 4.28 (d, *J* = 5.1, 2H), 0.43 (s, 6H).

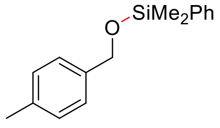

13r

dimethyl((4-methylbenzyl)oxy)(phenyl)silane was synthesised following the general procedure with 5 mol% of $\mathbf{3\cdot B_{Ar}^{F}}$, with 4-methylbenzaldehyde (0.25 mmol, 29.5 µL), dimethylphenylsilane (0.25 mmol, 38.3 µL) for 18 h at 80 °C to yield **13r** as a colourless oil (0.22 mmol, 57 mg, 89% isolated yield) which data are in accordance to these reported in the literature.[22] ^{1}H NMR (400 MHz, $CDCl_3$) δ (ppm) 7.62-7.12 (m, 9H), 4.70 (s, 2H), 2.34 (s, 3H), 0.36 (s, 3H), 0.35 (s, 3H); ^{13}C{^{1}H} NMR (126 MHz, $CDCl_3$) δ (ppm) 138.3, 136.5, 128.9,126.3, 64.6, 21.1, 6.8, 4.5.

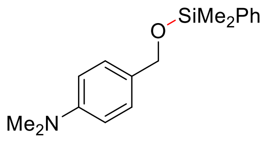

13s

4-(((dimethyl(phenyl)silyl)oxy)methyl)-N,N-dimethylaniline was synthesised the following general procedure with 10 mol% of $\mathbf{3\cdot B_{Ar}^{F}}$, with 4-(dimethylamino)benzaldehyde (0.25 mmol, 37.3 mg), dimethylphenylsilane (0.25 mmol, 38.3 µL) for 18 h at 80 °C to yield **13s** as a yellow solid ($5.5\cdot10^{-2}$ mmol, 15.7 mg, 22% conversion) which data are in accordance to these reported in the literature.[21] ^{1}H NMR (400 MHz, $CDCl_3$) δ (ppm) 7.60 (m, 2H), 7.15 (m, 5H), 6.57 (m, 2H), 4.65 (s, 2H), 2.47 (s, 6H), 0.32(s, 6H); ^{13}C{^{1}H} NMR (126 MHz, $CDCl_3$) δ (ppm) 161.3, 138.5, 133.9, 129.7, 129.3, 128.4, 128.3, 128.1,127.7, 122.8, 65.6, 40.5,−1.1.

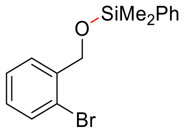

13t

(benzhydryloxy)dimethyl(phenyl)silane was synthesised following general procedure with 10 mol% of $\mathbf{3\cdot B_{Ar}^{F}}$ with 2-bromobenzophenone (0.25 mmol, 29.2 µL), dimethylphenylsilane (0.25 mmol, 38.3 µL) for 18 h at 80 °C to yield **13t** as a colourless oil (0.25 mmol, 50% isolated yield) which data are in accordance to these reported in the literature.[23] ^{1}H NMR (300 MHz, $CDCl_3$) δ 7.67 – 7.61 (m, 2H), 7.61 – 7.54 (m, 1H), 7.50 (dd, *J* = 7.9, 1.2, 1H), 7.44 – 7.38 (m, 3H), 7.34 (td, *J* = 7.5, 1.2, 1H), 7.13 (dddd, *J* = 8.0, 7.3, 1.7, 0.8, 1H), 4.75 (s, 2H), 0.47 (s, 6H). ^{13}C{^{1}H} NMR (75 MHz, $CDCl_3$) δ 139.8 (C-Br), 137.4 (Si-C_6H_5), 133.6 (Ar), 132.2 (Ar), 129.9 (Ar), 128.5 (Ar), 128.3 (Ar), 128.0 (*o*-CH), 127.4 (Ar), 121.4 (*q*-C), 64.6 (CH_2), −1.6 (Si-CH_3).

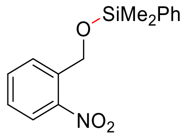

13u

dimethyl((2-nitrobenzyl)oxy)(phenyl)silane was synthesised following general procedure with 10 mol% of $\mathbf{3\cdot B_{Ar}^{F}}$ with 2-nitrobenzophenone (0.25 mmol, 37.8 mg), dimethylphenylsilane (0.25 mmol, 38.3 µL) for 18 h at 80 °C to yield **13u** as a colourless oil (0.11 mmol, 30.4 mg, 43% isolated yield); ^{1}H NMR (300 MHz, $CDCl_3$) δ 8.09 (dd, *J* = 8.2, 1.3, 1H), 7.94 (dq, *J* = 7.8, 1.2, 1H), 7.70 – 7.63 (m, 1H), 7.63 – 7.56 (m, 2H), 7.45 – 7.33 (m, 4H), 5.10 (d, *J* = 1.0, 2H), 0.47 (s, 6H); ^{13}C{^{1}H} NMR (75 MHz, $CDCl_3$) δ 137.5 (Cq), 136.7 (Cq), 133.6 (Ar), 133.1 (Ar), 129.6 (*o*-CH), 127.8 (Ar), 127.7 (Ar), 127.2 (Ar), 124.3 (Ar), 61.7 (CH_2), −2.18 ($SiCH_3$); HRMS (ESI): calcd for $C_{15}H_{17}NO_3Si^+$ $[M+Na]^+$: 310.0875, found: 310.0880.

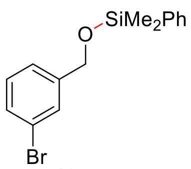

13v

((3-bromobenzyl)oxy)dimethyl(phenyl)silane was synthesised following the general procedure with 10 mol% of $\mathbf{3\cdot B_{Ar}^{F}}$, with 3-bromobenzophenone (0.25 mmol, 29.2 µL), dimethylphenylsilane (0.25 mmol, 38.3 µL) for 18 h at 80 °C to yield **13v** as a colourless solid ($7.5\cdot10^{-2}$ mmol, 24 mg, 30% isolated yield) which data are in accordance to these reported in the literature.[21] ^{1}H NMR (500 MHz, CD_3CN) δ (ppm) 8.04 (t, *J* = 1.9 Hz, 1H), 7.88 (d, *J* = 7.6, 1H), 7.82 (s, 2H), 7.68 – 7.63 (m, 2H), 7.63 – 7.56 (m, 3H), 4.71 (s, 2H), 0.37 (2 s, 6H); HRMS (ESI): calcd for $C_{15}H_{17}BrOSi^+$ $[M]^+$: 320.0232, found: 320.0172.

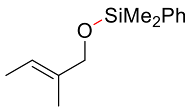

13w

(*E*)-dimethyl((2-methylbut-2-en-1-yl)oxy)(phenyl)silane was synthesised following the general procedure with 10 mol% of $\mathbf{3\cdot B_{Ar}^{F}}$, with (*E*)-2-methylbut-2-enal (0.25 mmol, 24.2 µL), dimethylphenylsilane (0.25 mmol, 38.3 µL) for 18 h at 80 °C to form **13w** in 41% spectroscopic yield and characterised *in situ*, traces of reduced alkene were observed; ^{1}H NMR (300 MHz, $CDCl_3$) δ 7.73 – 6.87 (m, 42H, Ar), 5.82 (ddt, *J* = 16.9, 10.1, 6.7, 2H, side-

product), 5.08 (s, 1H, CH) – 4.87 (m, 5H, side-product), 4.07 (t, *J* = 6.8, 2H, CH_2), 3.03 – 2.81 (m, 4H, minor), 2.59 (dd, *J* = 9.1, 6.9, 2H), 2.20 – 1.96 (m, 6H, CH_3), 1.43 (s, 18H, CH_3), 1.39 (d, *J* = 13.2 Hz, 5H, CH_3), 1.27 (d, *J* = 9.7 Hz, 85H, CH_3), 0.93 – 0.77 (m, 10H, $SiCH_3$); $^{13}C\{^1H\}$ NMR (75 MHz, $CDCl_3$) δ 174.1, 165.9, 162.2, 130.1, 128.3, 64.0, 36.2, 31.0, 30.3, 18.8, 13.4; HRMS (ESI): calcd for $C_{13}H_{20}OSi^+$ $[M+Na]^+$: 343.1181, found: 343.1197.

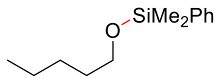

13x

dimethyl(pentyloxy)(phenyl)silane was synthesised following the general procedure with 10 mol% of **3·B$_{Ar}$^{F}**, with pentanal (0.25 mmol, 26.6 µL), dimethylphenylsilane (0.25 mmol, 38.3 µL) for 18 h at 80 °C to yield **13x** as a colourless oil (0.22 mmol, 55.1 mg, 72% isolated yield); ^1H NMR (300 MHz, $CDCl_3$) δ 7.59 (dddt, *J* = 12.3, 6.7, 3.0, 1.7 Hz, 9H), 7.39 (dq, *J* = 8.8, 3.8, 3.0, 14H), 3.60 (t, *J* = 6.7, 2H), 1.32 – 1.23 (m, 12H), 0.89 (pd, *J* = 8.0, 7.1, 2.9, 11H), 0.41 (d, *J* = 9.9, 10H), 0.35 (s, 9H); $^{13}C\{^1H\}$ NMR (75 MHz, $CDCl_3$) δ 139.9, 133.6, 129.4, 127.8, 63.3, 32.4, 28.1, 22.6, 14.2, 1.2, −1.6; HRMS (ESI): calcd for $C_{13}H_{22}OSi^+$ $[M+Na]^+$: 245.1338, found: 245.1361.

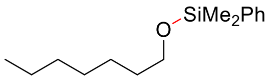

13y

(heptyloxy)dimethyl(phenyl)silane was synthesised following the general procedure with 10 mol% of **3·B$_{Ar}$^{F}**, with heptanal (0.25 mmol, 35.3 µL), dimethylphenylsilane (0.25 mmol, 38.3 µL) for 18 h at 80 °C to yield **13y** as a colourless solid (0.22 mmol, 55.1 mg, 88% isolated yield);^1H NMR (300 MHz, $CDCl_3$) δ 7.64 – 7.51 (m, 2H, Ar), 7.42 – 7.35 (m, 3H, Ar), 3.58 (t, *J* = 6.7, 2H, OCH_2), 1.51 (q, *J* = 6.8, 2H, $CH_2CH_2CH_2$), 1.35 – 1.16 (m, 8H, CH_2), 0.92 – 0.80 (m, 3H, CH_3), 0.38 (s, 6H, $SiCH_3$); $^{13}C\{^1H\}$ NMR (75 MHz, $CDCl_3$) δ 138.2, 133.6, 129.6, 127.9, 63.3, 32.8, 31.9, 29.2, 25.9, 22.7, 14.2, 1.2, −1.6 ($SiCH_3$); HRMS (ESI): calcd for $C_{15}H_{26}OSi^-$ $[M-H]^-$: 249.1753, found: 249.1676.

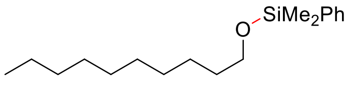

13z

(decyloxy)dimethyl(phenyl)silane was synthesised following the general procedure with 10 mol% of **3·B$_{Ar}$^{F}**, with decanal (0.25 mmol, 55.5 µL), dimethylphenylsilane (0.25 mmol, 38.3 µL) for 18 h at 80 °C to yield **13z** as a colourless solid (0.19 mmol, 57.7 mg, 79% isolated yield); ^1H NMR (300 MHz, $CDCl_3$) δ 7.57 – 7.42 (m, 2H), 7.34 – 7.25 (m, 3H), 3.51 (t, *J* = 6.7, 2H), 1.60 – 1.32 (m, 2H), 1.31 – 1.03 (m, 16H), 0.93 – 0.71 (m, 3H), 0.28 (d, *J* = 13.5, 6H); $^{13}C\{^1H\}$ NMR (75 MHz, $CDCl_3$) δ 138.2, 133.6, 129.7, 127.9, 63.3 (OCH_2), 32.8, 32.1, 29.8, 29.7, 29.5, 25.9, 22.8, 14.3, 1.2, −1.6 ($SiCH_3$); HRMS (ESI): calcd for $C_{18}H_{32}OSi^+$ $[M]^+$: 292.2222, found: 292.2243.

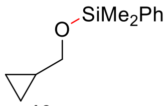

13aa

(cyclopropylmethoxy)dimethyl(phenyl)silane was synthesised following the general procedure with 10 mol% of **3·B$_{Ar}$^{F}**, with cyclopropanecarbaldehyde (0.25 mmol, 18.7 µL), dimethylphenylsilane (0.25 mmol, 38.3 µL) for 18 h at 80 °C to yield **13aa** through trap-to-trap distillation as a colourless solid (0.19 mmol, 39.7 mg, 77% isolated yield); ^1H NMR (400 MHz, $CDCl_3$) δ 7.64 – 7.57 (m, 2H), 7.41 – 7.34 (m, 3H), 3.44 (d, *J* = 6.7, 2H), 1.03 (ttt, *J* = 8.0, 6.7, 4.8, 1H), 0.51 – 0.44 (m, 2H), 0.40 (s, 6H), 0.13 (dt, *J* = 5.9, 4.5, 2H). $^{13}C\{^1H\}$ NMR (101 MHz, $CDCl_3$) δ 138.1, 133.6, 129.7, 127.9, 67.9 (OCH_2), 13.3 (CH), 3.2 (CH_2), −1.5 ($SiCH_3$); HRMS (ESI): calcd for $C_{12}H_{18}OSi^+$ $[M+H]^+$: 207.1127, found: 207.1200.

6.1 Deoxygenation reaction

To a sealed Schlenk tube diphenyl phosphine oxide (5 mol%, $12.5\cdot10^{-3}$ mmol, 2.5 mg), diphenylacetylene (5 mol%, $12.5\cdot10^{-3}$ mmol, 2.3 mg), triflic anhydride (5 mol%, $12.5\cdot10^{-3}$ mmol, 2.1 µL) were stirred in MeCN (3 mL, $8.3\cdot10^{-2}$ M) for 30 min at 60 °C. After formation of pre-catalyst **3·OTf**, the reaction mixture was cooled down benzophenone (0.25 mmol, 45.5 mg) and dimethylphenylsilane (0.25 mmol, 38.3 µL) were added and the reaction stirred at r.t. for 5 min or 18 h. After drying the volatile, pure product was obtained by purification by flash chromatography (5:1 Petroleum ether/EtOAc).

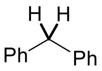

14a

diphenylmethane was synthesised following the general procedure with 5 mol% of **3·OTf**, with benzophenone (0.25 mmol, 45.5 mg), dimethylphenylsilane (0.25 mmol, 38.3 µL) for 5 min at r.t. to yield **14a** as a colourless oil (0.22 mmol, 37.8 mg, 90% isolated yield) which data are in accordance to these reported in the literature;[24] ^1H NMR (400 MHz, $CDCl_3$) δ (ppm) 7.31 (dd, *J* = 8.5, 6.9 Hz, 4H), 7.26 - 7.20 (m, 6H), 4.02 (s, 2H); $^{13}C\{^1H\}$ NMR (126 MHz, $CDCl_3$) δ (ppm) 141.1, 128.9, 128.5, 126.1, 41.9.

7. Mechanistic investigation

7.1 Understanding the pre-catalyst activation step

When analysing the reaction between $Cy_2P(O)H$ (0.35 mmol) and 1,2-bis(4-(trifluoromethyl)phenyl)ethyne (0.35 mmol) by $^{31}P\{^1H\}$ inv gated NMR, consumption of the phosphine oxide is observed at r.t. with formation of two AB systems at 17 and −14 ppm ($^1J_{P\text{-}P}$ = 351 Hz, Figure S2, insert) and the phosphirenium species at −80 ppm (Figure S2).[25] A peak at 119 ppm appears which could be related to a potential [Cy_2P][OTf] phosphenium species.[26-27]

Heating the mixture up in the spectrometer, the AB spin systems disappears, while the phosphirenium peak at −80 ppm increases to 17% at 313.15K to 45% (40 min) at 343.15K (70 min), Figure S3 and S4. The reaction mixture is extremely complex and the other species present in solution were not identified. The analysis was performed in presence of PPh_3 (0.2 M) in CD_3CN added in a sealed glass capillary, which peak appears at −5.2 ppm.

For completeness, the $^{19}F\{^1H\}$ NMR at different temperatures is reported in comparison to the alkyne (Figure S5); while complete conversion of the alkyne is observed, formation of multiple signals are found in the CF_3 region at −63 ppm and the counterion region at −79 ppm, which confirm the complexity of the reaction mixture.

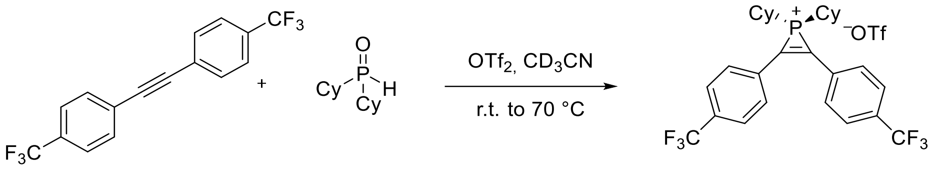

Figure S2 $^{31}P\{^1H\}$ inv gated NMR after 10 min at r.t.

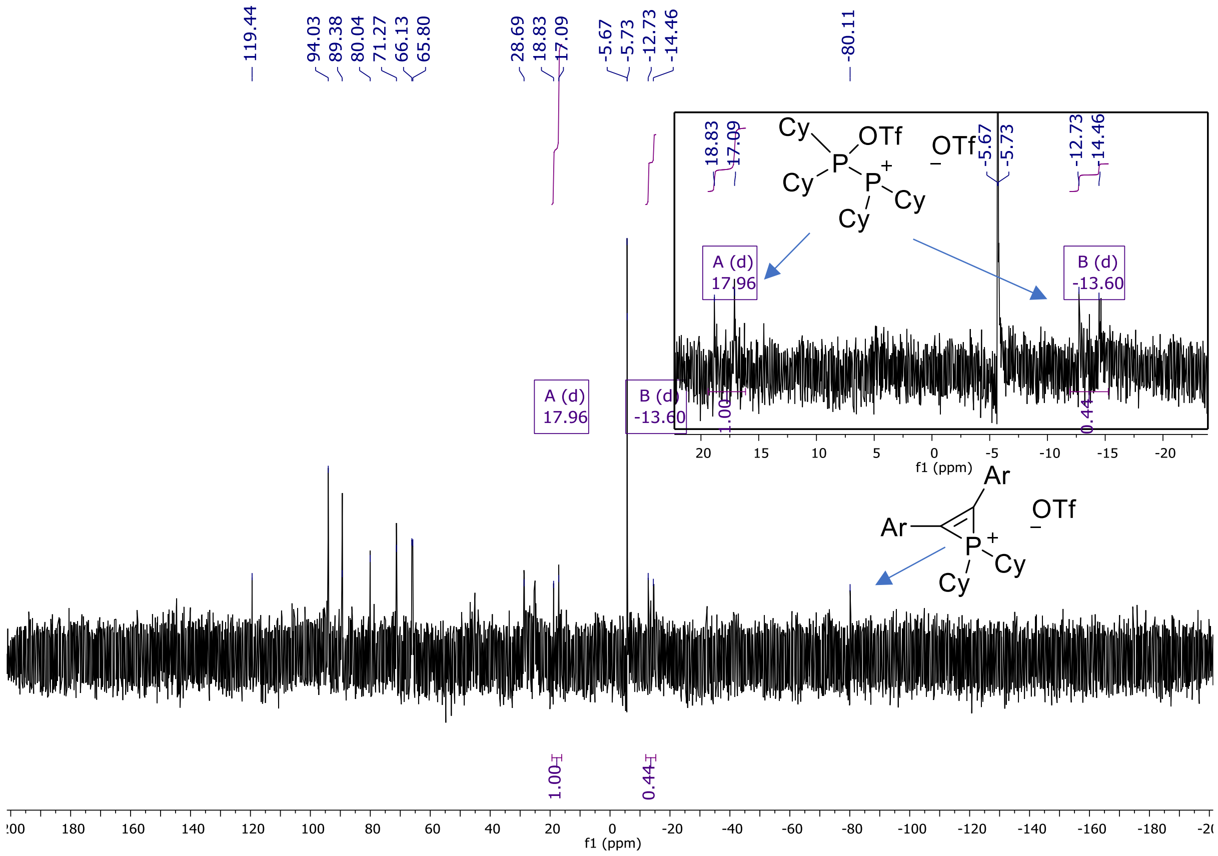

Figure S3 $^{31}P\{^{1}H\}$ inv gated NMR after 40 min at 40 °C

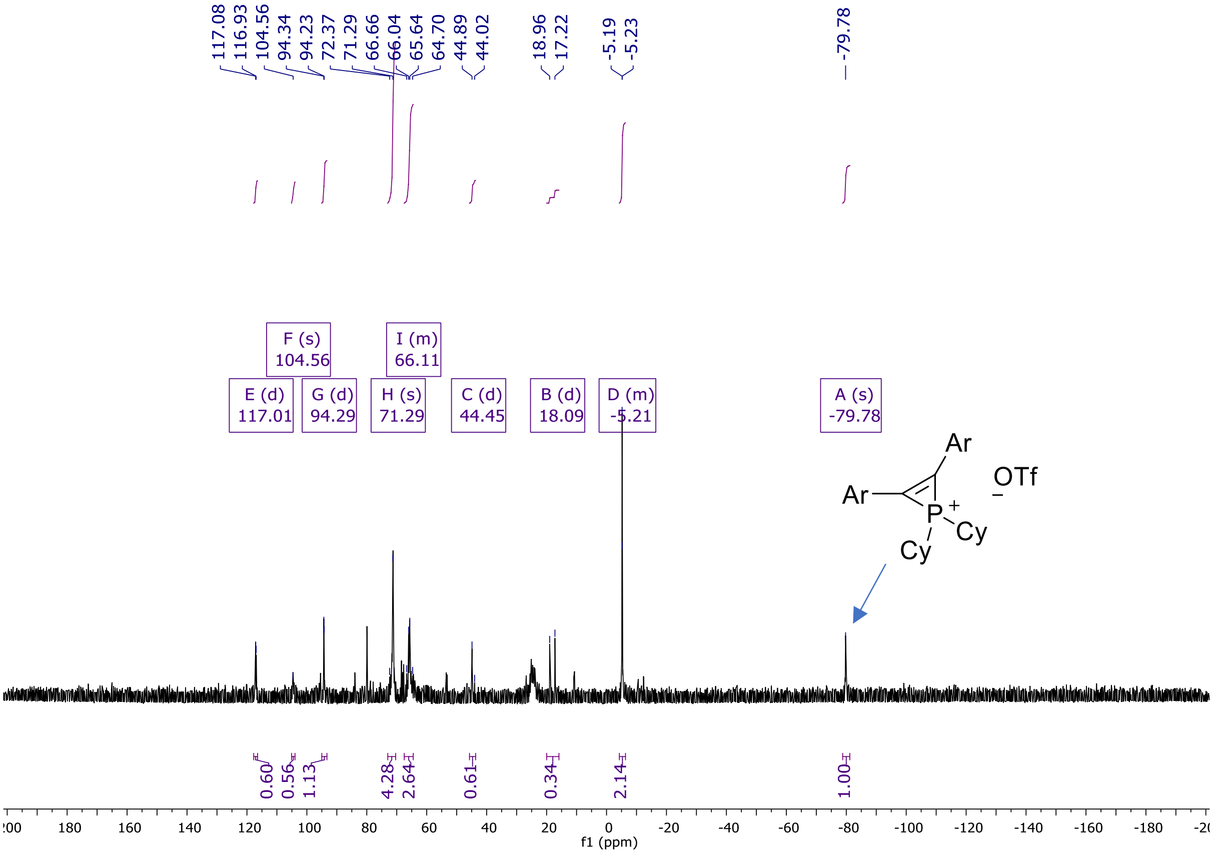

Figure S4 superimposed $^{31}P\{^{1}H\}$ inv gated NMR at r.t., 40 and 70 °C

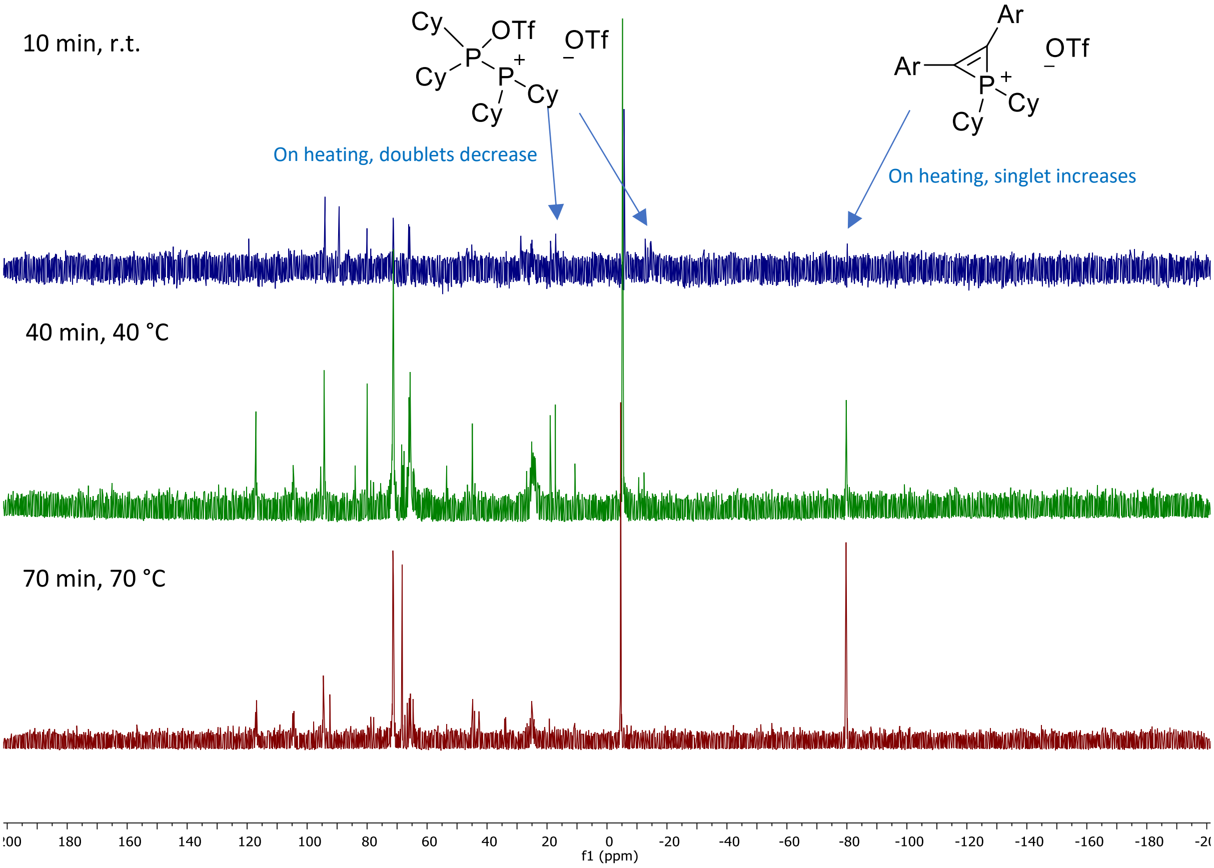

Figure S5 ^{19}F NMR at different temperatures compared to the alkyne (bottom NMR)

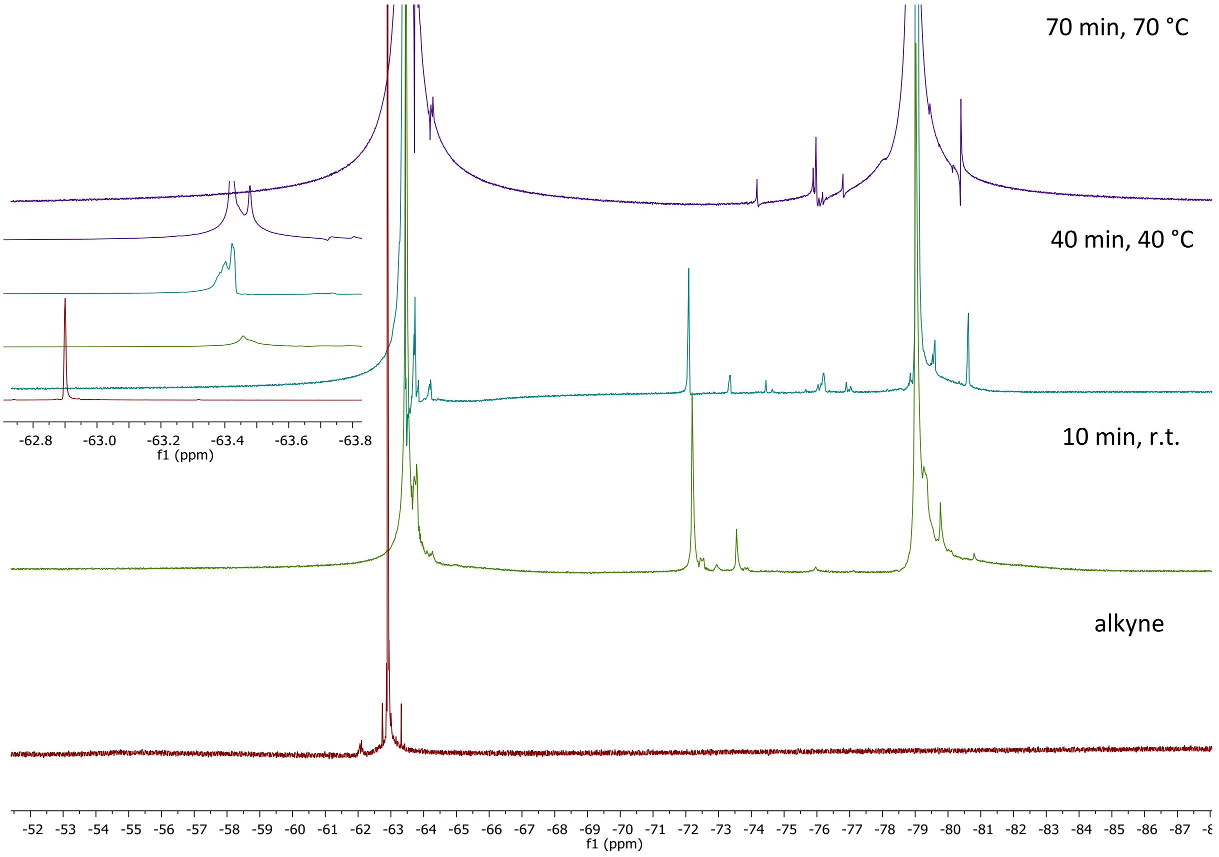

Mixing diphenyl phosphine oxide with triflic anhydride in a 1:1 ratio (0.1 mmol) in CD_2Cl_2 (0.6 mL) gives a mixture of products by $^{31}P\{^1H\}$ NMR (Figure S6, top); the singlet peak at δ 40.8 ppm has been identified as $Ph_2P(O)Cl$, while the AX-AB spin system 4.1 (dd, J = 476.4, 319.1 Hz) and −27.1 (d, J = 319.1 Hz) which has been reported as $[Ph_2P\text{-}P(H)Ph_2][OTf]$ **A•OTf**.[28]

The AB system at −18.1 (d, J = 368.5 Hz) and the singlet at 79.6 ppm have not been identified yet, however the 2 signals have been assigned to a potential $[Ph_2P(Cl)OP(H)Ph_2][2OTf]$ species.[29]

When using CD_3CN as solvent (Figure S6, bottom), a similar reaction mixture was observed. Formation of $Ph_2P(O)Cl$ could be due to traces amount of chlorinated residue coming from the synthesis of the SPO,[4] rather than halide abstraction from the solvent.

When analysing the ^{19}F NMR spectrum (Figure S7), other than full conversion of OTf_2 (−72 ppm), we observed other fluorine containing species (−30 and −52 ppm) which have not been fully characterised; we could not see traces of "P-OTf" bonds, which usually appears at >100ppm in the ^{31}P NMR, however the formation of these species cannot be excluded. Adding diphenyl acetylene to the reaction mixture, the phosphirenium species **3•OTf** was observed after 18 h at r.t. at −108.16 ppm. Formation of species **A•OTf** might help understand the reactivity of the phosphirenium species, particularly reinforcing the potential for a phosphenium species to form in solution, at least prior to cycloaddition.[25]

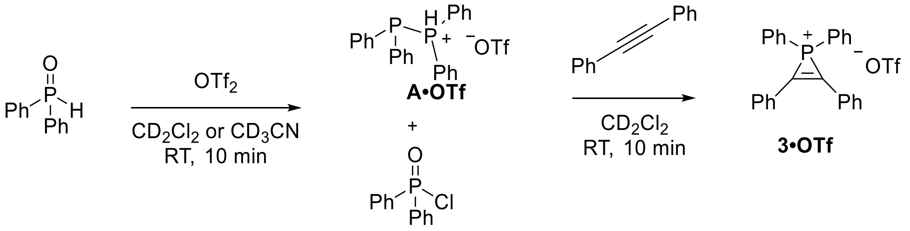

Figure S6 $^{31}P\{^1H\}$ NMR in CD_2Cl_2 (top) in CD_3CN (bottom)

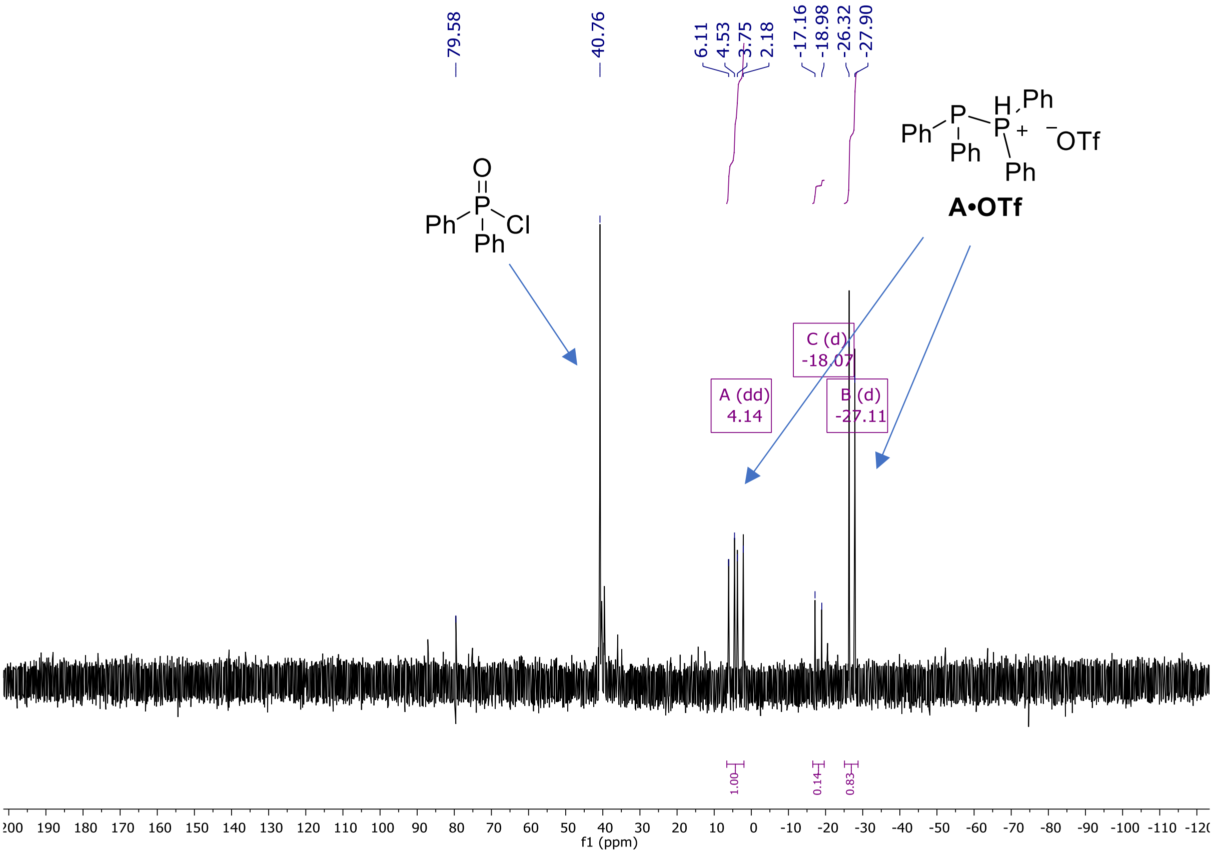

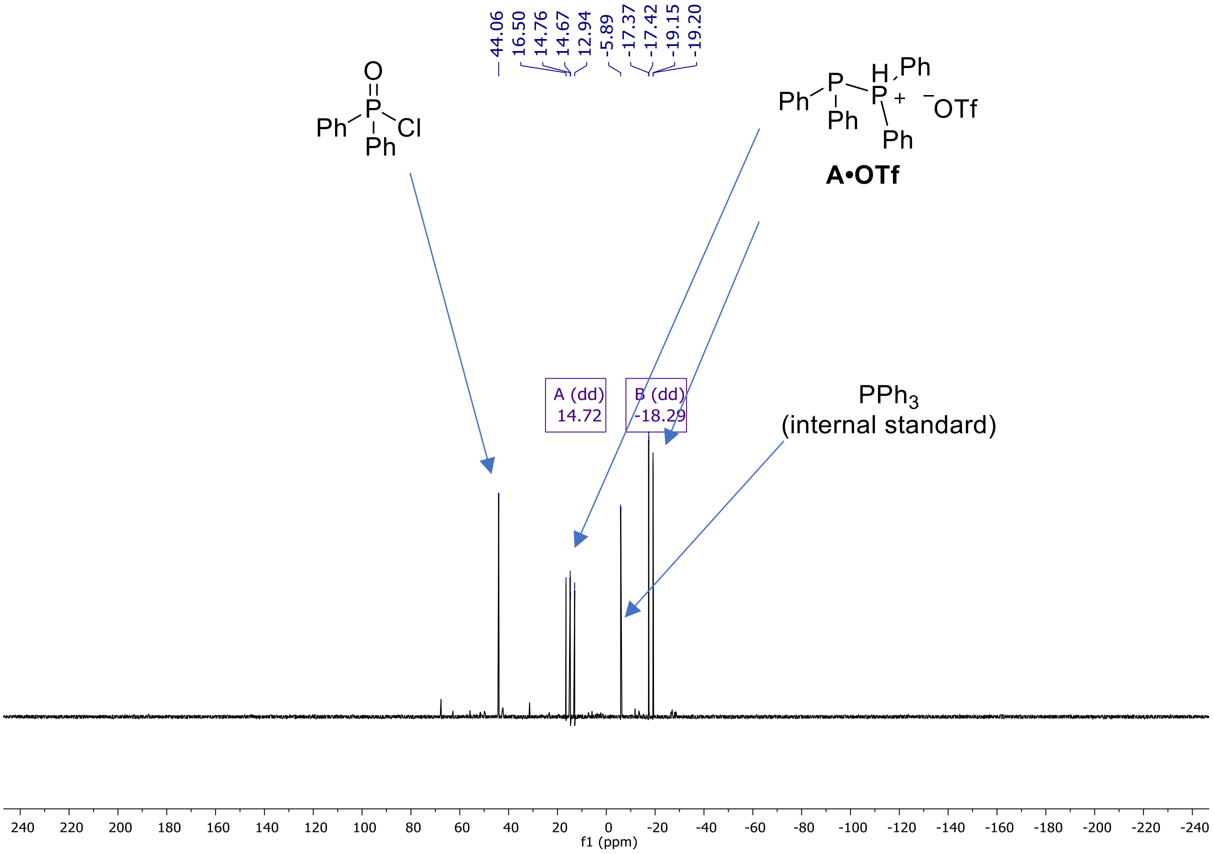

Figure S7 $^{19}F\{^1H\}$ NMR

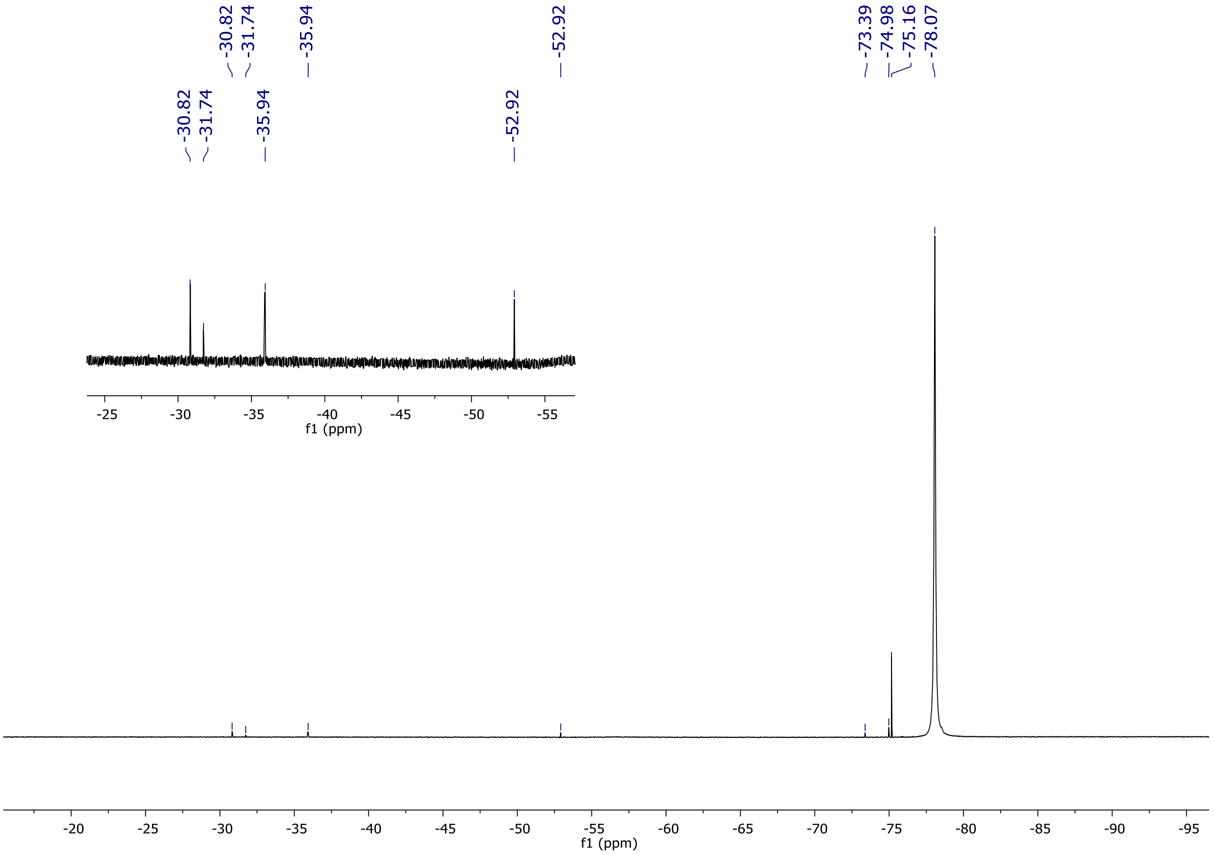

Mixing diphenyl phosphine oxide with triflic anhydride and NaB_{Ar}^{F} in a 1:1:1 ratio in $CDCl_3$ (0.16 M) a cleaner reaction mixture was observed by $^{31}P\{^{1}H\}$ NMR (Figure S8, top); of interest the AX-AB spin system at δ 7.0 (dd, *J* = 453.8, 334.1 Hz) and −24.9 (d, *J* = 331.5 Hz) assigned to $[Ph_2P\text{-}P(H)Ph_2][B_{Ar}^{F}]$ **A·B$_{Ar}^{F}$**. [28] The peaks at 143.3 and 113.4 ppm might be related to a form of phosphenium cation with a B_{Ar}^{F} counterion. [26-27]

After diphenylacetylene addition (Figure S9), and analysis at r.t., formation of the major product **3·B$_{Ar}^{F}$** was observed, with disappearance of **A·B$_{Ar}^{F}$**. It is worth noticing that the peak at 143.3 and 113.4 were not visible anymore, while peaks at 59.1 and 46.3 ppm increased.

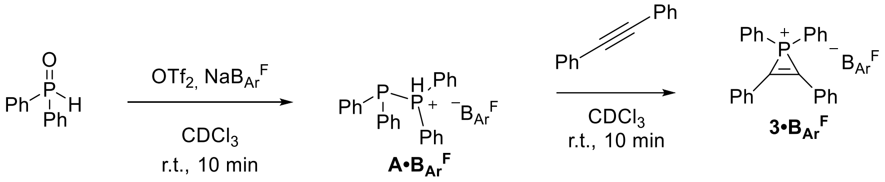

Figure S8 $^{31}P\{^{1}H\}$ NMR (top) and ^{19}F NMR (bottom) for the reaction mixture.

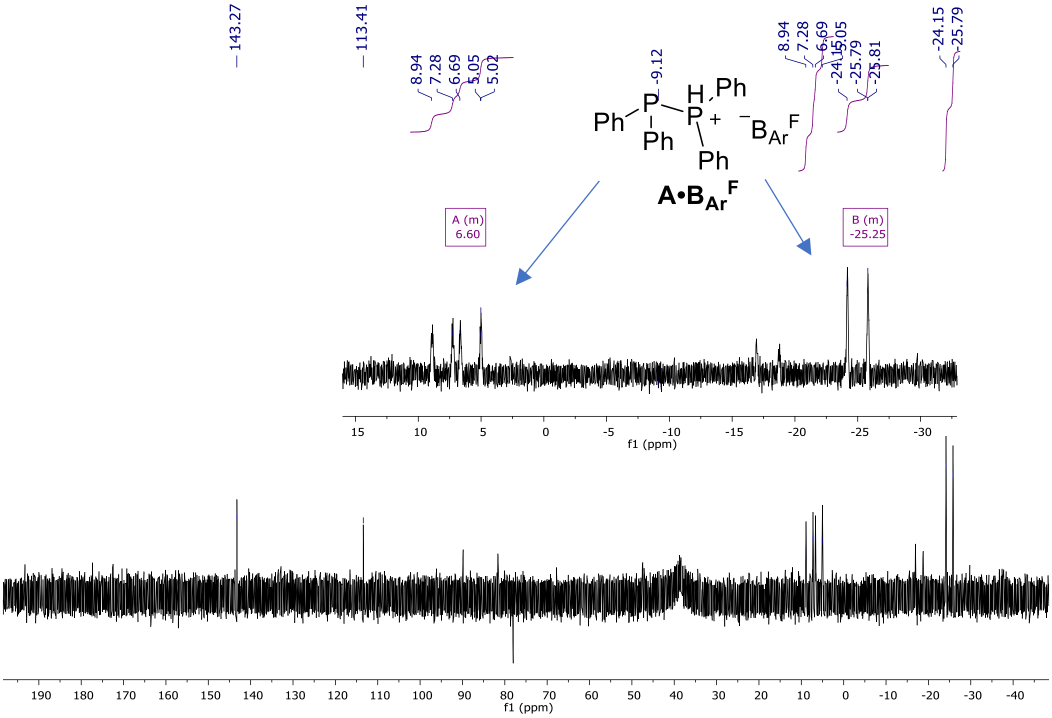

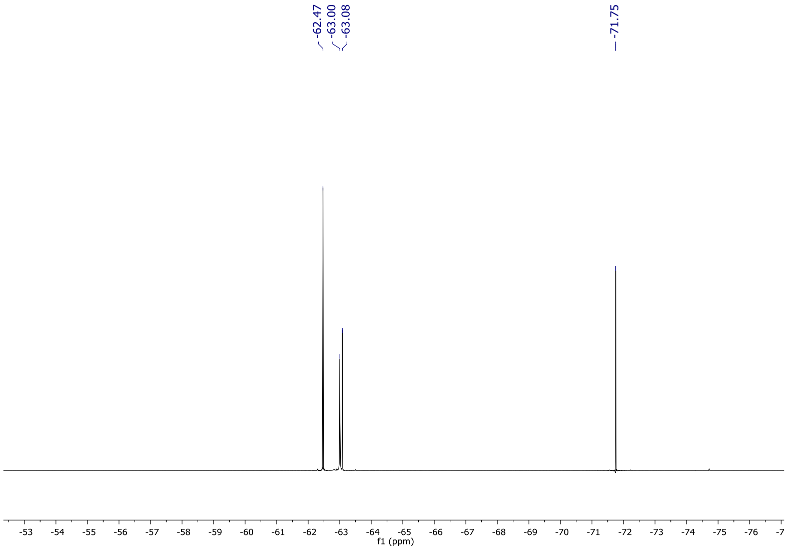

Figure S9 $^{31}P\{^1H\}$ NMR (top) and $^{19}F\{^1H\}$ NMR (bottom) for the reaction mixture after diphenylacetylene addition

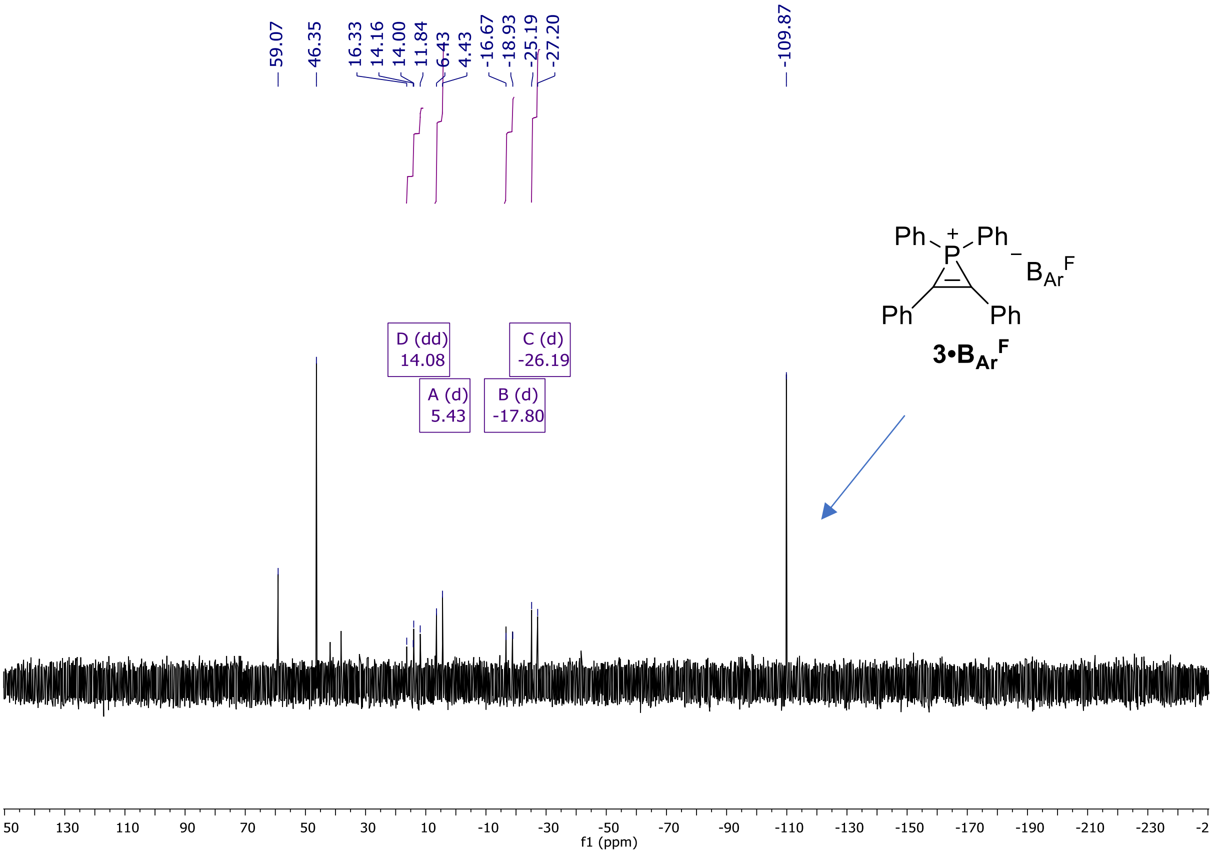

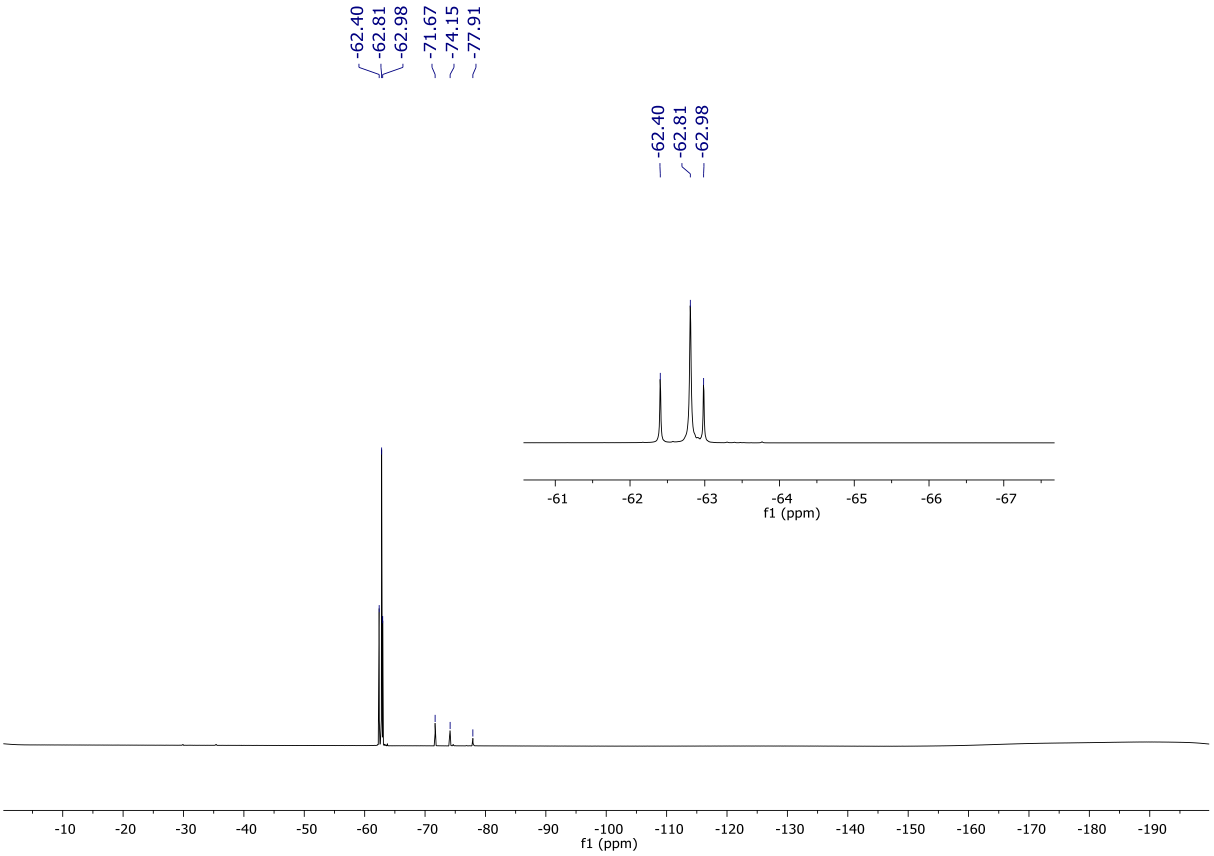

7.2 Stoichiometric studies

Figure S10 shows the outcome of *in situ* formation of **3·OTf** and onward reaction with dimethylphenyl silane **12** (0.2 mmol) at r.t. in CD_3CN (0.3 M). Is interesting to note that the phosphirenium ion does not interact directly with the silane. The major product identified by $^{31}P\{^{1}H\}$ NMR, is the peak at −108.6 ppm.

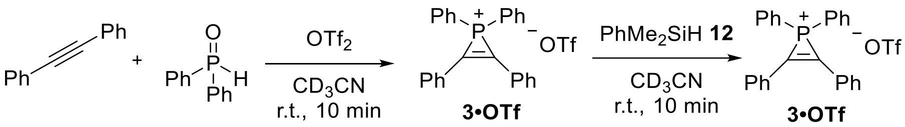

Figure S10 Inv gated $^{31}P\{^{1}H\}$ (top) and ^{1}H NMR (bottom) of the stoichiometric mixture of **3·OTf** with silane.

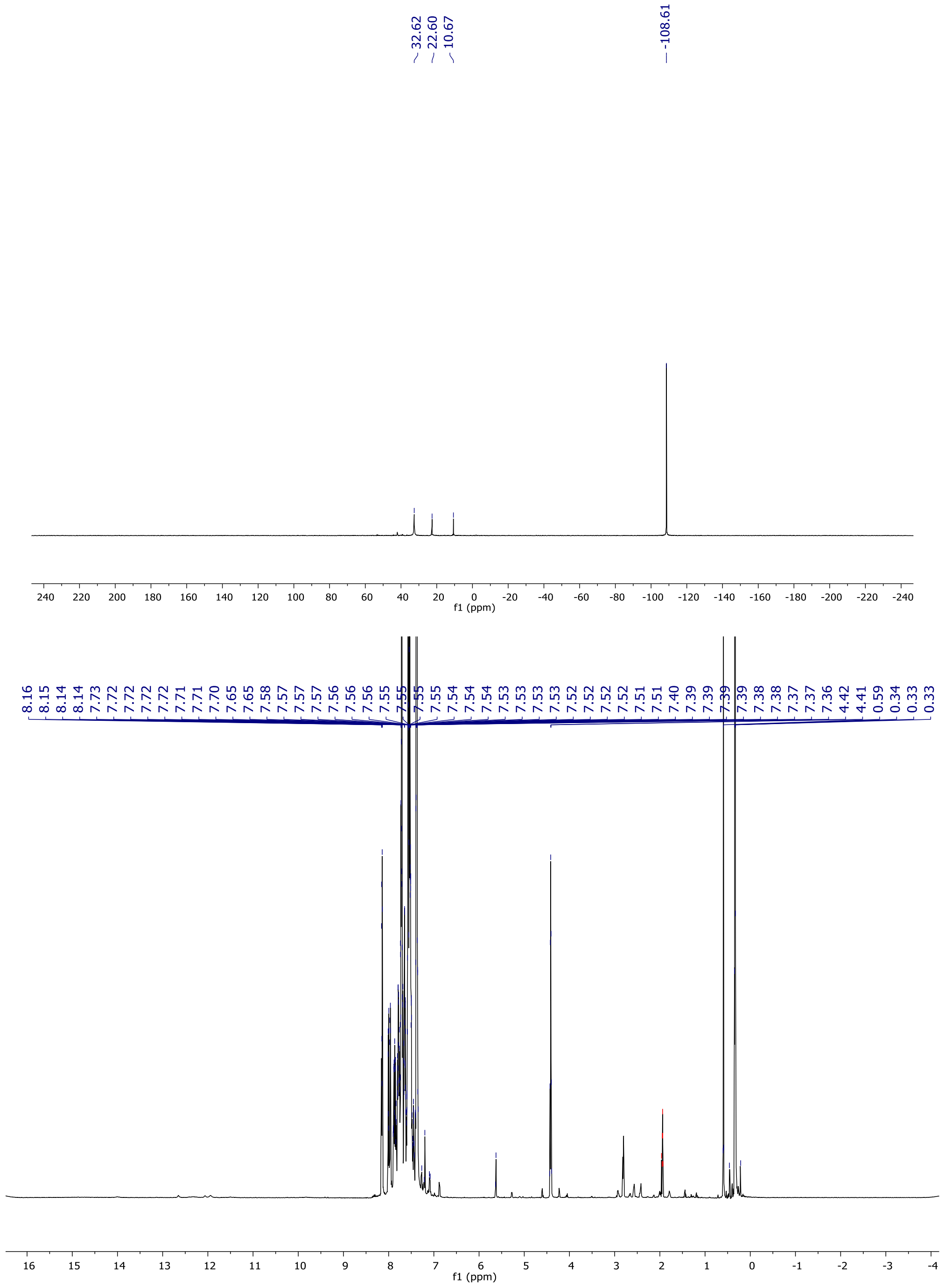

Figures S11 and S12 show the outcome of a stoichiometric reaction of *in situ* formed **3·B$_{Ar}$^{F}** with dimethylphenyl silane **12** (0.2 mmol) at r.t. in CD_3CN (0.3 M). Is interesting to note that there is immediate release of H_2 bubbles. The major products identified by inverse gated $^{31}P\{^1H\}$ NMR, which spectroscopic yield are calculated using PPh_3 as internal standard, are P_2Ph_4 at −17.6 ppm (11%), **3·B$_{Ar}$^{F}** at −108.6 ppm (66%) and Ph_2PH at −40.8 ppm (11%).

After 18 h at r.t., while **3·B$_{Ar}$^{F}** species persists, the silane is fully consumed and decomposition is observed both by $^{31}P\{^1H\}$ and ^1H NMR (Figure S13).

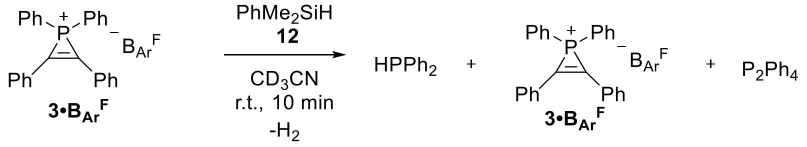

Figure S11 Inv gated $^{31}P\{^1H\}$ NMR of the stoichiometric mixture of **3·B$_{Ar}$^{F}** with silane **12** without 2,6-lutidine

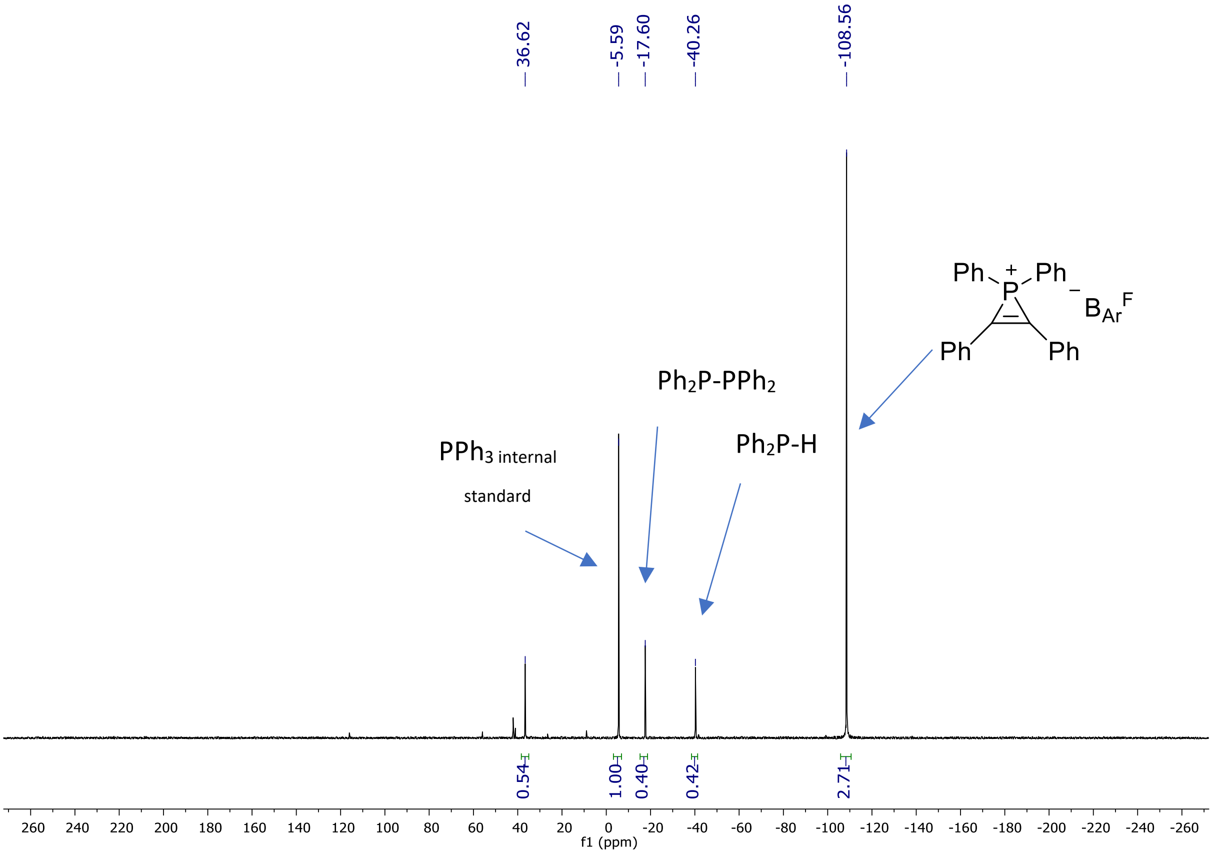

Figure S12 ^{1}H NMR of the stoichiometric mixture of **3·B_{Ar}^{F}** with silane **12** without 2,6-lutidine

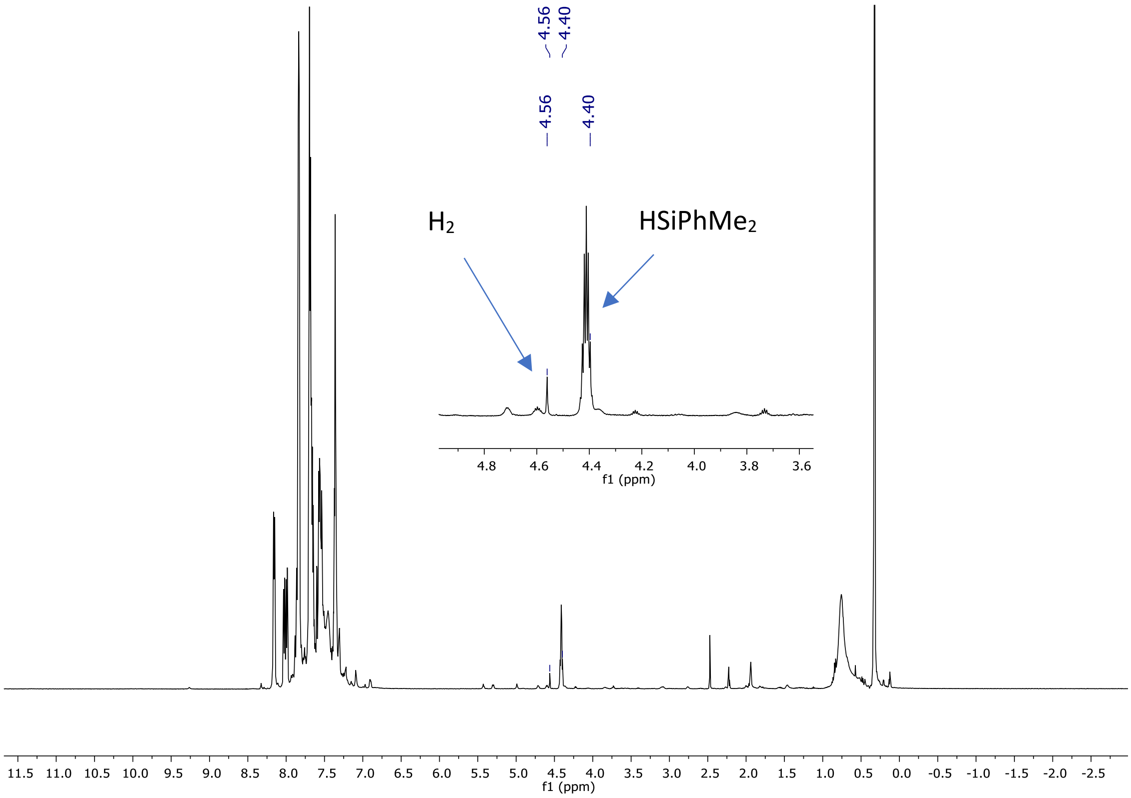

Figure S13 Inv gated $^{31}P\{^{1}H\}$ NMR of the stoichiometric mixture of **3·B_{Ar}^{F}** with silane **12** without 2,6-lutidine *after 18 h at r.t.*

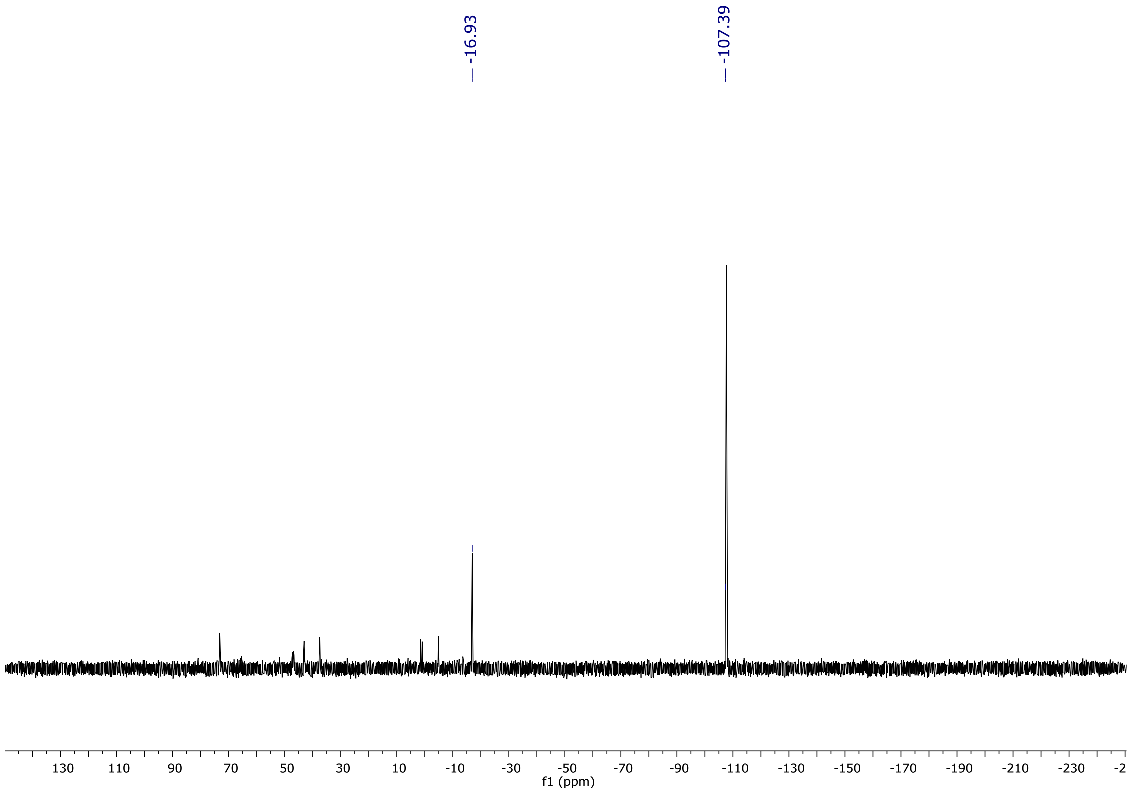

Figure S14 shows the outcome of a stoichiometric reaction of *in situ* formed **3·B$_{Ar}$^{F}** with 2,6 lutidine and dimethylphenyl silane **12** (0.15 mmol) at 80 °C for 18 h in CD_3CN (0.3 M), we observed formation of several phosphine containing; the major products identified and spectroscopic yield calculated using PPh_3 as internal standard are P_2Ph_4 at −17.6 ppm (36%), **3·B$_{Ar}$^{F}** at −108.6 ppm (46%) and Ph_2PH at −40.8 ppm (6%).

Three species are also observed by ^{1}H-^{29}Si HMQC (Figure S15); $PhMe_2SiH$ at −17.6 ppm, a peak at 23.4 ppm assigned as $Me_2HSiOTf$ formed by cleavage of the phenyl group,[30] and a peak at −0.3 ppm, which shows some long range interactions with the base, 2,6-lutidine.

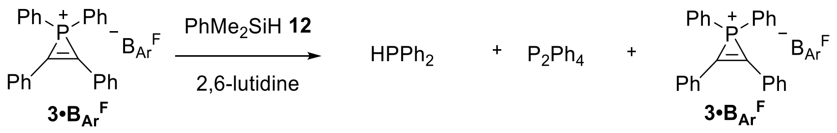

Figure S14 Inv gated ^{31}P{^{1}H} (top) and ^{1}H NMR (bottom) of the stoichiometric mixture of **3·B$_{Ar}$^{F}** with silane **12**

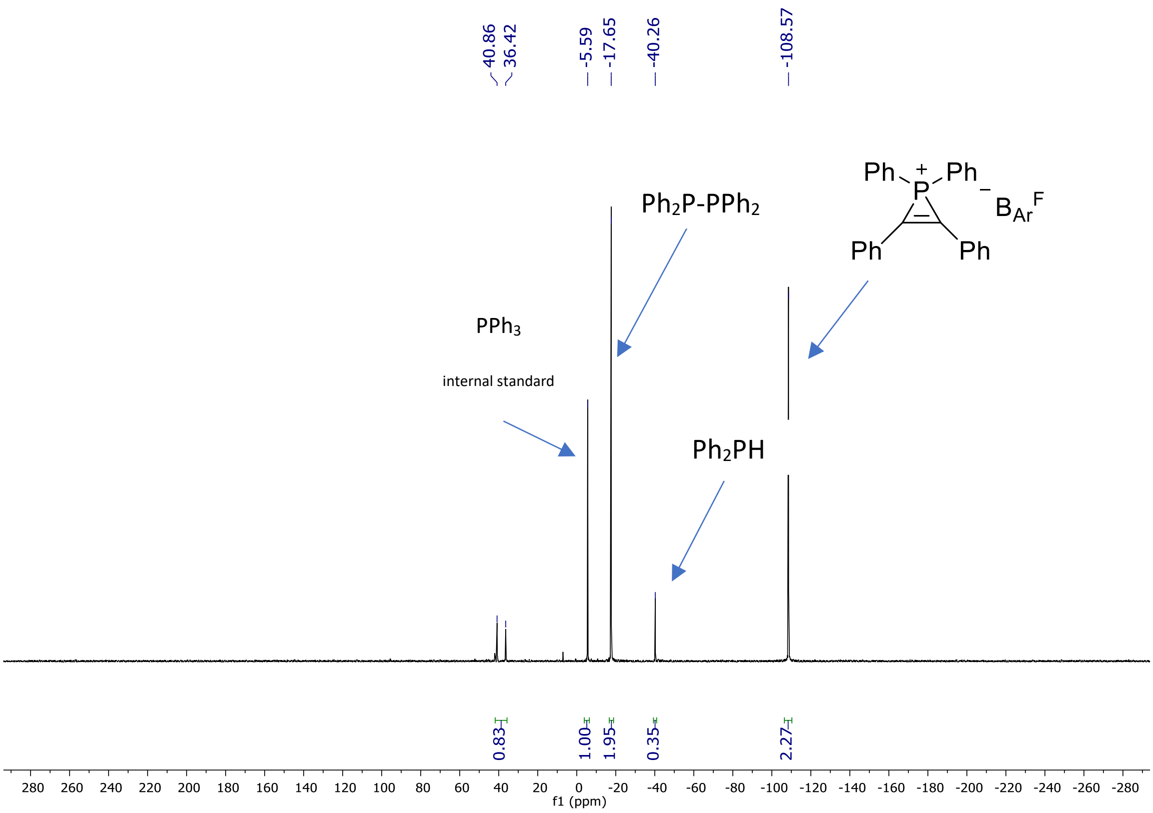

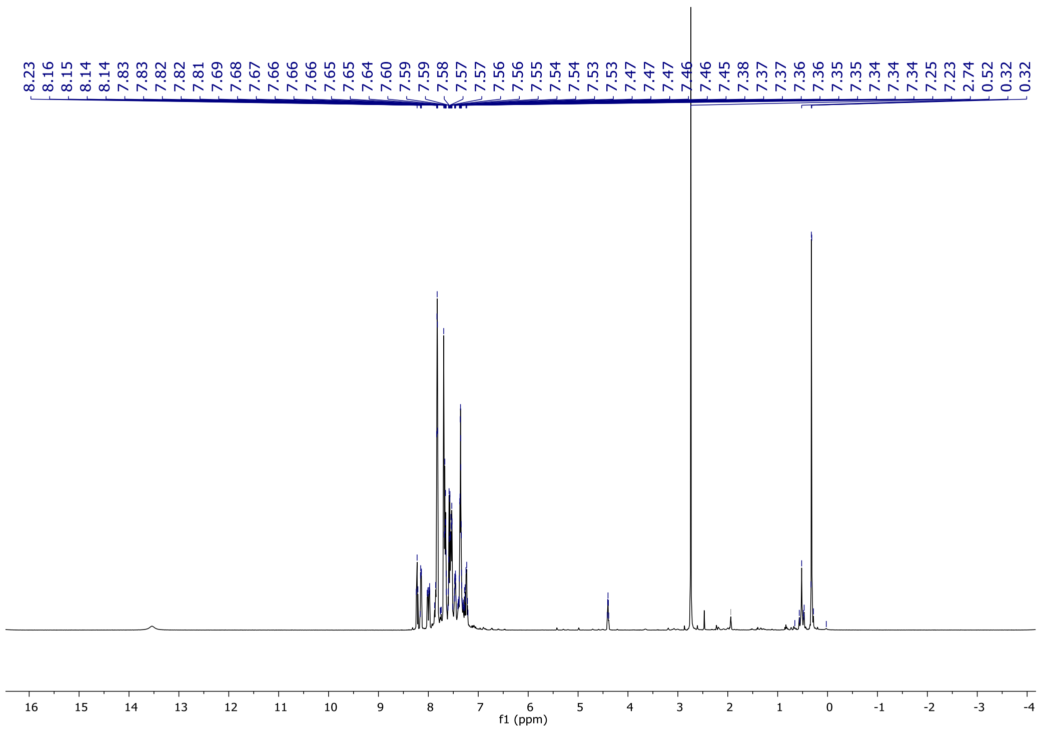

Figure S15 ^{1}H-^{29}Si HMQC of the stoichiometric mixture of **3·B$_{Ar}$^{F}** with silane **12**

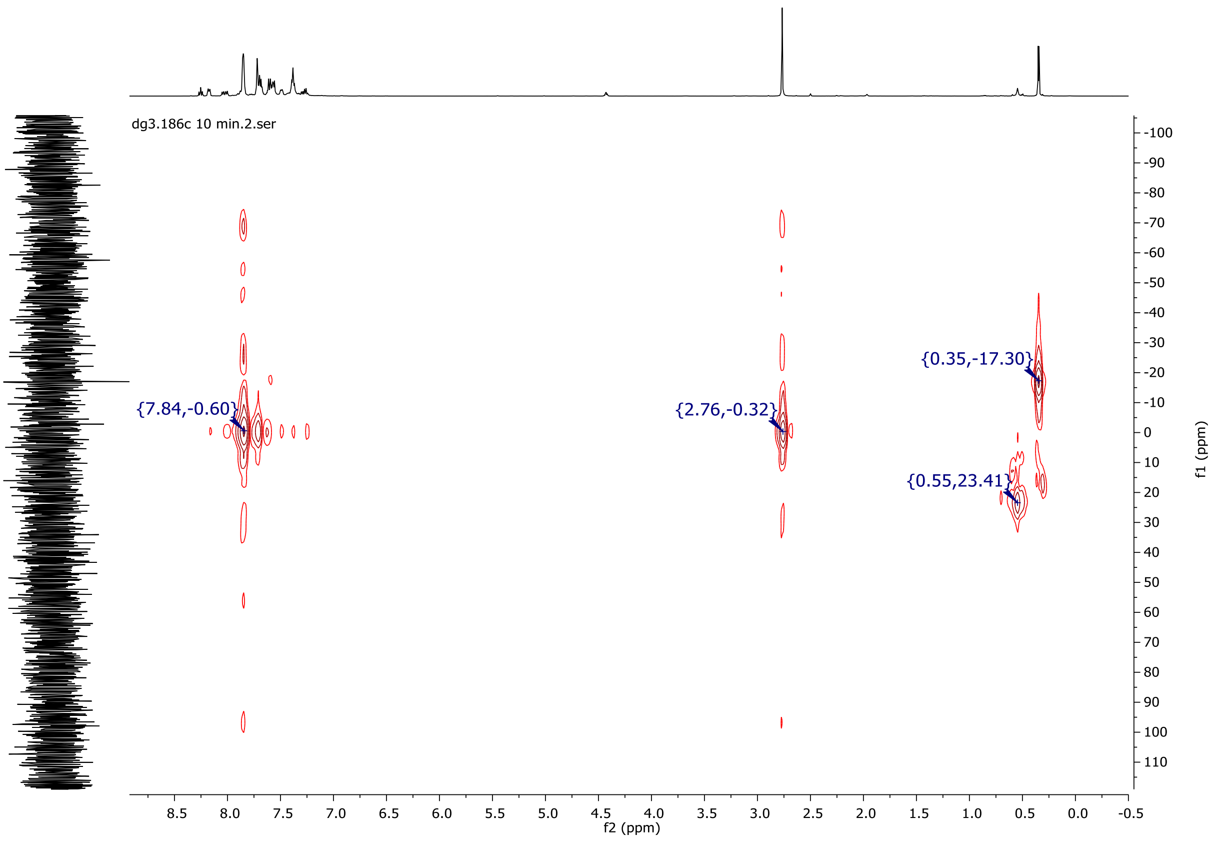

Figure S16b shows the outcome of a stoichiometric reaction of *in situ* formed **3·B$_{Ar}$^{F}** with 2,6 lutidine and D-dimethylphenyl silane **12** (0.15 mmol) at 80 °C for 2 to 18 h in CD_3CN (0.3 M). We observed formation of several phosphorus containing products, which match the spectrum observed when using $HSiMe_2Ph$ (Figure S16a); after 2 h the major products are P_2Ph_4 at −17.6 ppm, **3·B$_{Ar}$^{F}** at −108.6 ppm. Interestingly the incorporation of D was observed in the vinyl phosphine with a peak at 7.0 (t, *J* = 7.8 Hz) ppm, while no diphenyl phosphine was observed in this case, this could be due to a faster side-product formation when $DSiMe_2Ph$ is used.

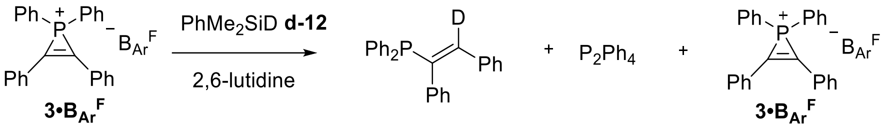

Figure S16 superimposed ^{31}P NMR experiment of the reaction with (a) $HSiMe_2Ph$ after 18 h, (b) with $DSiMe_2Ph$ after 2 h, (c) after 18 h at 80 °C

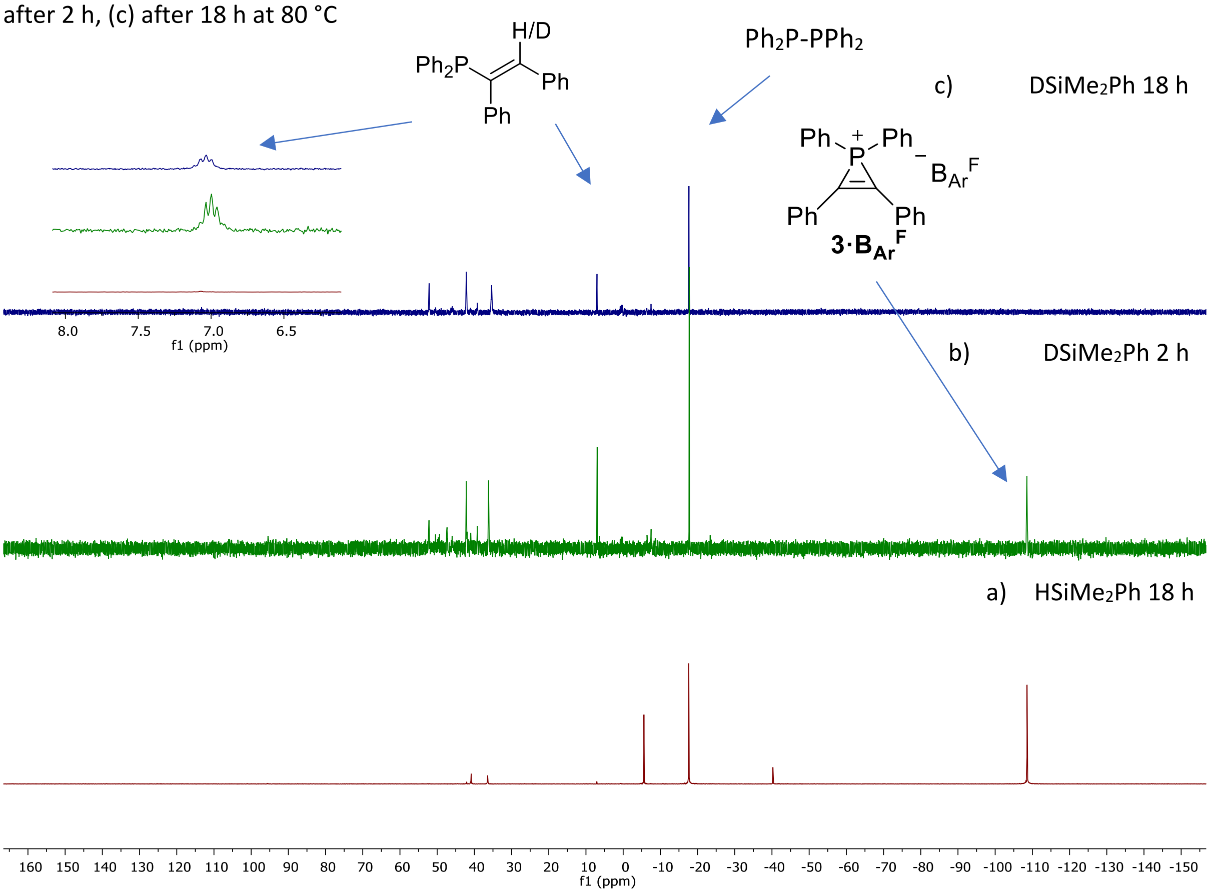

However, we also observed that deuterium exchange naturally occurs when mixing $DPPh_2$ (0.2 mmol, 90% D incorporation) with $HSiMe_2Ph$ (0.2 mmol) in CH_3CN (0.4 M) at 80 °C after 18 h (Figure S17). The experiment was performed in a J-Young NMR tube with a CD_3CN capillary. This phenomenon could potentially explain the absence of $DPPh_2$ in the experiment above.

Figure S17 ^{1}H and ^{31}P NMR of $DPPh_2$ and $HSiMe_2Ph$ in CH_3CN

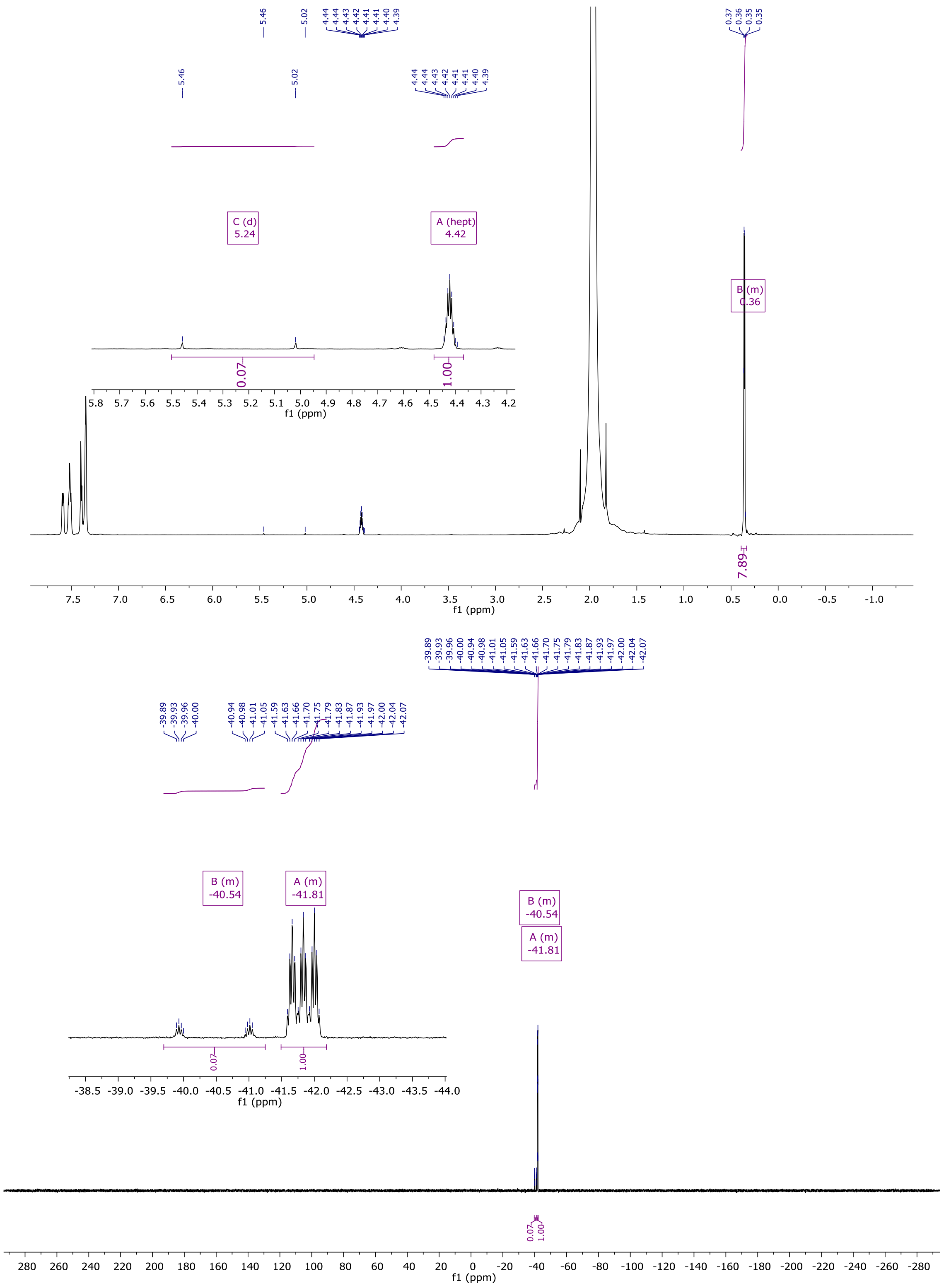

When adding benzophenone **11** (0.15 mmol) to the reaction mixture above and heating the reaction for 30 min at 80°C, the formation of 13a was observed in 37% spectroscopic yield (calculated with toluene as internal standard). The major species observed by inverse gated $^{31}P\{^1H\}$ NMR are the phosphirenium salt **3·B$_{Ar}$^{F}** at −108.5 ppm, vinylphosphine at 7.1, P_2Ph_4 at −17.6 ppm (Figure S18, top).

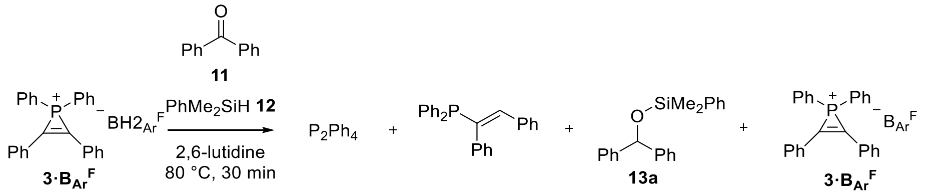

Figure S18 Inv gated $^{31}P\{^1H\}$ (top) and ^1H NMR (bottom) of the stoichiometric mixture of **3·B$_{Ar}$^{F}** with benzophenone **11** and silane **12**

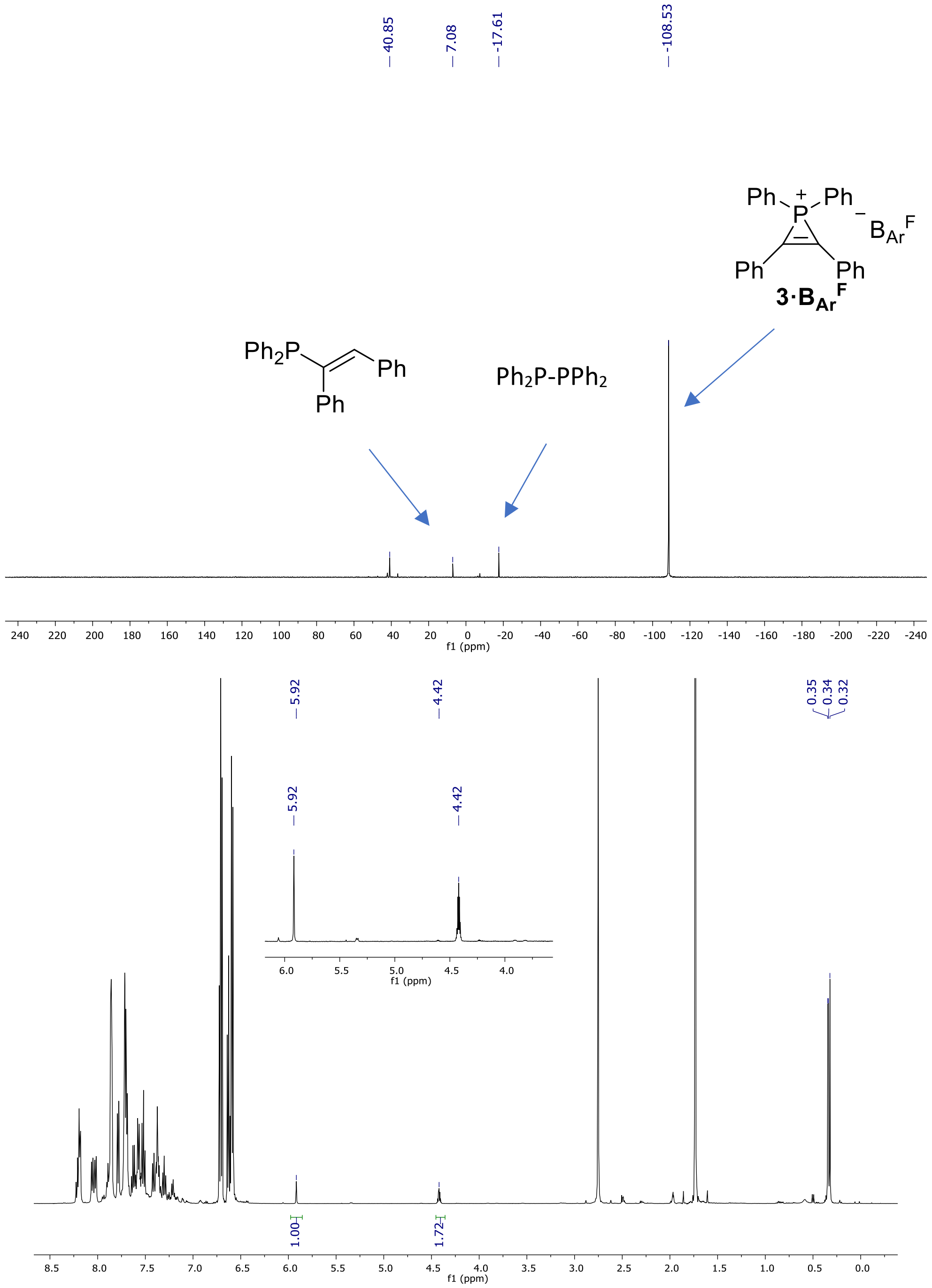

Reacting in a stoichiometric fashion **10·B$_{Ar}^F$** with 2,6 lutidine, benzophenone **11** and dimethylphenyl silane **12** (0.15 mmol) at 80 °C for 18 h in CD_3CN (0.3 M), we observed formation of product **13a** in 47% conversion, by ^1H NMR (Figure S19). This result highlights that **10·B$_{Ar}^F$** can effectively act as pre-catalyst for the hydrosilylation of ketone but with low conversion compared to the more active **3·B$_{Ar}^F$** and **9·B$_{Ar}^F$**. Several phosphine containing products are observed by $^{31}P\{^1H\}$ NMR which are difficult to interpret (Figure S20). The major products identified are P_2Ph_4 at −17 ppm, **10·B$_{Ar}^F$** at −110 ppm and $Ph_2P(O)Cl$ at 40.8 ppm. By comparison with literature reports,[31] the peak at 7.1 ppm could be related to vinyl phosphine species.

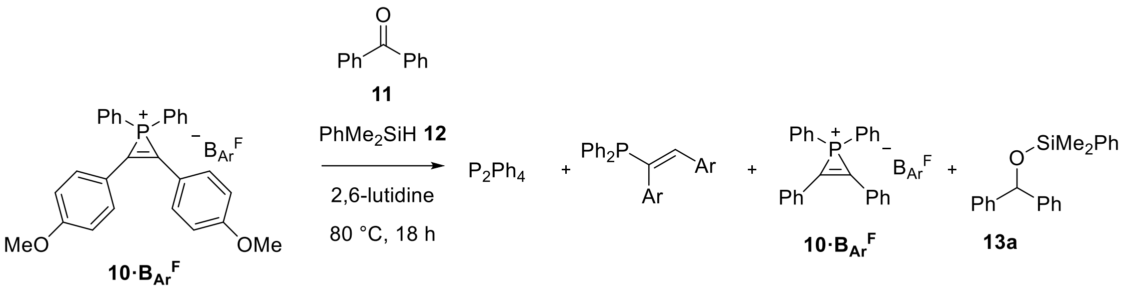

Figure S19 ^1H NMR for the stoichiometric mixture of **10·B$_{Ar}^F$** with benzophenone **11** and silane **12**

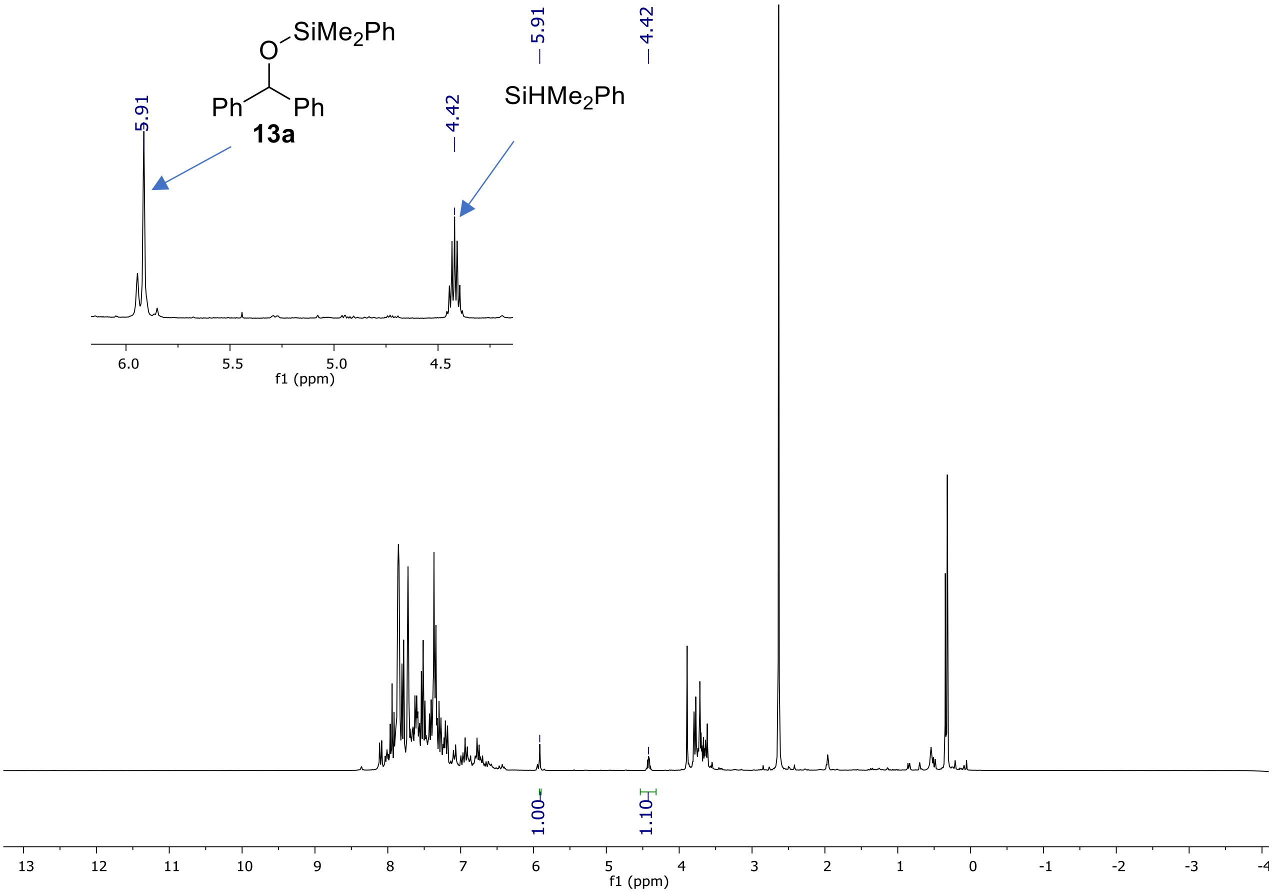

Figure S20 $^{31}P\{^1H\}$ NMR for the stoichiometric mixture of **10·B$_{Ar}$^{F}** with benzophenone **11** and silane **12**

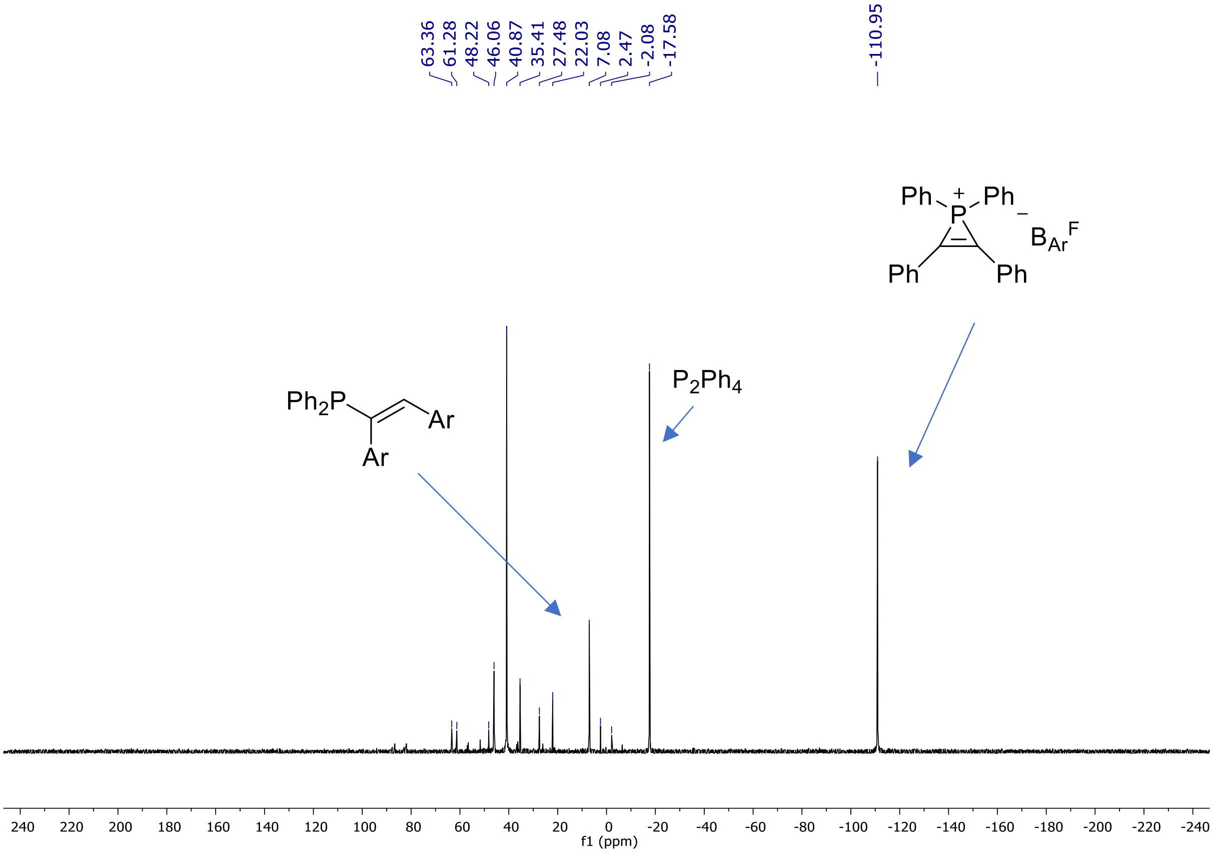

7.3 Phosphirenium cation in catalysis

Evidence of persistence of phosphirenium in the reaction was found when analysing the hydrosilylation reaction between benzophenone **11** (0.2 mmol) and dimethylphenyl silane **12** (0.2 mmol) with 10 mol% of **4·B$_{Ar}$^{F}** to product **13a**. An aliquot was taken from the reaction mixture after 2 h reaction at 80 °C; the aliquot was dried and analysed by ^{1}H (Figure S21) and ^{31}P{^{1}H} NMR. The presence of the phosphirenium cation is confirmed as major species visible in the ^{31}P{^{1}H} NMR (Figure S22).

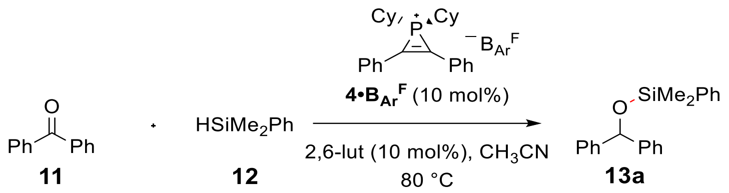

Figure S21^{1}H NMR at t$_0$ and after 2 h reaction

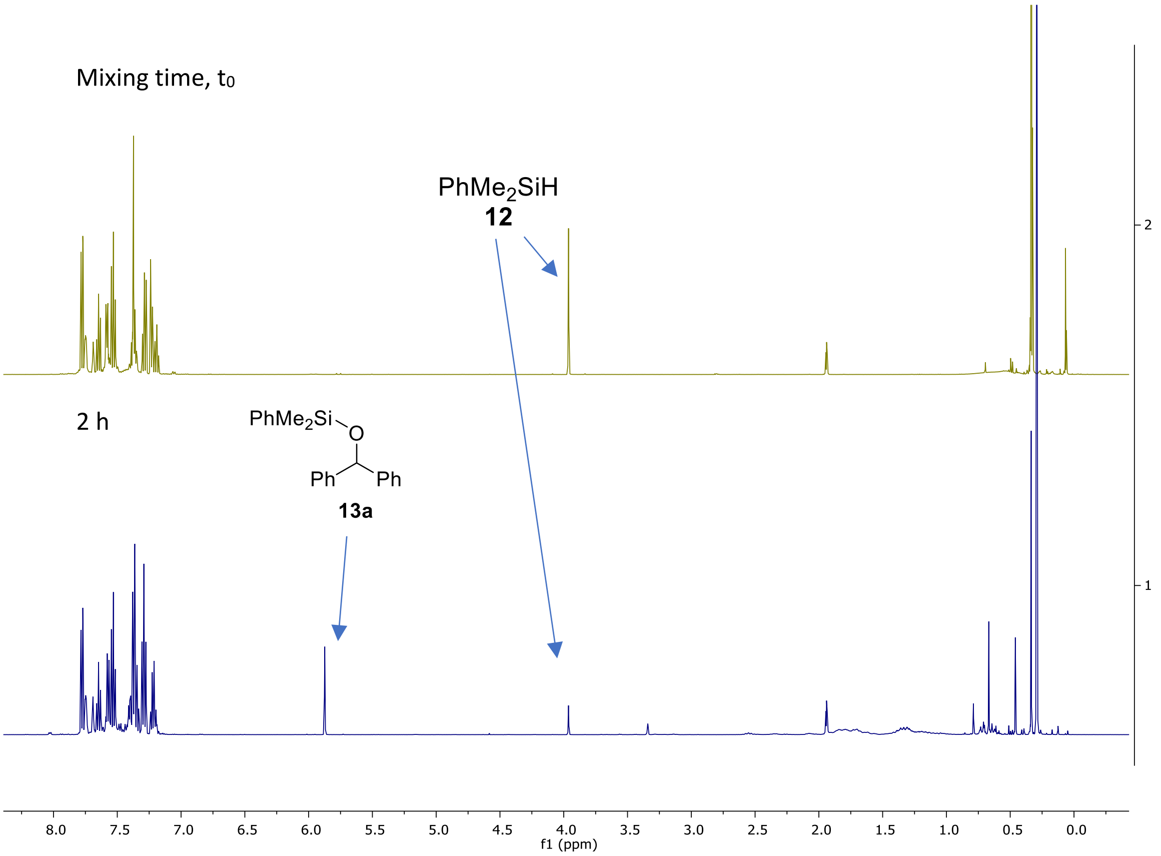

Figure S22 $^{31}P\{^{1}H\}$ NMR after 2 h reaction

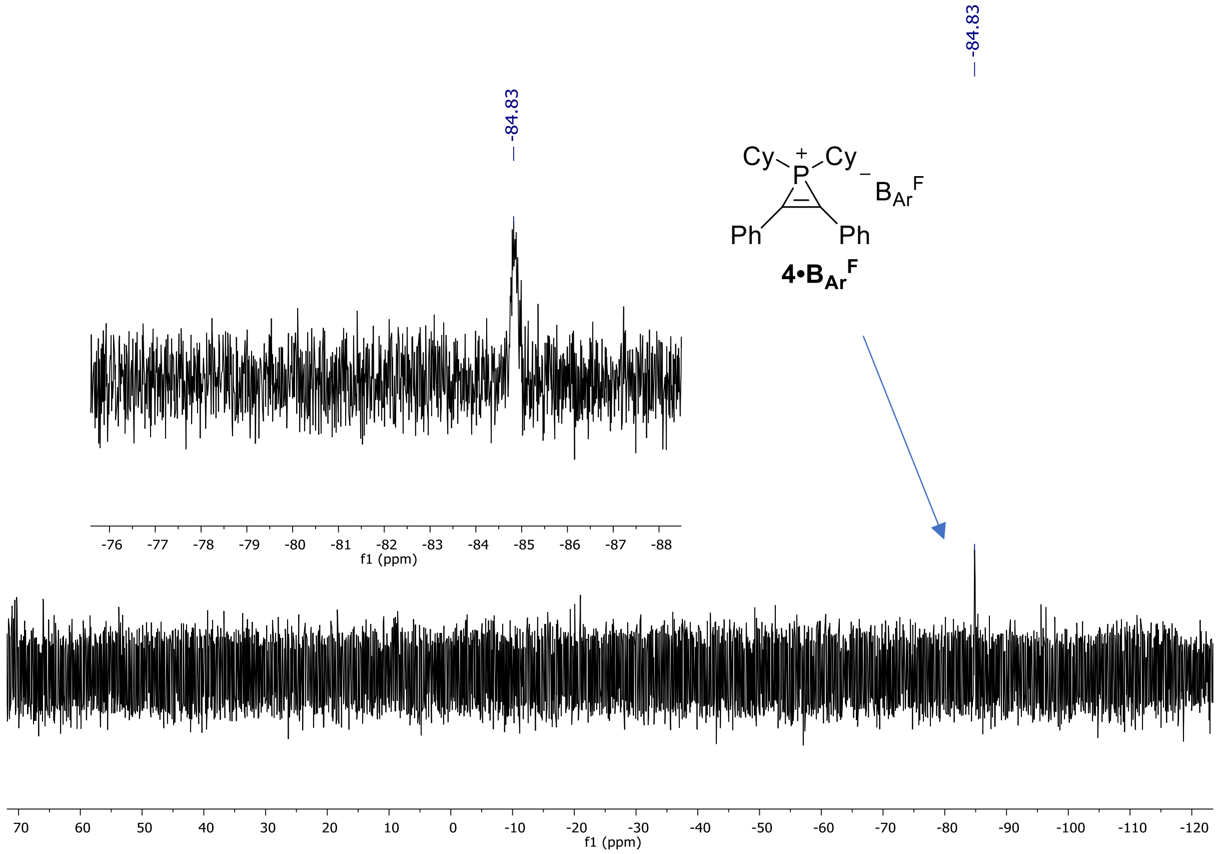

The catalytic hydrosilylation of benzophenone **11** and dimethylphenylsilane **12** (0.2 mmol) was performed with **3·B$_{Ar}$^{F}** (10 mol%) and 2,6-lutidine (10 mol%) in CD_3CN (0.4 M) at 80 °C. *In situ* spectroscopy analysis were performed at ~60% conversion of **13a**. Multiple species are observed by $^{31}P\{^{1}H\}$ NMR the major ones being the phosphirenium species **3·B$_{Ar}$^{F}** at −108.5 ppm, (*E*) and (*Z*)-vinyl phosphine (2:1) at 7.1 and −7.4 ppm and P_2Ph_4 at −17.5 ppm (Figure S23, bottom). We tentatively assign the peak at 36 ppm as the benzhydryl diphenyl phosphinate.[32] The species at 52 ppm has not been identified yet.

The (*E*)-vinyl phosphine C-H alkene is visible also by ^{1}H NMR at 6.49 (*J* = 8 Hz), Figure S23, top.

Analysis of the $^{11}B\{^{1}H\}$ NMR shows only one peak, at −6.6 ppm (Figure S24, top), which excludes boranes formation thus Lewis base catalysis.

The latter result is also supported by the ^{19}F NMR spectrum (Figure S24, bottom) which also shows one peak at −63.1 ppm corresponding to the $B_{Ar}{}^F$ counterion, and a peak at −79.1 ppm which was confirmed to be NaOTf when adding fresh NaOTf to the solution. It is worth reminding that NaOTf is not active for this transformation, see Table S2.

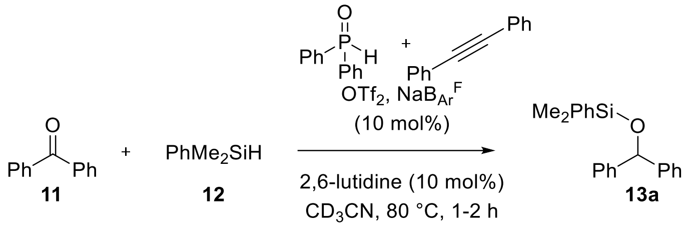

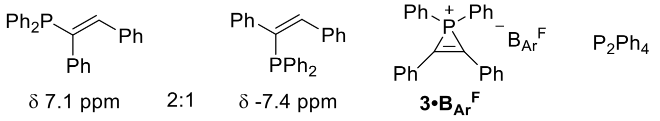

Figure S23 ^{1}H (top) and $^{31}P\{^{1}H\}$ NMR for the catalytic hydrosilylation of benzophenone **11** with silane **12**

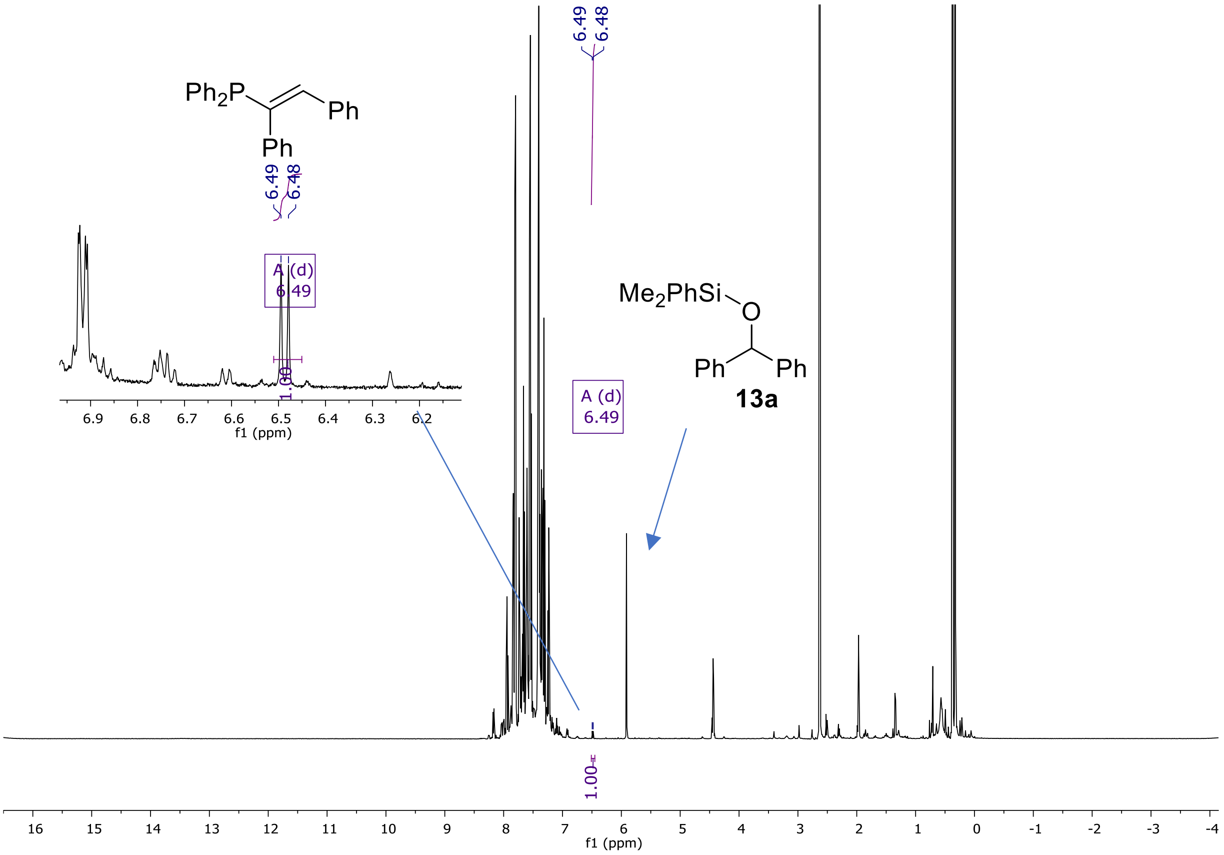

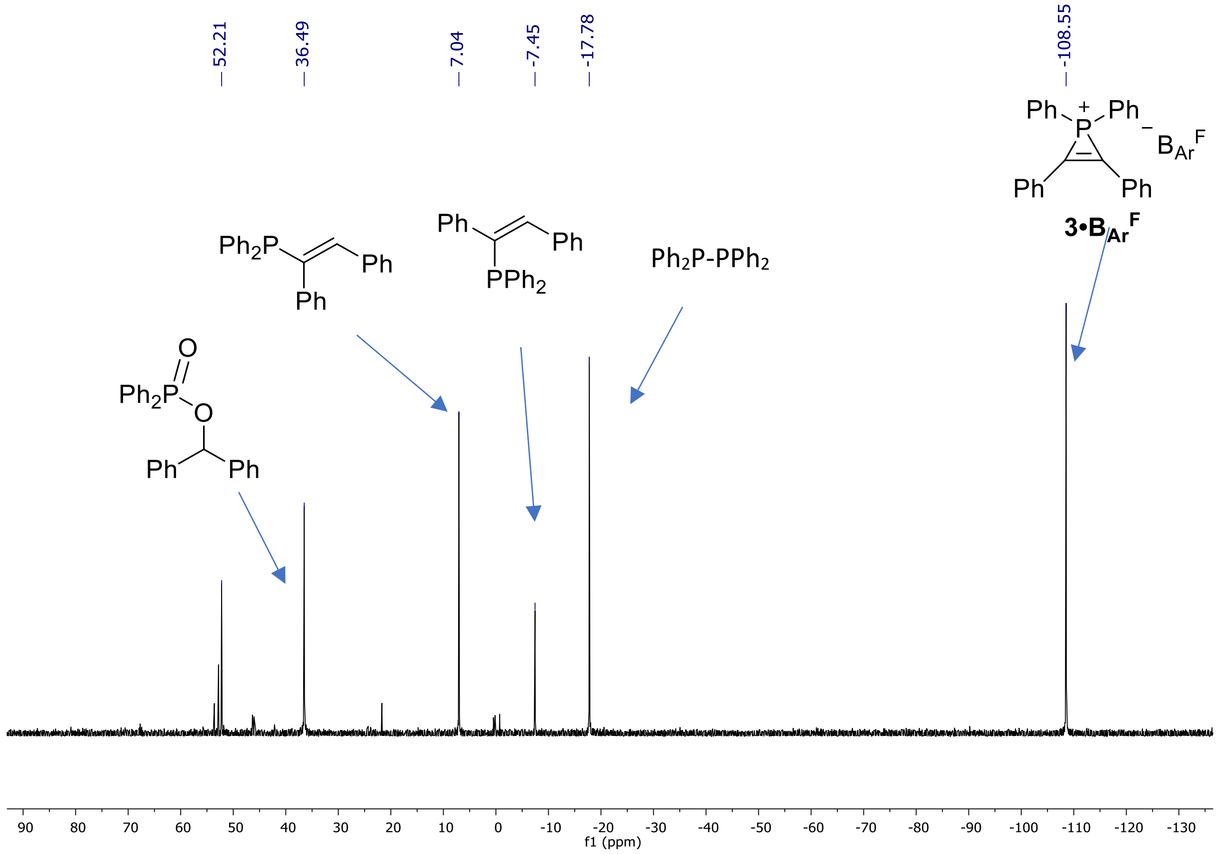

52.21
36.49
7.04
-7.45
-17.78
-108.55
Ph2P-PPh2
3•BArF
90
80
70
60
50
40
30
20
10
0
-10
-20
-30
-40
-50
-60
-70
-80
-90
-100
-110
-120
-130
f1 (ppm)

Figure S24 ^{11}B (top) and ^{19}F (bottom) NMR for the catalytic hydrosilylation of benzophenone **11** with silane **12**

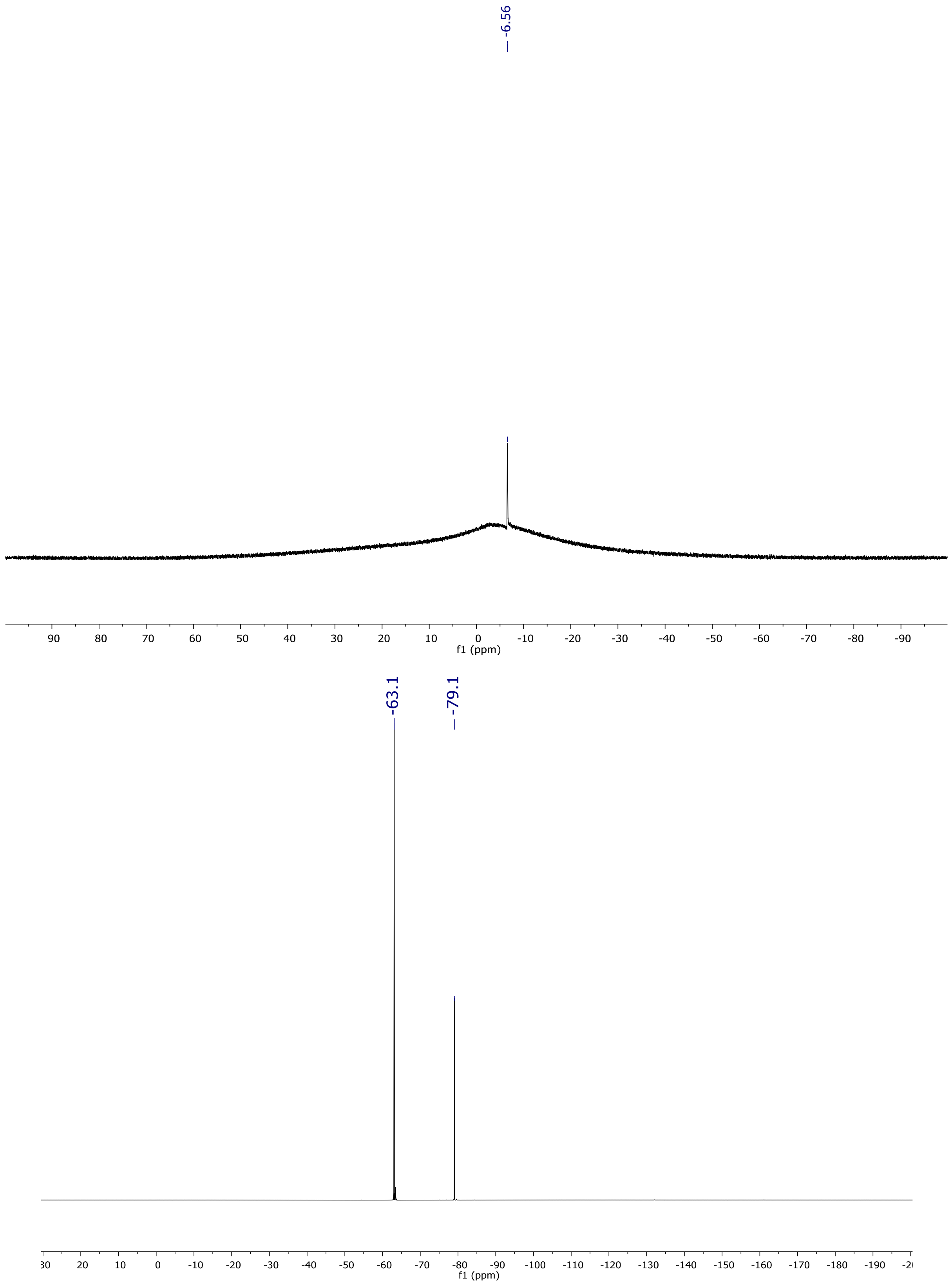

The catalytic hydrosilylation of benzophenone **11** and dimethylphenylsilane **12** (0.2 mmol) was performed with **9·B$_{Ar}$^{F}** (10 mol%) and 2,6-lutidine (10 mol%) in CD_3CN (0.4 M) at 80 °C. After 1h reaction and full conversion into product the reaction was opened to air and filtered through a short silica plug to yield the product and the (*E*)-vinyl phosphine which was characterised *in situ* (81% spectroscopic yield of vinyl phosphine isolated as phosphine oxide).

^{1}H NMR (500 MHz, $CDCl_3$) δ 7.81 – 7.69 (m, 1H), 7.64 – 7.50 (m, 1H), 7.50 – 7.43 (m, 1H), 7.43 – 7.25 (m, 2H), traces of **13a** and 2,6-lutidine. *See* Figure S25.

^{19}F NMR (470 MHz, $CDCl_3$) δ −62.4. *See* Figure S26, top.

^{31}P{^{1}H} NMR (122 MHz, $CDCl_3$) δ 22.4, −17.1 (P_2Ph_4). *See* Figure S26, bottom.

HRMS (ESI): calcd for $C_{28}H_{19}F_6OP^+$ [M-H]$^-$: 515.1078, found: 515.1025.

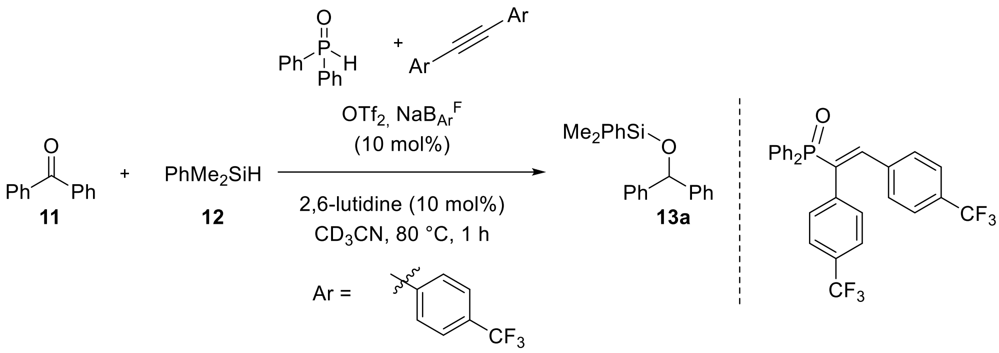

Figure S25 ^{1}H NMR of the quenched reaction mixture with **9·B$_{Ar}$^{F}**.

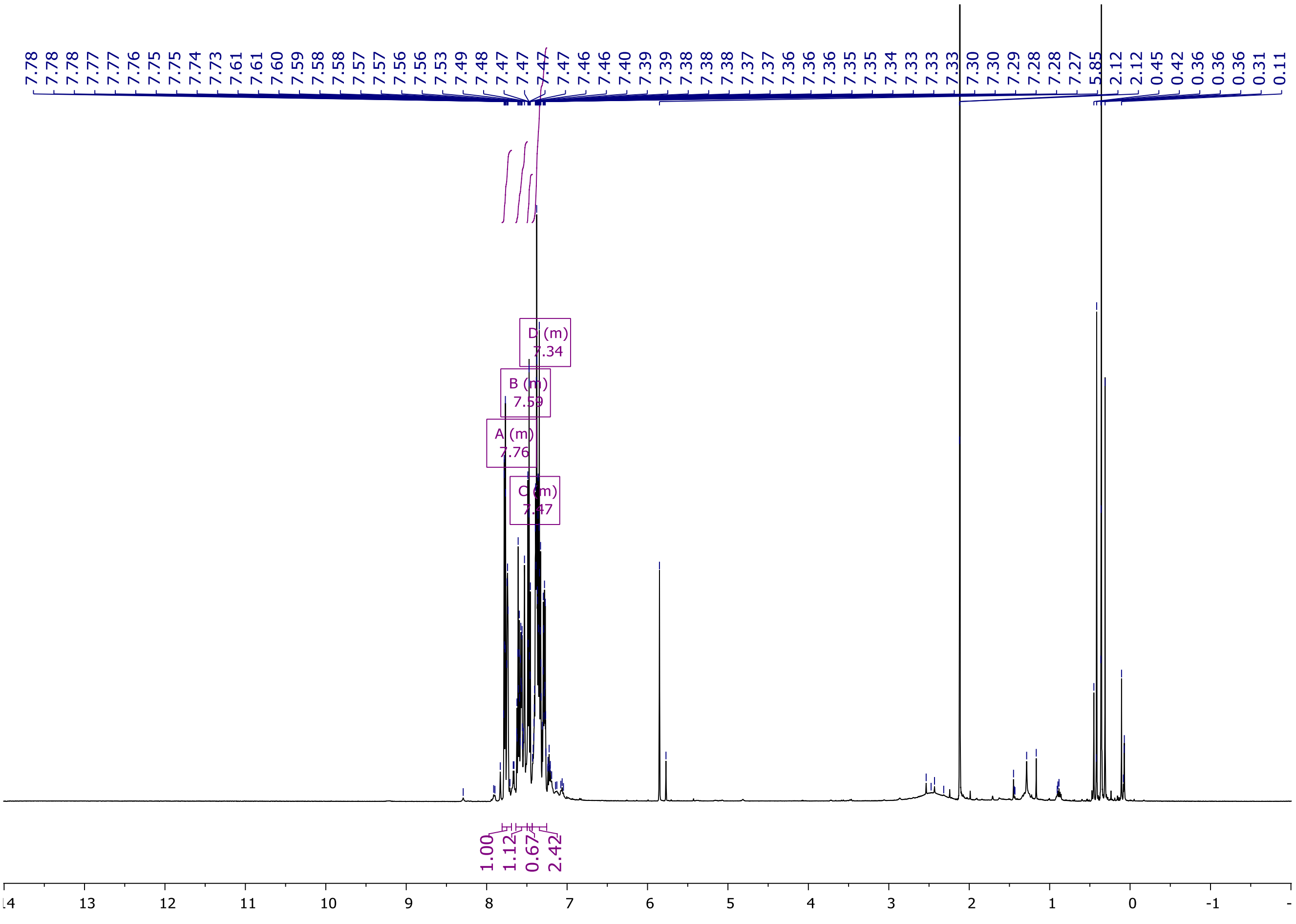

Figure S26 ^{19}F and $^{31}P\{^{1}H\}$ NMR of the quenched reaction mixture with **9·B$_{Ar}$^{F}**.

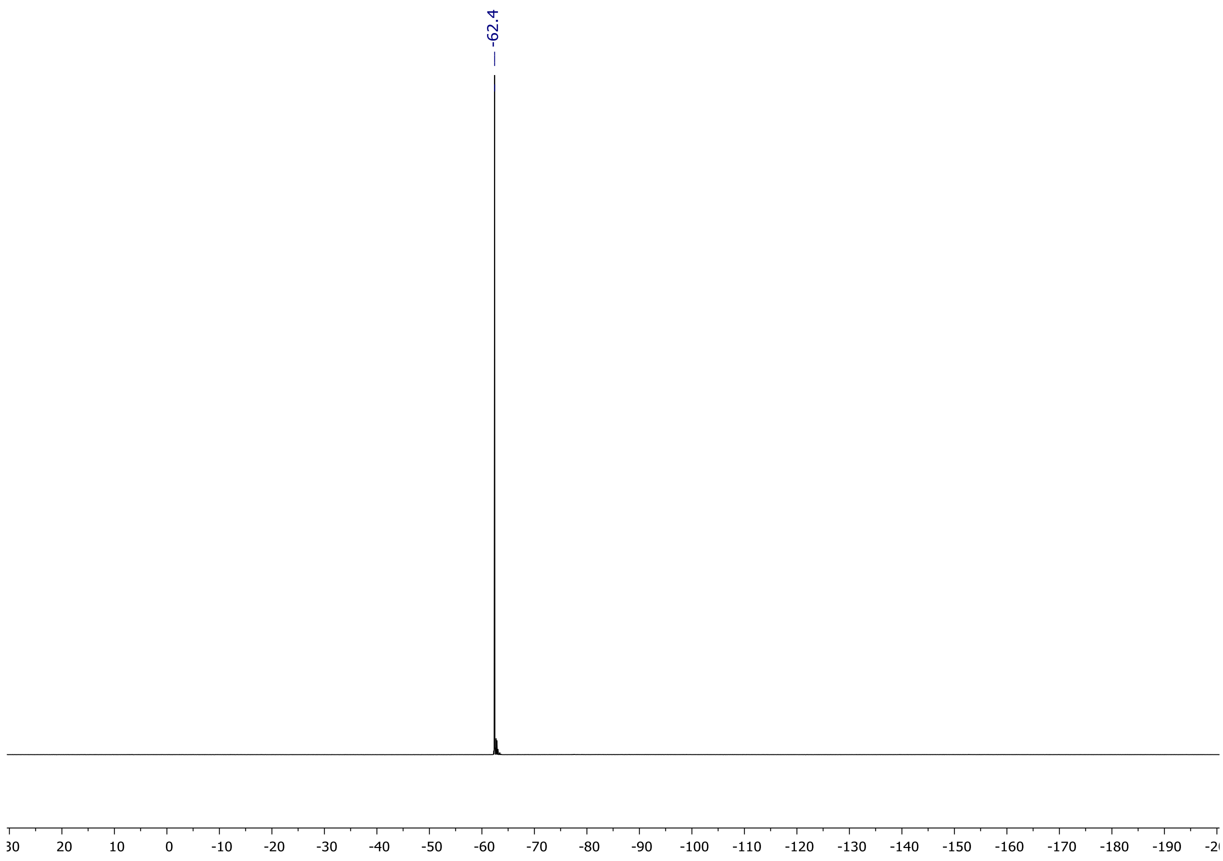

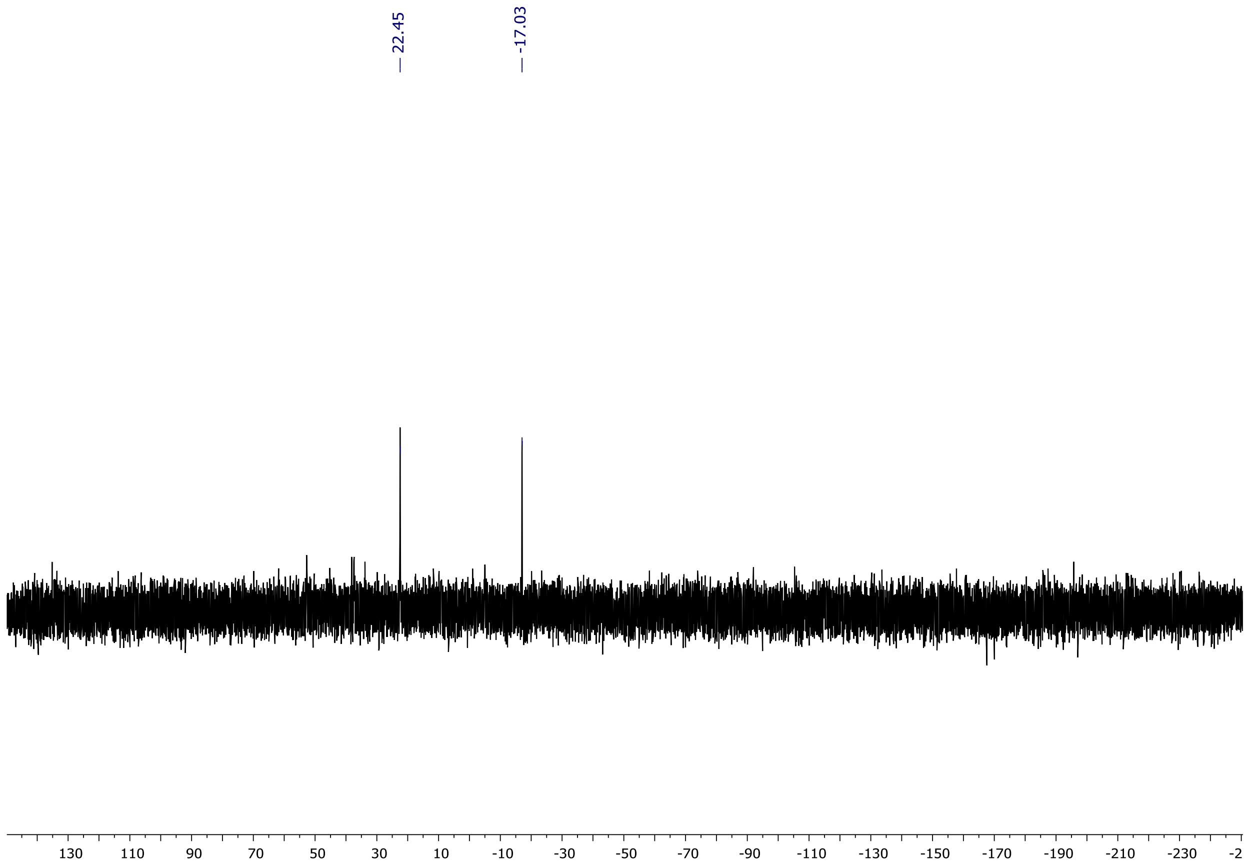

7.4 Reaction kinetics

Plots in Figure S27 to S30 show the reaction profiles for the following study: kinetic runs were carried out using variable amounts of benzophenone **11** (0.75 to 2 equiv.) and dimethylphenyl silane **12** (0.2 mmol) with 10 mol% catalyst loadings prepared *in situ* and 2,6-lutidine (10 mol%) in CD_3CN (0.4 M). The reaction was monitored by ^1H NMR heating the spectrometer up to 350K in presence of toluene as internal standard. In an effort to decrease the catalyst decomposition, as described in the previous section, the reaction was also performed with 14 equiv. of **11** to **12** (0.2 mmol) with **3·B$_{Ar}$^{F}** (10 mol%) formed *in situ* and 2,6-lutidine (10 mol%) in CD_3CN (0.4 M) at 80 °C. However, when performing the analysis of the reaction mixture at 78% conversion of **13a**, multiple species are observed by $^{31}P\{^1H\}$ NMR as observed for the catalytic reaction at 1:1 ratio, which shows that an increased amount of ketone still does not prevent side-reaction.

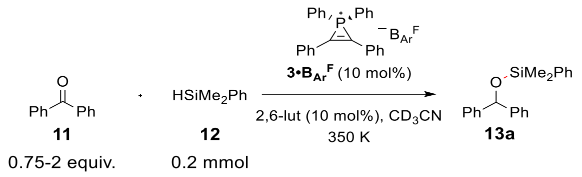

(a) conv (mmol/dm³) *vs* time (min) 0.75 equiv.

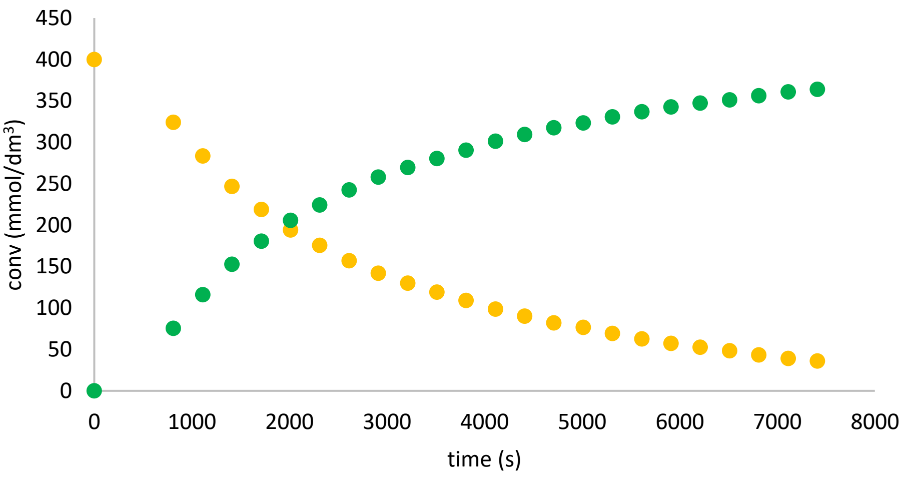

(b) conv (mmol/dm³) *vs* time (min) 1 equiv.

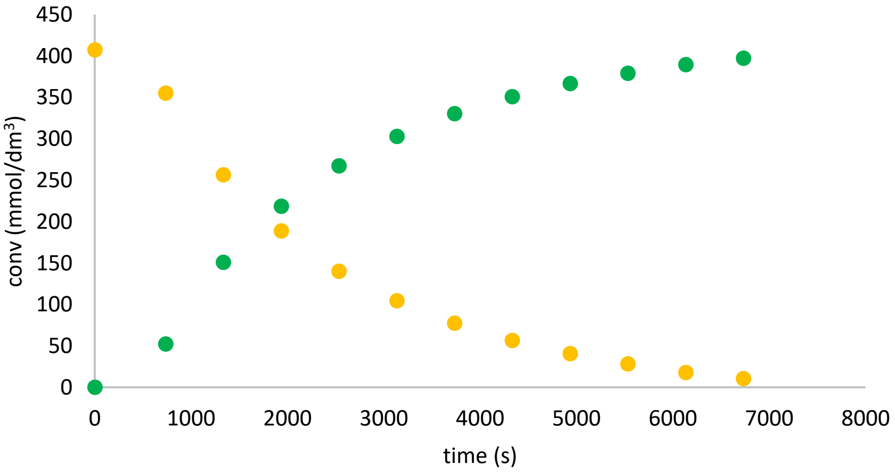

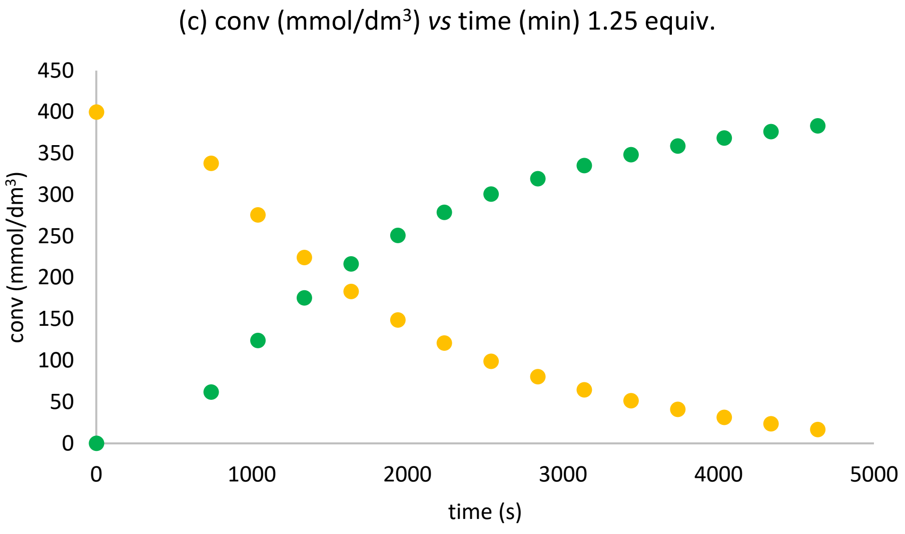

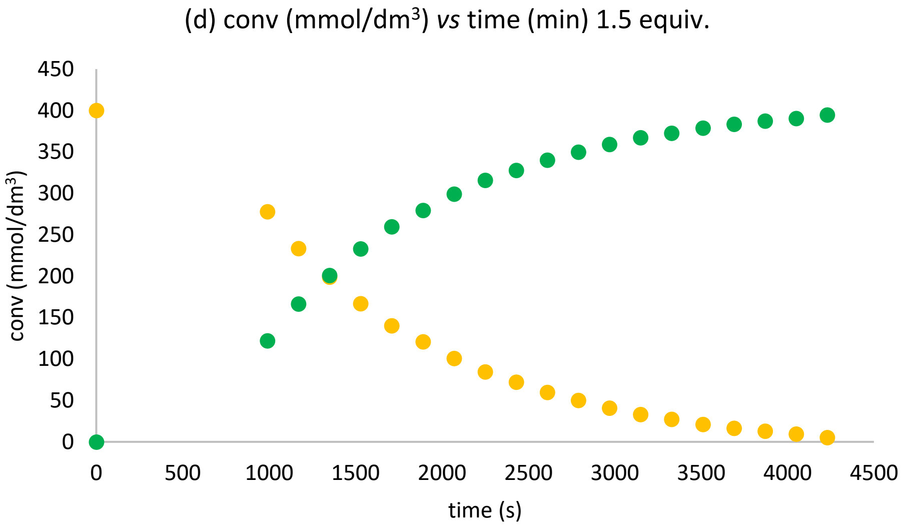

Figure S27 Plot of conversion (mmol/dm^3) of dimethylphenyl silane **12** over time (min) with different amount of benzophenone **11**; a) 0.75, b) 1, c) 1.25, d) 1.5 equiv.

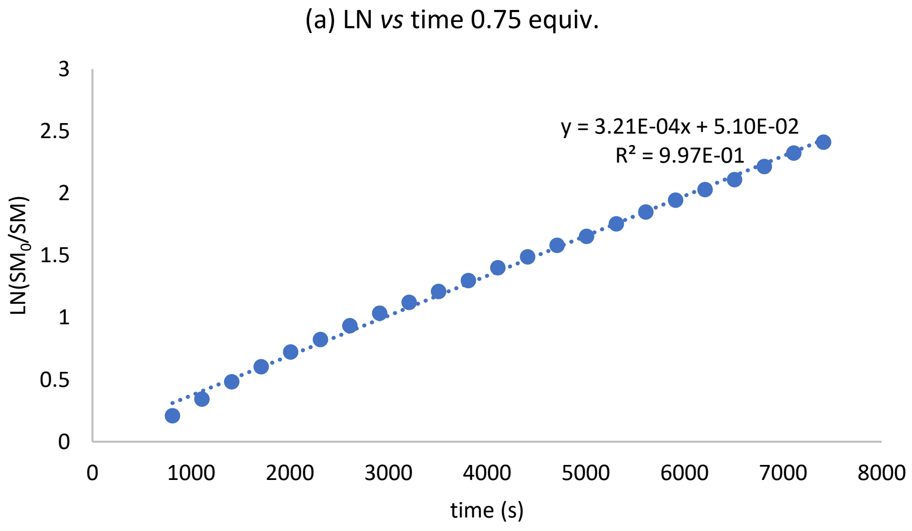

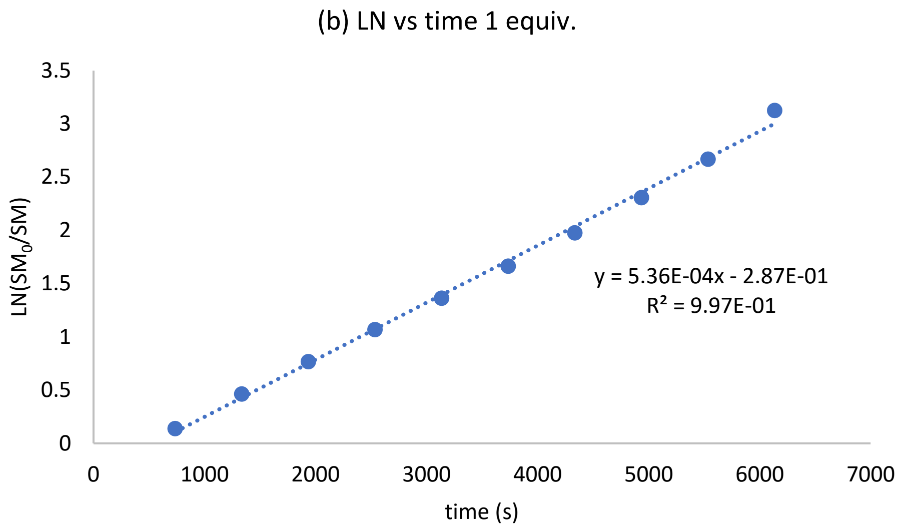

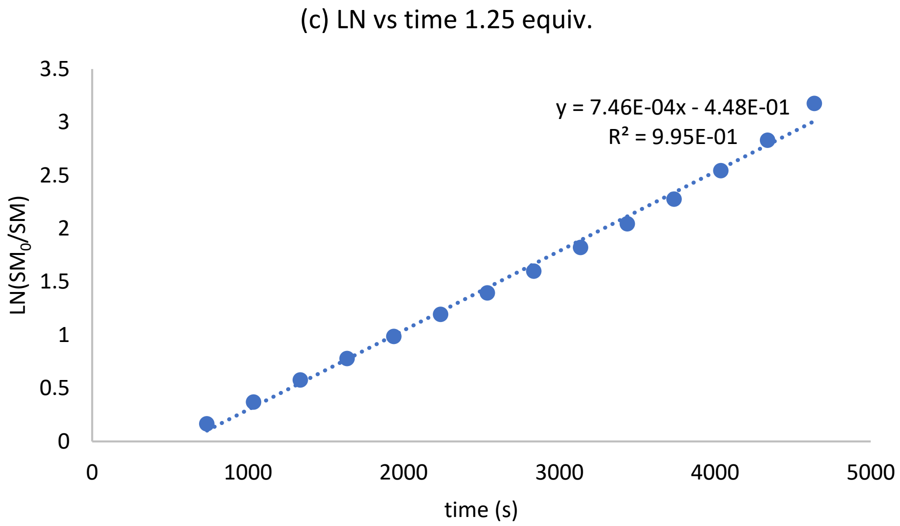

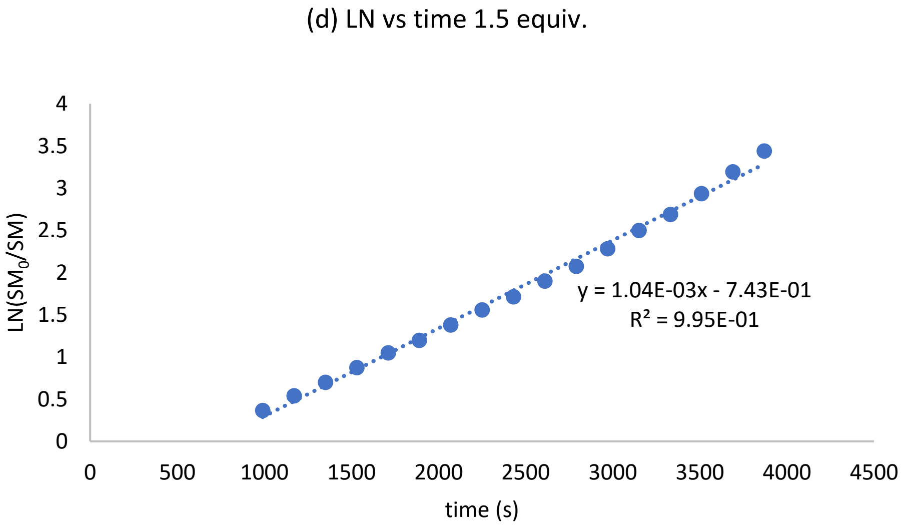

Figure S28 LN(initial starting material/starting material) over time(s) with different amount of benzophenone **11**; a) 0.75, b) 1, c) 1.25, d) 1.5 equiv.

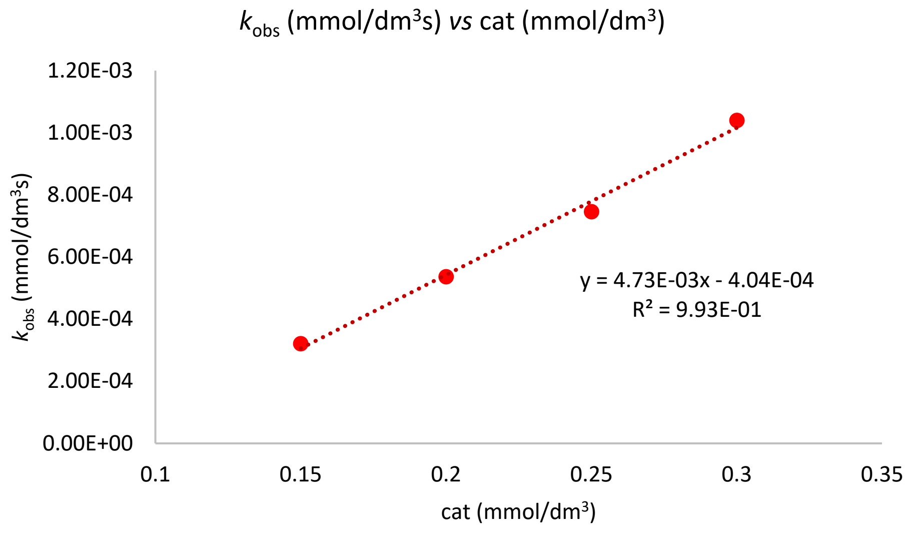

Figure S29 reaction rate (mmol/dm^{3}s) *vs* benzophenone **11** concentration (mmol/dm^3)

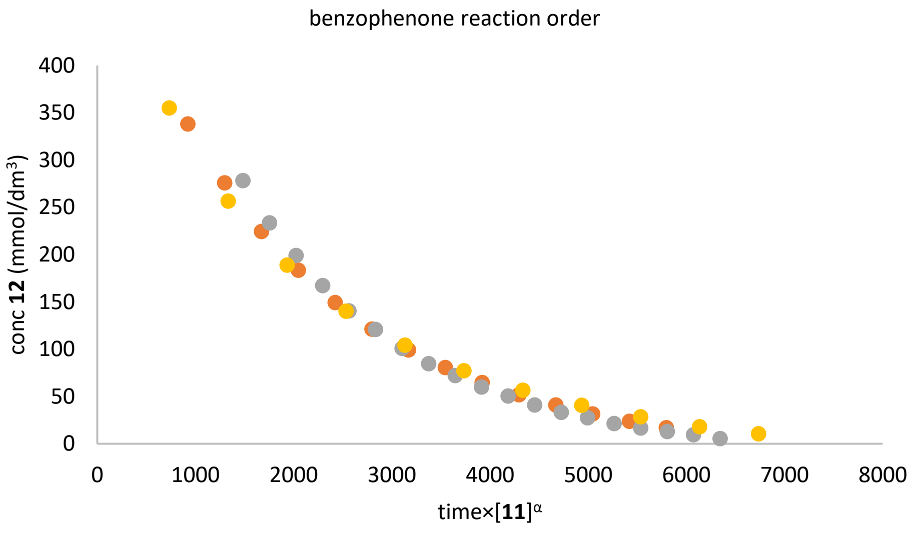

Figure S30 conc **12** (mmol/dm^3) vs time×[**11**]α (s·mmol/dm^3)

Plots in Figure S31 to S34 show the reaction profiles when varying the quantity of silane. Kinetic runs were carried out using benzophenone (0.2 mmol) and dimethylphenyl silane (0.75 to 2 equiv.) with 10 mol% catalyst loadings prepared *in situ* and 2,6-lutidine (10 mol%) in CD_3CN (0.4 M). The reaction was monitored by ^1H NMR heating the spectrometer up to 350K in presence of toluene as internal standard.

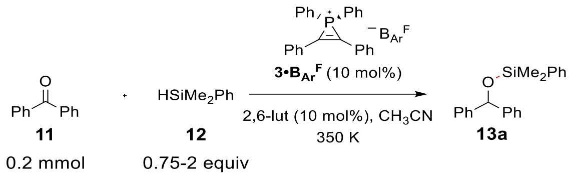

(a) conv (mmol/dm^3) *vs* time (min) 0.75 equiv.

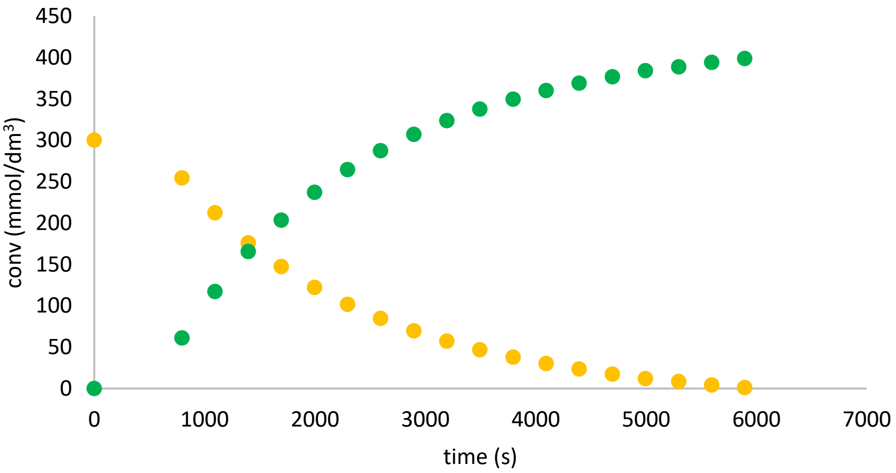

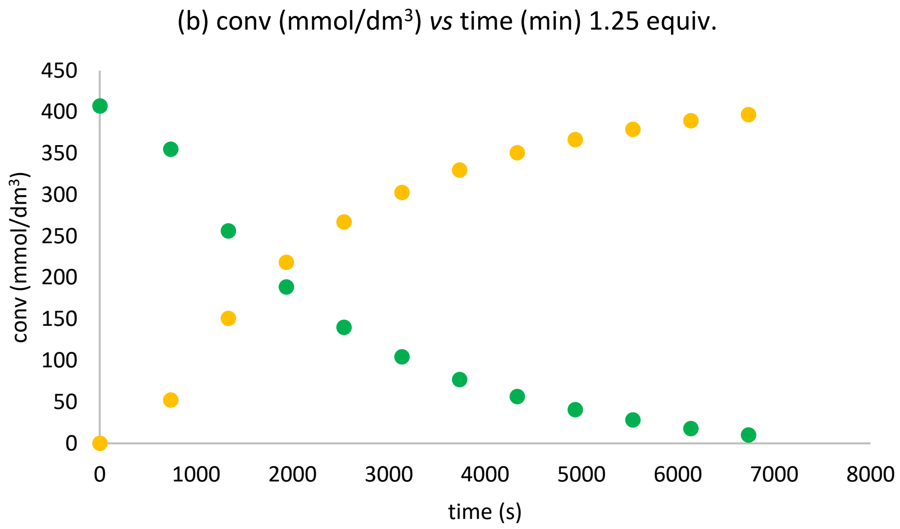

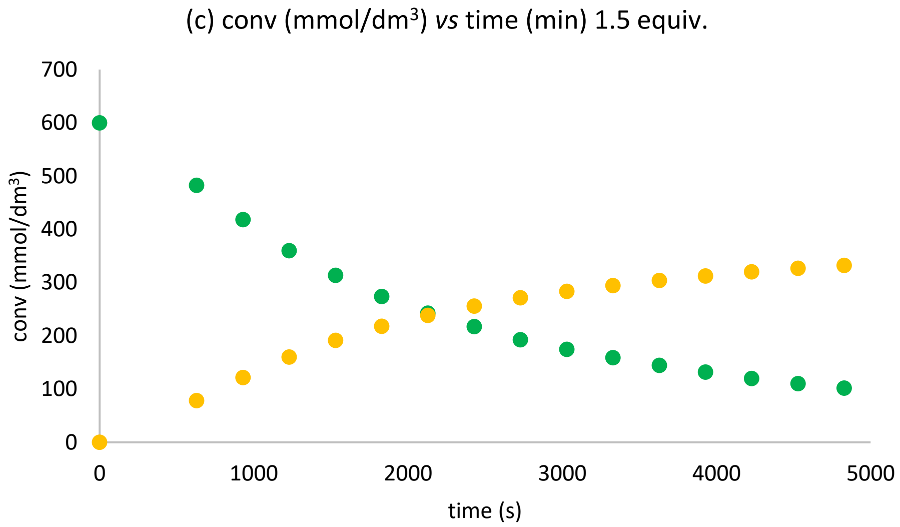

Figure S31 Plot of conversion (mmol/dm^3) of dimethylphenyl silane **12** over time (min) with different amount of dimethylphenyl silane **12**; a) 0.75, b) 1.25, c) 1.5 equiv.

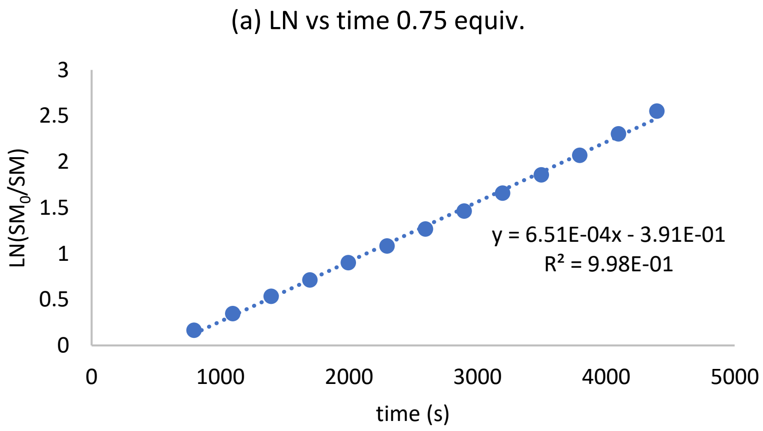

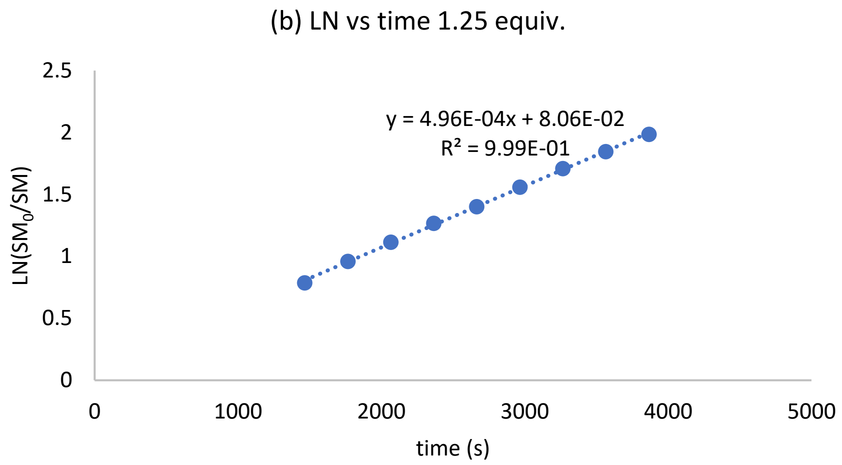

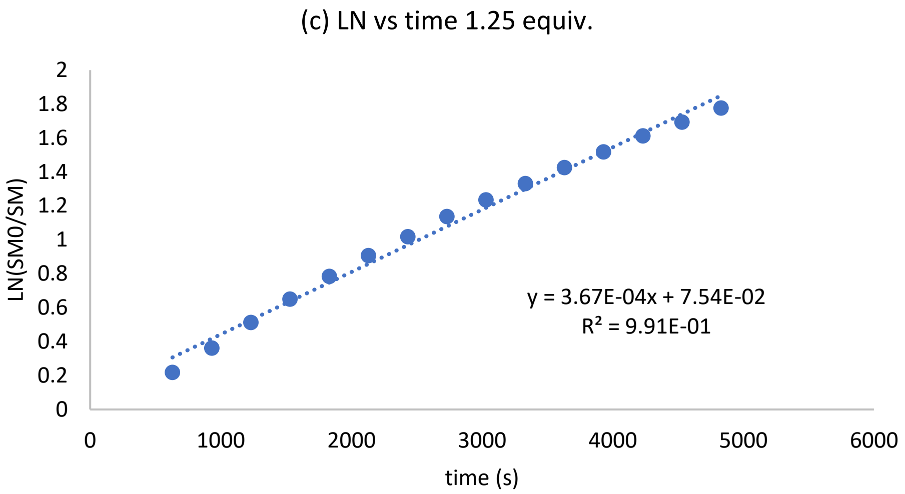

Figure S32 LN(initial starting material/starting material) over time(s) with different amount of dimethylphenylsilane **12**; a) 0.75, b) 1.25, c) 1.5 equiv.

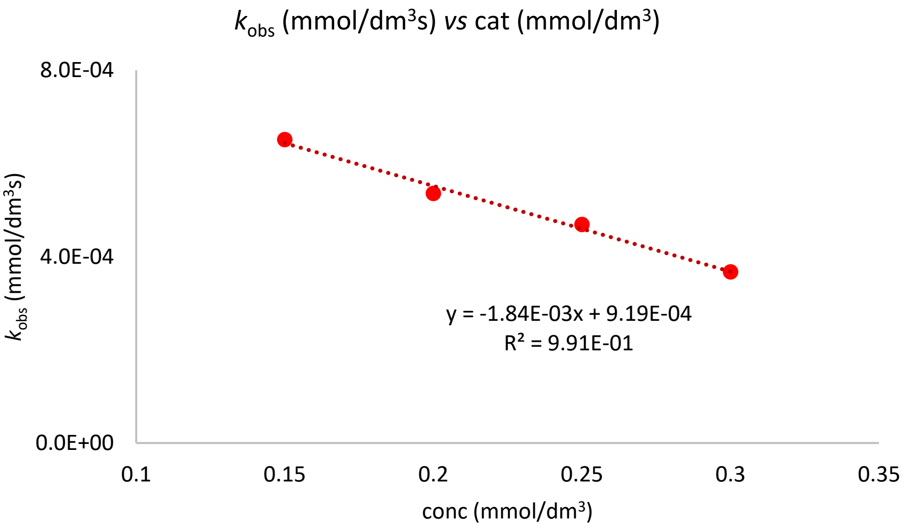

Figure S33 reaction rate (mmol/dm^{3}s) *vs* dimethylphenyl silane **12** concentration (mmol/dm^3)

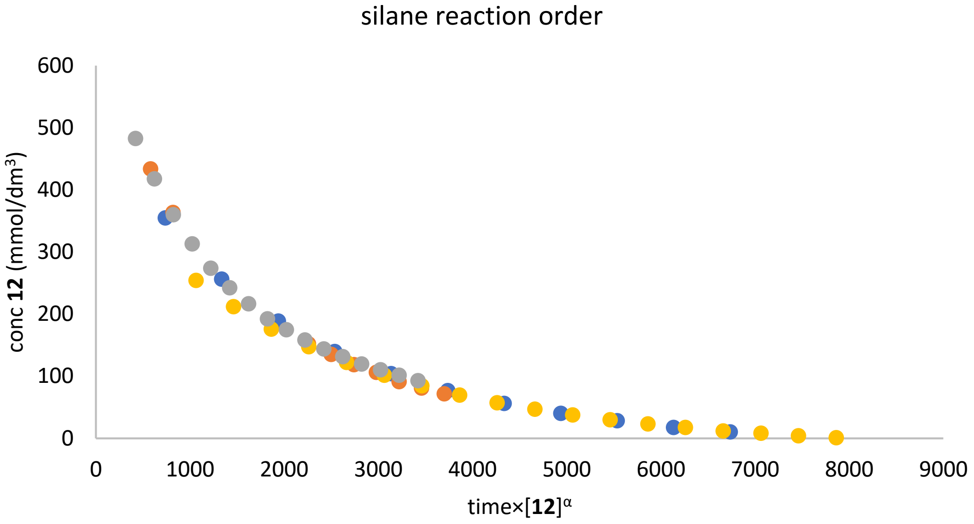

Figure S34 conc **12** (mmol/dm^3) vs time×[**12**]α (s·mmol/dm^3)

Plots in Figure S35 to S37 show the reaction profiles for kinetic runs carried out using variable amounts of benzophenone **11** (0.75 to 2 equiv.) and dimethylphenyl silane **12** (0.2 mmol) with 10 mol% of freshly prepared and isolated catalyst **3·B$_{Ar}$^{F}** without 2,6-lutidine in CD_3CN (0.4 M). The reaction was monitored by ^1H NMR heating the spectrometer up to 325K in presence of toluene as internal standard; the reaction was lowered to allow visualisation of the initial reaction point, because at 350K the reaction proceed to quickly to allow a full kinetic profile.

Important to note is that the same trend in reactivity and order in reagents is observed using in situ prepared (and in the presence of 2,6-lutidine, plots in Figure S27 to S34) **3·B$_{Ar}$^{F}** as when using the isolated **3·B$_{Ar}$^{F}** (no 2,6-lutidine present, shown in the proceeding Figures S35 to S40).

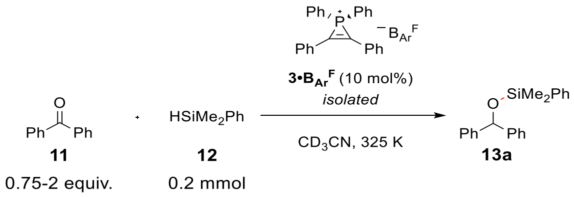

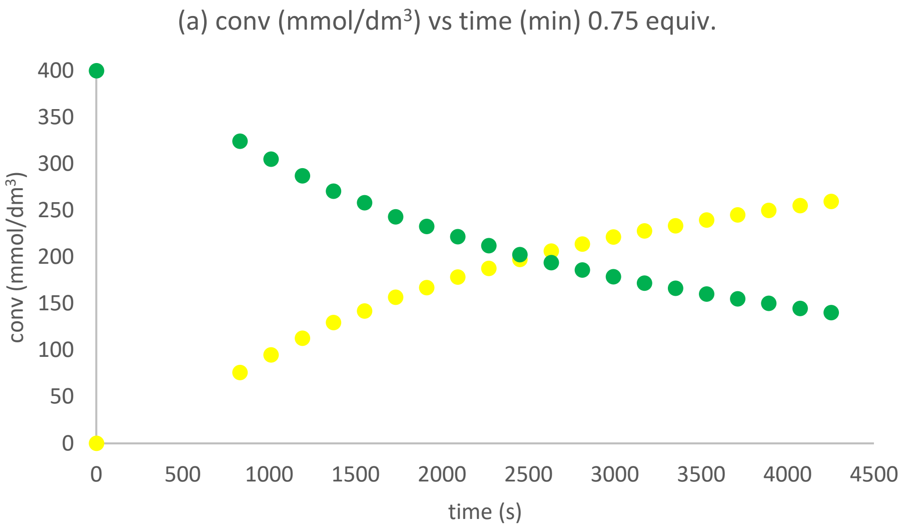
(a) conv (mmol/dm3) vs time (min) 0.75 equiv.
conv (mmol/dm3)
400
350
300
250
200
150
100
50
0
0
500
1000
1500
2000
2500
3000
3500
4000
4500
time (s)

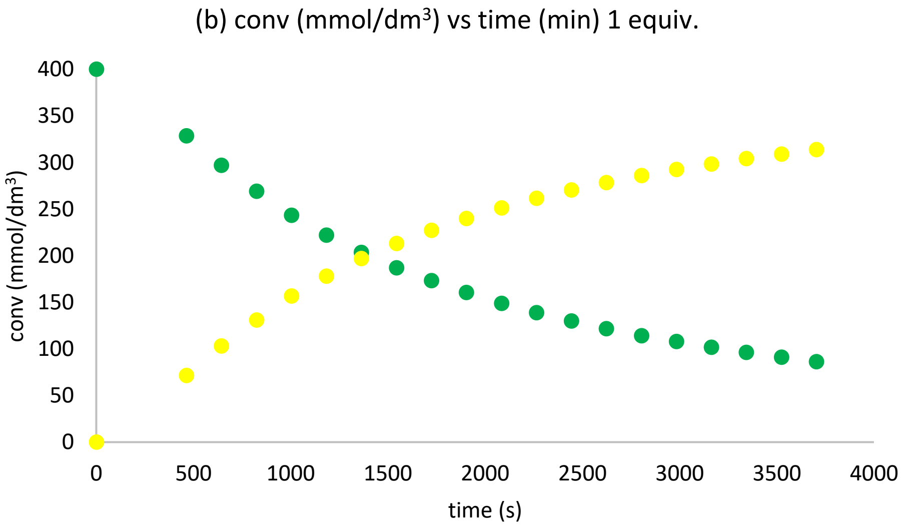
(b) conv (mmol/dm3) vs time (min) 1 equiv.
conv (mmol/dm3)
400
350
300
250
200
150
100
50
0
0
500
1000
1500
2000
2500
3000
3500
4000
time (s)

(c) conv (mmol/dm³) vs time (min) 1.25 equiv.

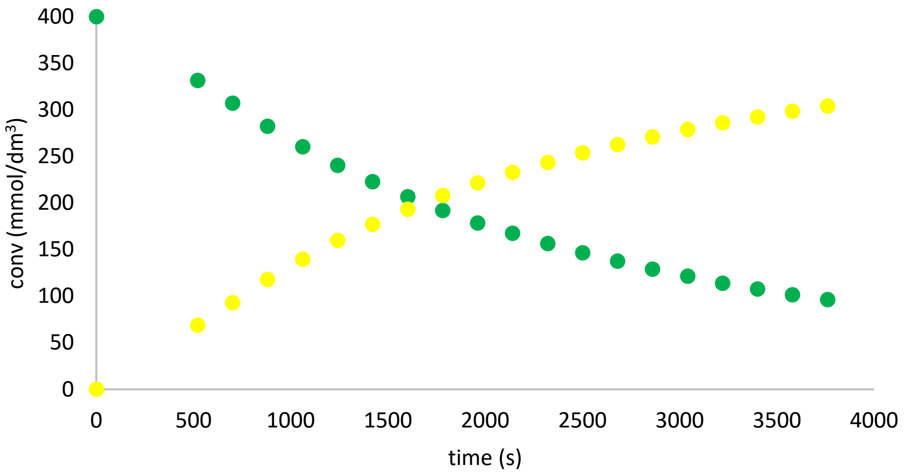

(d) conv (mmol/dm³) vs time (min) 1.5 equiv.

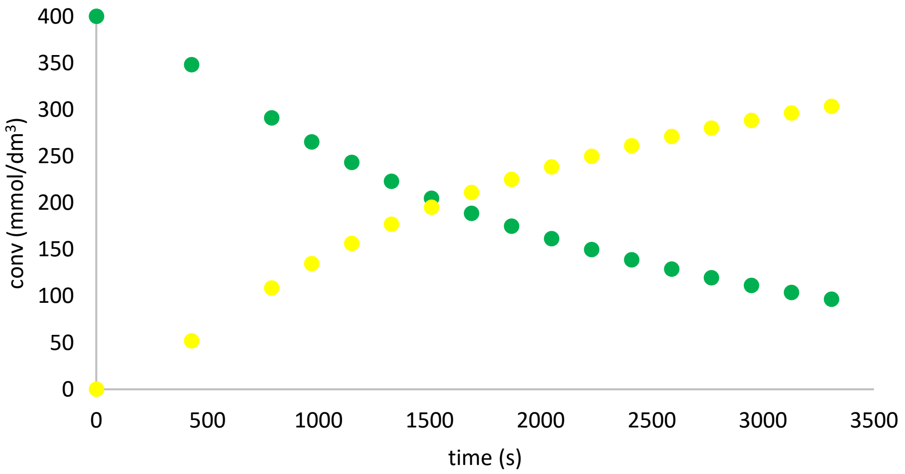

Figure S35 Plot of conversion (mmol/dm^3) of dimethylphenyl silane **12** over time (min) with different amount of benzophenone **11**; a) 0.75, b) 1, c) 1.25, d) 1.5 equiv.

(a) LN vs time 0.75 equiv.

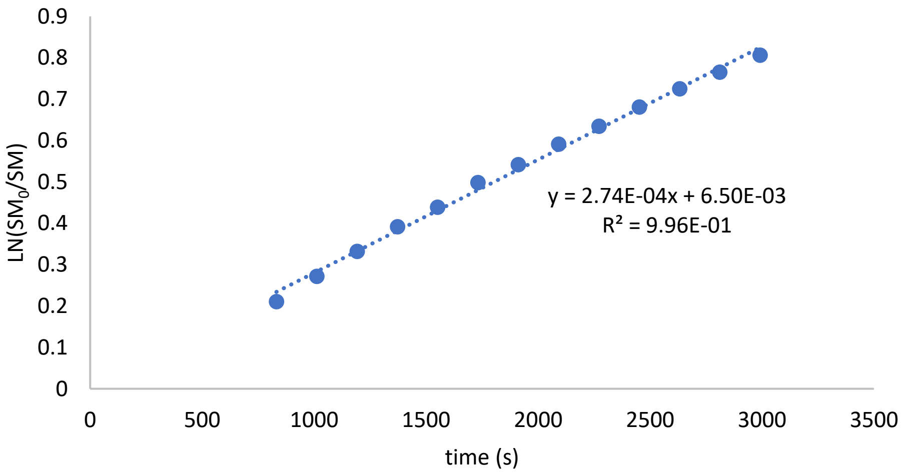

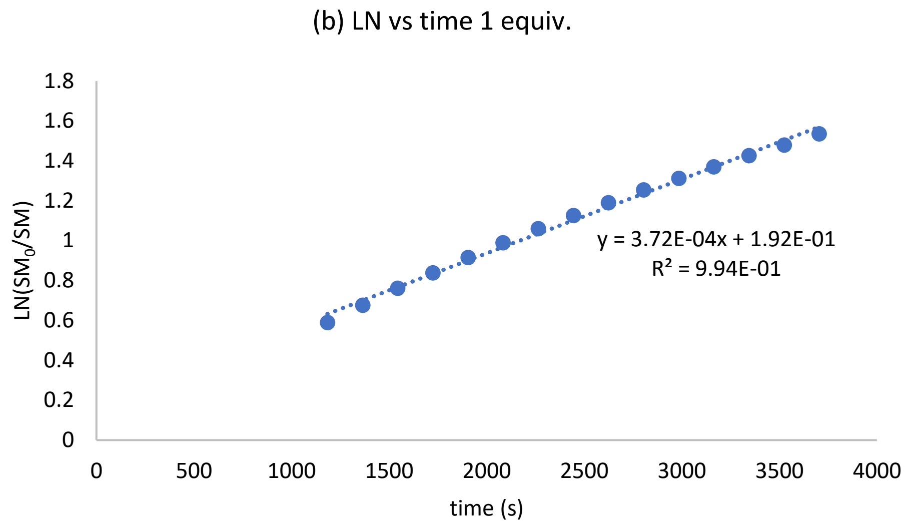

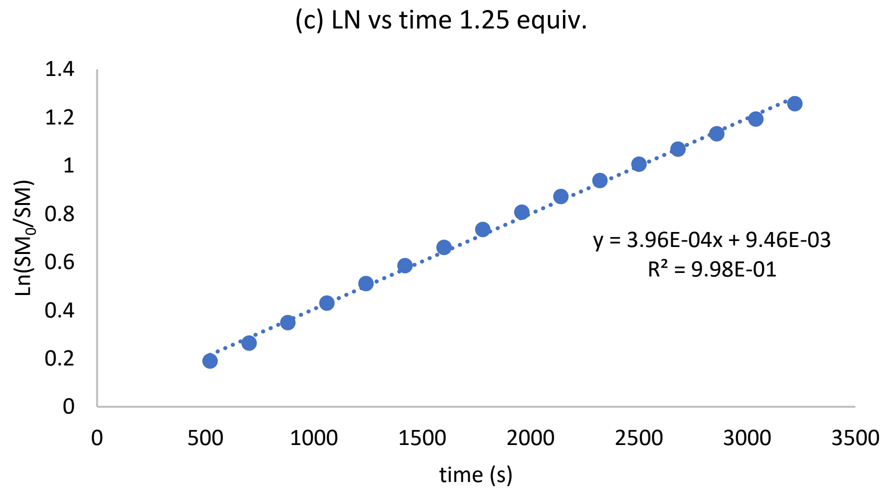

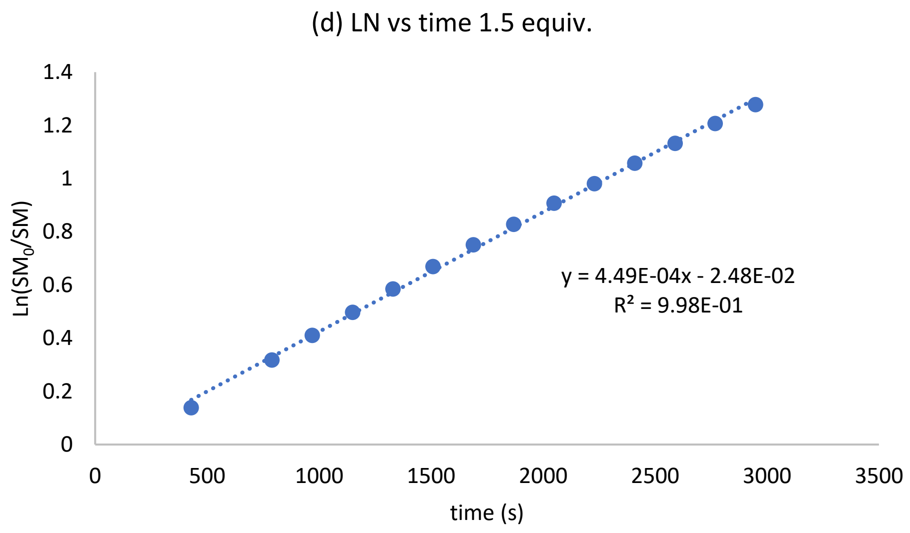

Figure S36 LN(initial starting material/starting material) over time(s) with different amount of benzophenone **11**; a) 0.75, b) 1, c) 1.25, d) 1.5 equiv.

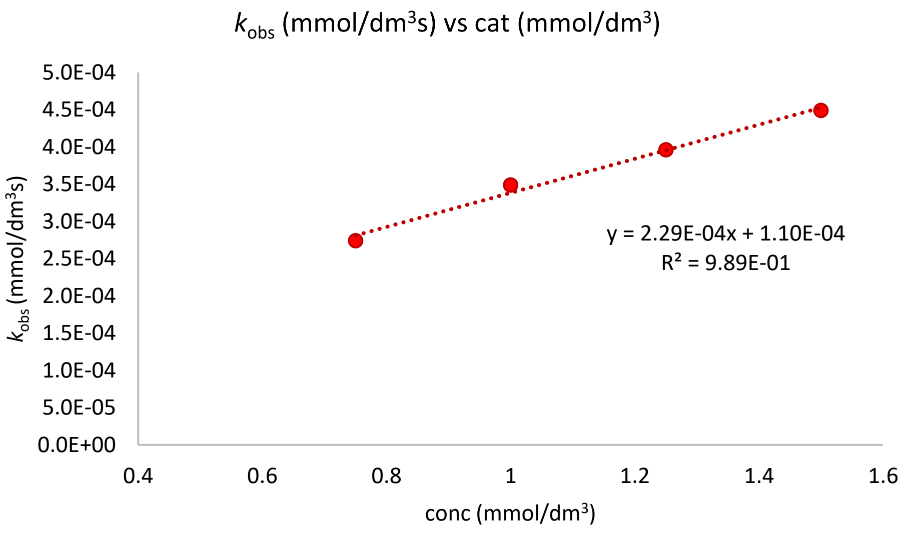

Figure S37 reaction rate (mmol/dm^{3}s) *vs* benzophenone **11** concentration (mmol/dm^3)

Figures S38 to S40 show the reaction profiles for kinetic runs carried out using variable amounts of silane. Kinetic runs were carried out using benzophenone (0.2 mmol) and dimethylphenyl silane (0.75 to 2 equiv.) with 10 mol% of freshly prepared and isolated catalyst **3·B$_{Ar}$^{F}** without 2,6-lutidine in CD_3CN (0.4 M). The reaction was monitored by ^{1}H NMR heating the spectrometer up to 325K in presence of toluene as internal standard; the reaction was lowered to allow visualisation of the initial reaction point, because at 350K the reaction proceed to quickly to allow a full kinetic profile.

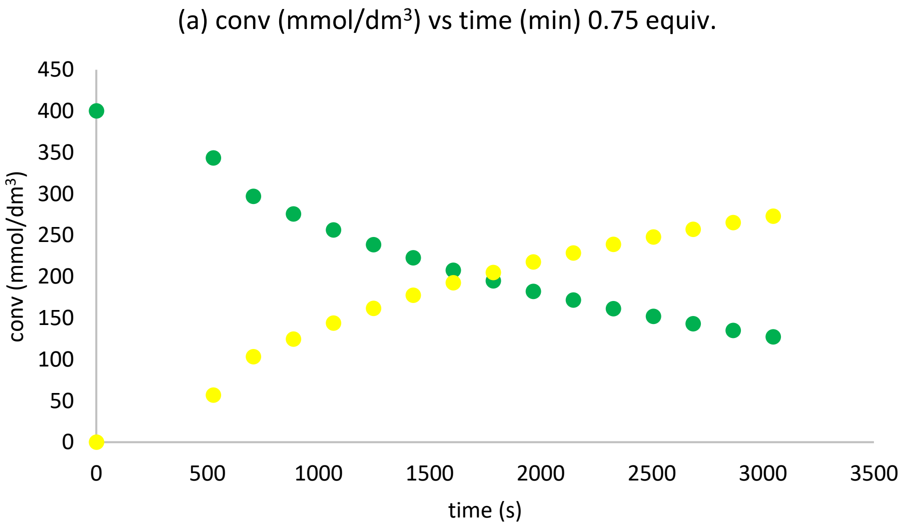

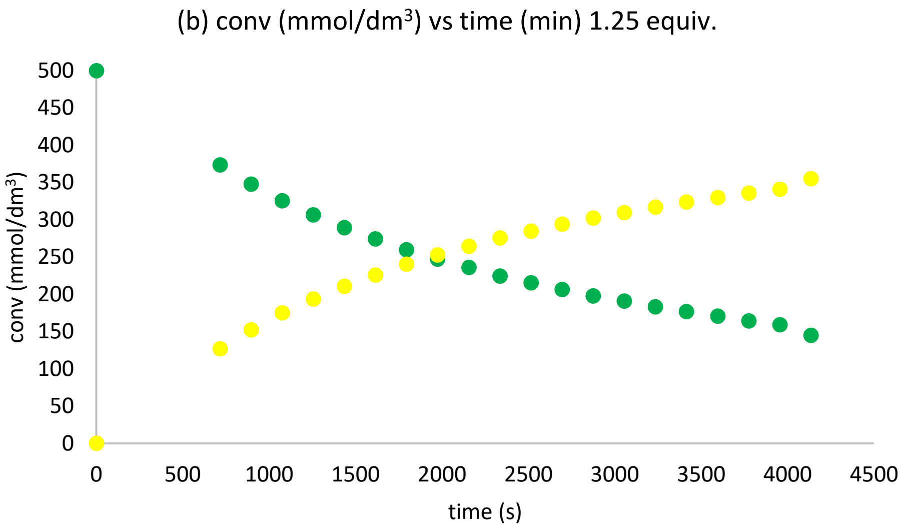

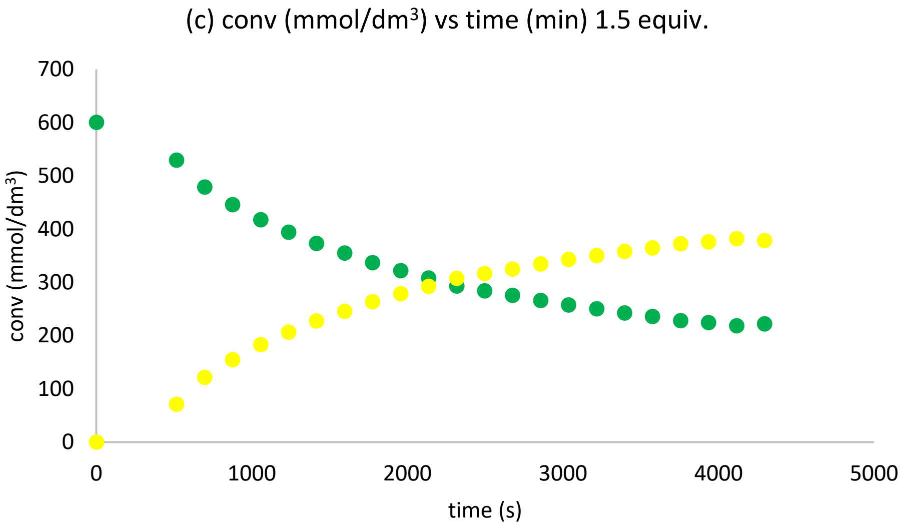

Figure S38 Plot of conversion (mmol/dm^3) of dimethylphenyl silane **12** over time (min) with different amount of dimethylphenyl silane **12**; a) 0.75, b) 1.25, c) 1.5 equiv.

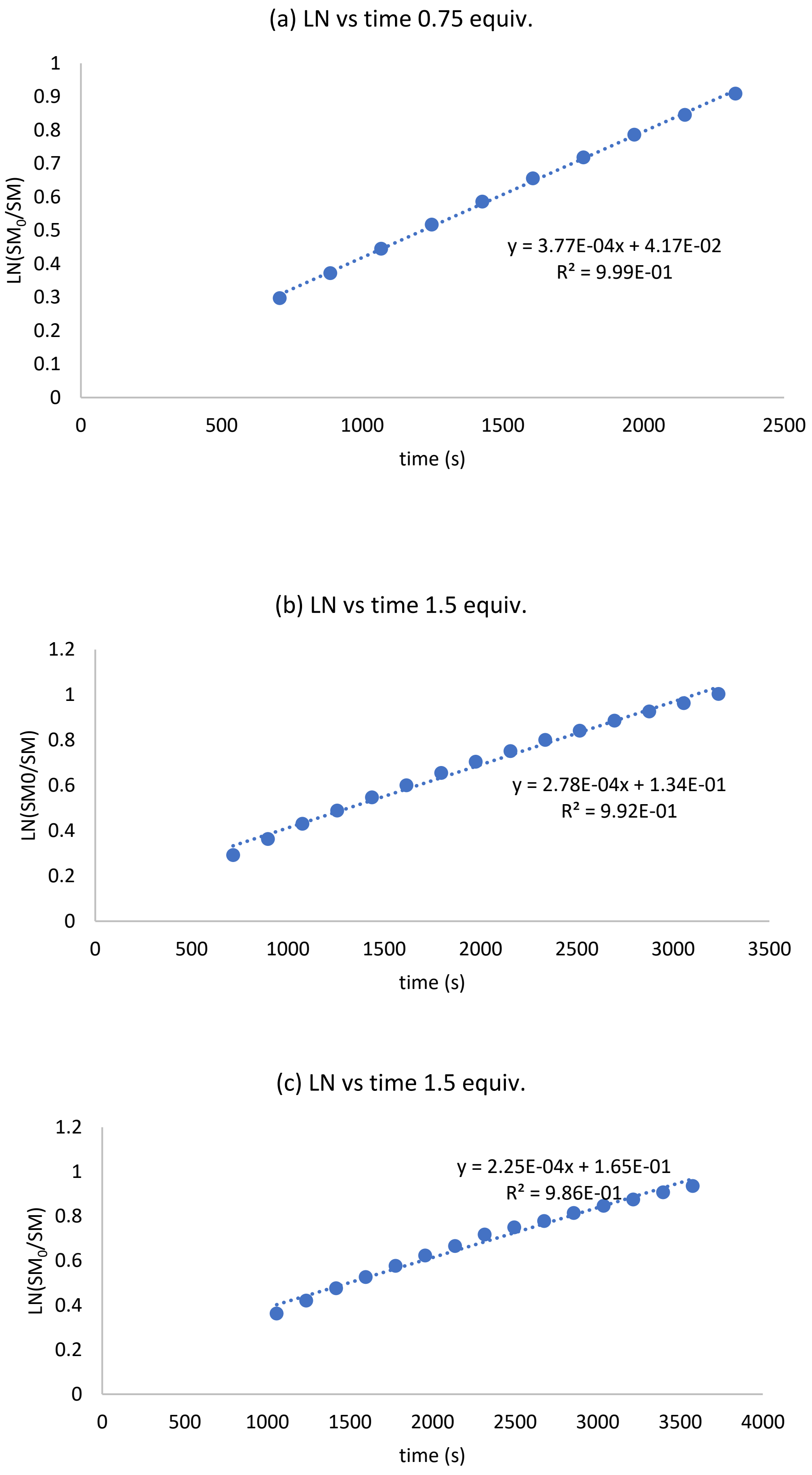

Figure S39 LN(initial starting material/starting material) over time(s) with different amount of **12**; a) 0.75, b) 1.25, c) 1.5 equiv.

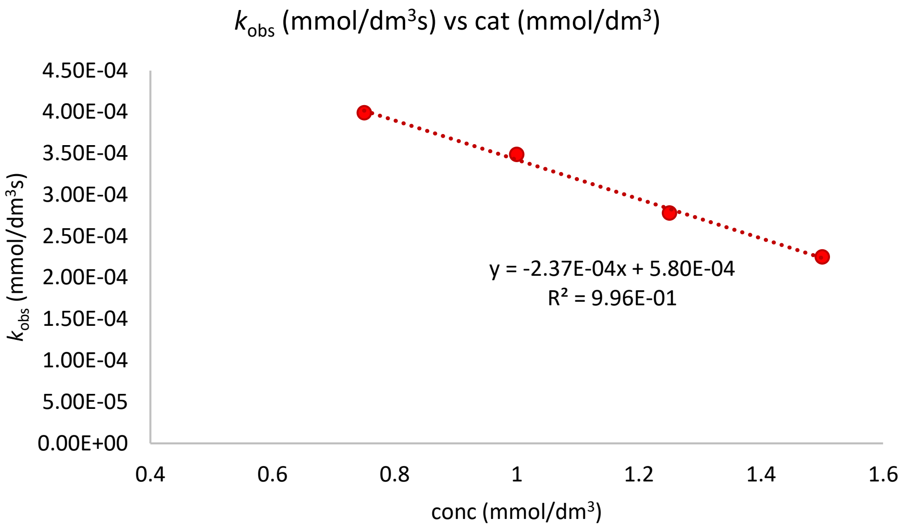

Figure S40 Reaction rate (mmol/dm^3s) *vs* dimethylphenyl silane **12** concentration (mmol/dm^3)

7.4.1 Inhibition kinetics

Inhibition kinetic analysis was performed on the reduction of benzophenone **11** (0.2 mmol), dimethylphenyl silane **12** (0.2 mmol) and (benzhydryloxy)dimethyl(phenyl)silane **13a** (0.1 mmol), with 10 mol% catalyst loadings in CD_3CN (0.4 M). The reaction was monitored by ^1H NMR heating the spectrometer up to 350 K in presence of toluene as internal standard. Product inhibition is not observed with similar reaction rate (slightly slower with **13a**, yellow dots) to the reaction with a 1 to 1 ratio of starting materials **11** to **12** (Figure S41).

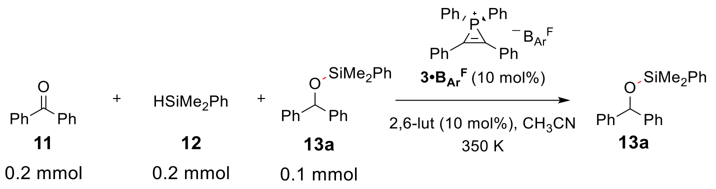

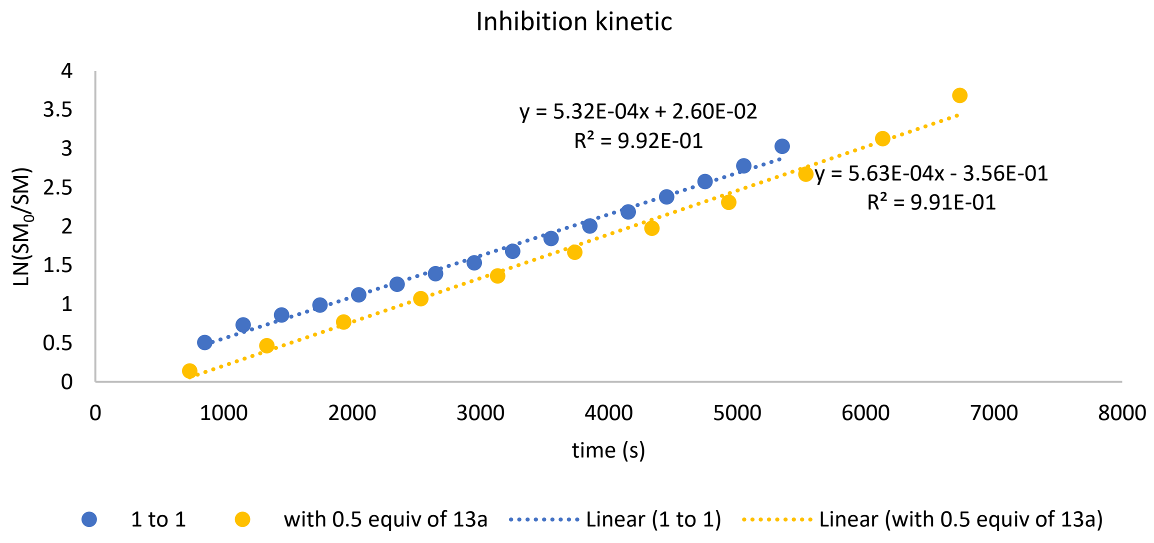

Figure S41 Comparison of the reaction rate with vs without 0.5 equiv. of product **13a**.

8. NMR, Mass and IR Spectra

^{1}H NMR

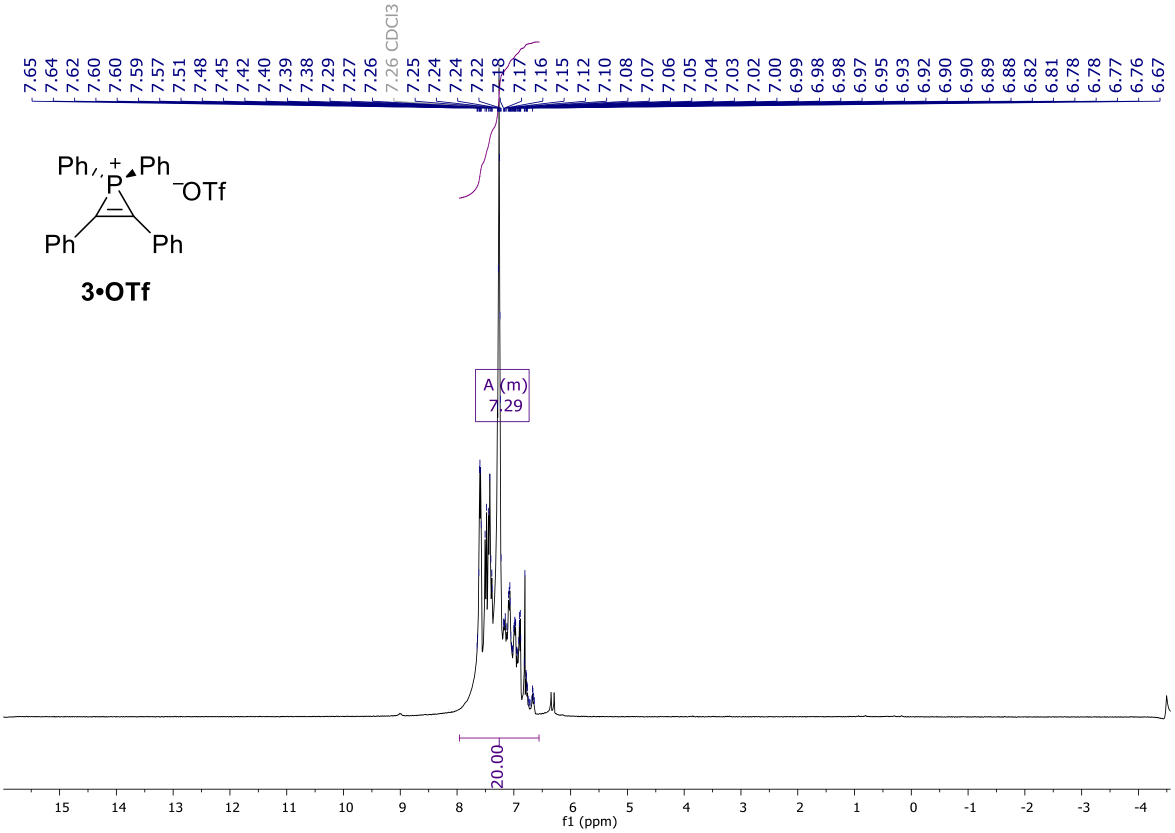

$^{13}C\{^{1}H\}$ NMR

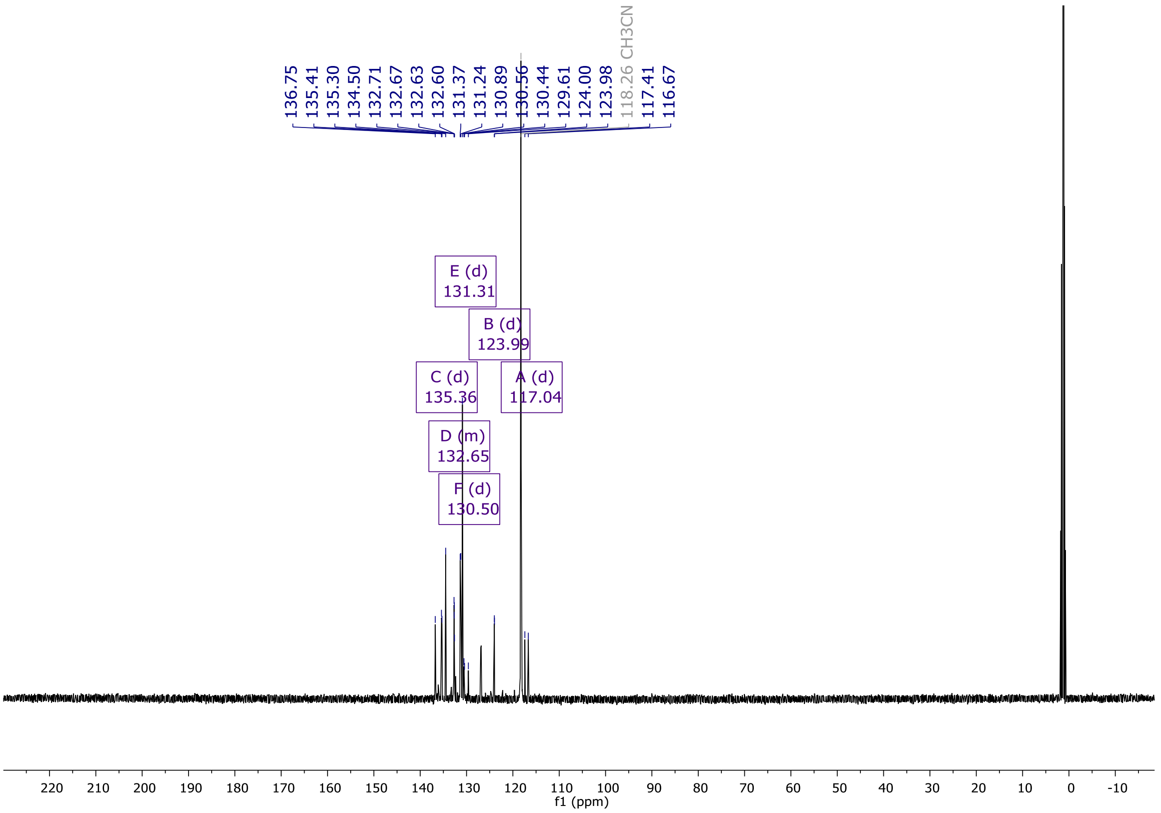

$^{31}P\{^{1}H\}$ NMR

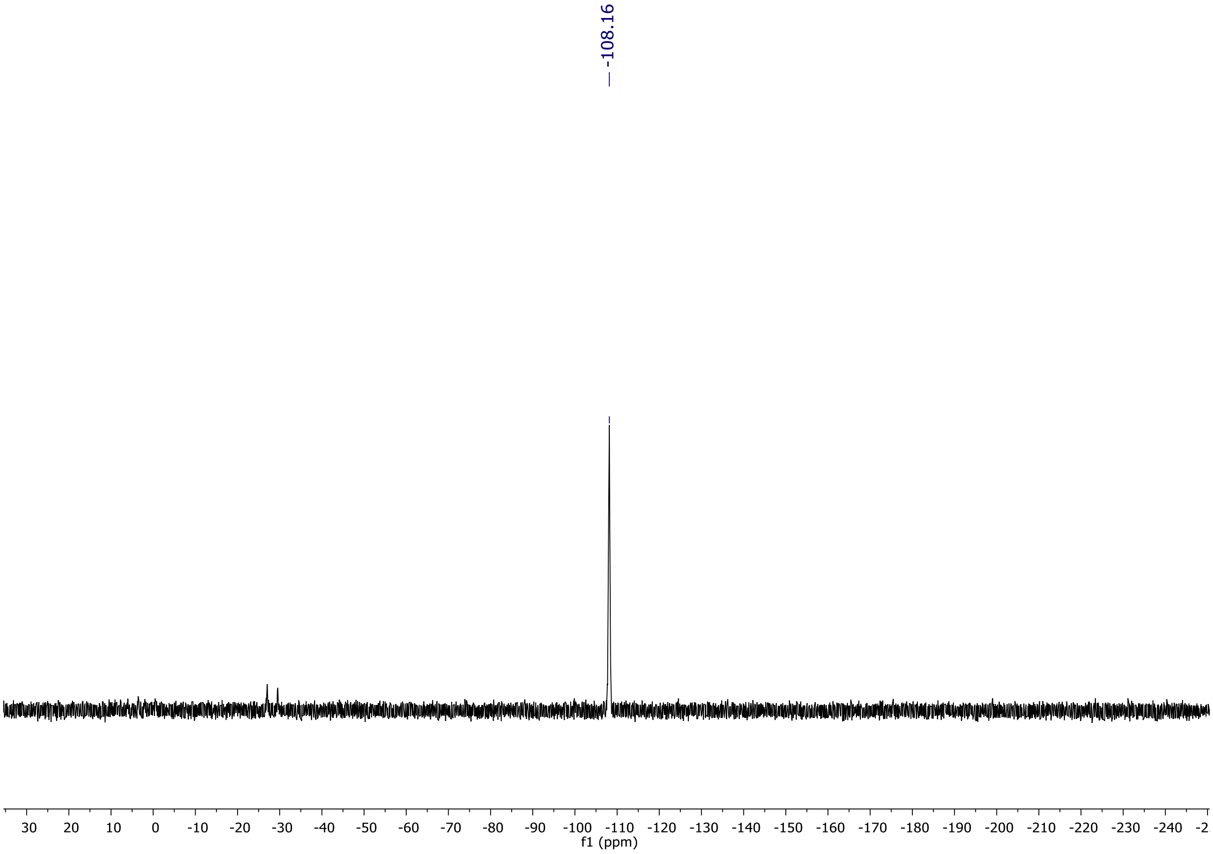

^{19}F NMR

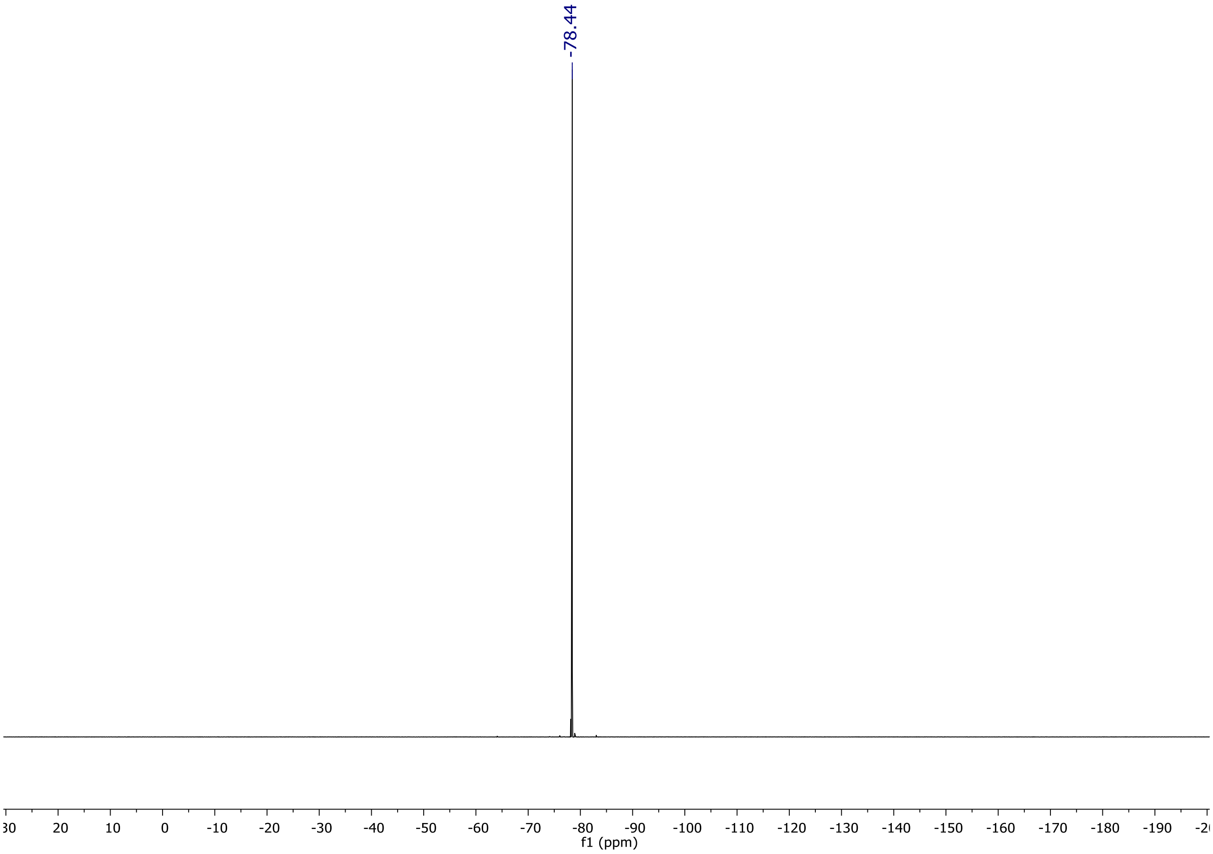

^{1}H NMR

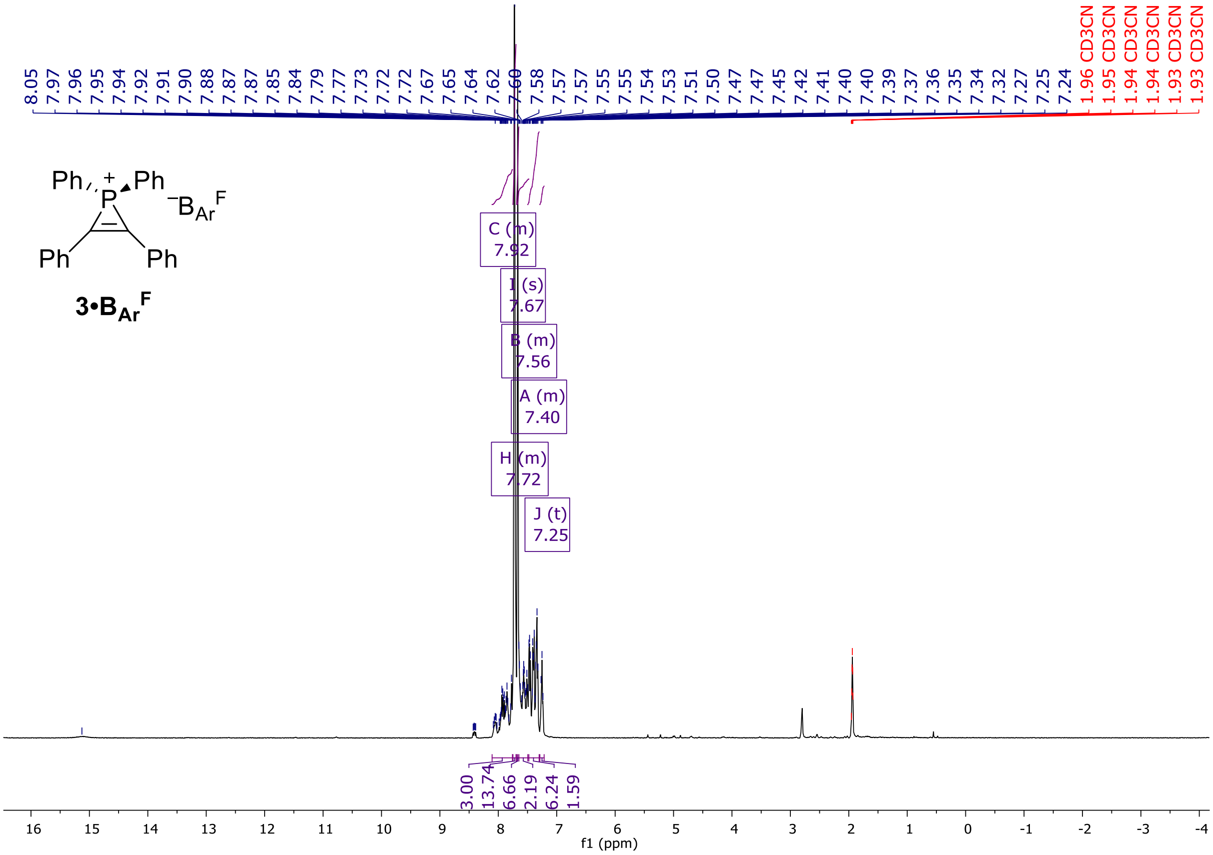

$^{13}C\{^{1}H\}$ NMR

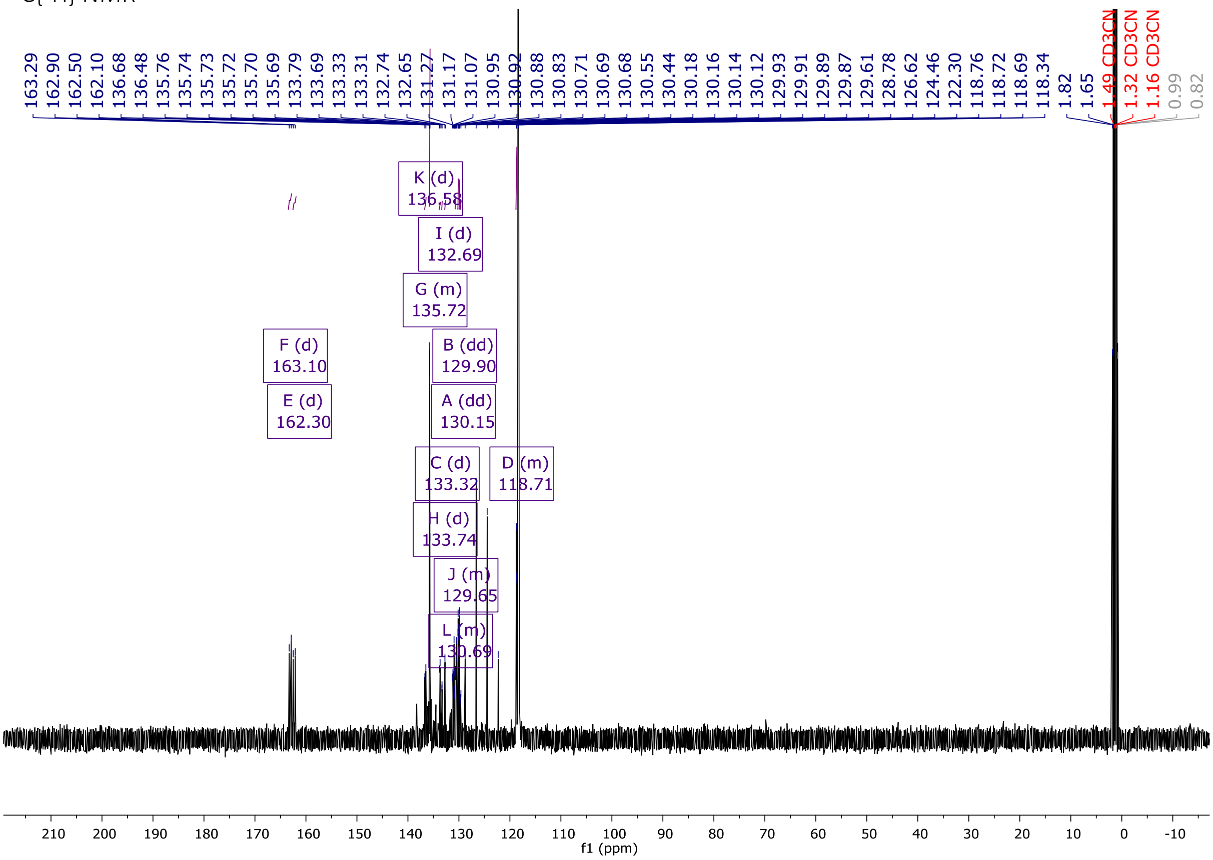

$^{31}P\{^1H\}$ NMR

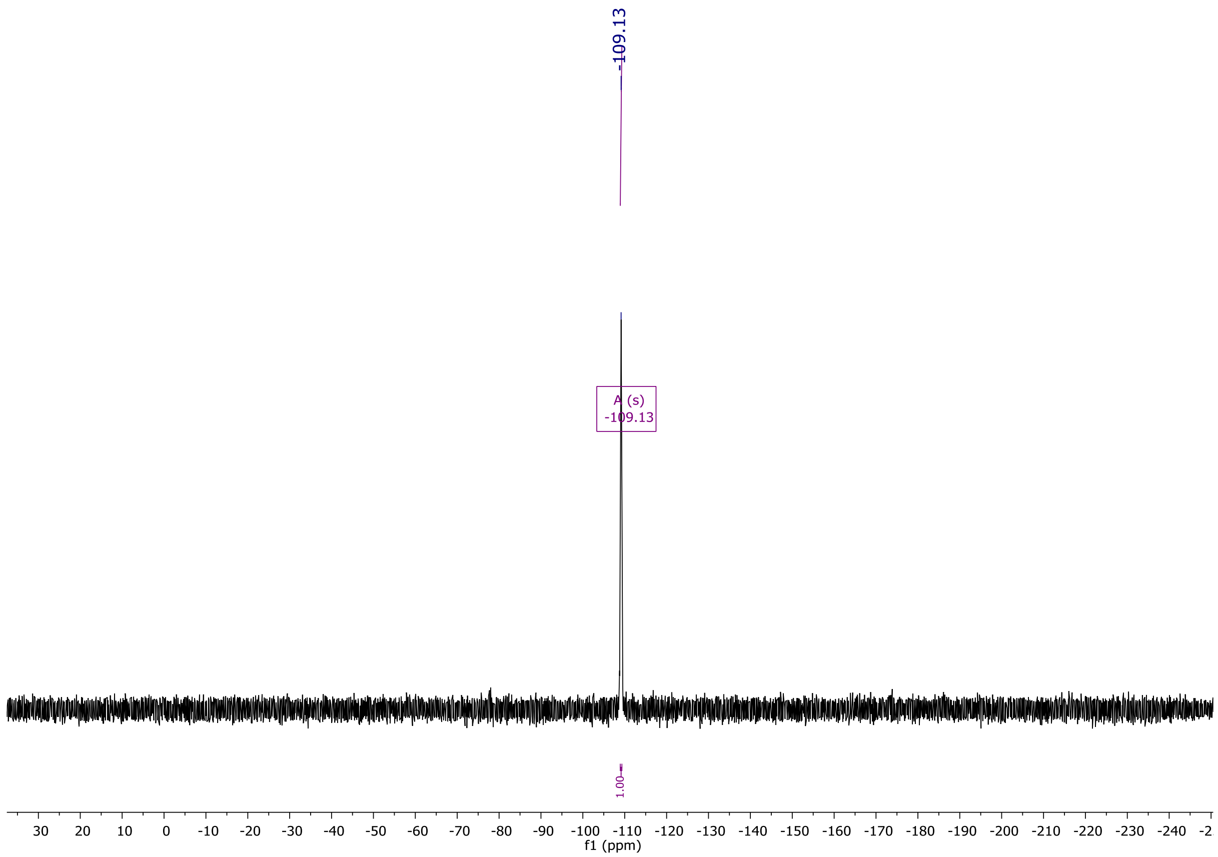

$^{11}B\{^1H\}$ NMR

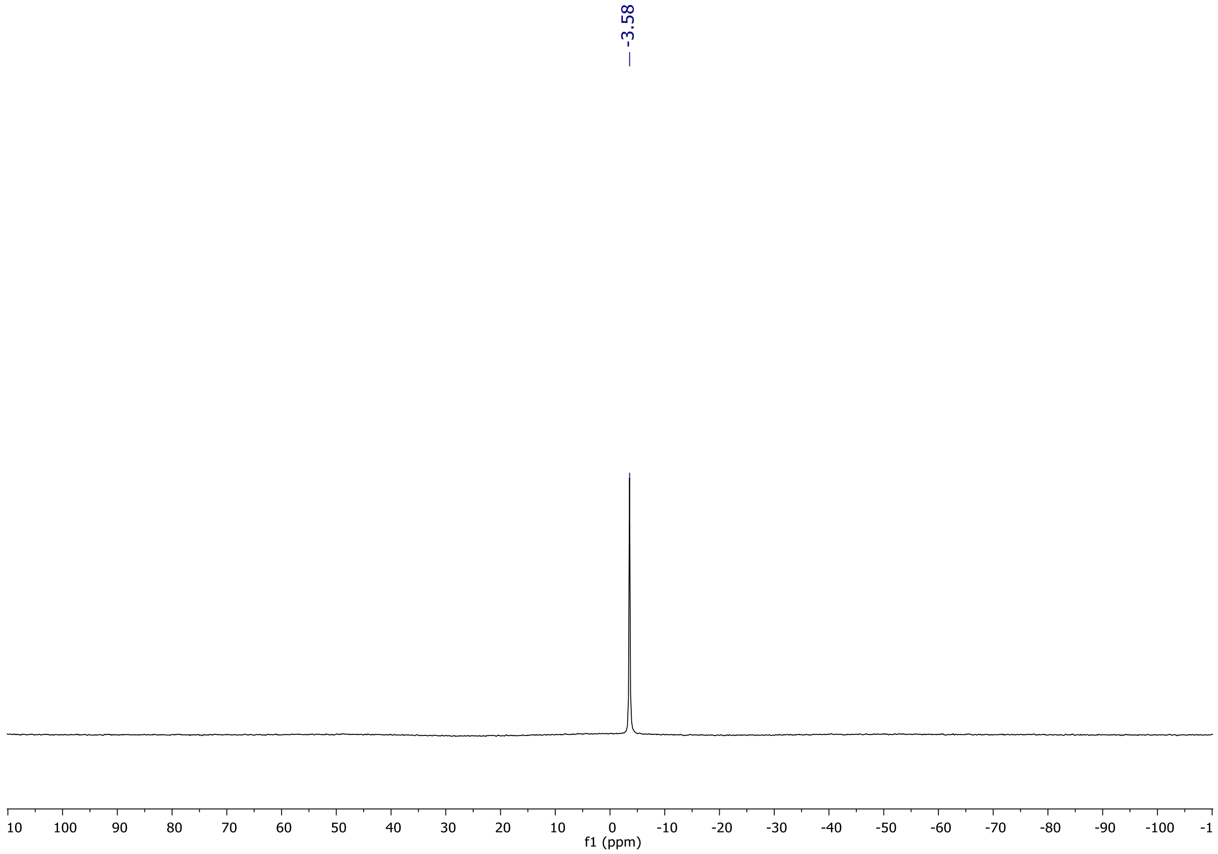

$^{19}F\{^1H\}$ NMR

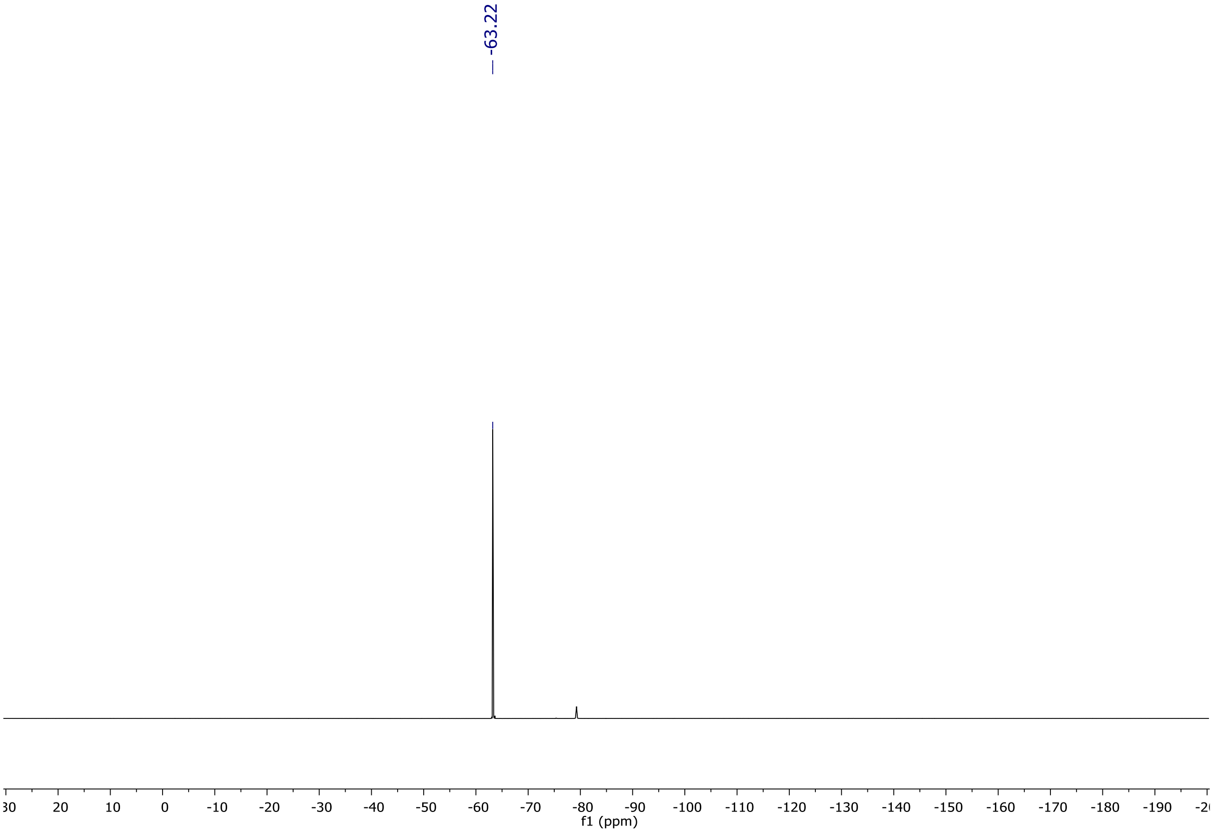

^{1}H NMR

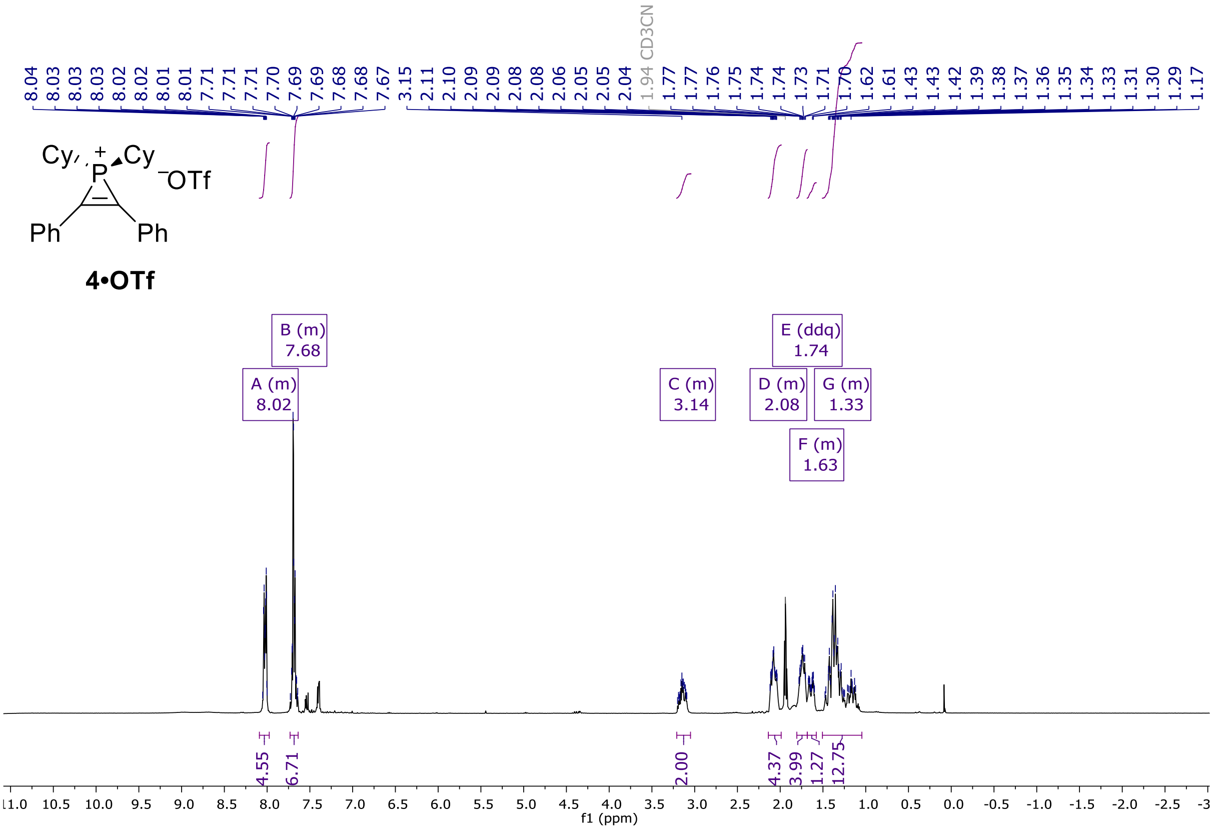

$^{13}C\{^{1}H\}$ NMR

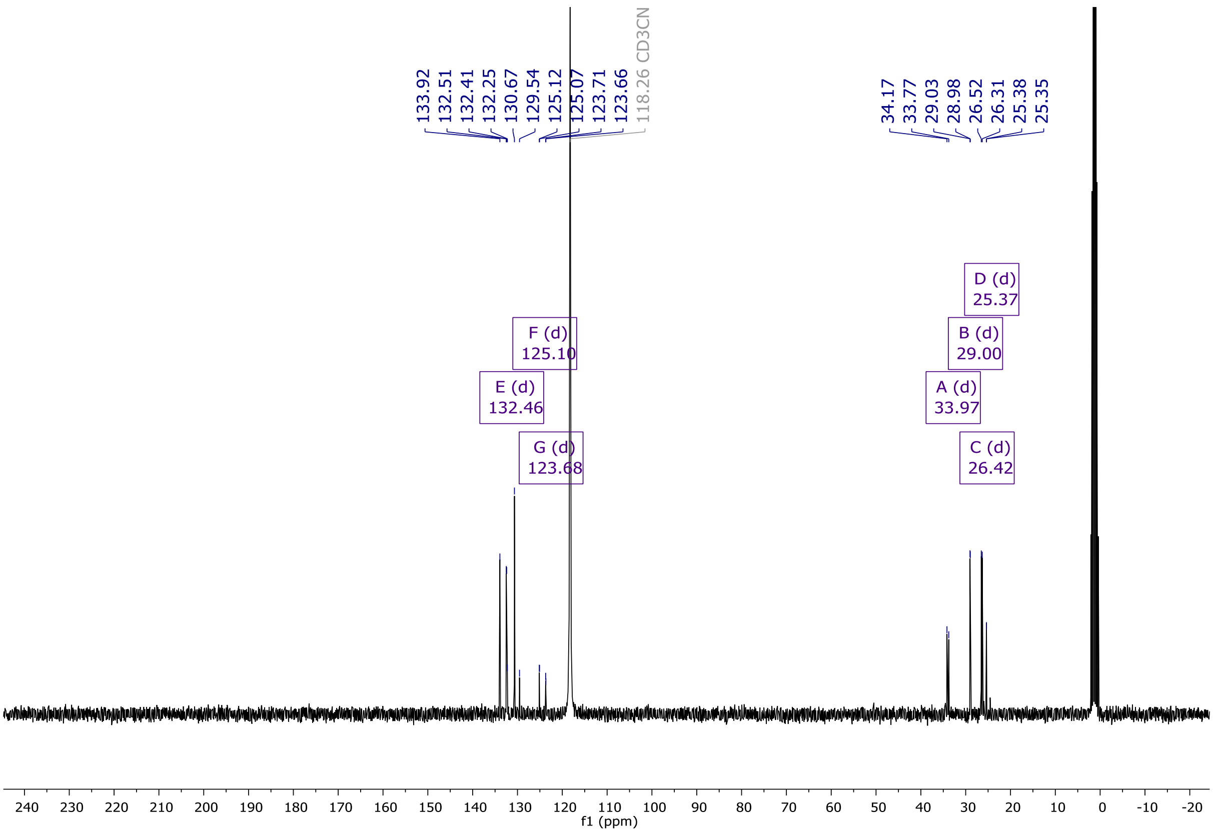

$^{31}P\{^1H\}$ NMR

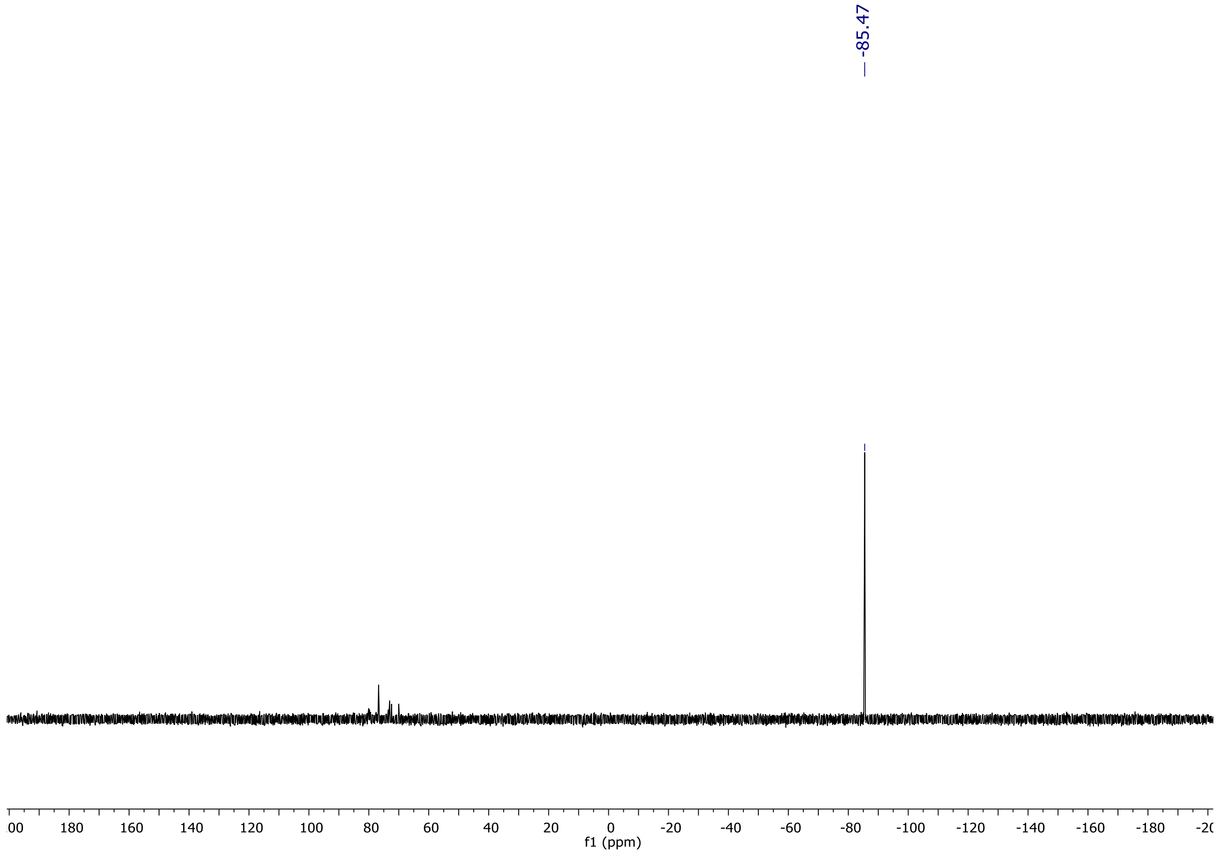

$^{19}F\{^1H\}$ NMR

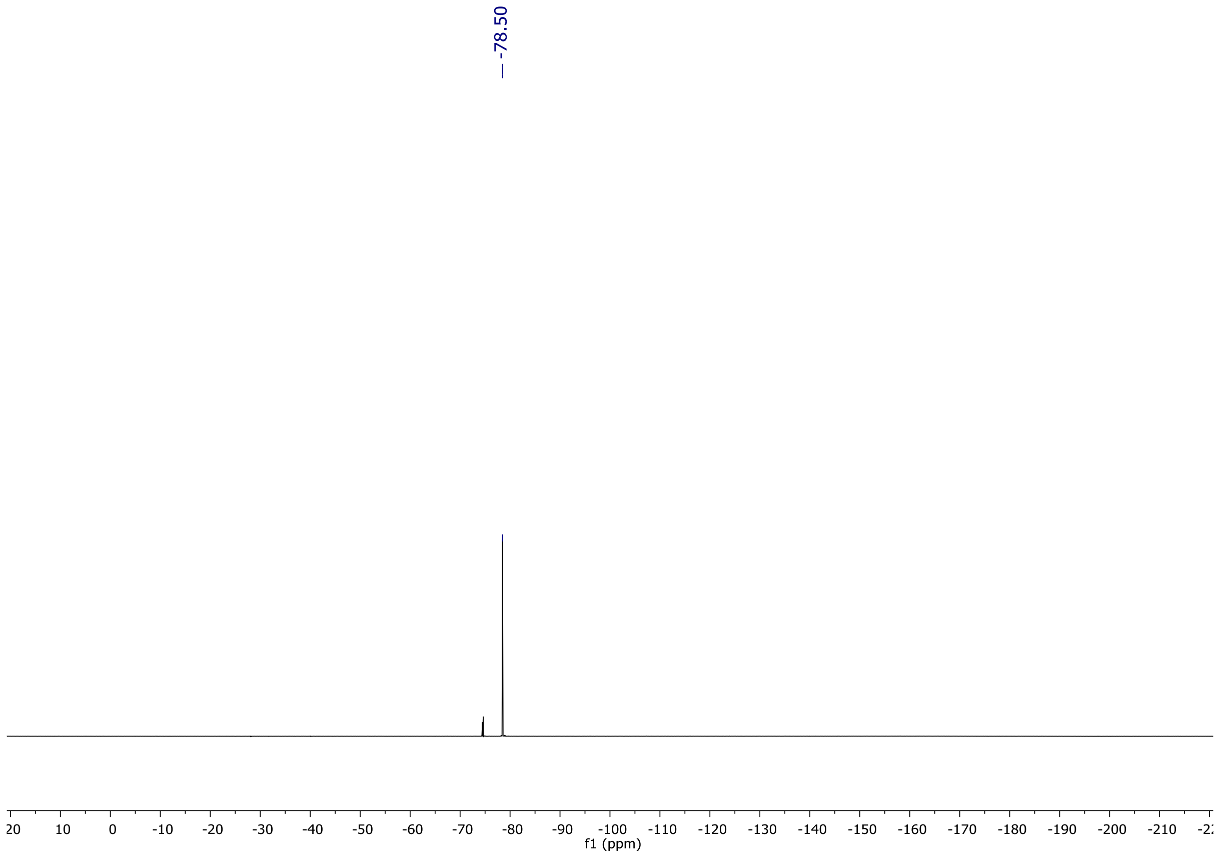

^{1}H NMR

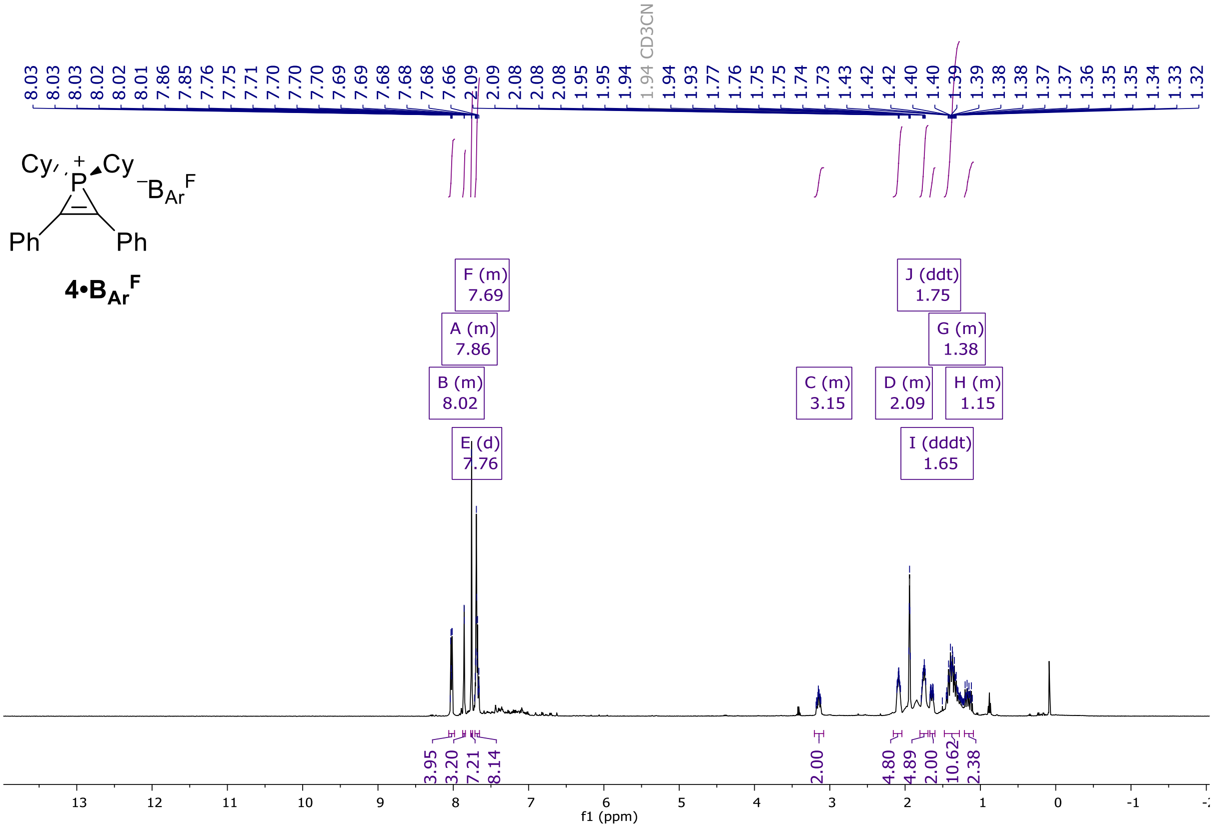

4•B$_{Ar}$^{F}

^{13}C{^{1}H} NMR

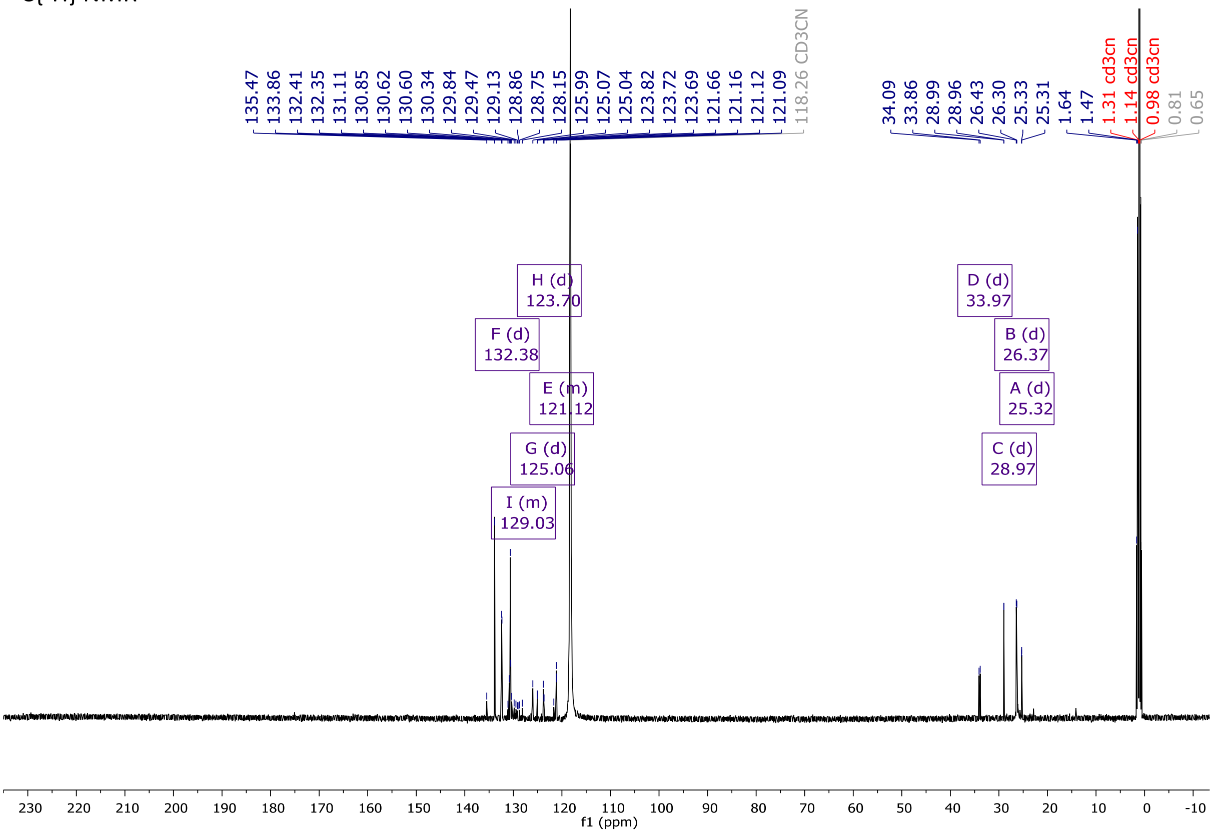

$^{31}P\{^1H\}$ NMR

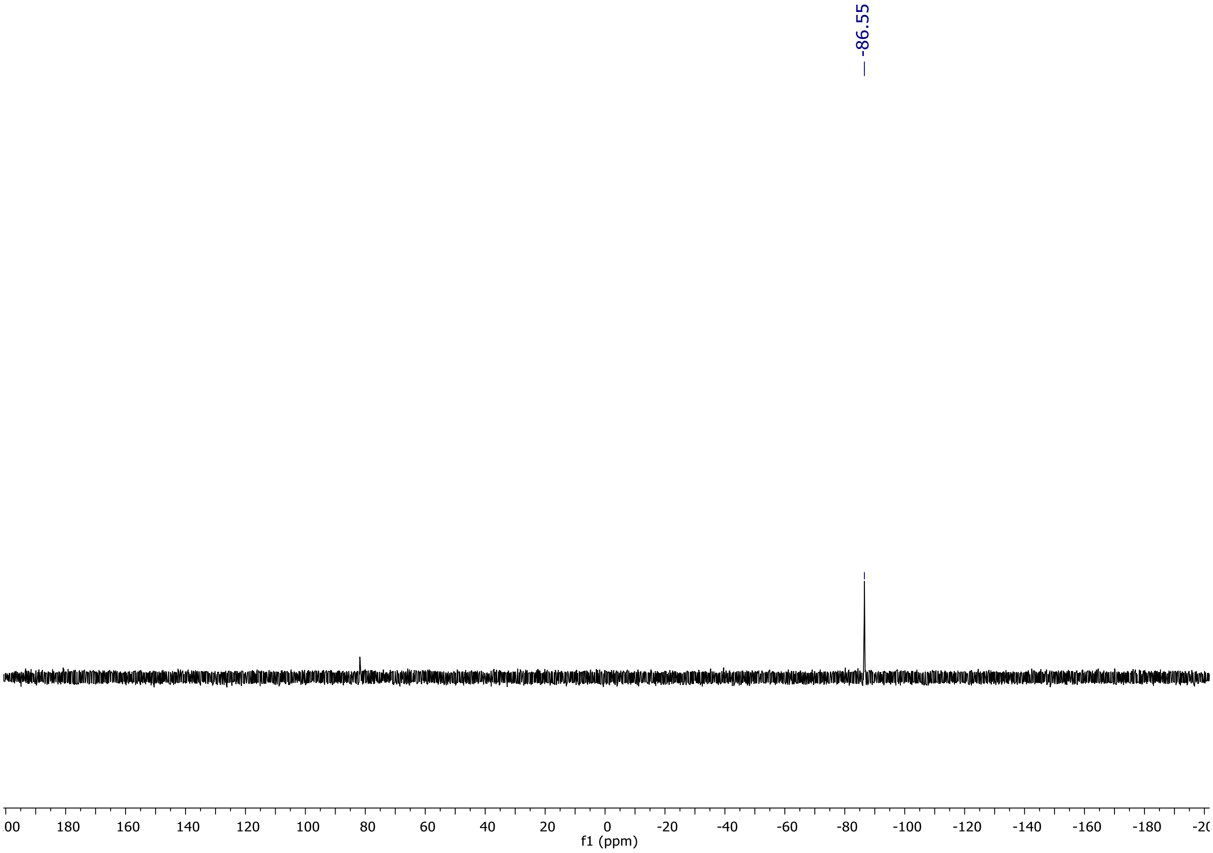

$^{19}F\{^1H\}$ NMR

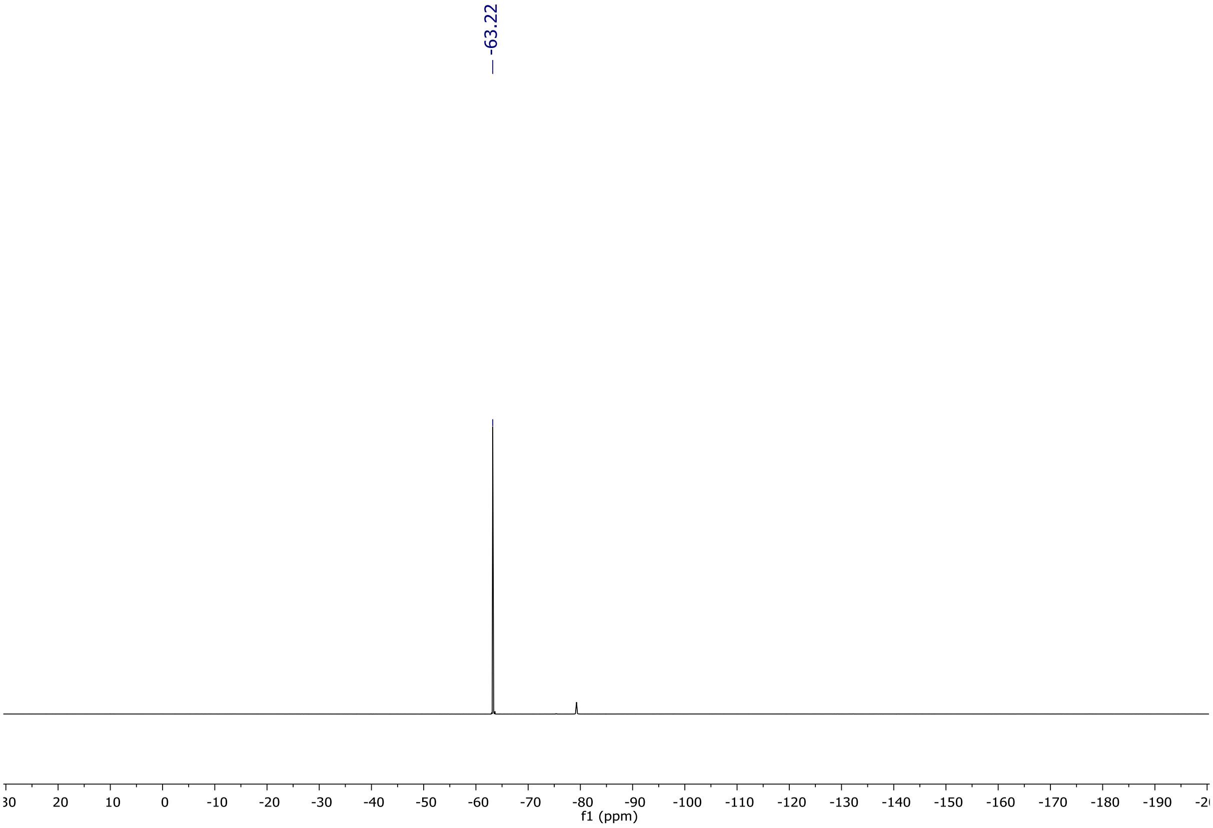

^{1}H NMR

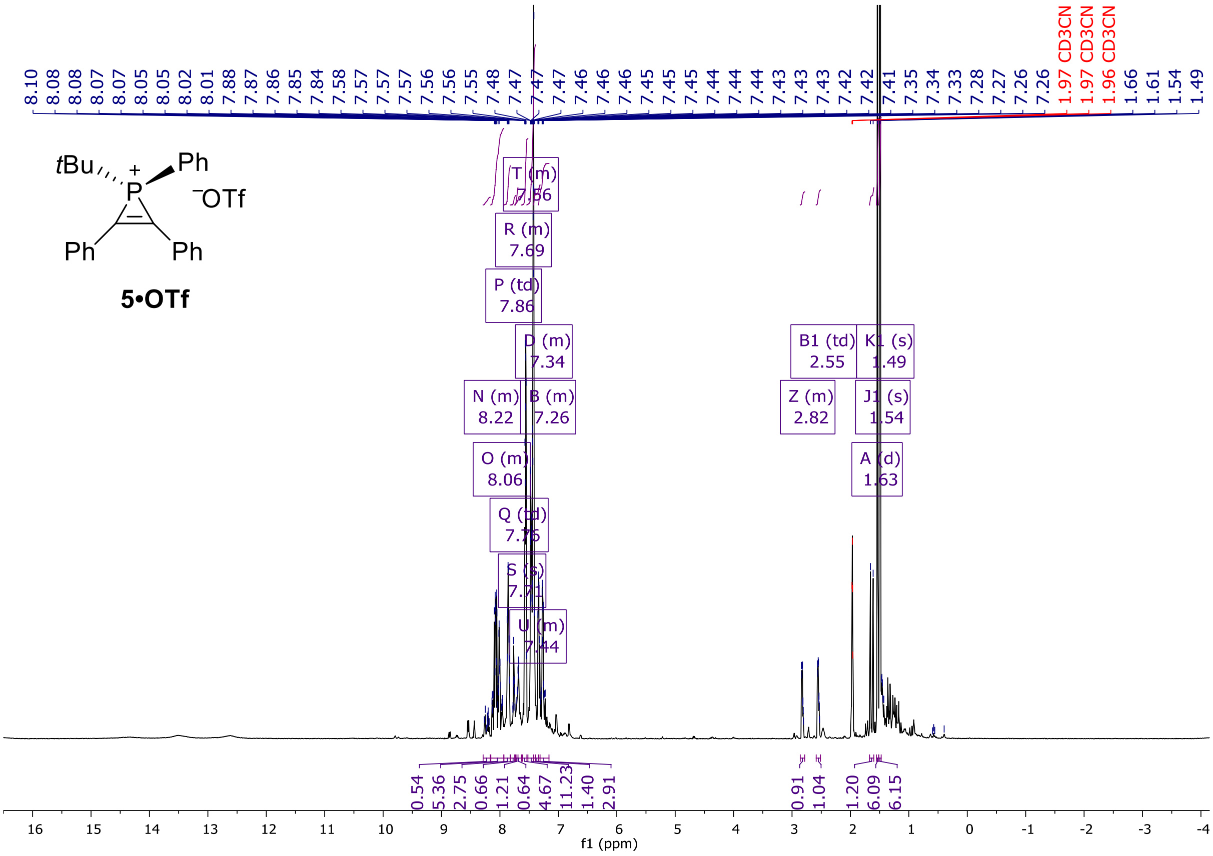

$^{31}P\{^{1}H\}$ NMR

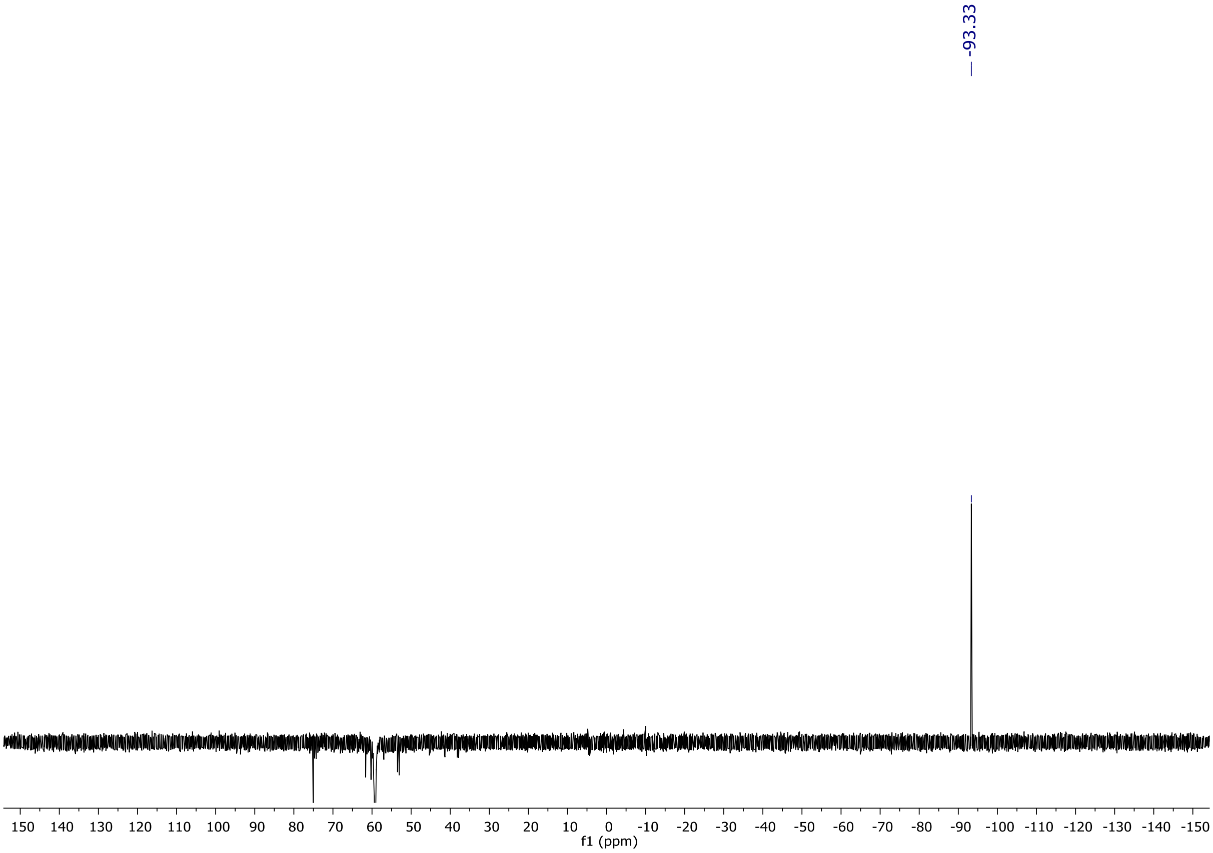

$^{13}C\{^{1}H\}$ NMR

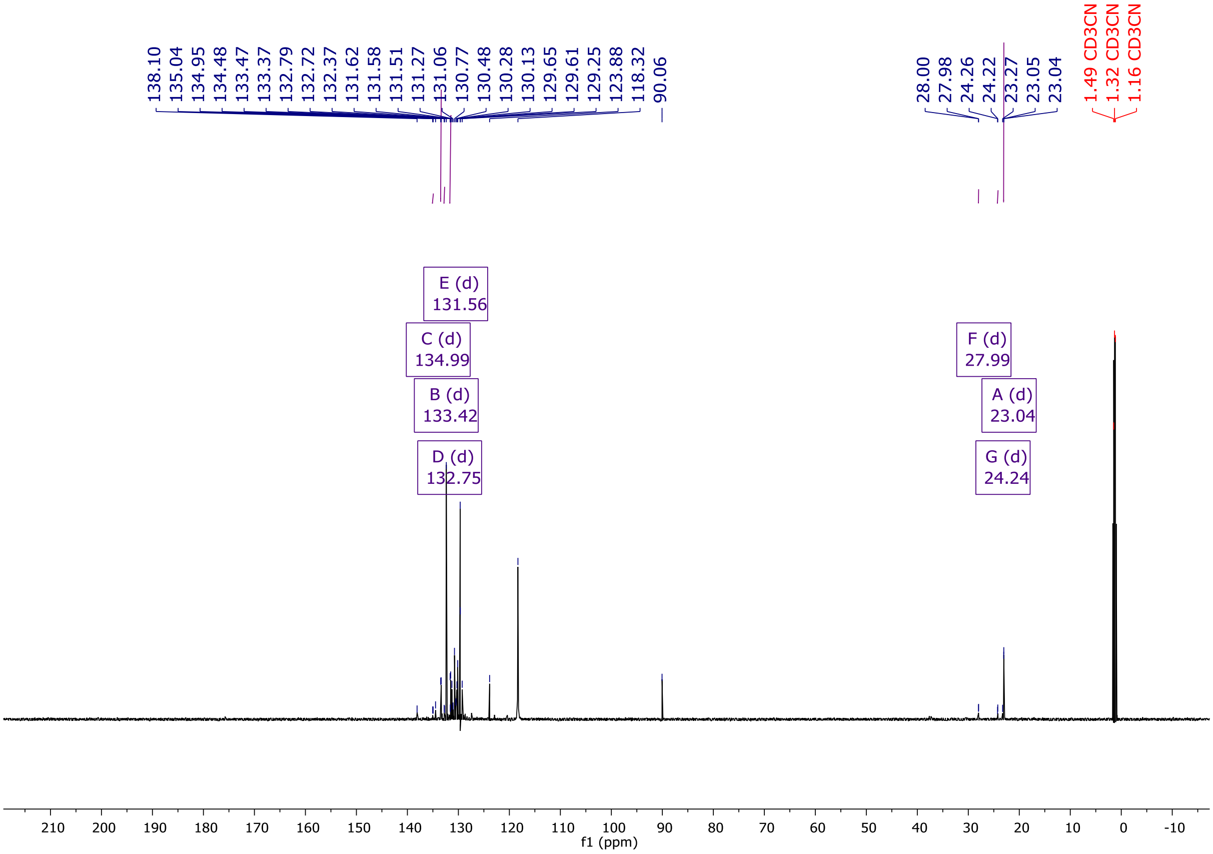

^{19}F NMR

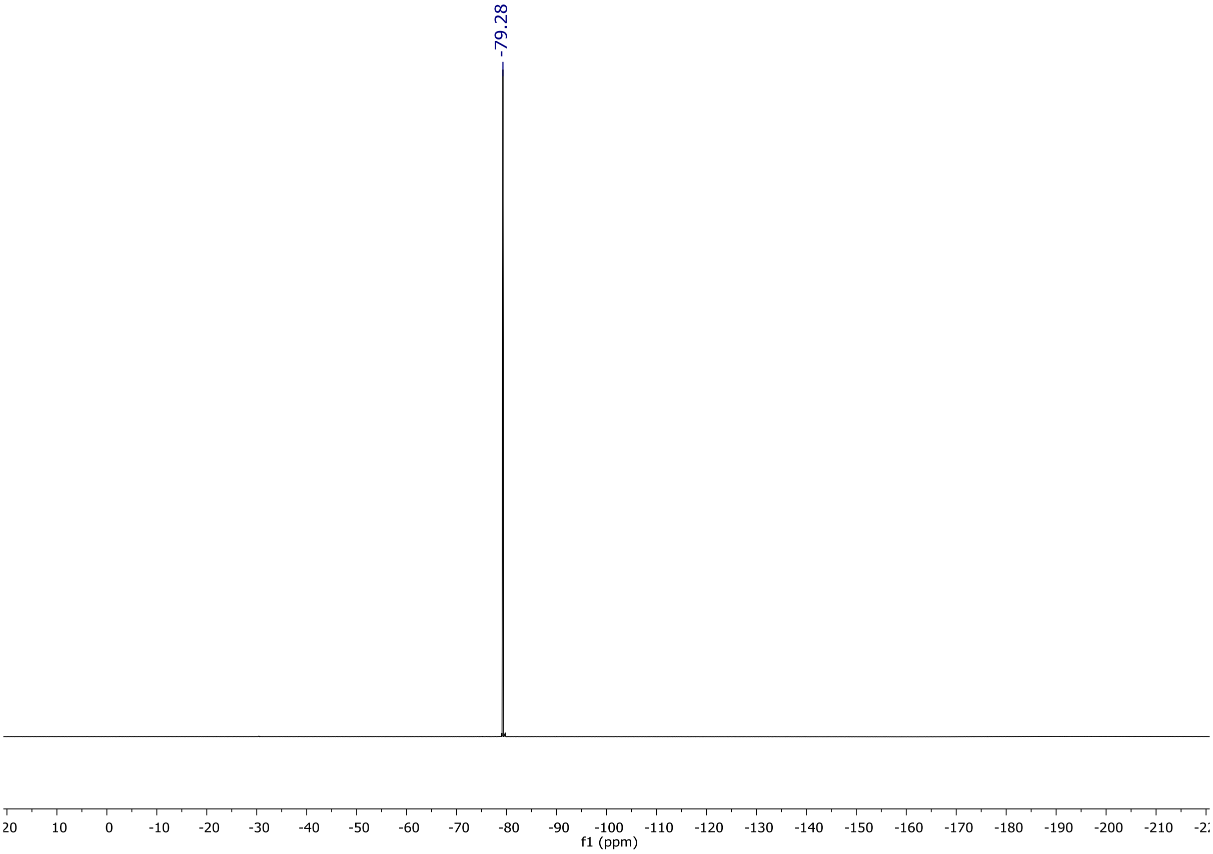

^1H NMR

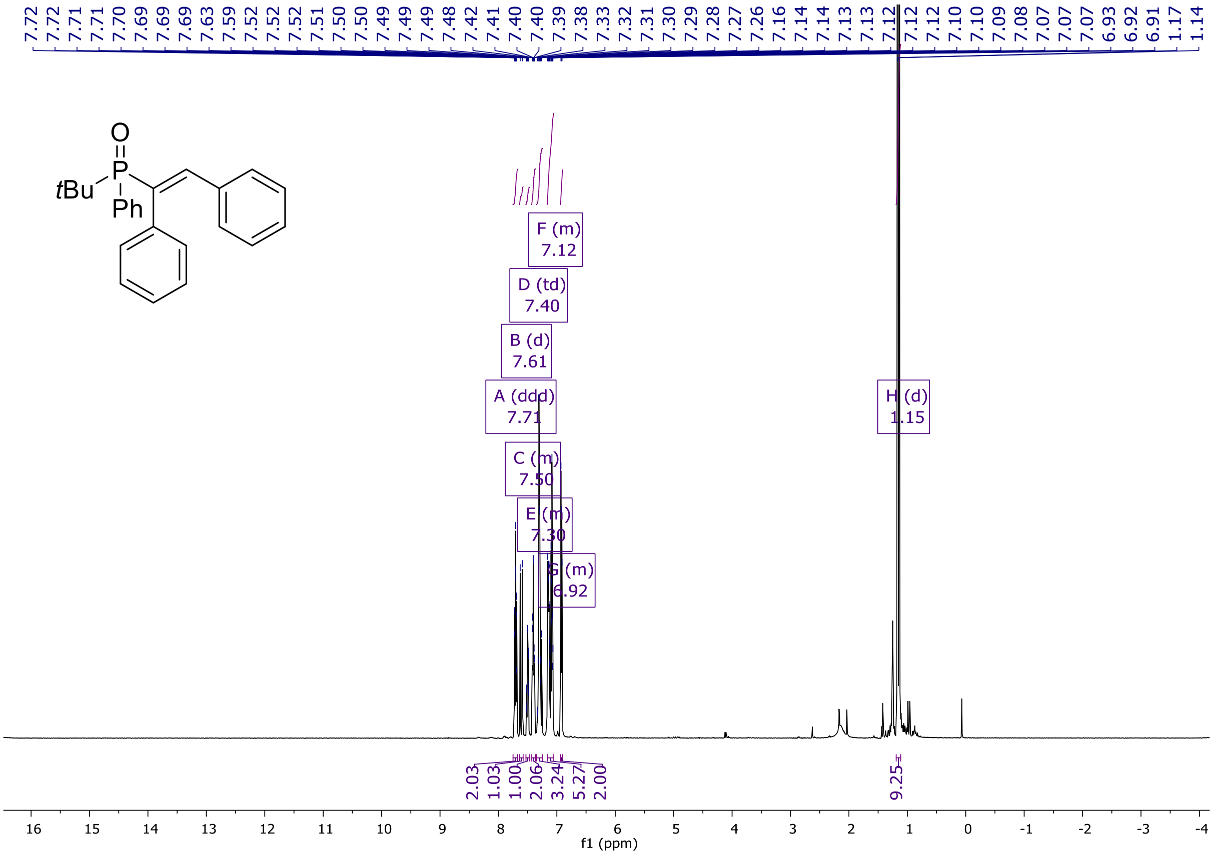

$^{13}C\{^1H\}$ NMR

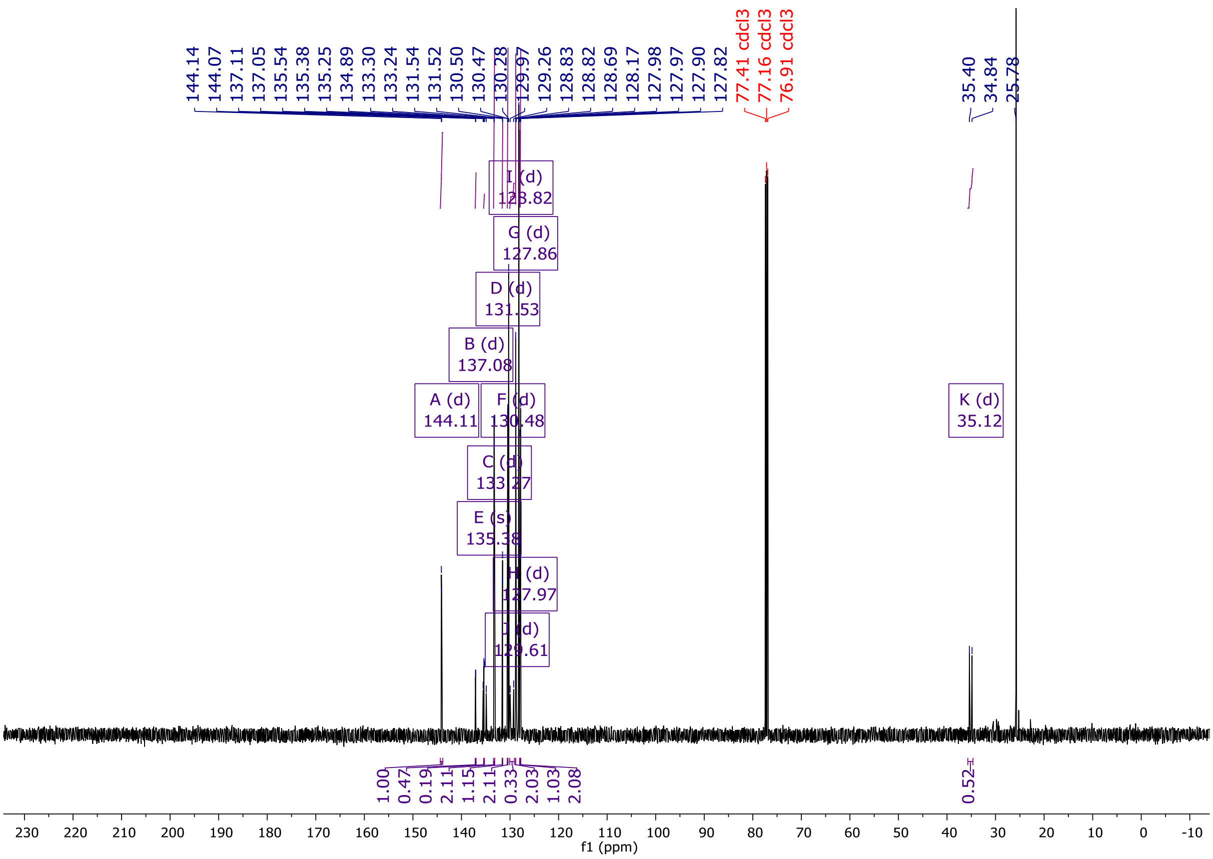

$^{31}P\{^{1}H\}$ NMR

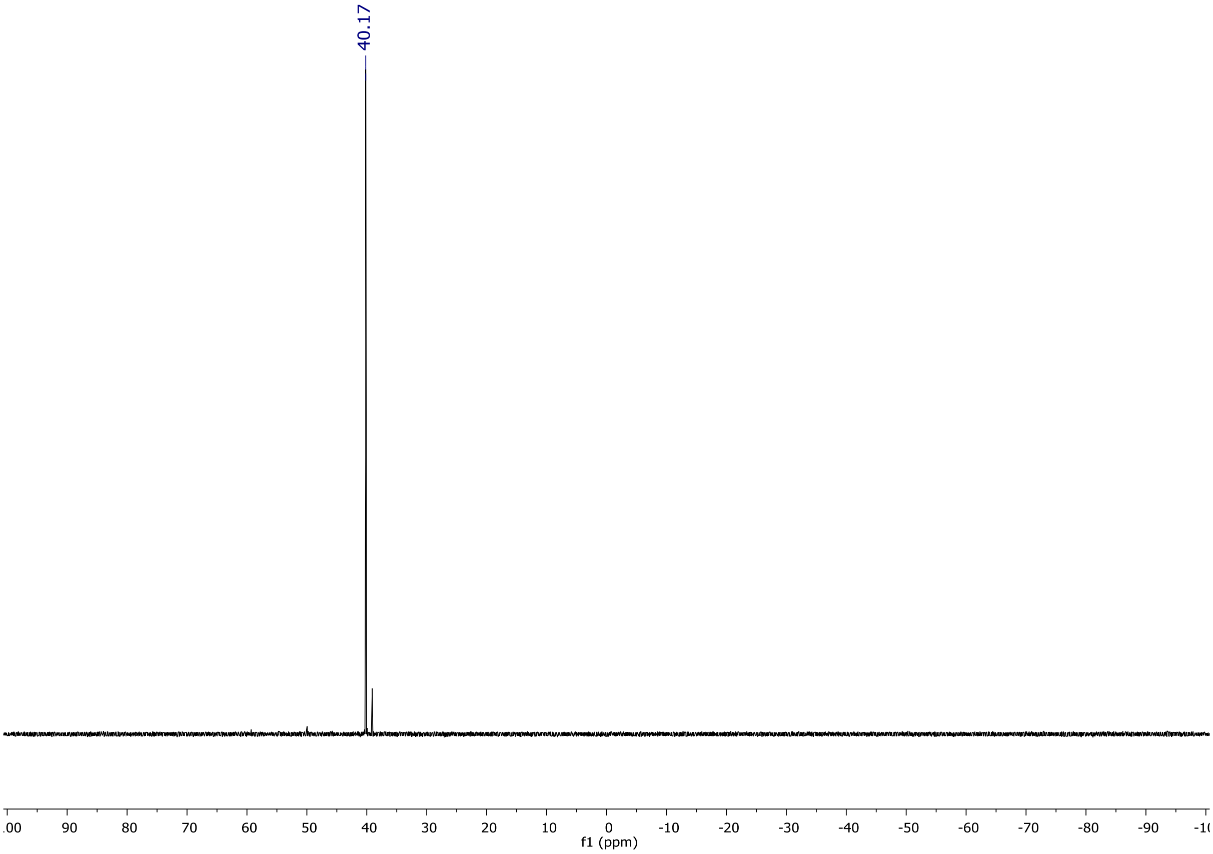

^{1}H NMR

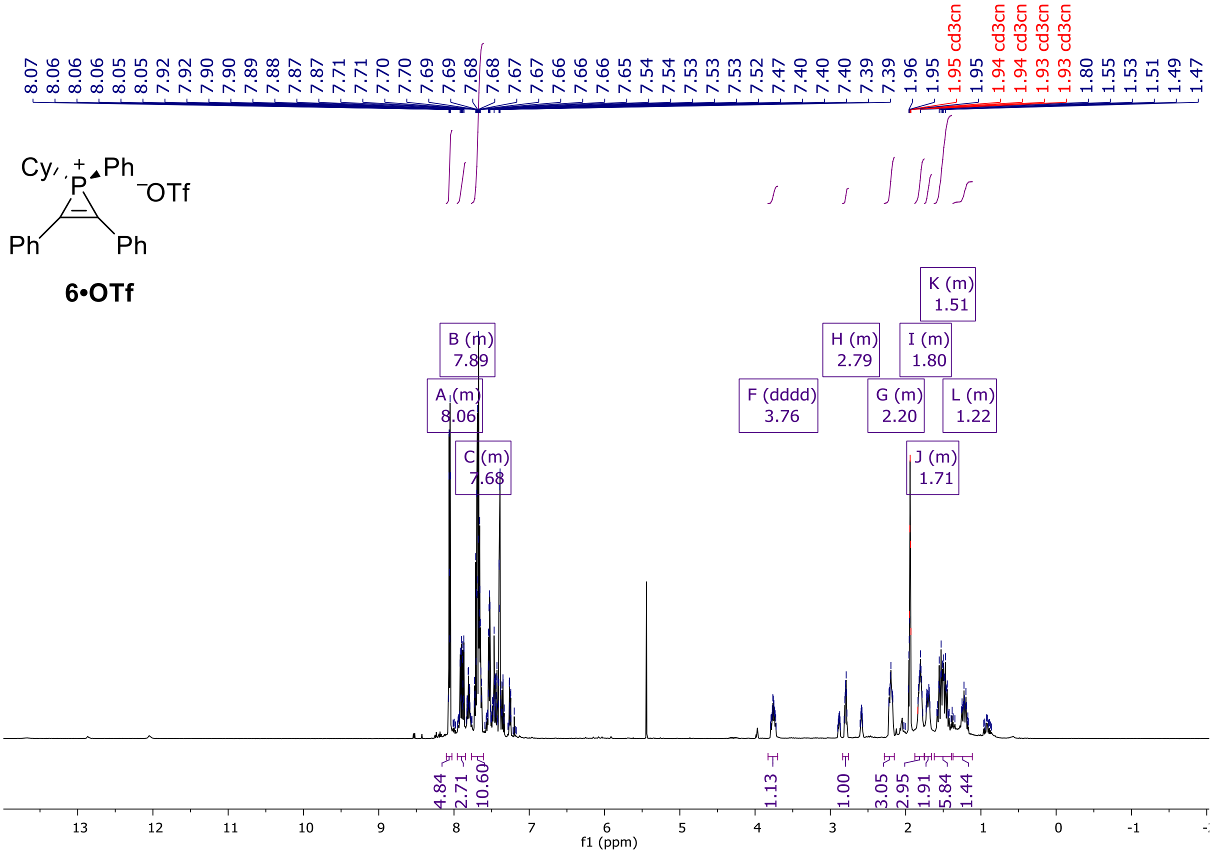

6•OTf

$^{31}P\{^{1}H\}$ NMR

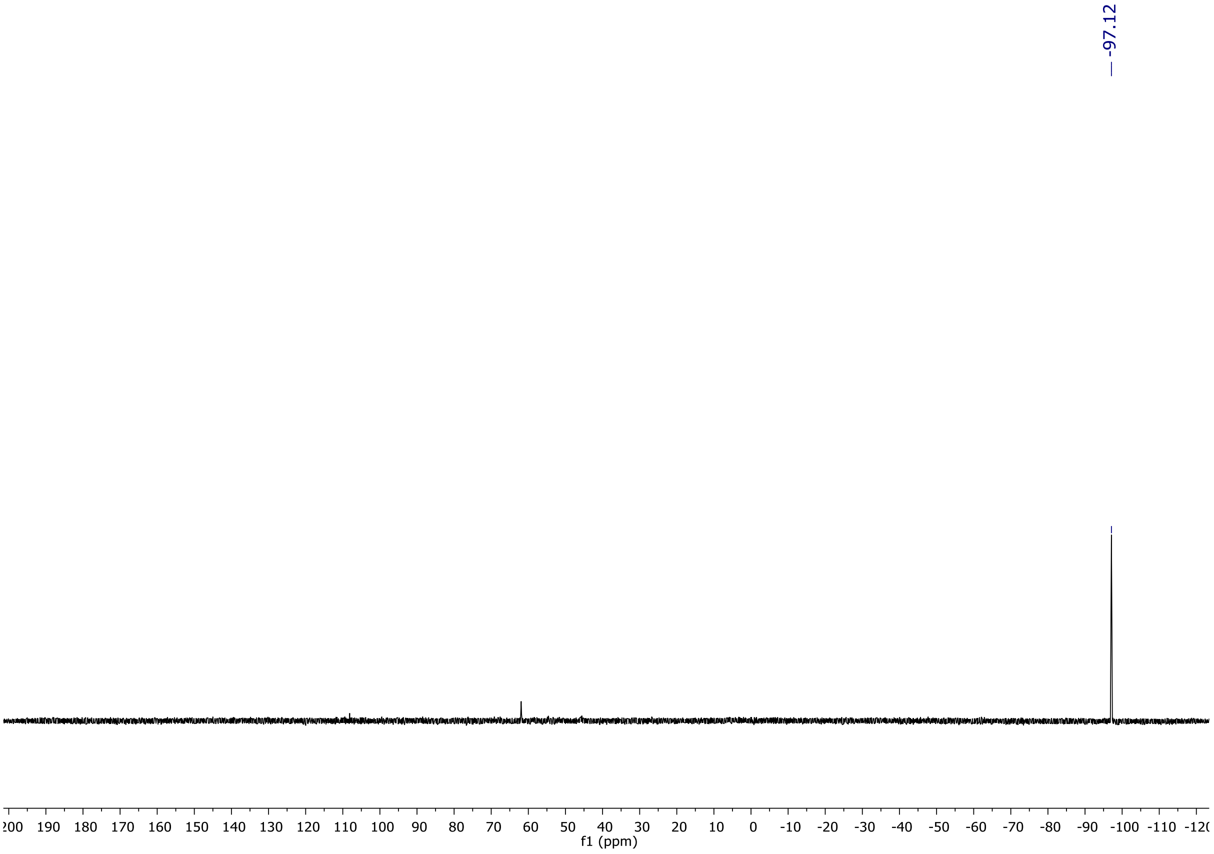

$^{13}C\{^{1}H\}$ NMR

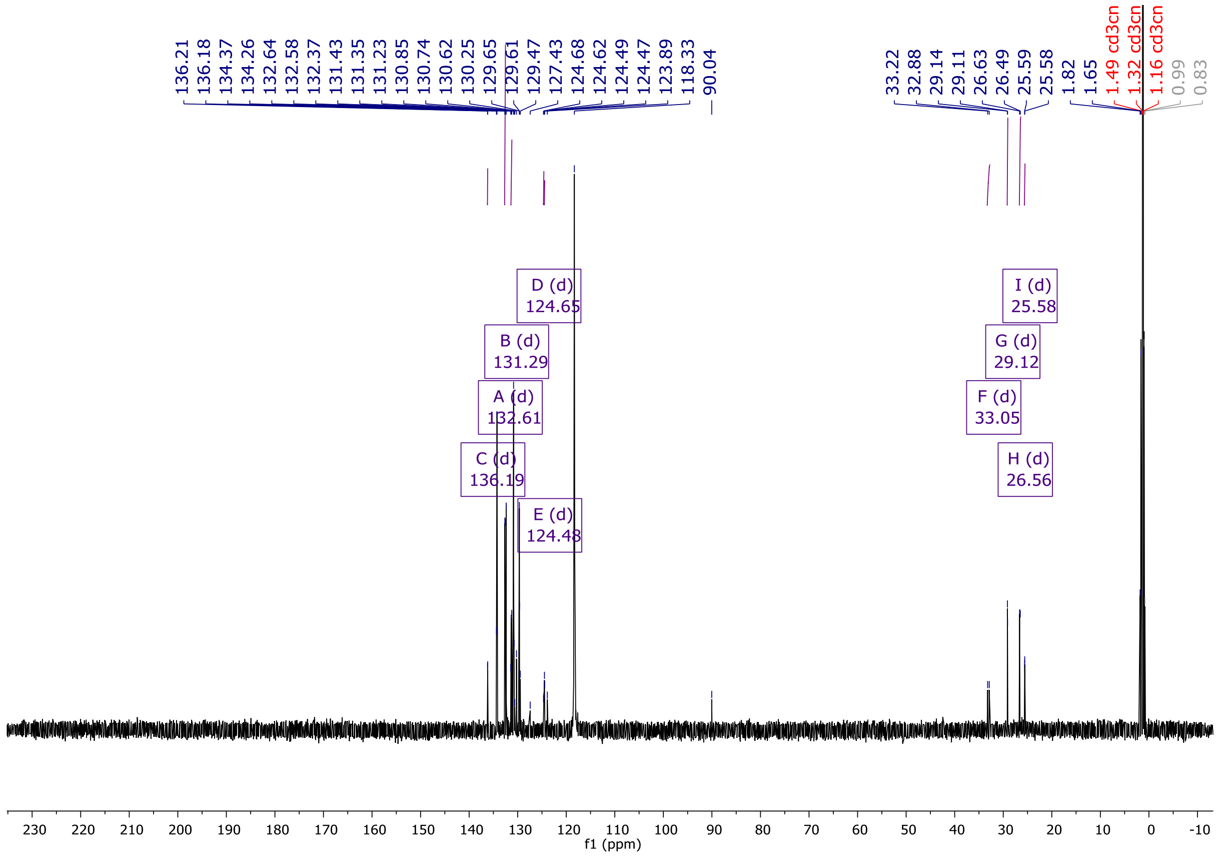

^{19}F NMR

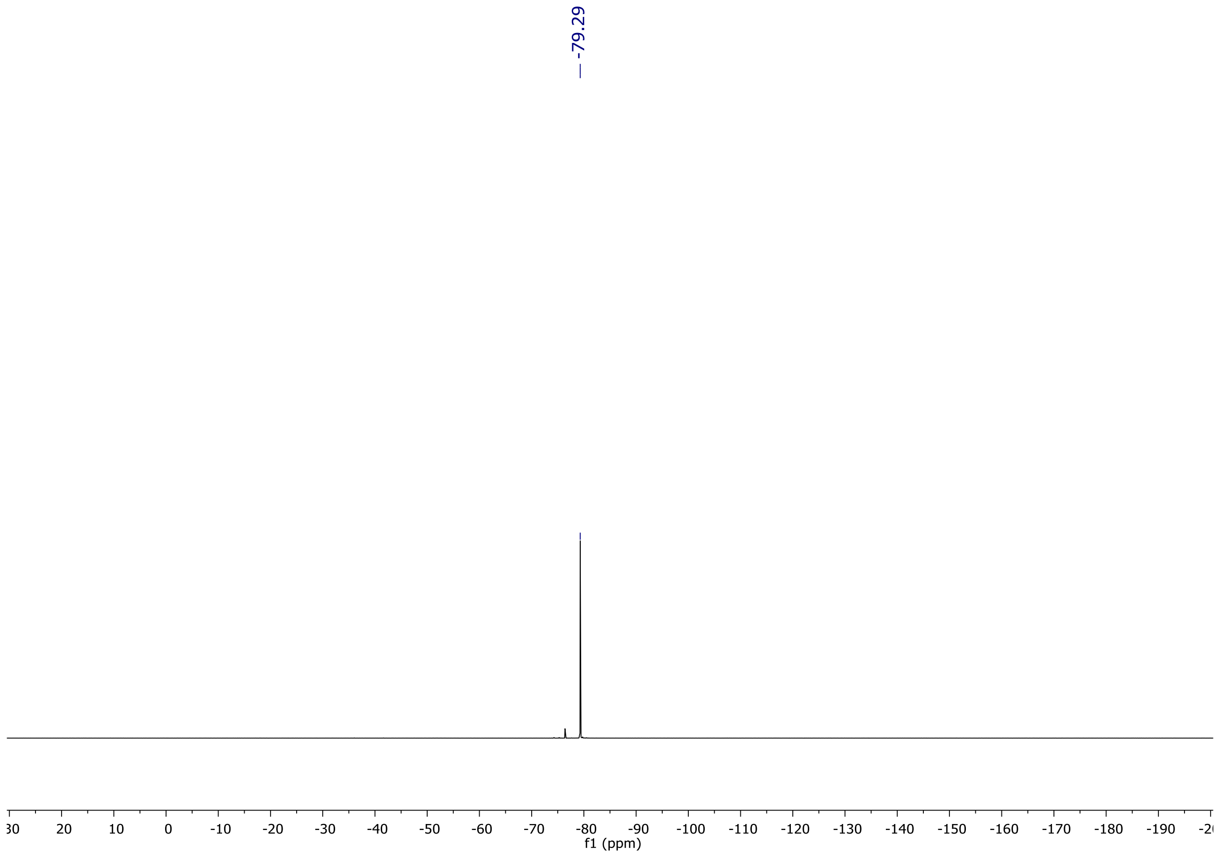

^{1}H NMR

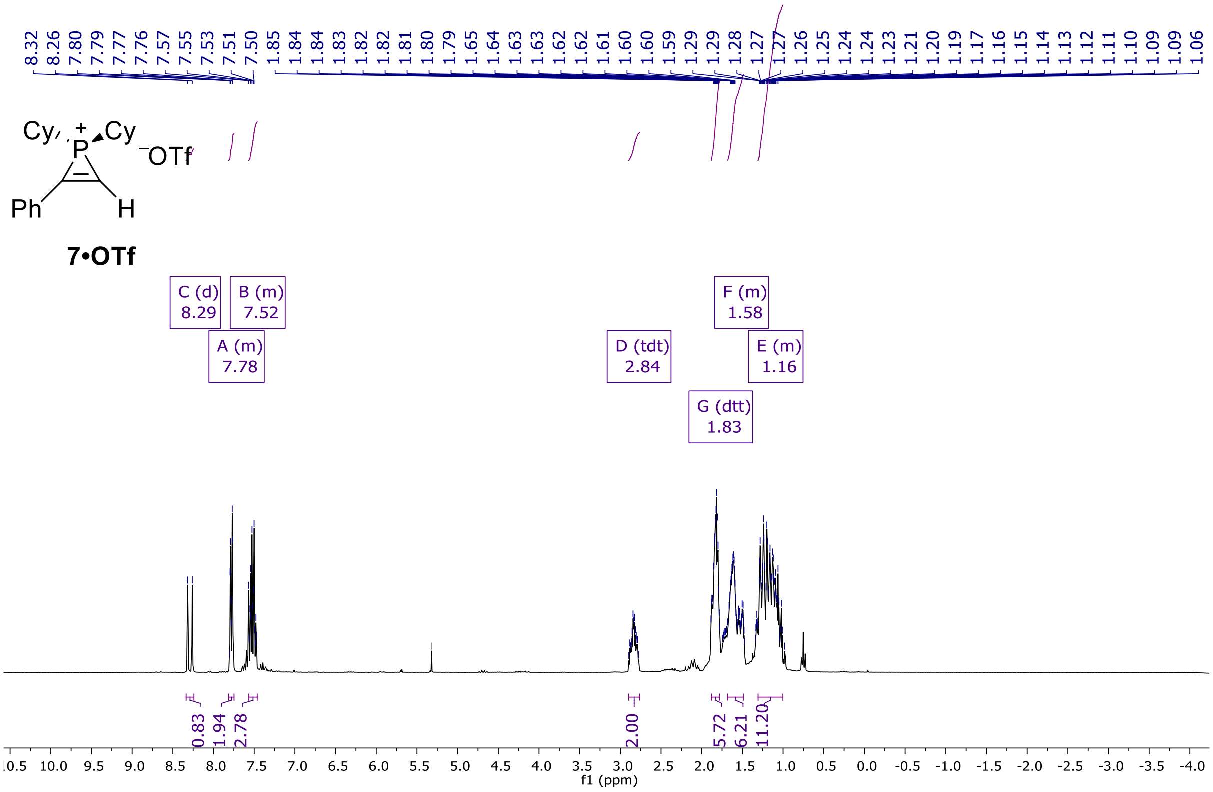

^{13}C{^{1}H} NMR

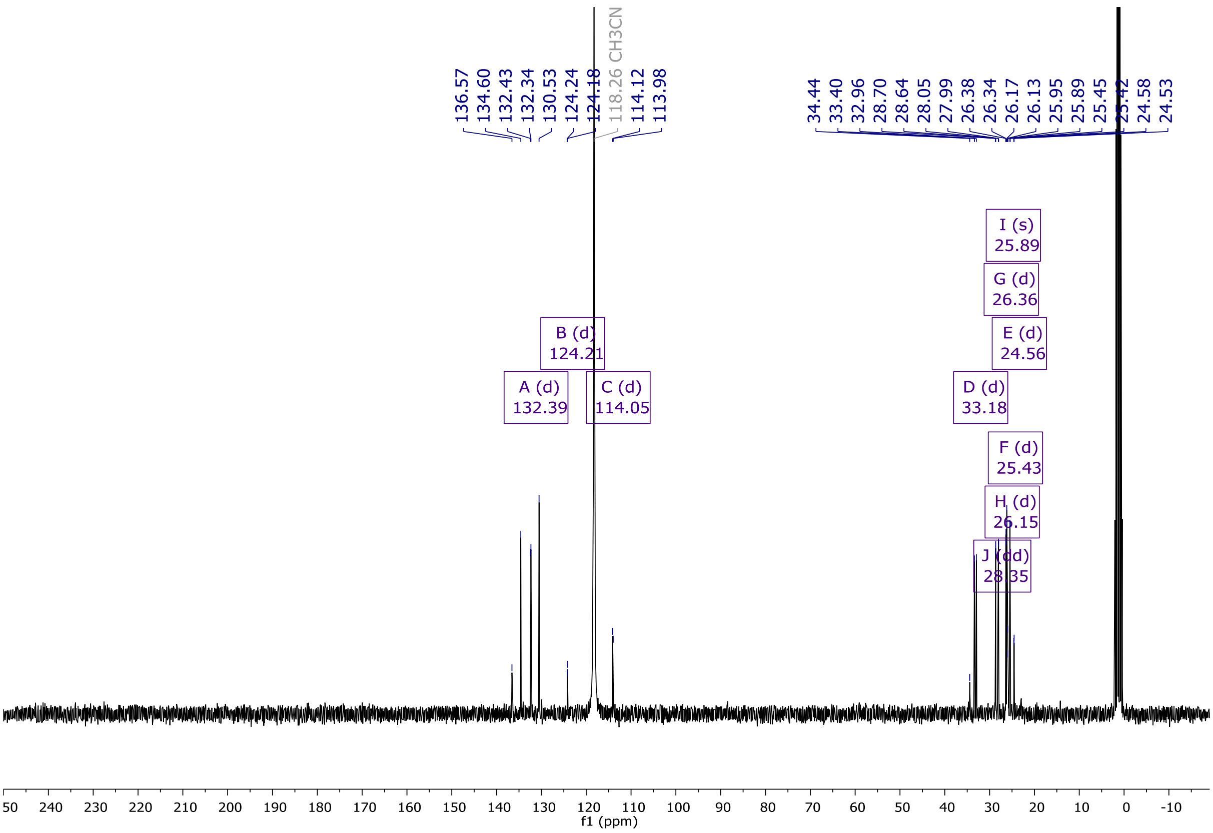

$^{31}P\{^{1}H\}$ NMR

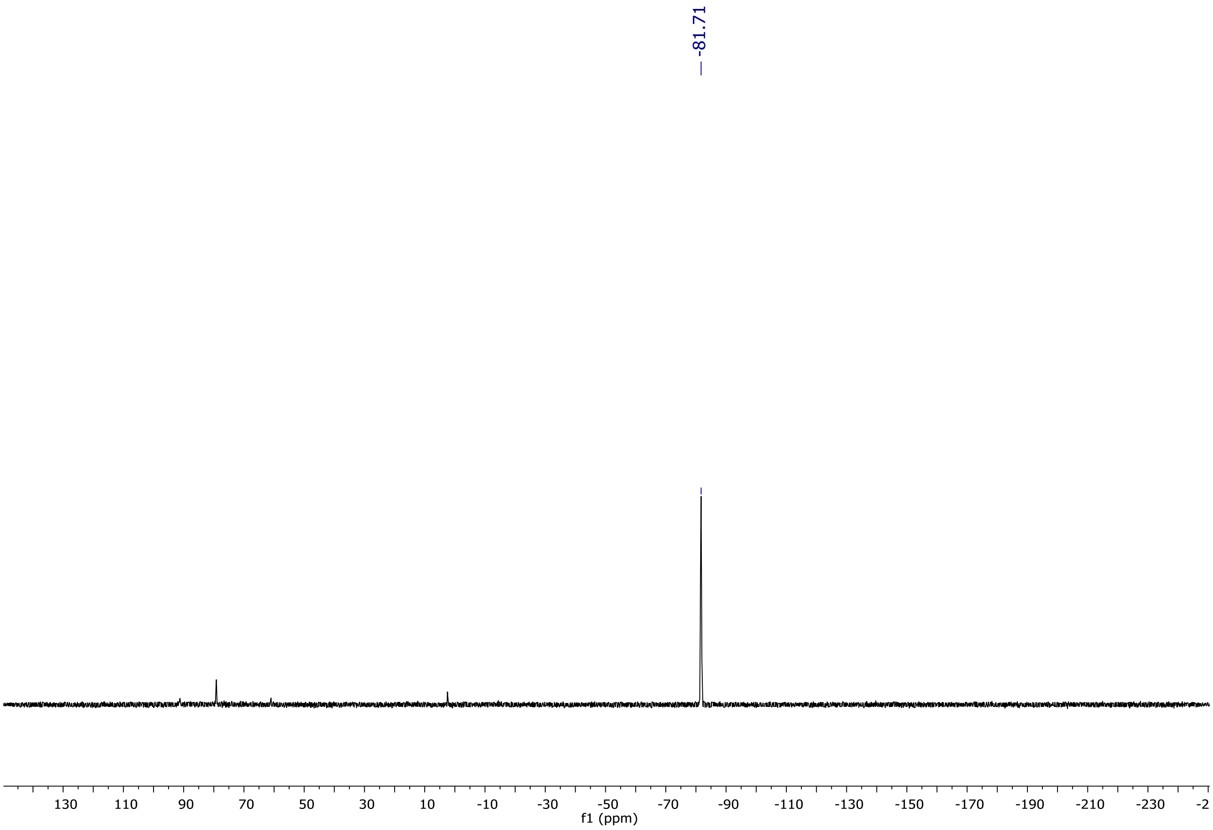

^{19}F NMR

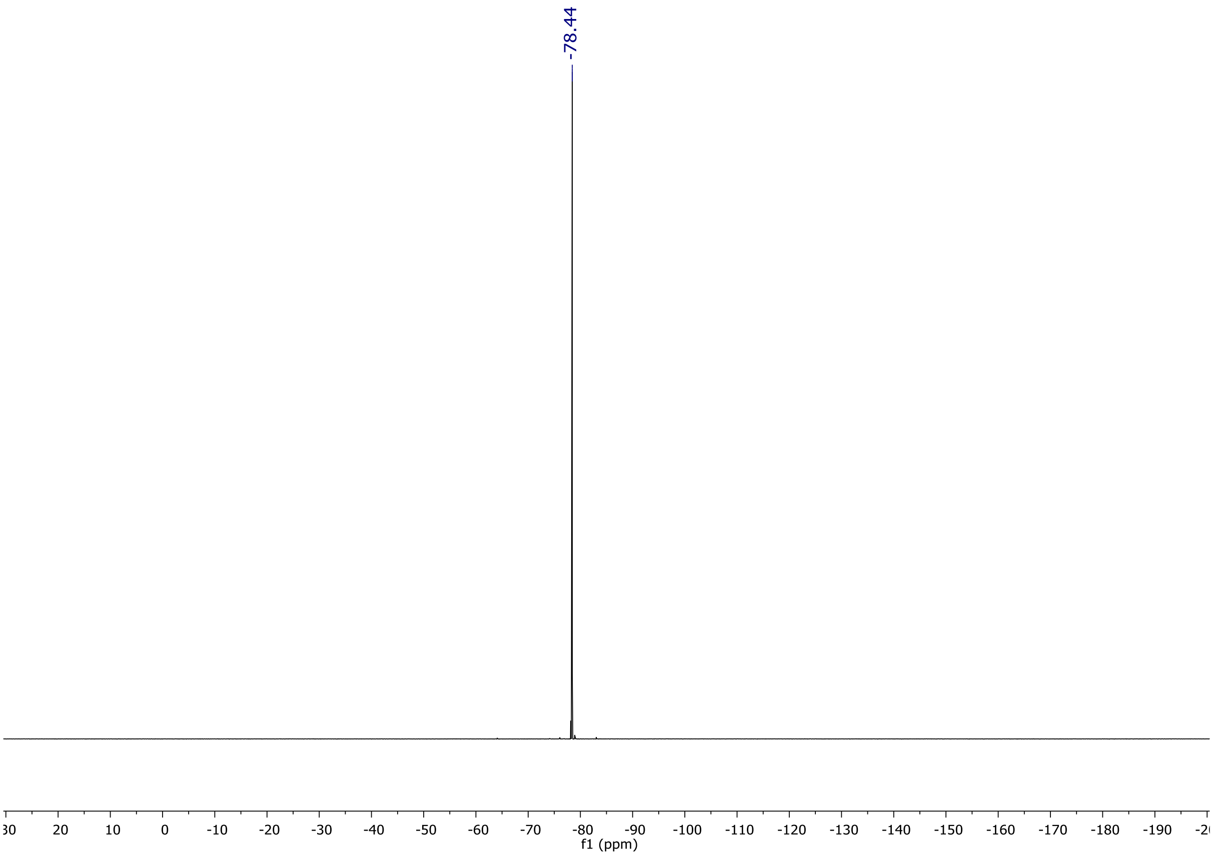

^{1}H NMR

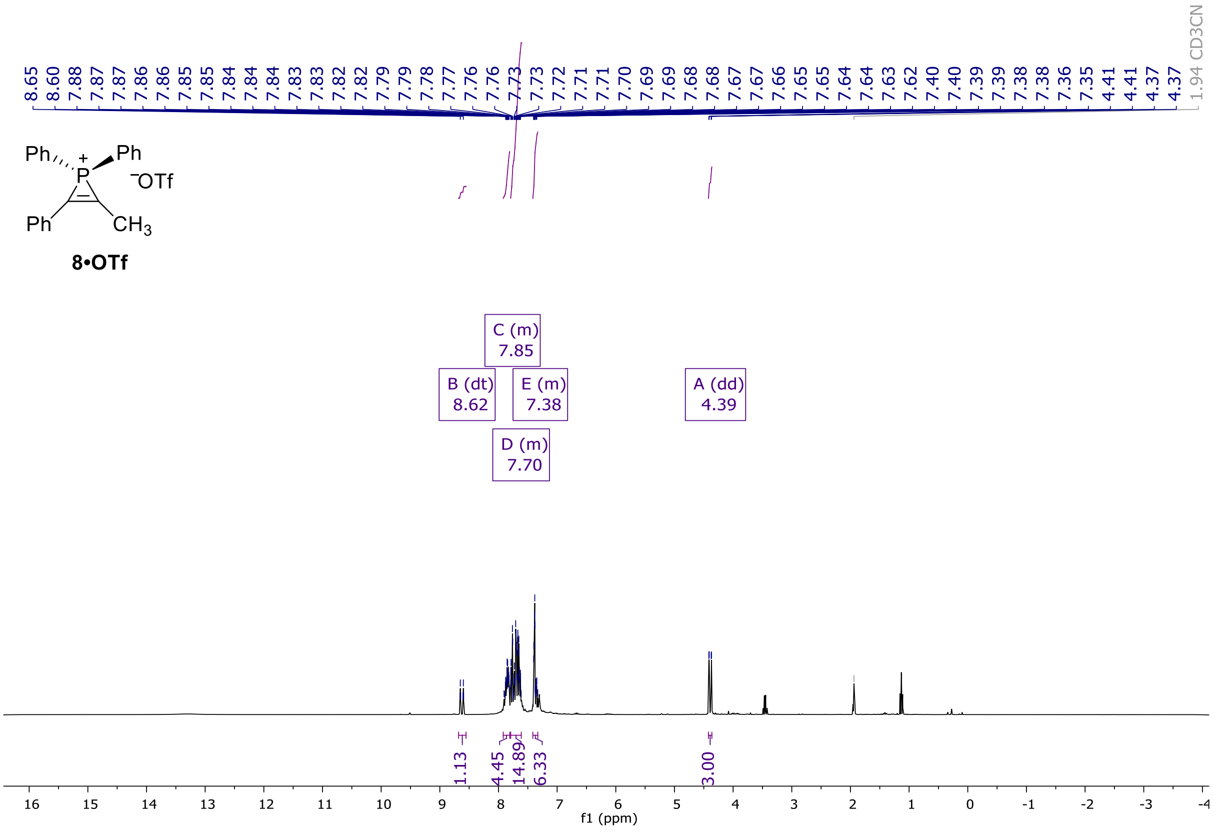

^{13}C{^{1}H} NMR

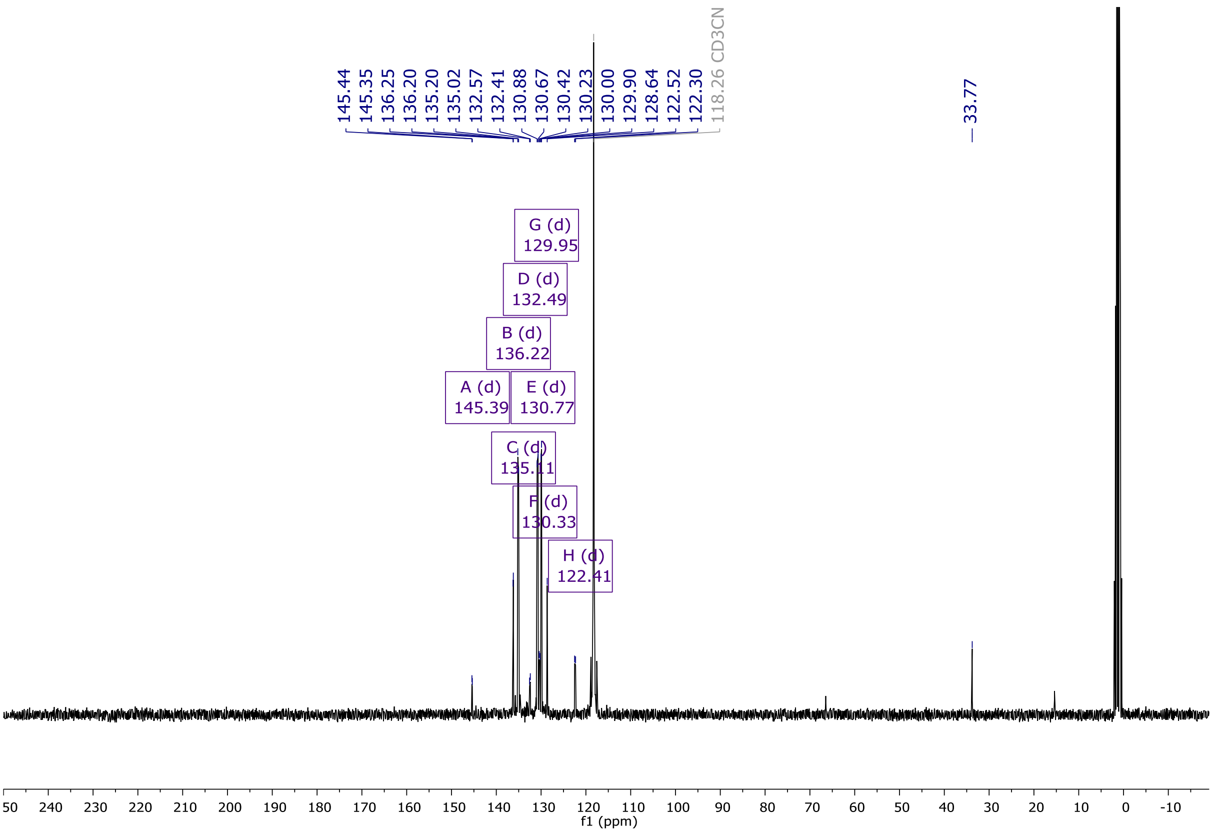

$^{31}P\{^1H\}$ NMR with PPh_3 (-5.8 ppm)

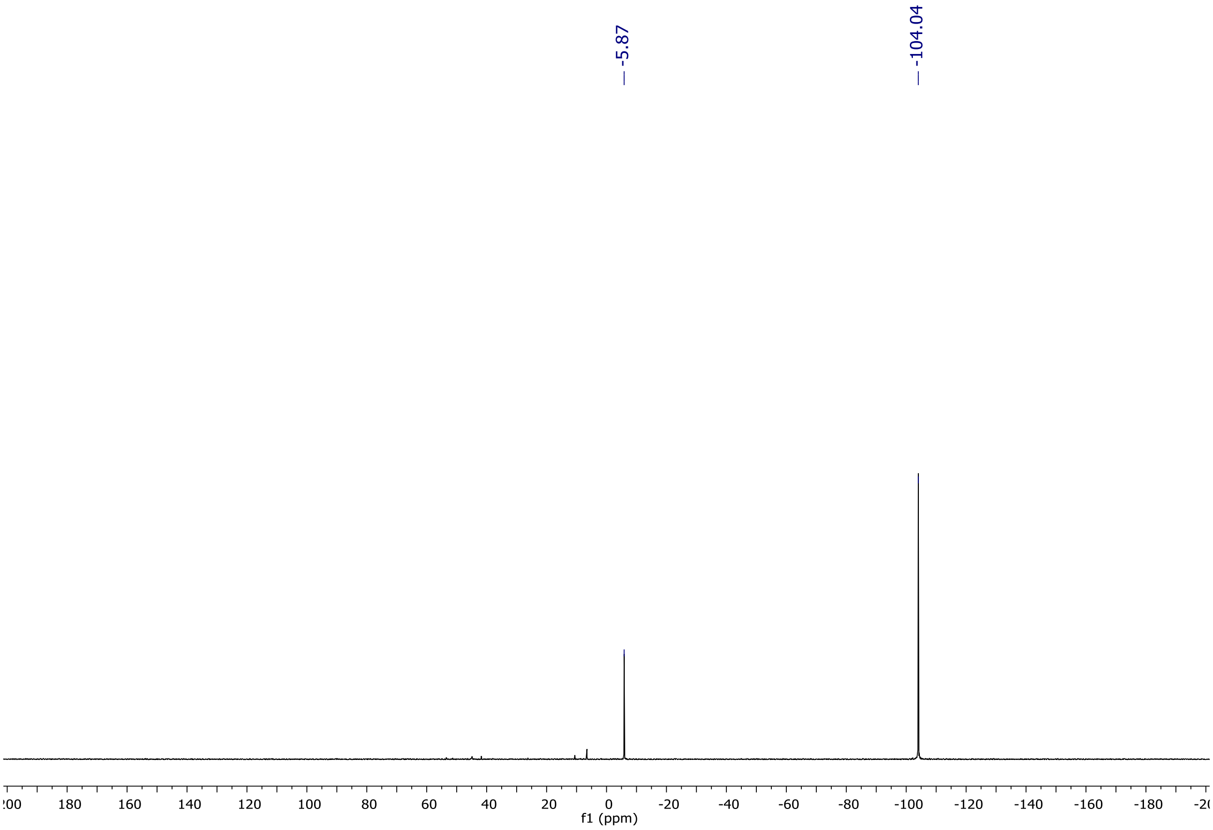

$^{19}F\{^1H\}$ NMR

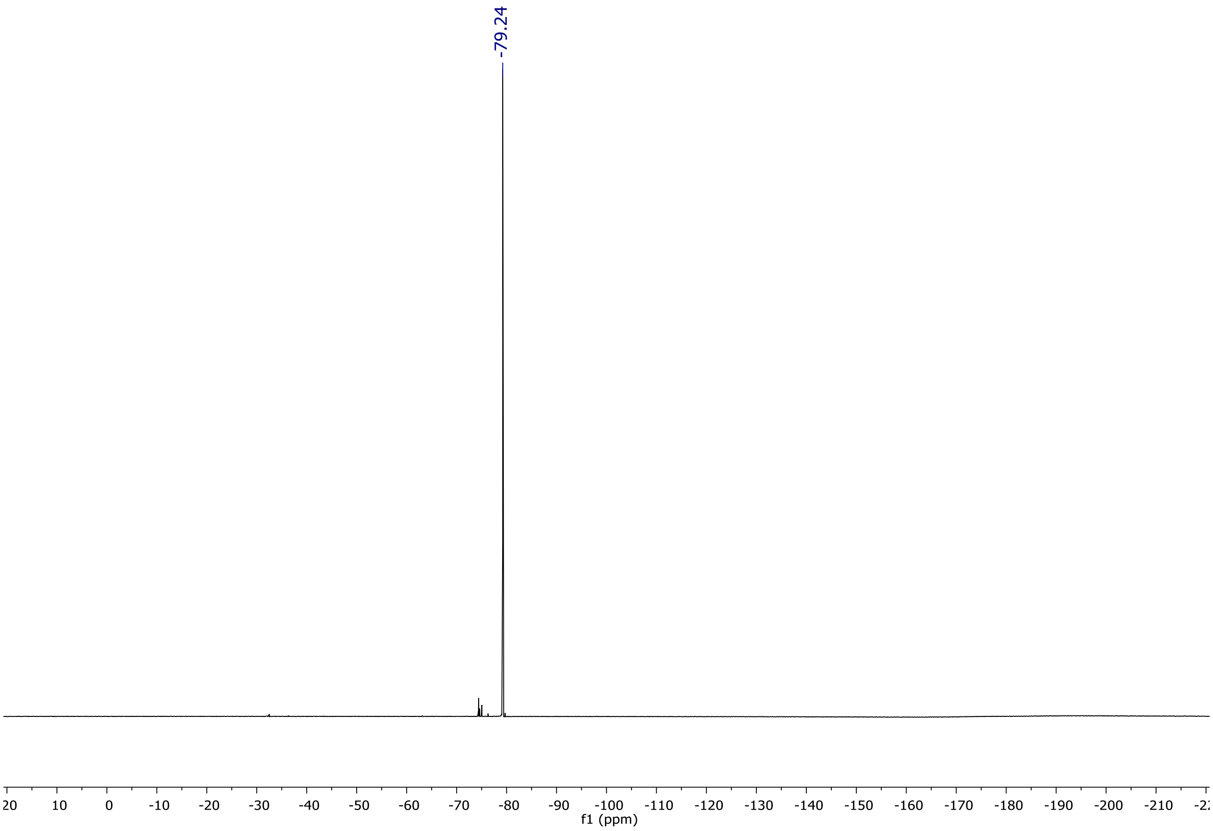

^{1}H NMR

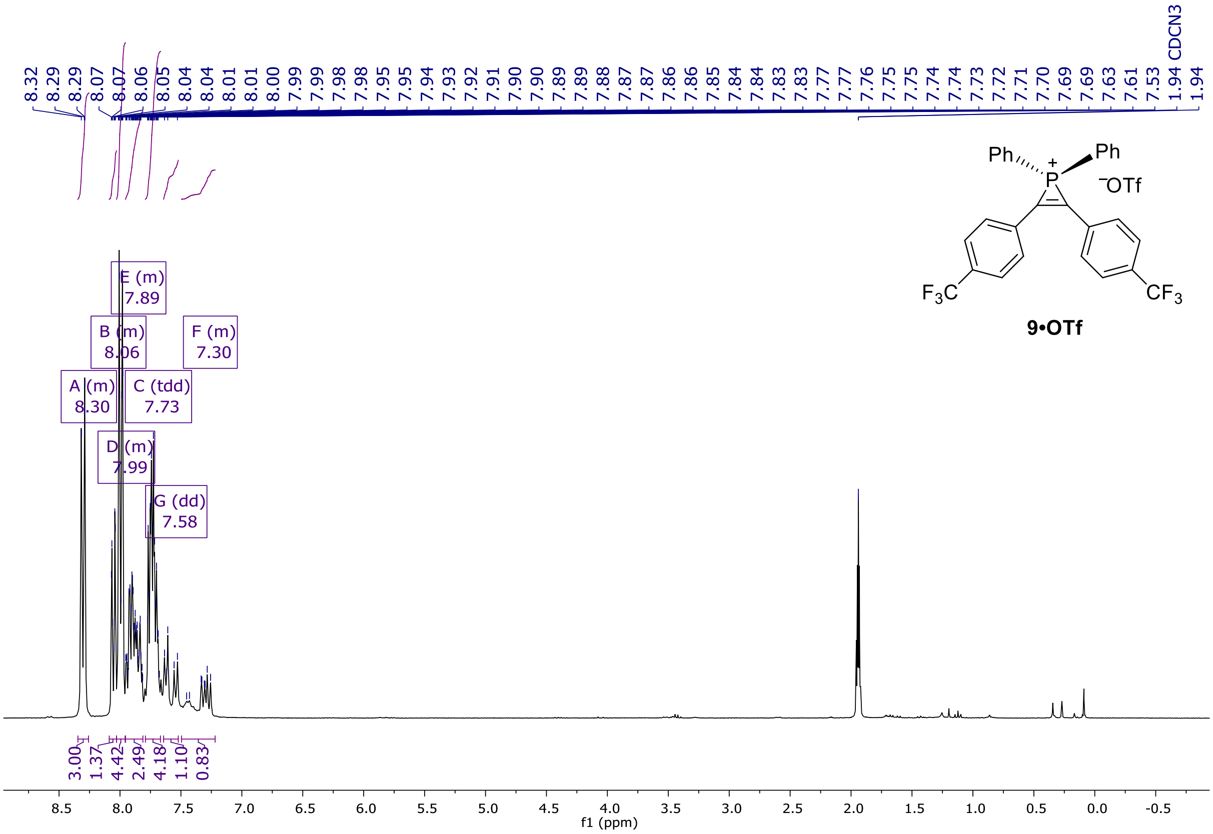

$^{13}C\{^{1}H\}$ NMR

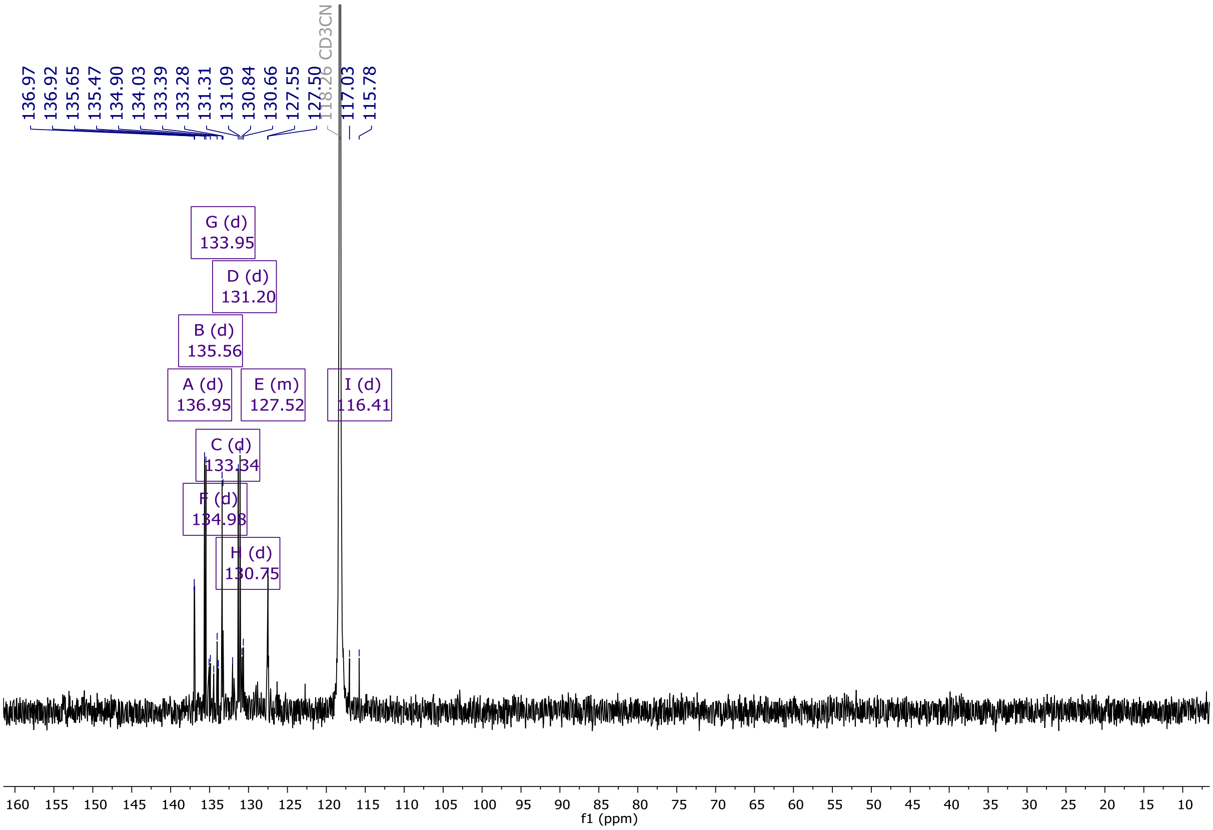

$^{31}P\{^1H\}$ NMR

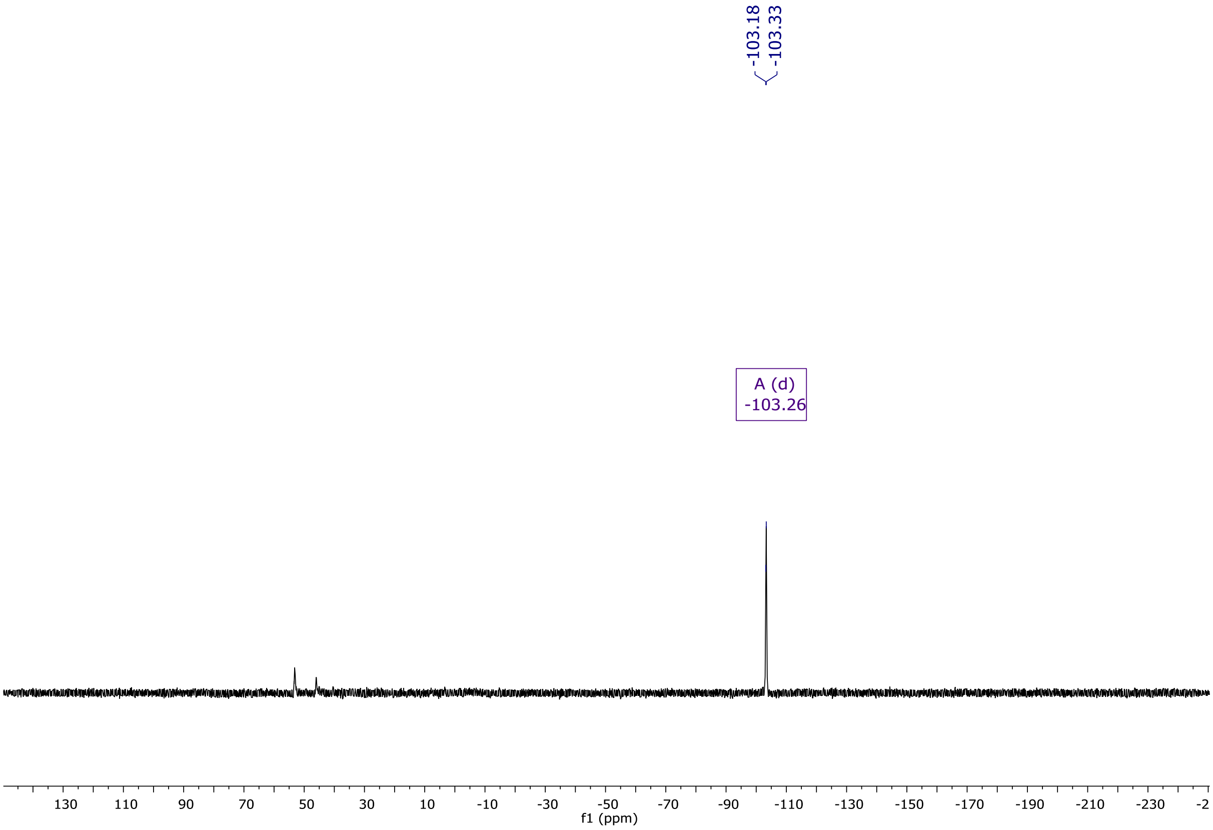

$^{19}F\{^1H\}$ NMR

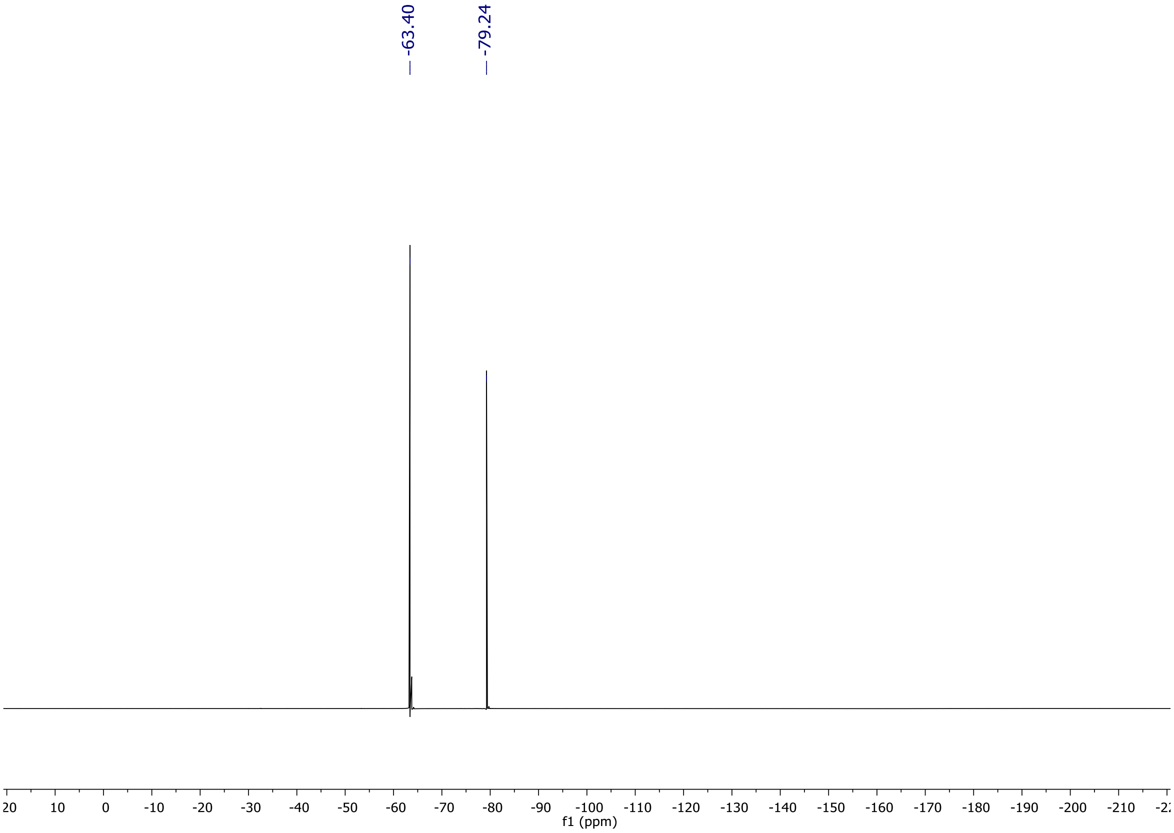

^{1}H NMR

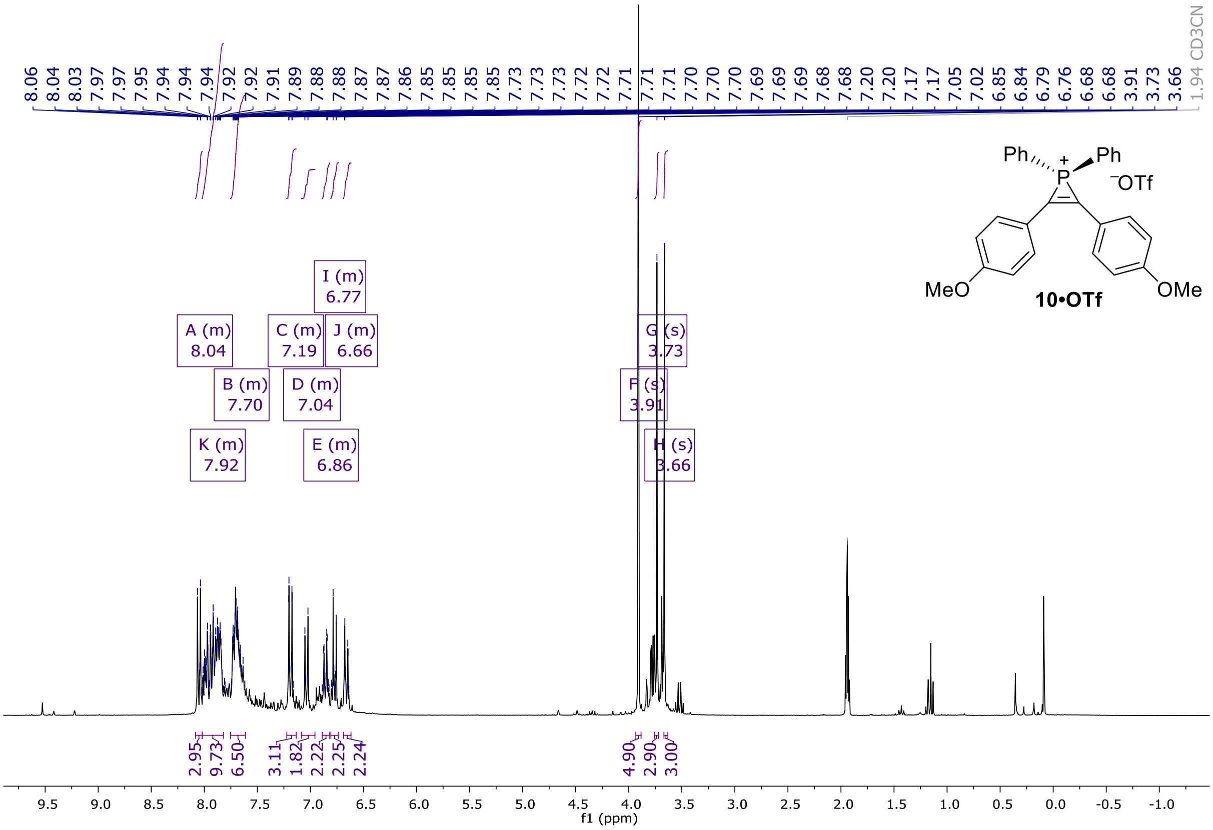

$^{13}C\{^1H\}$ NMR

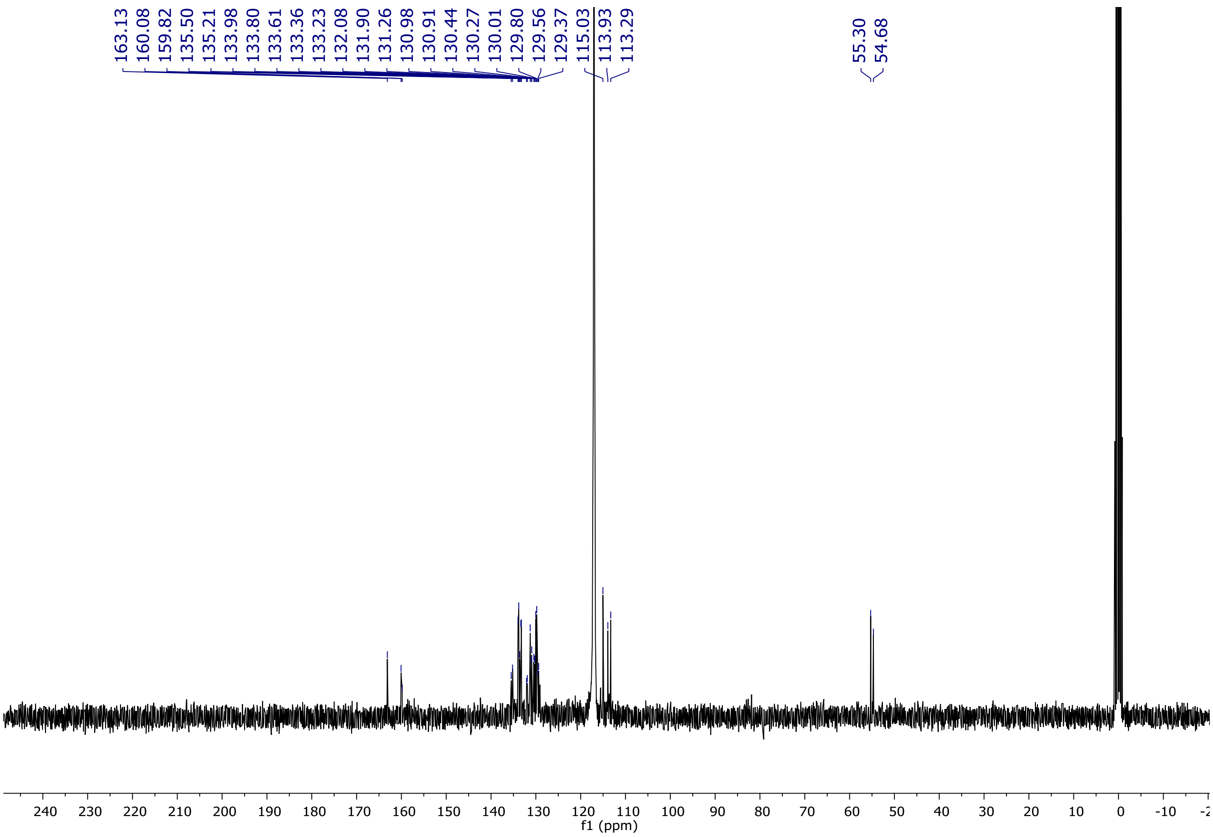

$^{31}P\{^1H\}$ NMR

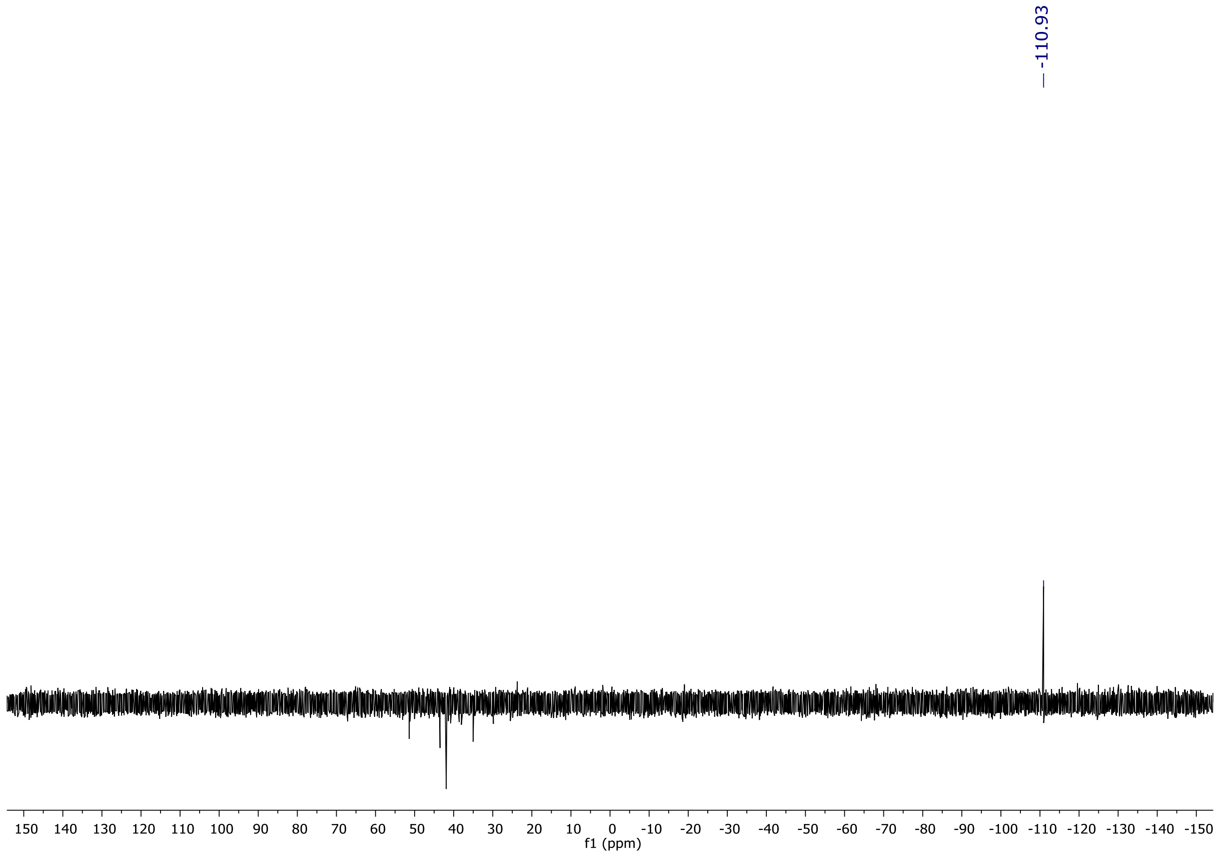

$^{19}F\{^1H\}$ NMR

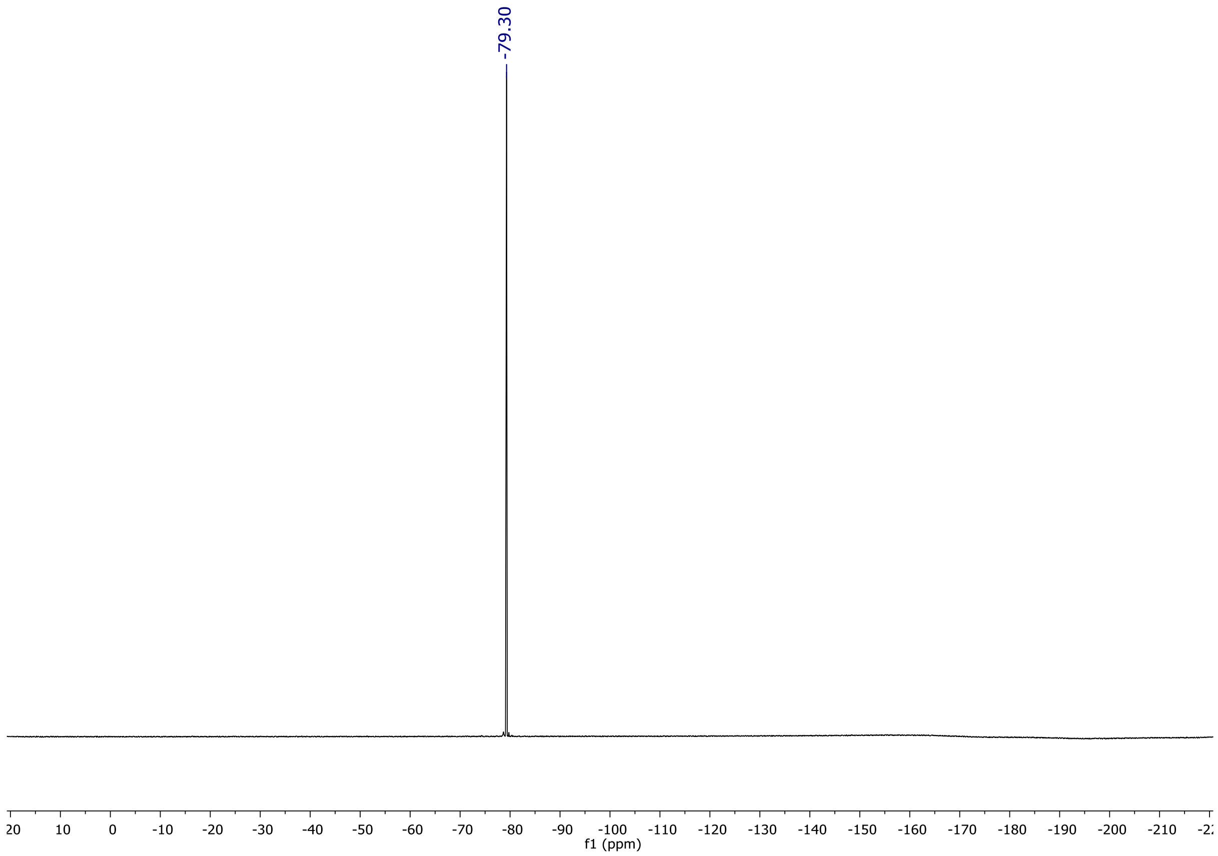

^{1}H NMR

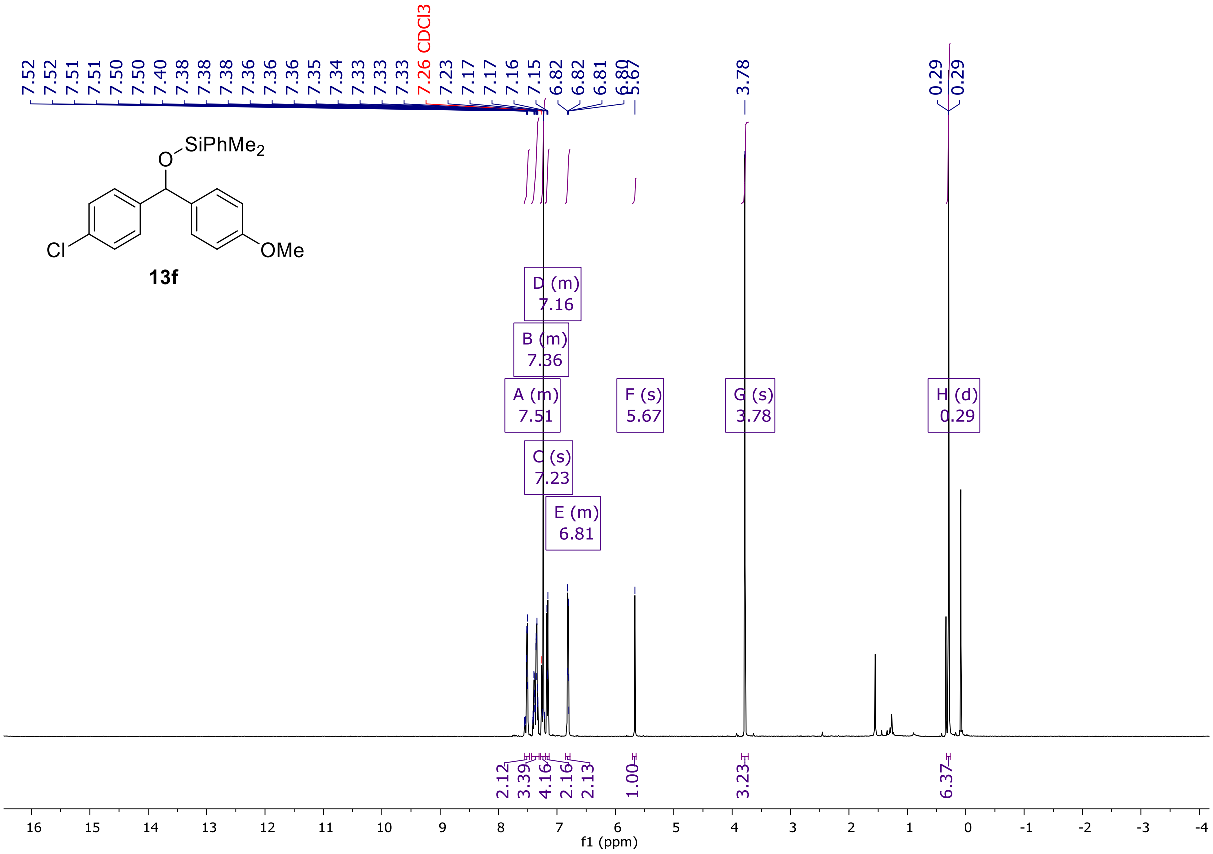

^{13}C{^{1}H} NMR

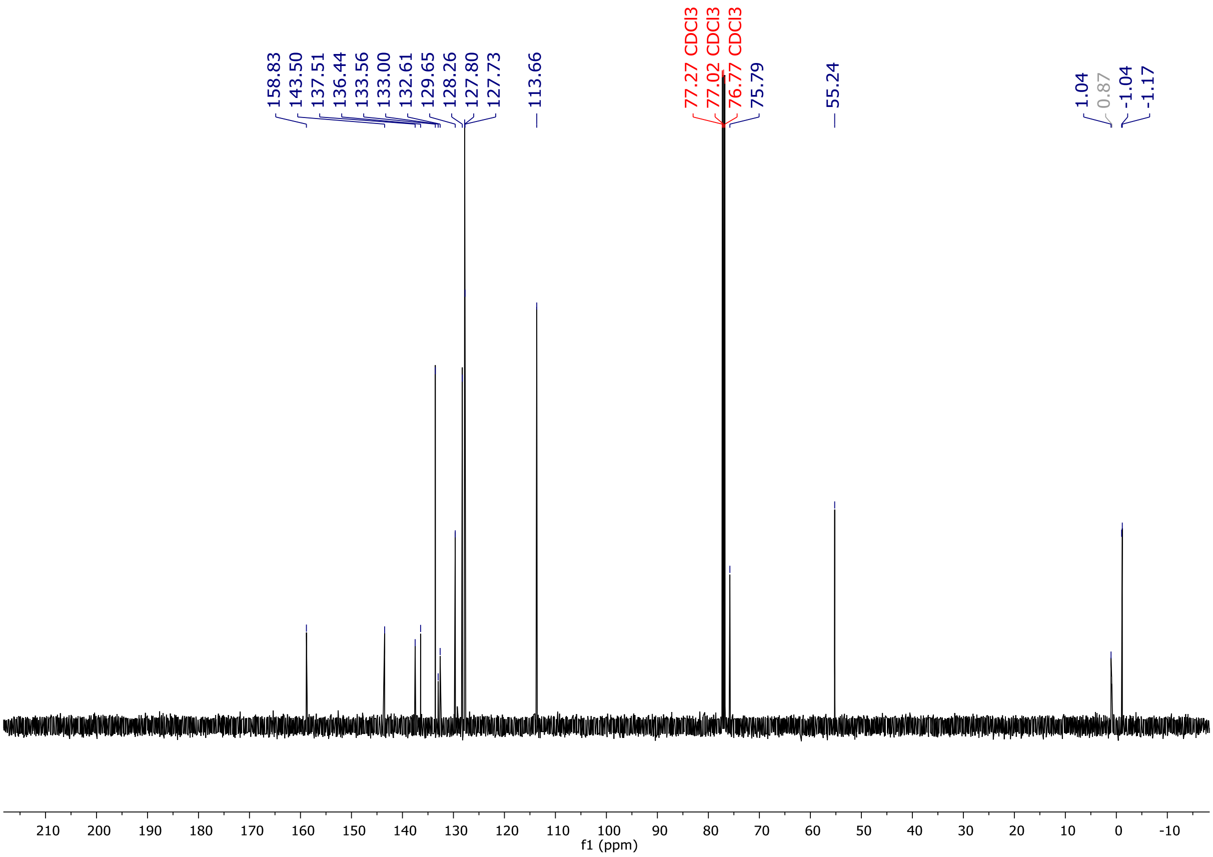

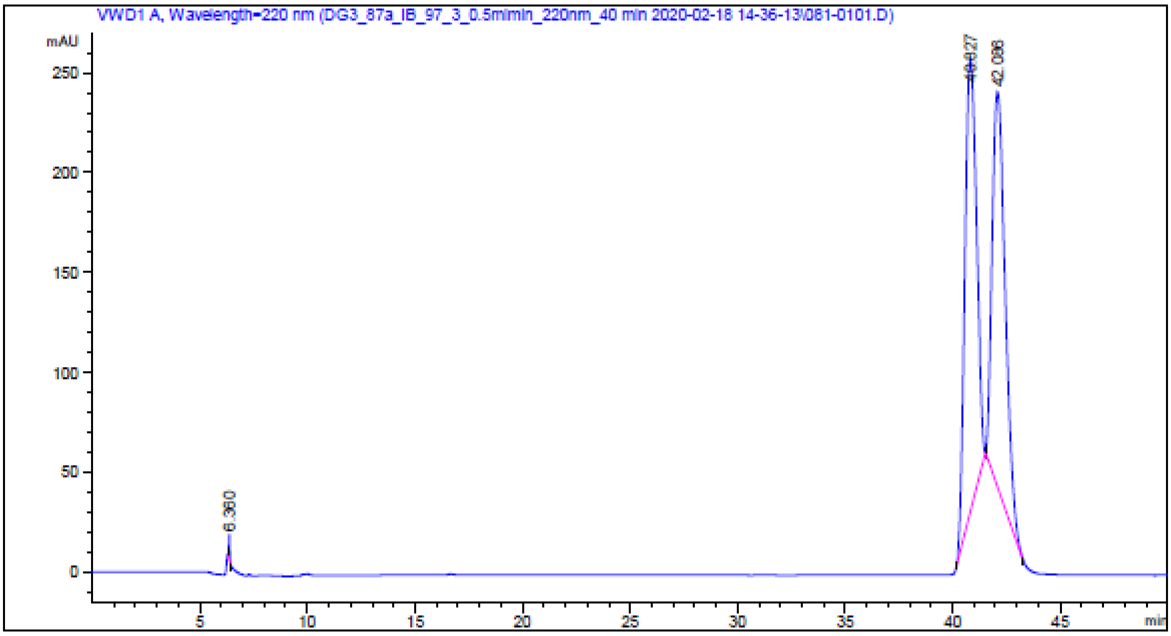

Peak #	RetTime [min]	Type	Width [min]	Area [mAU*s]	Height [mAU]	Area %
1	6.360	BB	0.0463	35.92137	12.22068	0.2130
2	40.827	BB	0.5982	8448.68555	226.50081	50.1091
3	42.086	BB	0.6652	8375.97168	197.73247	49.6778

^{1}H NMR

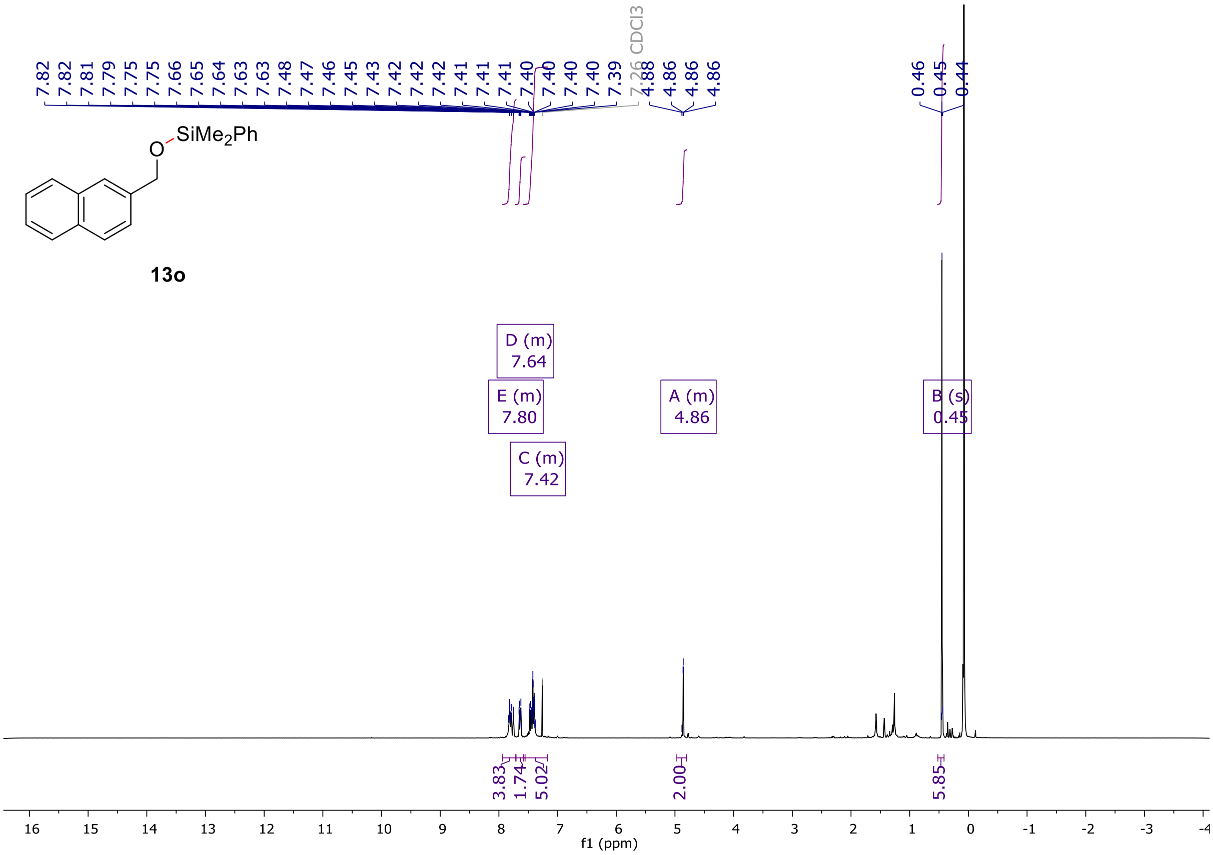

$^{13}C\{^{1}H\}$ NMR

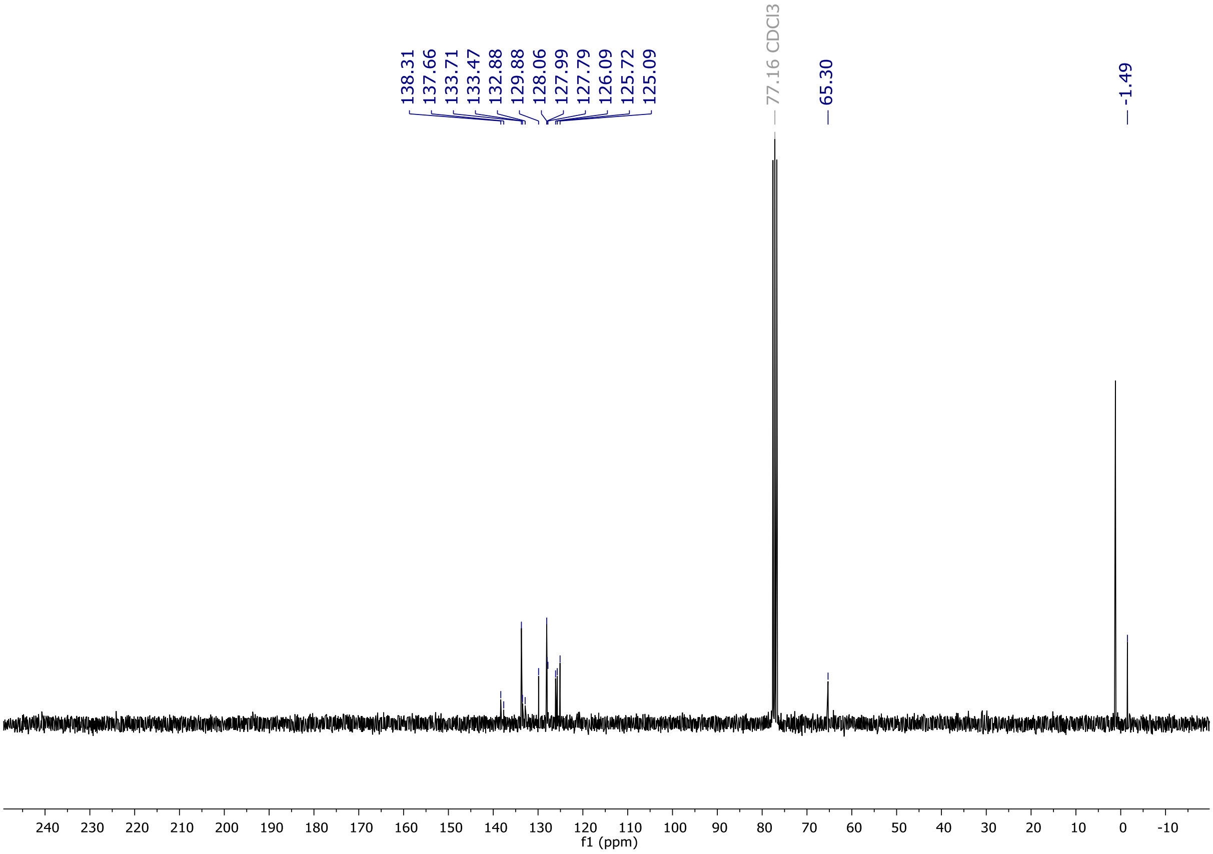

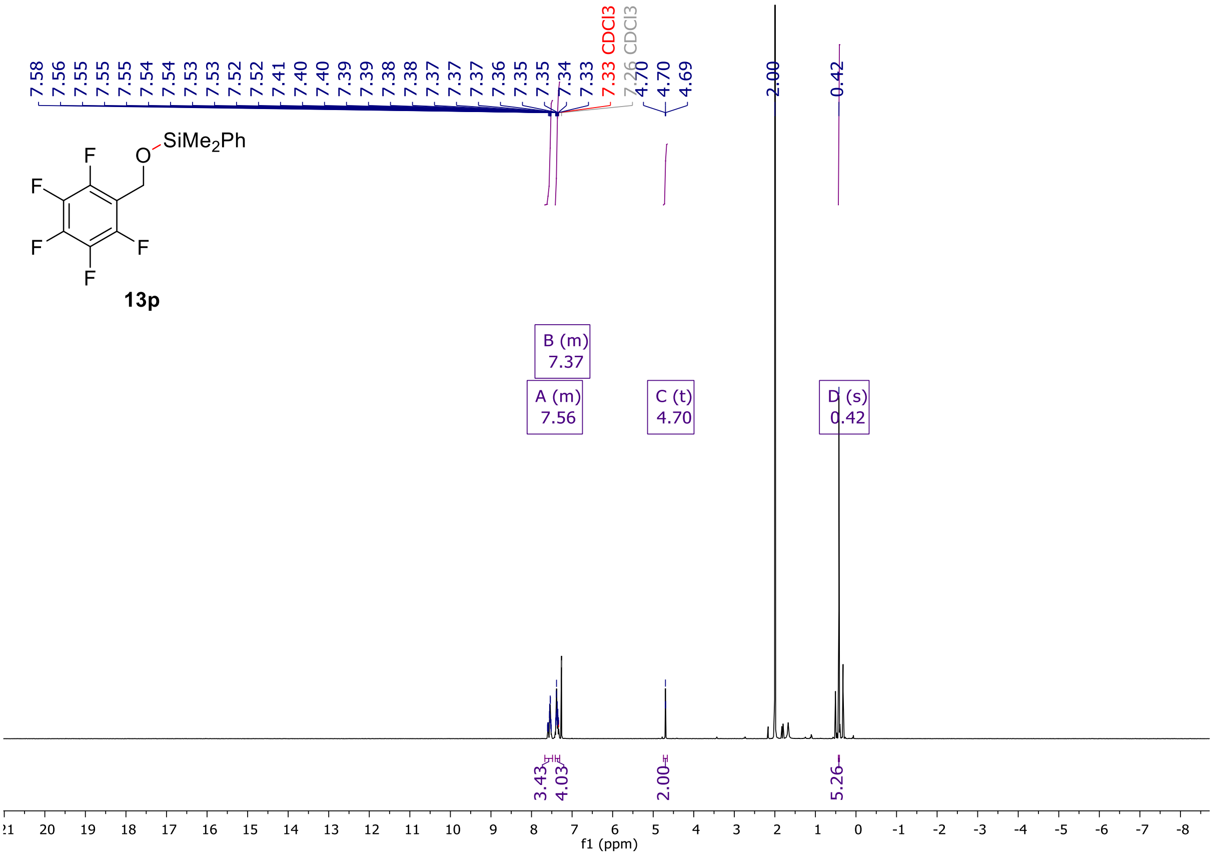
7.58 7.56 7.55 7.55 7.55 7.54 7.54 7.53 7.53 7.52 7.52 7.41 7.40 7.40 7.39 7.39 7.38 7.38 7.37 7.37 7.37 7.36 7.35 7.35 7.34 7.33
7.33 CDCl3
7.26 CDCl3
4.70 4.70 4.69
2.00
0.42
F
F
F
F
F
O
SiMe2Ph
13p
B (m) 7.37
A (m) 7.56
C (t) 4.70
D (s) 0.42
3.43
4.03
2.00
5.26
f1 (ppm)

$^{13}C\{^1H\}$ NMR

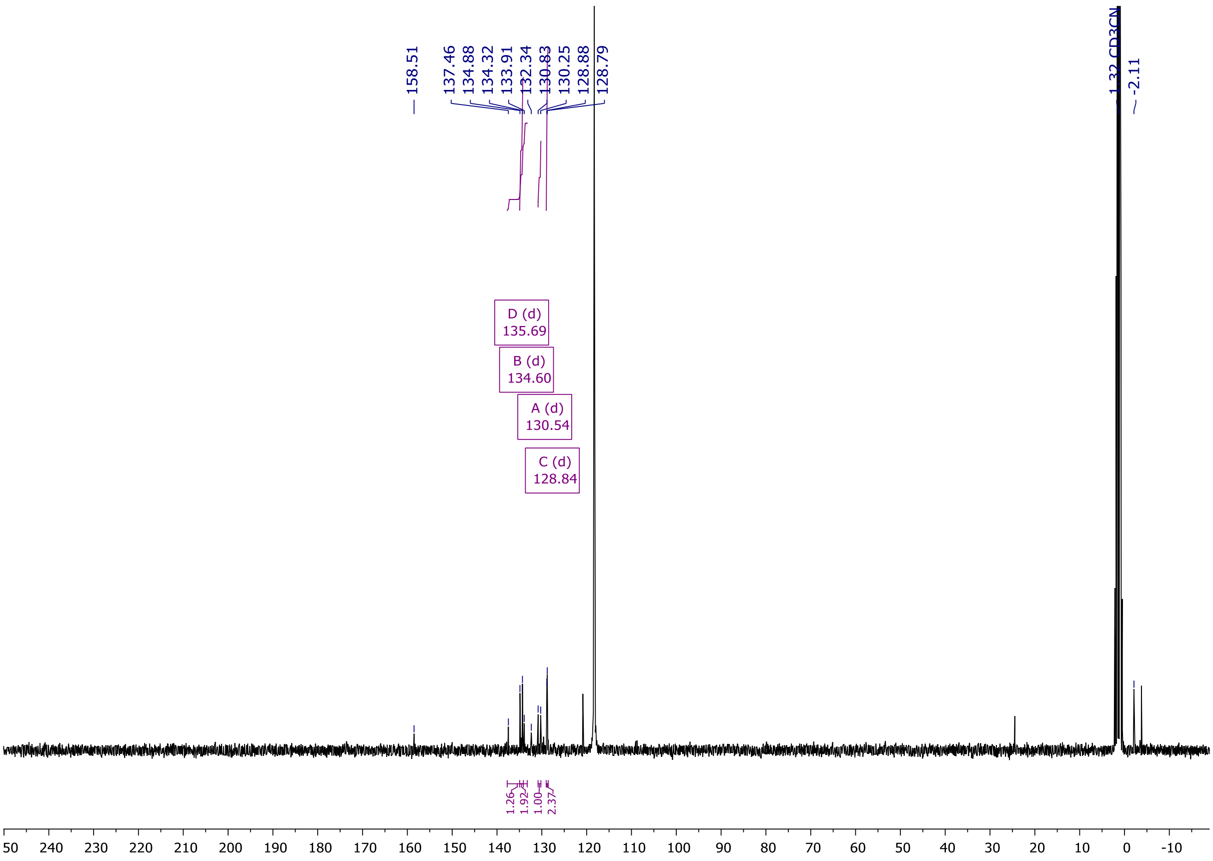
158.51
137.46 134.88 134.32 133.91 132.34 130.83 130.25 128.88 128.79
1.32 CD3CN
-2.11
D (d) 135.69
B (d) 134.60
A (d) 130.54
C (d) 128.84
1.26
1.92
1.00
2.37

$^{19}F\{^{1}H\}$ NMR

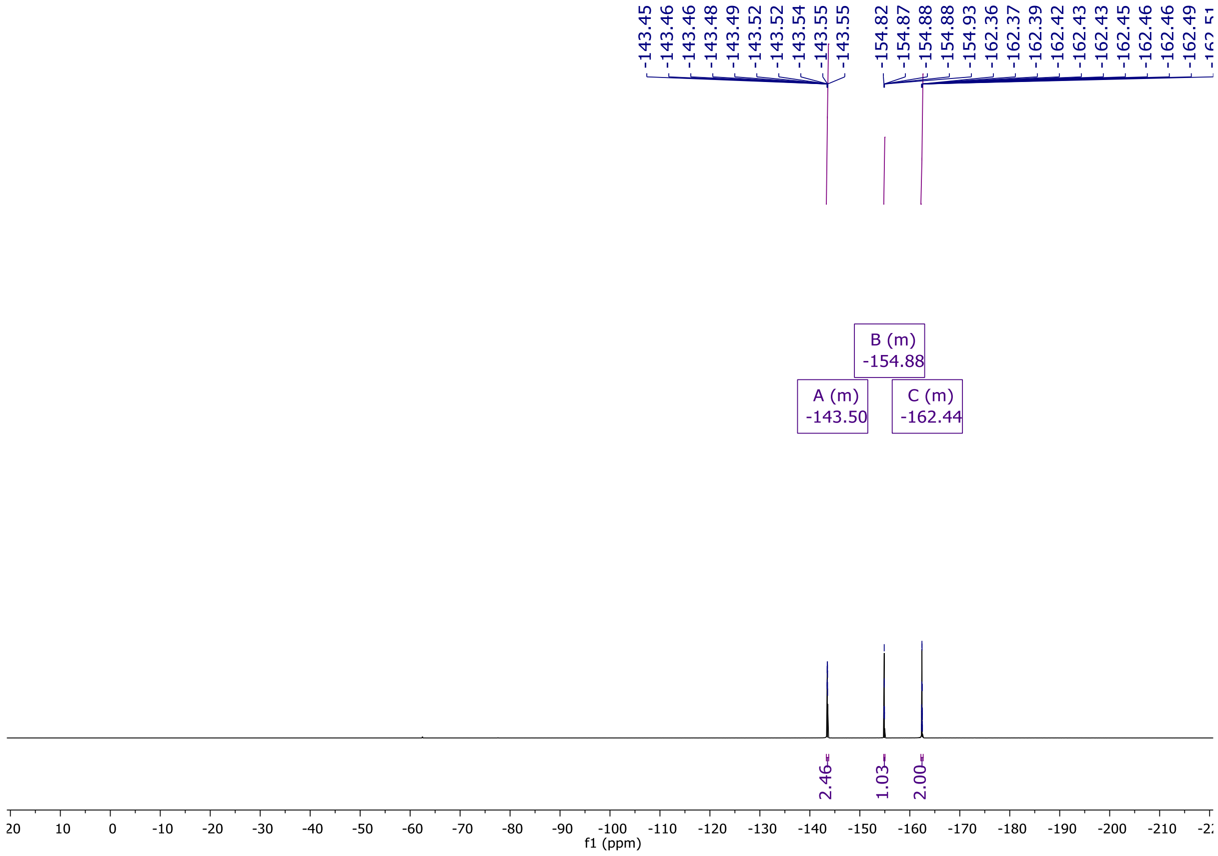
-143.45
-143.46
-143.46
-143.48
-143.49
-143.52
-143.52
-143.54
-143.55
-143.55
-154.82
-154.87
-154.88
-154.88
-154.93
-162.36
-162.37
-162.39
-162.42
-162.43
-162.43
-162.45
-162.46
-162.46
-162.49
B (m)
-154.88
A (m)
-143.50
C (m)
-162.44
2.46
1.03
2.00
20
10
0
-10
-20
-30
-40
-50
-60
-70
-80
-90
-100
-110
-120
-130
-140
-150
-160
-170
-180
-190
-200
-210
f1 (ppm)

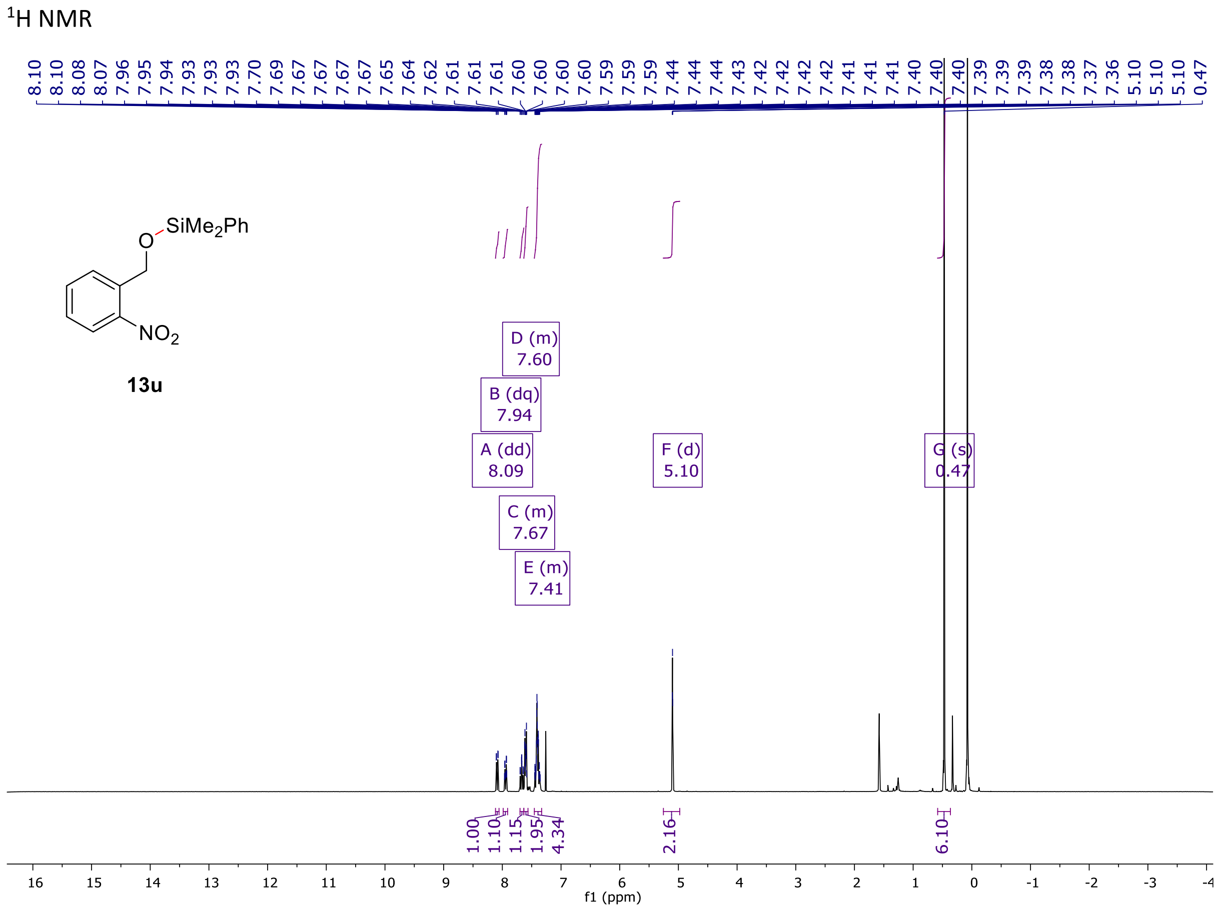

¹H NMR
SiMe2Ph
NO2
13u
A (dd) 8.09
B (dq) 7.94
C (m) 7.67
D (m) 7.60
E (m) 7.41
F (d) 5.10
G (s) 0.47
1.00
1.10
1.15
1.95
4.34
2.16
6.10
f1 (ppm)

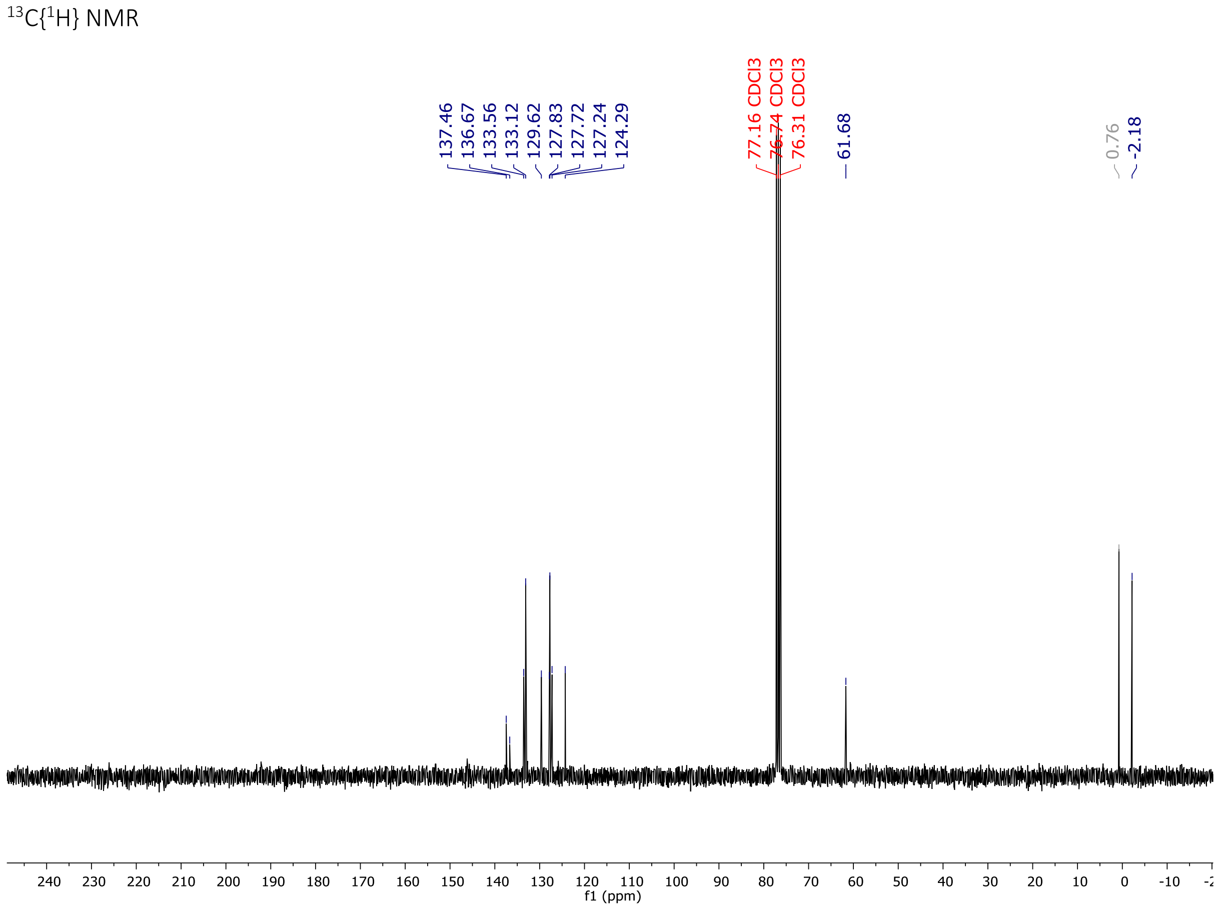

13C{1H} NMR
137.46
136.67
133.56
133.12
129.62
127.83
127.72
127.24
124.29
77.16 CDCl3
76.74 CDCl3
76.31 CDCl3
61.68
0.76
-2.18
f1 (ppm)

^1H NMR

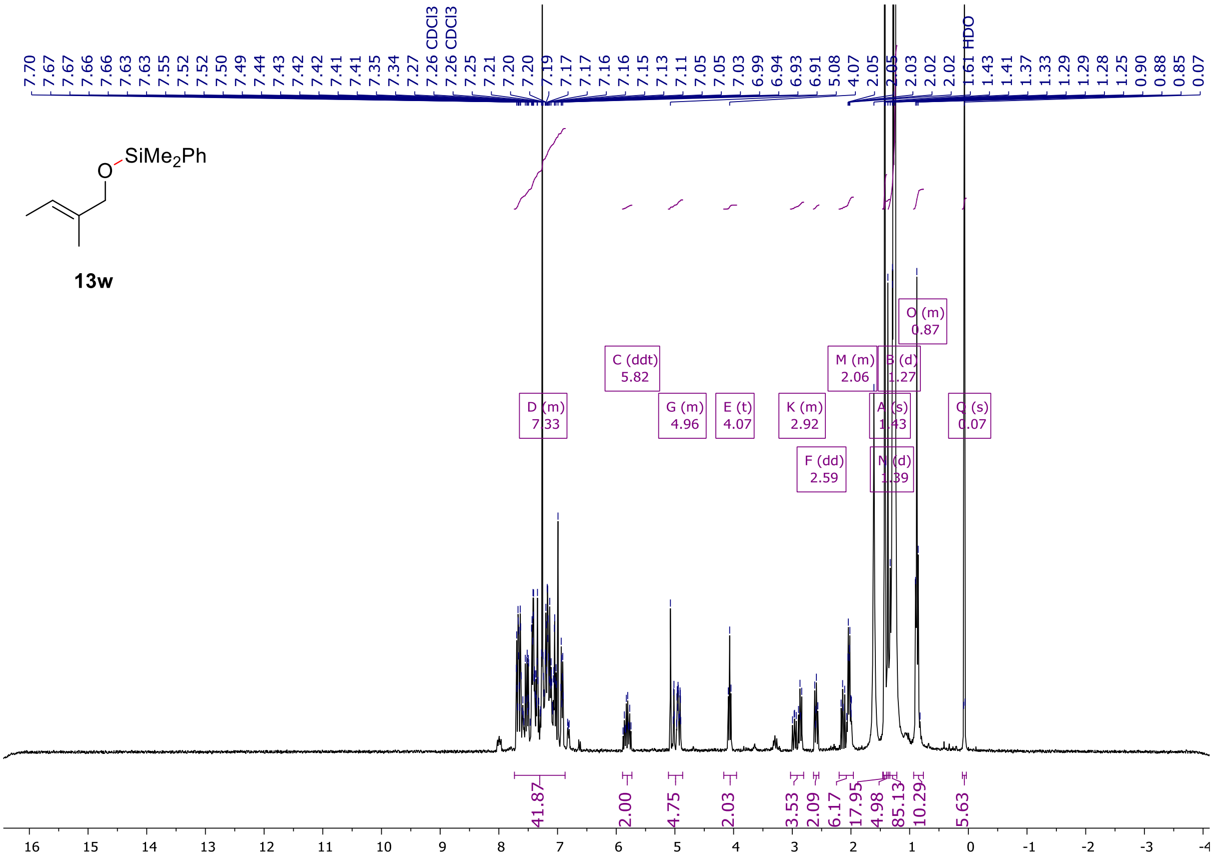

$^{13}C\{^1H\}$ NMR

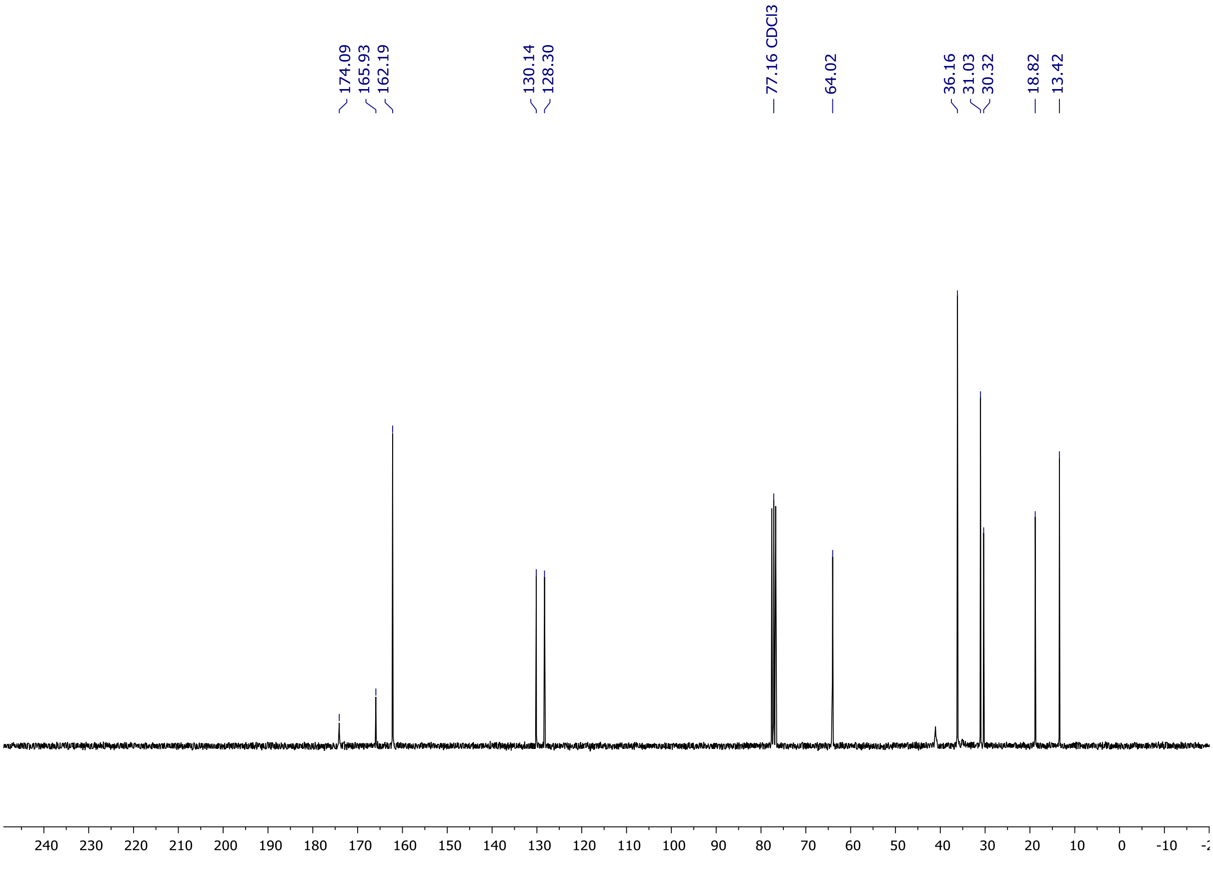

^{1}H NMR

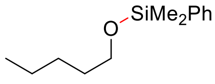

13x

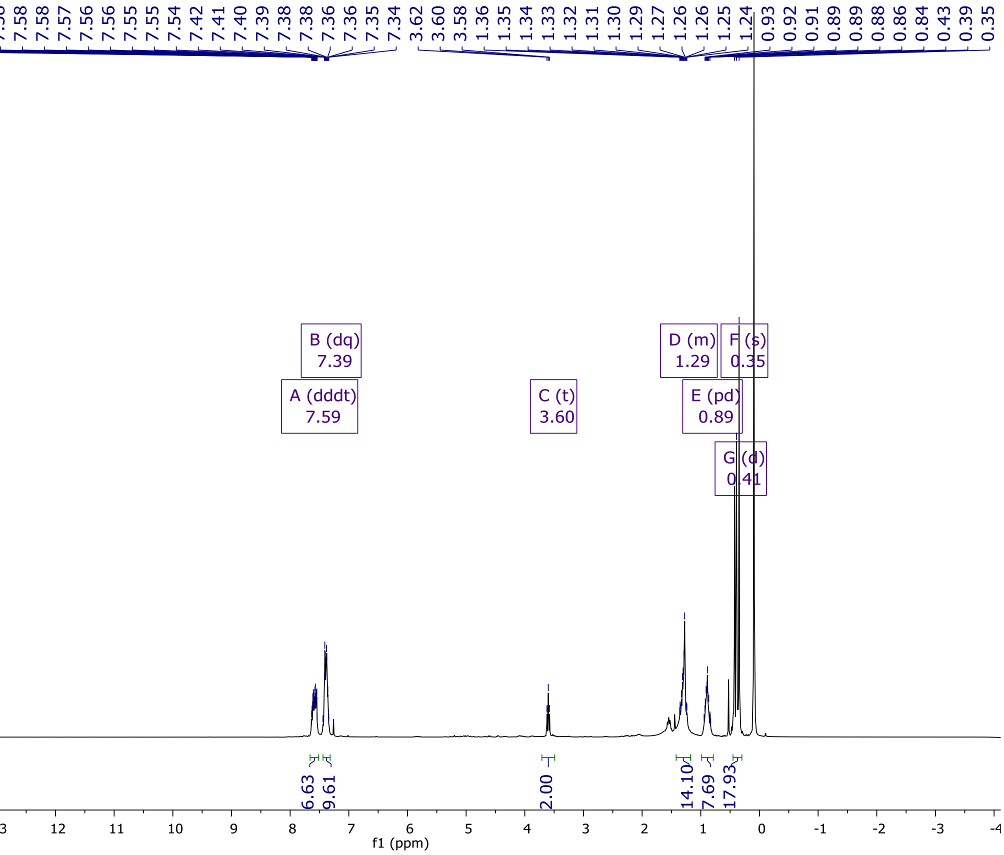

^{13}C{^{1}H} NMR

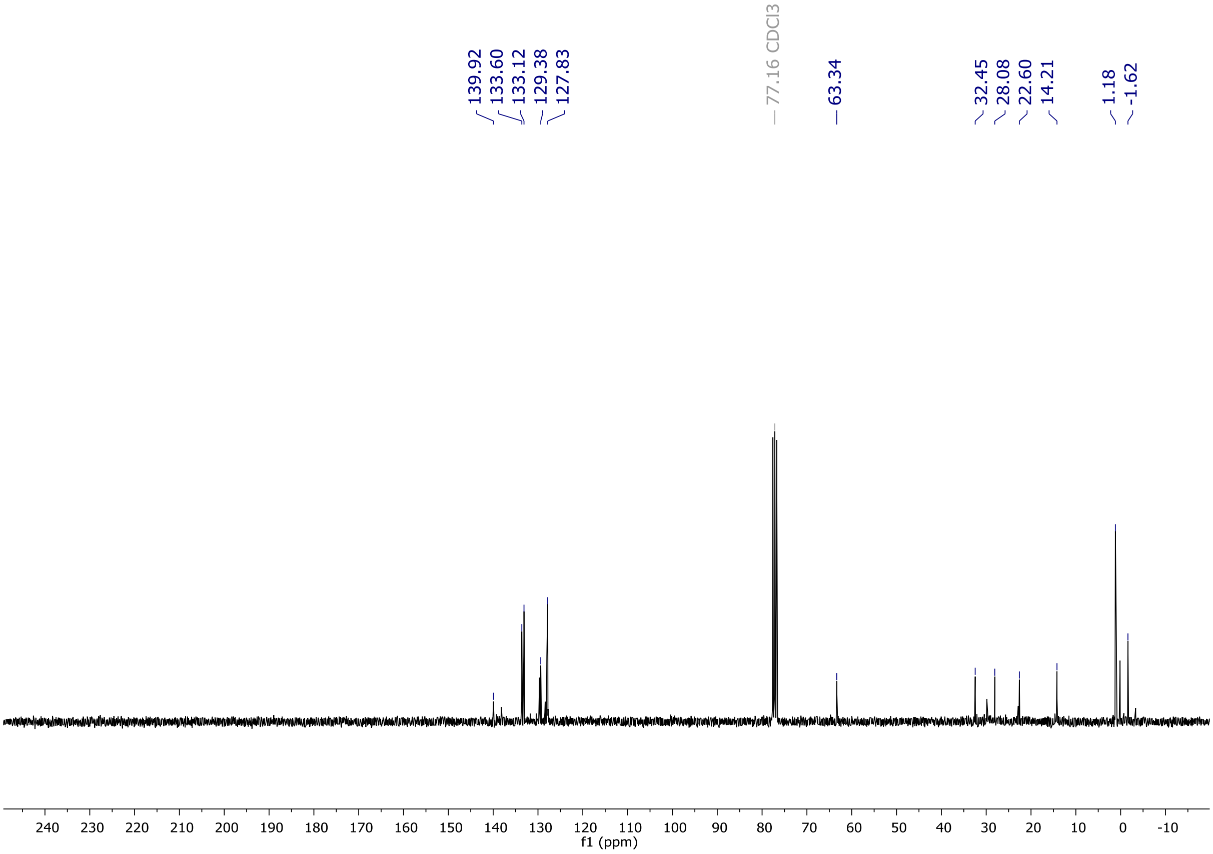

^{1}H NMR

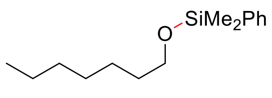

13y

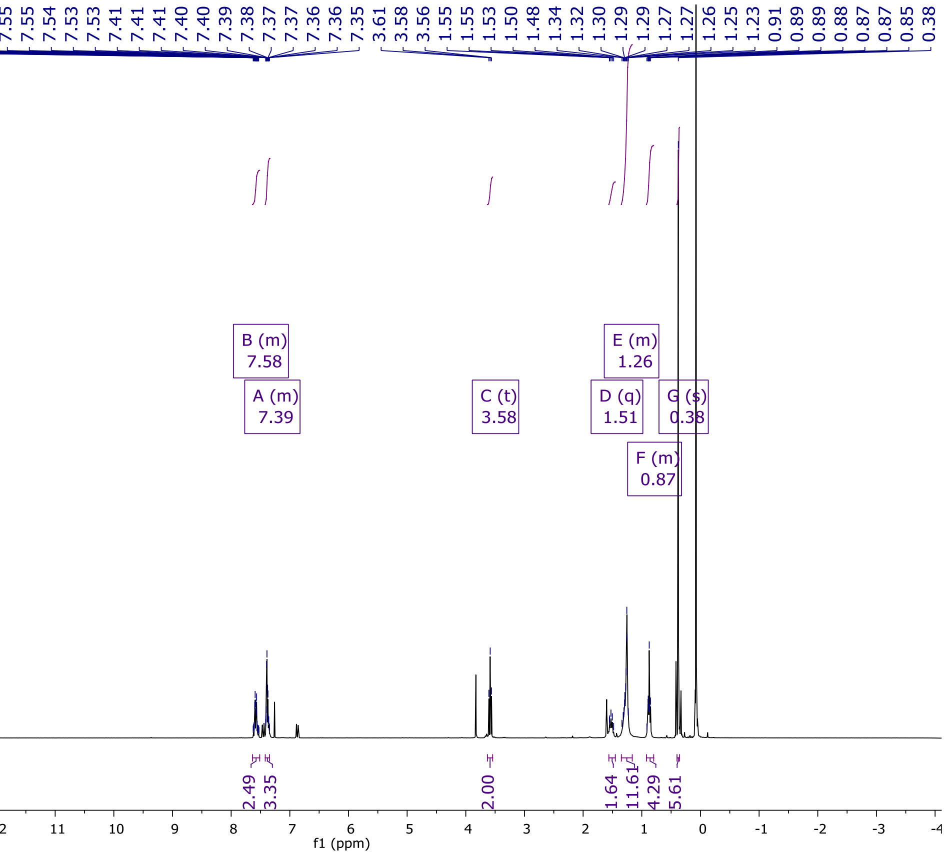

7.62
7.62
7.61
7.60
7.59
7.58
7.58
7.57
7.57
7.57
7.56
7.55
7.55
7.54
7.53
7.53
7.41
7.41
7.41
7.40
7.40
7.39
7.38
7.37
7.37
7.36
7.36
7.35
3.61
3.58
3.56
1.55
1.55
1.53
1.50
1.48
1.34
1.32
1.30
1.29
1.29
1.27
1.27
1.26
1.25
1.23
0.91
0.89
0.88
0.87
0.87
0.85
0.38
B (m) 7.58
A (m) 7.39
C (t) 3.58
E (m) 1.26
D (q) 1.51
G (s) 0.38
F (m) 0.87
2.49
3.35
2.00
1.64
11.61
4.29
5.61
f1 (ppm)

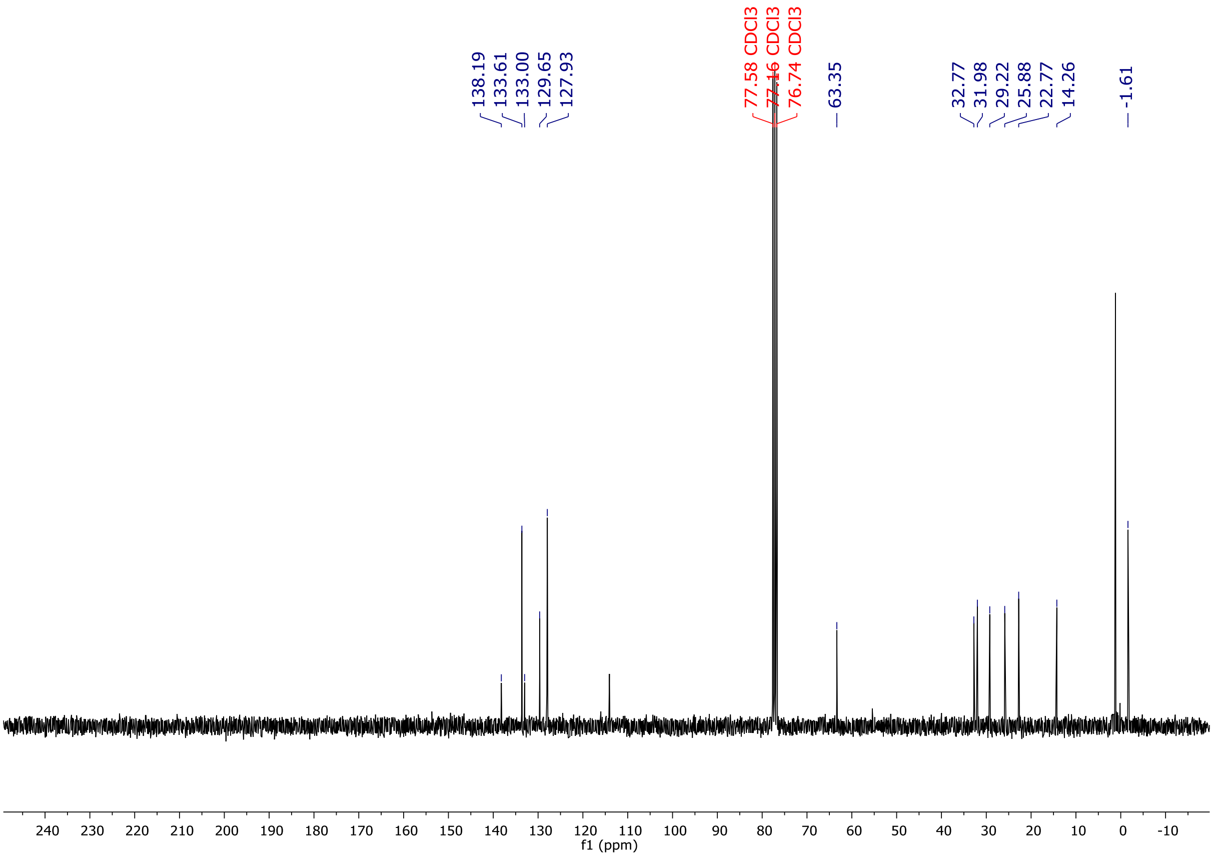

138.19
133.61
133.00
129.65
127.93
77.58 CDCl3
77.16 CDCl3
76.74 CDCl3
63.35
32.77
31.98
29.22
25.88
22.77
14.26
-1.61
f1 (ppm)

^{1}H NMR

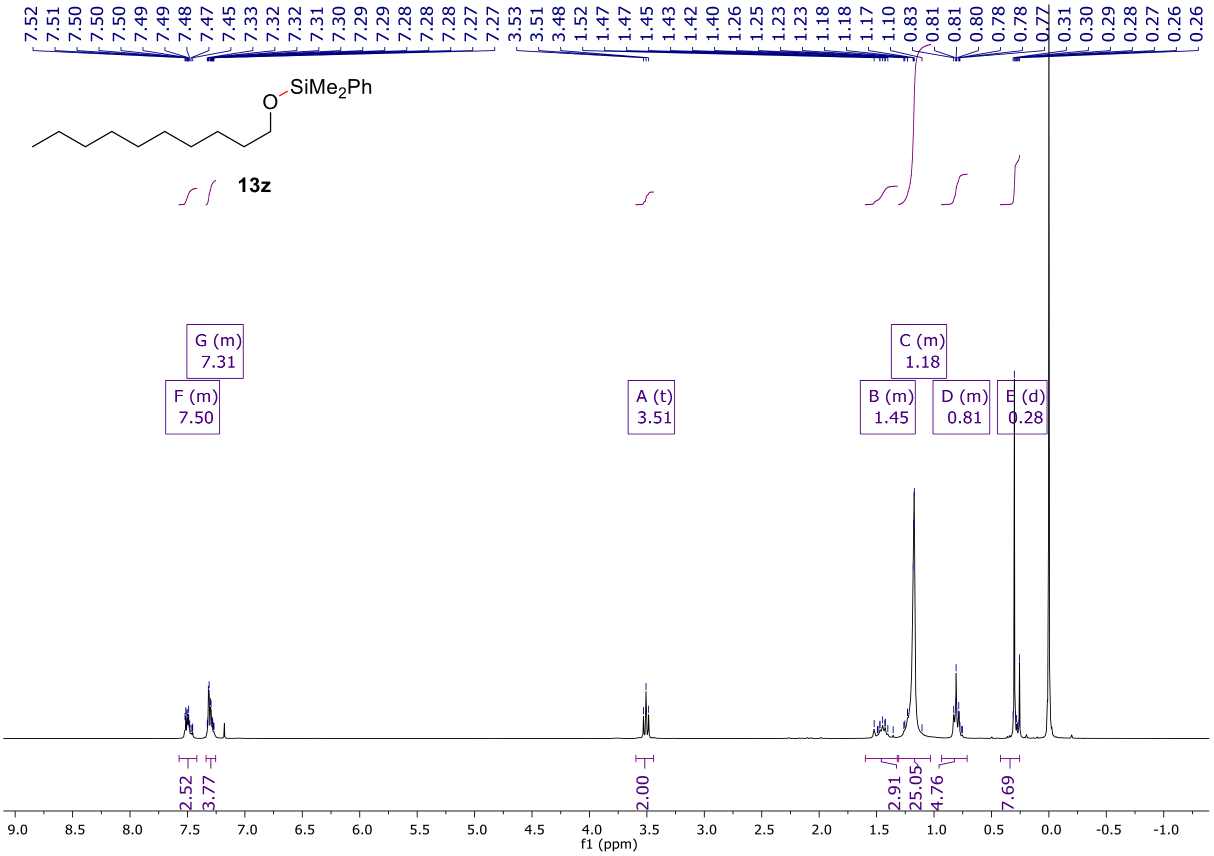

$^{13}C\{^{1}H\}$ NMR

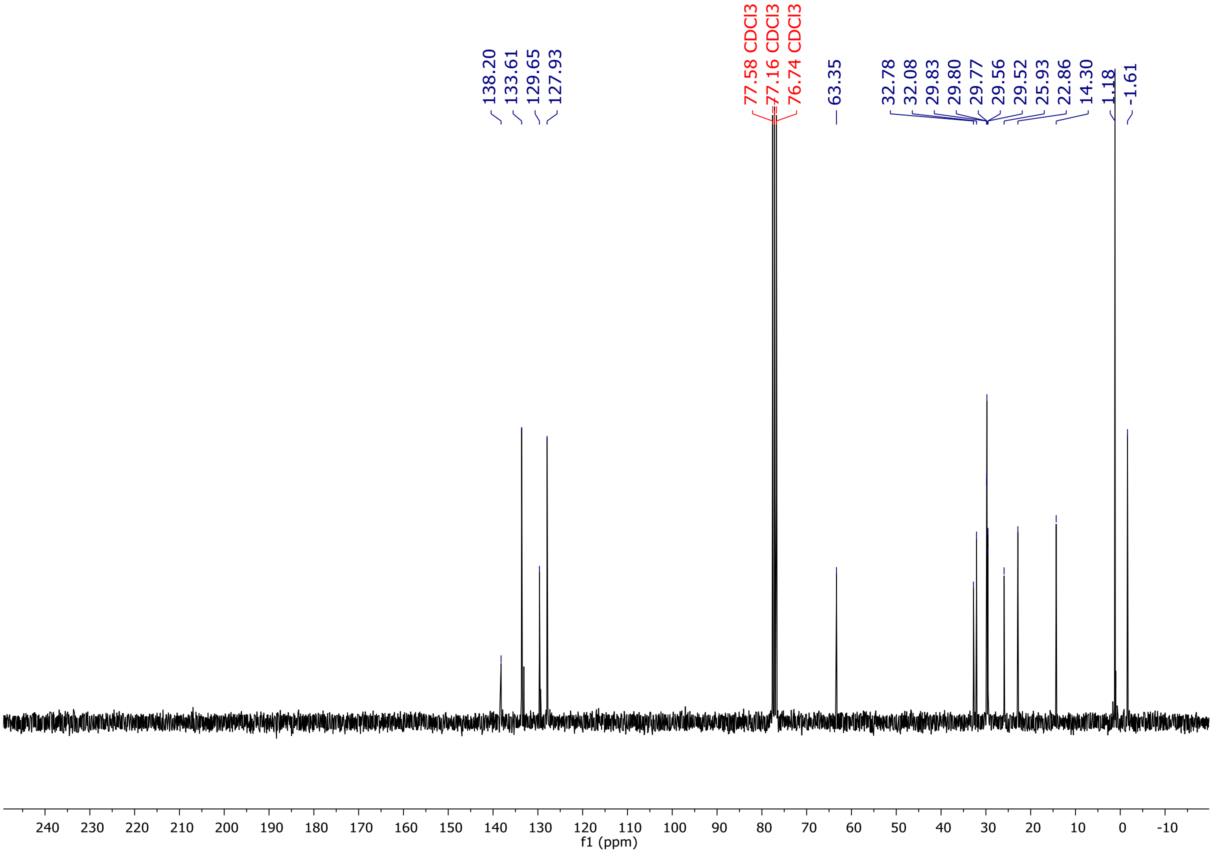

^{1}H NMR

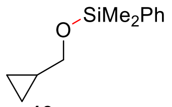

13aa

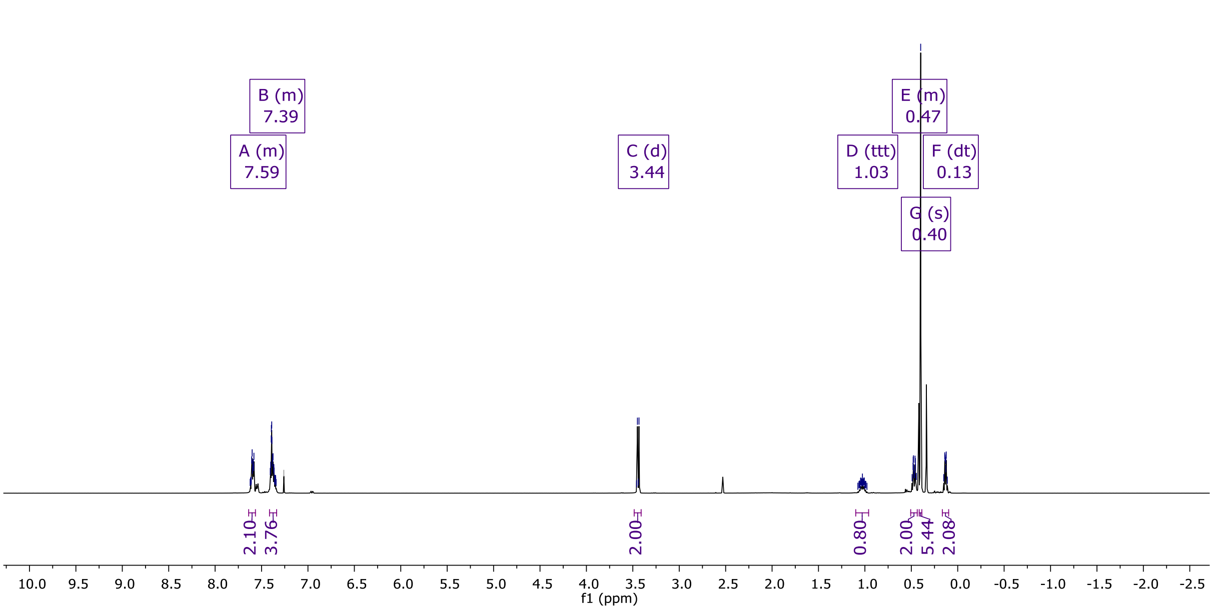

$^{13}C\{^{1}H\}$ NMR

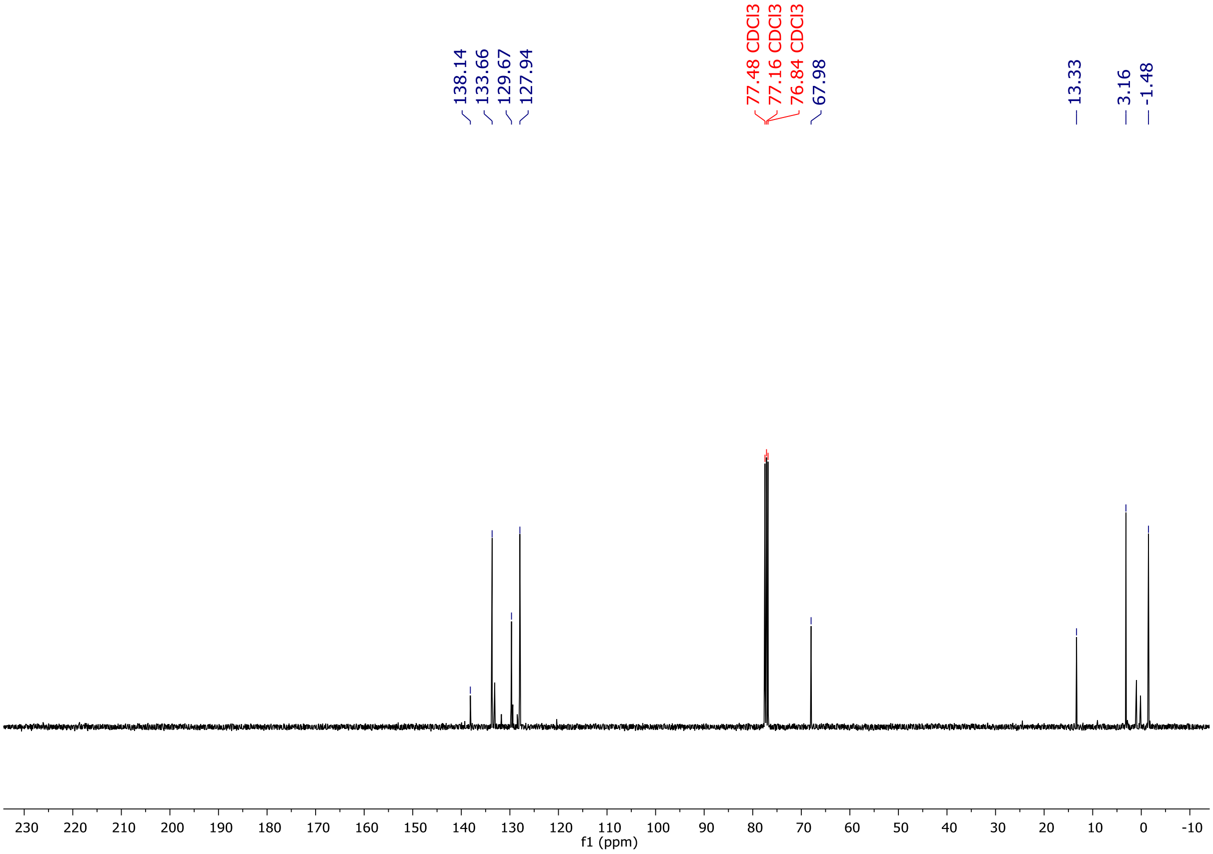

9. Crystallographic details

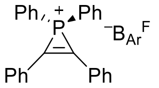

3·B$_{Ar}^F$

Table S3 Crystal data and structure refinement for **3·B$_{Ar}^F$**.

Identification code	s18rlw22
Empirical formula	$C_{60}H_{36}BF_{24}PCl_2$
Formula weight	1325.57
Temperature/K	150.01(10)
Crystal system	monoclinic
Space group	$P2_1/c$
a/Å	13.4192(2)
b/Å	25.9960(4)
c/Å	18.4476(2)
α/°	90
β/°	101.882(1)
γ/°	90
Volume/Å^3	6297.48(15)
Z	4
ρ_{calc}g/cm^3	1.398
μ/mm^{-1}	2.145
F(000)	2664.0
Crystal size/mm^3	0.293 × 0.203 × 0.168
Radiation	CuKα (λ = 1.54184)
2Θ range for data collection/°	5.96 to 146.508
Index ranges	-16 ≤ h ≤ 14, -32 ≤ k ≤ 31, -22 ≤ l ≤ 22
Reflections collected	63310
Independent reflections	12536 [R_{int} = 0.0465, R_{sigma} = 0.0322]
Data/restraints/parameters	12536/418/892
Goodness-of-fit on F^2	1.047
Final R indexes [I>=2σ (I)]	R_1 = 0.0670, wR_2 = 0.1944
Final R indexes [all data]	R_1 = 0.0810, wR_2 = 0.2087
Largest diff. peak/hole / e Å^{-3}	0.61/-0.45

The asymmetric unit in the structure of ***3·B$_{Ar}^F$*** comprises one cation, one BAr^F anion and two regions of solvent. The cation was entirely ordered, but (not unexpectedly) some of the anion CF_3 groups fell foul of disorder. In particular, the fluorine atoms attached to C34 were treated for 55:45 disorder, while the entire trifluoromethyl groups based on C49, C50 and C85 were each split over 2 sites in respective disorder ratios of 65:35, 50:50 and 80:20. C-F, C-C and F…F distance restraints (along with some ADP restraints) were used in these regions to assist convergence.
The electron density for the guest solvent was very smeared, and an elaborate model would have been required in order to take account of same as part of the refinement. After multiple abortive attempts, an allowance for 2 molecules of CH_2Cl_2 per asymmetric unit was made in the formula as presented, alongside employment of the solvent mask algorithm available in Olex-2. Examination of the gross structure revealed that there are channels present in the lattice which accommodate said solvent. Thus, the accompanying disorder level was not surprising.

Table S4 Bond Lengths for **3·B$_{Ar}$^{F}**.

Atom	Atom	Length/Å
P1	C7	1.748(2)
P1	C8	1.746(3)
P1	C15	1.788(3)
P1	C21	1.777(3)
C1	C2	1.383(4)
C1	C6	1.397(4)
C2	C3	1.375(5)
C3	C4	1.370(5)
C4	C5	1.390(4)
C5	C6	1.383(4)
C6	C7	1.451(3)
C7	C8	1.340(4)
C8	C9	1.448(4)
C9	C10	1.378(4)
C9	C14	1.386(4)
C10	C11	1.375(5)
C11	C12	1.354(5)
C12	C13	1.370(5)
C13	C14	1.380(5)
C15	C16	1.379(4)
C15	C20	1.395(5)
C16	C17	1.383(5)
C17	C18	1.376(6)
C18	C19	1.378(7)
C19	C20	1.386(5)
C21	C22	1.395(4)
C21	C26	1.390(4)
C22	C23	1.380(5)
C23	C24	1.390(6)
C24	C25	1.366(6)
C25	C26	1.388(5)
F4	C33	1.335(4)
F5	C33	1.328(3)
F6	C33	1.332(4)
F7	C42	1.313(4)
F8	C42	1.348(5)
F9	C42	1.307(4)
F19	C57	1.303(4)
F20	C57	1.313(4)
F21	C57	1.314(4)
F22	C58	1.342(5)
F23	C58	1.332(4)
F24	C58	1.288(5)
C27	C28	1.402(3)
C27	C32	1.397(3)
C27	B1	1.636(3)
C28	C29	1.393(4)
C29	C30	1.384(4)
C34	F2	1.279(5)
C34	F3	1.331(6)
C34	F1A	1.263(7)
C34	F2A	1.298(7)
C34	F3A	1.319(8)
C35	C36	1.400(3)
C35	C40	1.394(3)
C35	B1	1.632(3)
C36	C37	1.382(4)
C37	C38	1.388(4)
C37	C42	1.492(4)
C38	C39	1.378(4)
C39	C40	1.389(4)
C39	C85	1.486(5)
C39	C85A	1.466(15)
C43	C44	1.402(3)
C43	C48	1.385(4)
C43	B1	1.639(3)
C44	C45	1.392(3)
C45	C46	1.375(4)
C45	C50	1.486(10)
C45	C50A	1.502(9)
C46	C47	1.388(4)
C47	C48	1.395(4)
C47	C49	1.490(5)
C47	C49A	1.488(6)
C51	C52	1.398(3)
C51	C56	1.398(3)
C51	B1	1.644(3)
C52	C53	1.394(4)
C53	C54	1.384(4)
C53	C58	1.507(4)
C54	C55	1.382(4)
C55	C56	1.397(4)
C55	C57	1.493(4)
F10	C85	1.300(16)
F11	C85	1.315(5)
F12	C85	1.398(7)
F13	C50	1.326(5)
F14	C50	1.331(5)
F15	C50	1.332(5)
F16	C49	1.287(15)
F17	C49	1.322(15)
F18	C49	1.339(12)
F10A	C85A	1.30(2)
F11A	C85A	1.284(17)
F12A	C85A	1.357(19)
F13A	C50A	1.323(10)

C29	C34	1.497(4)		F14A	C50A	1.376(10)
C30	C31	1.382(4)		F15A	C50A	1.317(9)
C31	C32	1.394(3)		F16A	C49A	1.25(2)
C31	C33	1.494(4)		F17A	C49A	1.39(2)
C34	F1	1.320(6)		F18A	C49A	1.31(2)

Table S5 Bond Angles for ***3·B$_{Ar}$^{F}***

Atom	Atom	Atom	Angle/˚	Atom	Atom	Atom	Angle/˚
C7	P1	C15	121.77(12)	F7	C42	F8	102.1(3)
C7	P1	C21	120.30(12)	F7	C42	C37	112.7(3)
C8	P1	C7	45.12(12)	F8	C42	C37	111.7(3)
C8	P1	C15	117.58(13)	F9	C42	F7	110.1(3)
C8	P1	C21	126.23(12)	F9	C42	F8	104.7(3)
C21	P1	C15	111.19(12)	F9	C42	C37	114.5(3)
C2	C1	C6	119.5(3)	C44	C43	B1	121.4(2)
C3	C2	C1	120.2(3)	C48	C43	C44	115.9(2)
C4	C3	C2	120.5(3)	C48	C43	B1	122.3(2)
C3	C4	C5	120.2(3)	C45	C44	C43	122.0(2)
C6	C5	C4	119.7(3)	C44	C45	C50	118.8(3)
C1	C6	C7	120.4(3)	C44	C45	C50A	118.1(4)
C5	C6	C1	119.9(2)	C46	C45	C44	121.1(2)
C5	C6	C7	119.7(3)	C46	C45	C50	120.0(3)
C6	C7	P1	145.7(2)	C46	C45	C50A	120.6(4)
C8	C7	P1	67.37(15)	C45	C46	C47	117.9(2)
C8	C7	C6	146.9(3)	C46	C47	C48	120.8(3)
C7	C8	P1	67.51(16)	C46	C47	C49	120.3(5)
C7	C8	C9	146.9(2)	C46	C47	C49A	117.0(11)
C9	C8	P1	144.4(2)	C48	C47	C49	118.9(5)
C10	C9	C8	120.2(3)	C48	C47	C49A	121.3(11)
C10	C9	C14	118.2(3)	C43	C48	C47	122.3(2)
C14	C9	C8	121.4(2)	C52	C51	B1	121.9(2)
C11	C10	C9	120.5(3)	C56	C51	C52	115.9(2)
C12	C11	C10	121.0(3)	C56	C51	B1	121.9(2)
C11	C12	C13	119.4(3)	C53	C52	C51	121.9(2)
C12	C13	C14	120.4(3)	C52	C53	C58	119.7(2)
C13	C14	C9	120.4(3)	C54	C53	C52	121.1(2)
C16	C15	P1	121.3(2)	C54	C53	C58	119.2(2)
C16	C15	C20	120.8(3)	C55	C54	C53	118.1(2)
C20	C15	P1	117.8(2)	C54	C55	C56	120.7(2)
C15	C16	C17	119.5(4)	C54	C55	C57	120.1(2)
C18	C17	C16	120.1(4)	C56	C55	C57	119.2(2)
C17	C18	C19	120.4(3)	C55	C56	C51	122.2(2)
C18	C19	C20	120.5(4)	F19	C57	F20	105.6(3)
C19	C20	C15	118.6(4)	F19	C57	F21	104.3(3)
C22	C21	P1	120.0(2)	F19	C57	C55	112.7(3)
C26	C21	P1	118.9(2)	F20	C57	F21	106.2(3)
C26	C21	C22	120.8(3)	F20	C57	C55	113.8(2)
C23	C22	C21	118.8(3)	F21	C57	C55	113.4(3)
C22	C23	C24	120.6(3)	F22	C58	C53	111.8(3)

C25	C24	C23	120.1(3)	F23	C58	F22	102.9(3)
C24	C25	C26	120.6(3)	F23	C58	C53	112.6(3)
C25	C26	C21	119.0(3)	F24	C58	F22	106.3(3)
C28	C27	B1	122.6(2)	F24	C58	F23	109.8(4)
C32	C27	C28	115.7(2)	F24	C58	C53	112.8(4)
C32	C27	B1	121.6(2)	C27	B1	C43	104.44(19)
C29	C28	C27	122.2(2)	C27	B1	C51	112.05(19)
C28	C29	C34	119.2(3)	C35	B1	C27	111.32(18)
C30	C29	C28	120.9(2)	C35	B1	C43	112.64(19)
C30	C29	C34	119.9(3)	C35	B1	C51	104.13(18)
C31	C30	C29	118.0(2)	C43	B1	C51	112.48(18)
C30	C31	C32	121.0(2)	F16	C49	C47	111.5(10)
C30	C31	C33	121.1(2)	F16	C49	F17	107.0(8)
C32	C31	C33	117.9(2)	F16	C49	F18	104.3(8)
C31	C32	C27	122.2(2)	F17	C49	C47	114.9(8)
F4	C33	C31	112.0(3)	F17	C49	F18	103.7(10)
F5	C33	F4	107.3(3)	F18	C49	C47	114.5(8)
F5	C33	F6	106.2(2)	F13	C50	C45	108.0(6)
F5	C33	C31	113.8(3)	F13	C50	F14	107.4(6)
F6	C33	F4	104.1(3)	F13	C50	F15	107.2(6)
F6	C33	C31	112.8(3)	F14	C50	C45	114.7(6)
F1	C34	C29	113.3(3)	F14	C50	F15	106.1(5)
F1	C34	F3	104.4(5)	F15	C50	C45	113.0(6)
F2	C34	C29	113.8(3)	F10	C85	C39	110.5(8)
F2	C34	F1	109.7(6)	F10	C85	F11	96.6(8)
F2	C34	F3	102.3(5)	F10	C85	F12	123.8(8)
F3	C34	C29	112.5(3)	F11	C85	C39	114.0(4)
F1A	C34	C29	113.3(4)	F11	C85	F12	99.8(4)
F1A	C34	F2A	106.8(6)	F12	C85	C39	110.7(4)
F1A	C34	F3A	104.0(7)	F16A	C49A	C47	109.2(15)
F2A	C34	C29	112.1(4)	F16A	C49A	F17A	104.9(17)
F2A	C34	F3A	106.7(7)	F16A	C49A	F18A	116.9(16)
F3A	C34	C29	113.3(4)	F17A	C49A	C47	114.7(15)
C36	C35	B1	121.6(2)	F18A	C49A	C47	115.3(15)
C40	C35	C36	115.4(2)	F18A	C49A	F17A	95.0(15)
C40	C35	B1	122.8(2)	F13A	C50A	C45	112.3(8)
C37	C36	C35	122.4(2)	F13A	C50A	F14A	101.3(7)
C36	C37	C38	121.1(2)	F14A	C50A	C45	114.1(7)
C36	C37	C42	118.7(2)	F15A	C50A	C45	115.3(6)
C38	C37	C42	120.1(3)	F15A	C50A	F13A	110.5(8)
C39	C38	C37	117.5(2)	F15A	C50A	F14A	102.0(7)
C38	C39	C40	121.2(2)	F10A	C85A	C39	123.5(14)
C38	C39	C85	122.2(3)	F10A	C85A	F12A	99.7(13)
C38	C39	C85A	110.3(8)	F11A	C85A	C39	111.7(13)
C40	C39	C85	116.7(3)	F11A	C85A	F10A	105.2(15)
C40	C39	C85A	128.1(8)	F11A	C85A	F12A	102.6(14)
C39	C40	C35	122.4(2)	F12A	C85A	C39	111.7(13)

Experimental

Single crystals of $C_{60}H_{36}BF_{24}PCl_2$ [**3·B$_{Ar}$^{F}**] were grown by slow diffusion in pentane/dichloromethane solution. A suitable crystal was selected and mounted on a SuperNova, Dual, Cu at home/near, EosS2 diffractometer. The crystal was kept at 150.01(10) K during data collection. Using Olex2,[33] the structure was solved with the olex2.solve[34] structure solution program using Charge Flipping and refined with the ShelXL[35] refinement package using Least Squares minimisation.

Crystal structure determination of [3·B$_{Ar}$^{F}]

Crystal Data for $C_{60}H_{36}BF_{24}PCl_2$ (M =1325.57 g/mol): monoclinic, space group P2$_1$/c (no. 14), a = 13.4192(2) Å, b = 25.9960(4) Å, c = 18.4476(2) Å, β = 101.8820(10)°, V = 6297.48(15) Å^3, Z = 4, T = 150.01(10) K, μ(CuKα) = 2.145 mm^{-1}, $Dcalc$ = 1.398 g/cm^3, 63310 reflections measured (5.96° ≤ 2Θ ≤ 146.508°), 12536 unique (R_{int} = 0.0465, R_{sigma} = 0.0322) which were used in all calculations. The final R_1 was 0.0670 (I > 2σ(I)) and wR_2 was 0.2087 (all data).

Refinement model description

Number of restraints - 418, number of constraints - unknown.

Details:

```
1. Fixed Uiso
 At 1.2 times of:
  All C(H) groups
2. Restrained distances
 F1-C34 ≈ F1A-C34
 with sigma of 0.02
 F2-C34 ≈ F2A-C34
 with sigma of 0.02
 F3-C34 ≈ F3A-C34
 with sigma of 0.02
 F16-C49 ≈ F16A-C49A
 with sigma of 0.02
 F17-C49 ≈ F17A-C49A
 with sigma of 0.02
 F18-C49 ≈ F18A-C49A
 with sigma of 0.02
 C47-C49 ≈ C47-C49A
 with sigma of 0.02
 F16-F17 ≈ F16A-F17A
 with sigma of 0.04
 F16-F18 ≈ F16A-F18A
 with sigma of 0.04
 F16-C47 ≈ F16A-C47
 with sigma of 0.04
 F17-F18 ≈ F17A-F18A
 with sigma of 0.04
 F17-C47 ≈ F17A-C47
 with sigma of 0.04
 F18-C47 ≈ F18A-C47
 with sigma of 0.04
 F13-C50 ≈ F13A-C50A
 with sigma of 0.02
 F14-C50 ≈ F14A-C50A
 with sigma of 0.02
 F15-C50 ≈ F15A-C50A
 with sigma of 0.02
 C45-C50 ≈ C45-C50A
 with sigma of 0.02
 F13-F14 ≈ F13A-F14A
 with sigma of 0.04
 F13-F15 ≈ F13A-F15A
 with sigma of 0.04
 F13-C45 ≈ F13A-C45
```

with sigma of 0.04
F14-F15 ≈ F14A-F15A
with sigma of 0.04
F14-C45 ≈ F14A-C45
with sigma of 0.04
F15-C45 ≈ F15A-C45
with sigma of 0.04
F10-C85 ≈ F10A-C85A
with sigma of 0.02
F11-C85 ≈ F11A-C85A
with sigma of 0.02
F12-C85 ≈ F12A-C85A
with sigma of 0.02
C39-C85 ≈ C39-C85A
with sigma of 0.02
F10-F11 ≈ F10A-F11A
with sigma of 0.04
F10-F12 ≈ F10A-F12A
with sigma of 0.04
F10-C39 ≈ F10A-C39
with sigma of 0.04
F11-F12 ≈ F11A-F12A
with sigma of 0.04
F11-C39 ≈ F11A-C39
with sigma of 0.04
F12-C39 ≈ F12A-C39
with sigma of 0.04
F13-F14 ≈ F13-F15 ≈ F14-F15
with sigma of 0.01
C50-F13 ≈ C50-F14 ≈ C50-F15
with sigma of 0.005
C49-C47 ≈ C49A-C47
with sigma of 0.005

3. Uiso/Uaniso restraints and constraints
F1 ≈ F2 ≈ F3 ≈ F1A ≈ F2A ≈ F3A: within 2A with sigma of 0.02 and sigma for terminal atoms of 0.01
F16 ≈ F17 ≈ F18 ≈ C49 ≈ F16A ≈ F17A ≈ F18A ≈ C49A: within 2A with sigma of 0.02 and sigma for terminal atoms of 0.01
F13 ≈ F14 ≈ F15 ≈ C50 ≈ F13A ≈ F14A ≈ F15A ≈ C50A: within 2A with sigma of 0.02 and sigma for terminal atoms of 0.01
F10 ≈ F11 ≈ F12 ≈ C85 ≈ F10A ≈ F11A ≈ F12A ≈ C85A: within 2A with sigma of 0.02 and sigma for terminal atoms of 0.01

4. Others
Fixed Sof: F1(0.55) F2(0.55) F3(0.55) F10(0.2) F11(0.8) F12(0.8) F13(0.5) F14(0.5) F15(0.5) F16(0.65) F17(0.65) F18(0.65) C49(0.65) C50(0.5) C85(0.8) F1A(0.45) F2A(0.45) F3A(0.45) F10A(0.2) F11A(0.2) F12A(0.8) F13A(0.5) F14A(0.5) F15A(0.5) F16A(0.35) F17A(0.35) F18A(0.35) C49A(0.35) C50A(0.5) C85A(0.2)

5.a Aromatic/amide H refined with riding coordinates:
C1(H1), C2(H2), C3(H3), C4(H4), C5(H5), C10(H10), C11(H11), C12(H12), C13(H13), C14(H14), C16(H16), C17(H17), C18(H18), C19(H19), C20(H20), C22(H22), C23(H23), C24(H24), C25(H25), C26(H26), C28(H28), C30(H30), C32(H32), C36(H36), C38(H38), C40(H40), C44(H44), C46(H46), C48(H48), C52(H52), C54(H54), C56(H56)

This report has been created with Olex2, compiled on 2018.05.29 svn.r3508 for OlexSys. Please let us know if there are any errors or if you would like to have additional features.

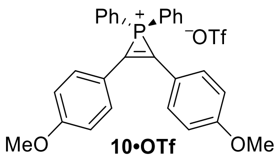

Table S6 Crystal data and structure refinement for **10·OTf**

Identification code	**10·OTf**
Empirical formula	$C_{29}H_{24}F_3O_5PS$
Formula weight	572.51
Temperature/K	150.00(10)
Crystal system	monoclinic
Space group	$P2_1/n$
a/Å	10.5938(2)
b/Å	15.2705(2)
c/Å	16.8486(2)
α/°	90
β/°	95.456(1)
γ/°	90
Volume/Å^3	2713.29(7)
Z	4
ρ_{calc}g/cm^3	1.402
μ/mm^{-1}	2.136
F(000)	1184.0
Crystal size/mm^3	0.189 × 0.102 × 0.068
Radiation	CuKα (λ = 1.54184)
2Θ range for data collection/°	7.83 to 146.092
Index ranges	-13 ≤ h ≤ 11, -18 ≤ k ≤ 18, -20 ≤ l ≤ 20
Reflections collected	23939
Independent reflections	5389 [R_{int} = 0.0353, R_{sigma} = 0.0272]
Data/restraints/parameters	5389/0/354
Goodness-of-fit on F^2	1.028

Final R indexes [I>=2σ (I)]	R_1 = 0.0354, wR_2 = 0.0869
Final R indexes [all data]	R_1 = 0.0426, wR_2 = 0.0914
Largest diff. peak/hole / e Å^{-3}	0.27/-0.39

Table S7 Bond Lengths for **10·OTf**.

Atom	Atom	Length/Å	Atom	Atom	Length/Å
S1	O3	1.4403(12)	C6	C7	1.377(2)
S1	O4	1.4391(12)	C8	C9	1.349(2)
S1	O5	1.4444(13)	C9	C10	1.447(2)
S1	C29	1.8244(19)	C10	C11	1.393(2)
F1	C29	1.341(2)	C10	C15	1.400(2)
F2	C29	1.331(2)	C11	C12	1.385(2)
F3	C29	1.333(2)	C12	C13	1.388(2)
P1	C8	1.7464(16)	C13	C14	1.396(2)
P1	C9	1.7394(16)	C14	C15	1.375(2)
P1	C17	1.7790(16)	C17	C18	1.388(2)
P1	C23	1.7848(17)	C17	C22	1.382(2)
O1	C1	1.428(2)	C18	C19	1.384(3)
O1	C2	1.3612(19)	C19	C20	1.375(3)
O2	C13	1.361(2)	C20	C21	1.376(3)
O2	C16	1.427(2)	C21	C22	1.379(3)
C2	C3	1.390(2)	C23	C24	1.387(2)
C2	C7	1.396(2)	C23	C28	1.386(2)
C3	C4	1.382(2)	C24	C25	1.385(3)
C4	C5	1.394(2)	C25	C26	1.374(3)
C5	C6	1.402(2)	C26	C27	1.375(3)
C5	C8	1.449(2)	C27	C28	1.386(3)

Table S8 Bond Angles for **10·OTf**

Atom	Atom	Atom	Angle/˚	Atom	Atom	Atom	Angle/˚
O3	S1	O5	114.91(7)	C9	C8	P1	66.96(9)
O3	S1	C29	102.62(8)	C9	C8	C5	148.69(15)
O4	S1	O3	115.09(8)	C8	C9	P1	67.51(9)
O4	S1	O5	115.13(8)	C8	C9	C10	148.41(15)
O4	S1	C29	103.42(9)	C10	C9	P1	143.87(13)
O5	S1	C29	103.16(8)	C11	C10	C9	119.37(15)
F1	C29	S1	111.11(13)	C11	C10	C15	118.81(15)
F2	C29	S1	111.45(13)	C15	C10	C9	121.82(14)
F2	C29	F1	107.14(16)	C12	C11	C10	121.13(16)
F2	C29	F3	107.69(16)	C11	C12	C13	119.26(15)

F3	C29	S1	111.44(14)	O2	C13	C12	125.14(16)
F3	C29	F1	107.80(16)	O2	C13	C14	114.57(16)
C8	P1	C17	124.75(8)	C12	C13	C14	120.28(15)
C8	P1	C23	119.69(8)	C15	C14	C13	120.01(16)
C9	P1	C8	45.54(7)	C14	C15	C10	120.47(15)
C9	P1	C17	122.10(8)	C18	C17	P1	121.03(13)
C9	P1	C23	117.87(8)	C22	C17	P1	118.40(13)
C17	P1	C23	111.63(7)	C22	C17	C18	120.42(16)
C2	O1	C1	117.86(15)	C19	C18	C17	119.20(17)
C13	O2	C16	117.97(15)	C20	C19	C18	120.39(17)
O1	C2	C3	124.33(16)	C19	C20	C21	120.04(17)
O1	C2	C7	115.69(15)	C20	C21	C22	120.41(17)
C3	C2	C7	119.97(15)	C21	C22	C17	119.53(16)
C4	C3	C2	119.43(15)	C24	C23	P1	120.99(13)
C3	C4	C5	121.24(15)	C28	C23	P1	119.12(12)
C4	C5	C6	118.77(14)	C28	C23	C24	119.72(16)
C4	C5	C8	119.15(14)	C25	C24	C23	119.57(18)
C6	C5	C8	122.08(14)	C26	C25	C24	120.55(18)
C7	C6	C5	120.28(15)	C25	C26	C27	120.05(18)
C6	C7	C2	120.31(16)	C26	C27	C28	120.07(18)
C5	C8	P1	144.09(12)	C27	C28	C23	120.03(16)

Experimental

Single crystals of $C_{29}H_{24}F_3O_5PS$ [**10·OTf**] were grown by slow diffusion in pentane/dichloromethane solution. A suitable crystal was selected and mounted on a SuperNova, Dual, Cu at home/near, EosS2 diffractometer. The crystal was kept at 150.00(10) K during data collection. Using Olex2,[33] the structure was solved with the ShelXT[36] structure solution program using Intrinsic Phasing and refined with the ShelXL[35] refinement package using Least Squares minimisation.

Crystal structure determination of [10·OTf]

Crystal Data for $C_{29}H_{24}F_3O_5PS$ (M =572.51 g/mol): monoclinic, space group $P2_1/n$ (no. 14), a = 10.5938(2) Å, b = 15.2705(2) Å, c = 16.8486(2) Å, β = 95.4560(10)°, V = 2713.29(7) Å^3, Z = 4, T = 150.00(10) K, μ(CuKα) = 2.136 mm^{-1}, $Dcalc$ = 1.402 g/cm^3, 23939 reflections measured (7.83° ≤ 2Θ ≤ 146.092°), 5389 unique (R_{int} = 0.0353, R_{sigma} = 0.0272) which were used in all calculations. The final R_1 was 0.0354 (I > 2σ(I)) and wR_2 was 0.0914 (all data).

Refinement model description

Number of restraints - 0, number of constraints - unknown.

Details:

```
1. Fixed Uiso
 At 1.2 times of:
  All C(H) groups
 At 1.5 times of:
  All C(H,H,H) groups
2.a Aromatic/amide H refined with riding coordinates:
 C3(H3), C4(H4), C6(H6), C7(H7), C11(H11), C12(H12), C14(H14), C15(H15),
 C18(H18), C19(H19), C20(H20), C21(H21), C22(H22), C24(H24), C25(H25),
```

```
C26(H26),
  C27(H27), C28(H28)
2.b Idealised Me refined as rotating group:
 C1(H1A,H1B,H1C), C16(H16A,H16B,H16C)
```

This report has been created with Olex2, compiled on 2018.05.29 svn.r3508 for OlexSys. Please let us know if there are any errors or if you would like to have additional features.

10. Computational Details

DFT calculations were carried out with Gaussian 09 (Revision D.01).[37] Geometry optimizations were carried out at the TPSS/def2-SVP level,[38-39] with the ultrafine integral grid option invoked. Analytical frequency calculations were carried out at the same level of theory to yield thermodynamic free energy corrections to the SCF energy. Minima along the energy profiles were confirmed with no imaginary frequencies, and saddle points were confirmed with identification of one negative eigenvalue upon frequency analysis constituting the relevant bond formation/breaking process. To characterise and confirm the minima either side of the saddle points, the transition state structures were perturbed along the forward and reverse directions of the imaginary frequency, and the resulting structures subsequently optimized. Single point energy corrections were obtained at the M052X-D3(MeCN)/def2-TZVP level of theory,[39-41] which was identified as a reliable functional in a rigorous benchmarking evaluation of main group kinetics, thermodynamics and non-covalent interactions by Grimme.[42] Solvation corrections were carried out using the IEFPCM approximation[43] with acetonitrile as the solvent (where $\varepsilon = 35.688$). Empirical corrections for dispersion interactions were obtained with the zero-damping function.[44]

10.1 Free energy profiles

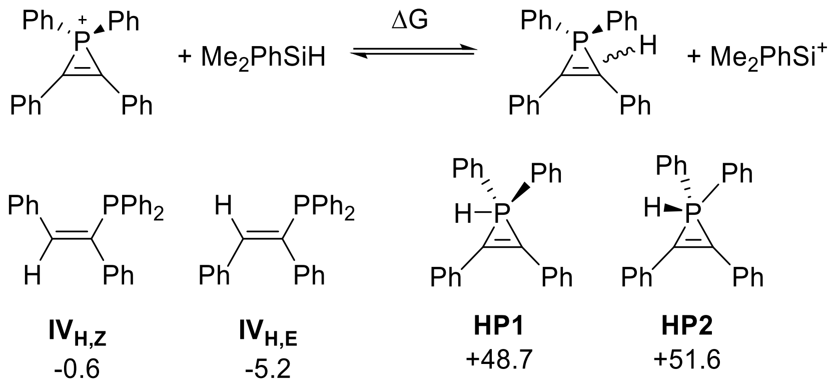

Figure S42. Free energies of species resulting from hydride addition from Me_2PhSiH to phosphirenium species **3**$^+$, either at unsaturated C (**IV**$_{H,Z}$, **IV**$_{H,E}$) or at phosphorus (**HP1**, **HP2**).

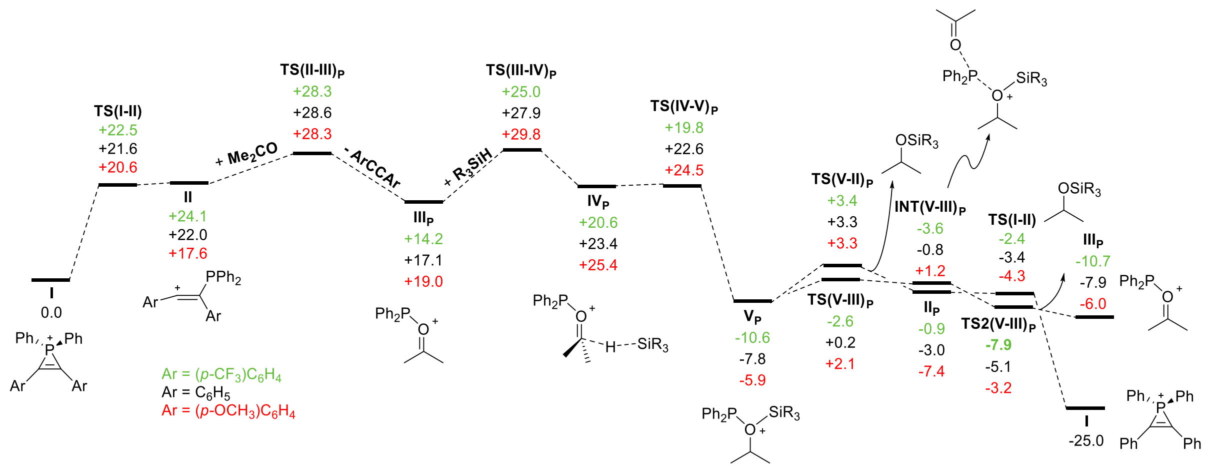

Figure S43. Computed free energy profile of catalytic hydrosilylation of acetone via phosphenium adduct **III**$_P$, with the 3 different substituted phosphirenium precatalysts, computed at the M052X-D3(MeCN)/def2-TZVP//TPSS/def2-SVP level of theory. **INT(V-III)**$_P$ and **TS2(V-III)**$_P$ were located as stationary points on the TPSS/def2-SVP surface but fell below the free energy of the preceding **TS(V-III)**$_P$ upon including the energy corrections. These are therefore omitted from Figure 3 in the main text for clarity but are included here.

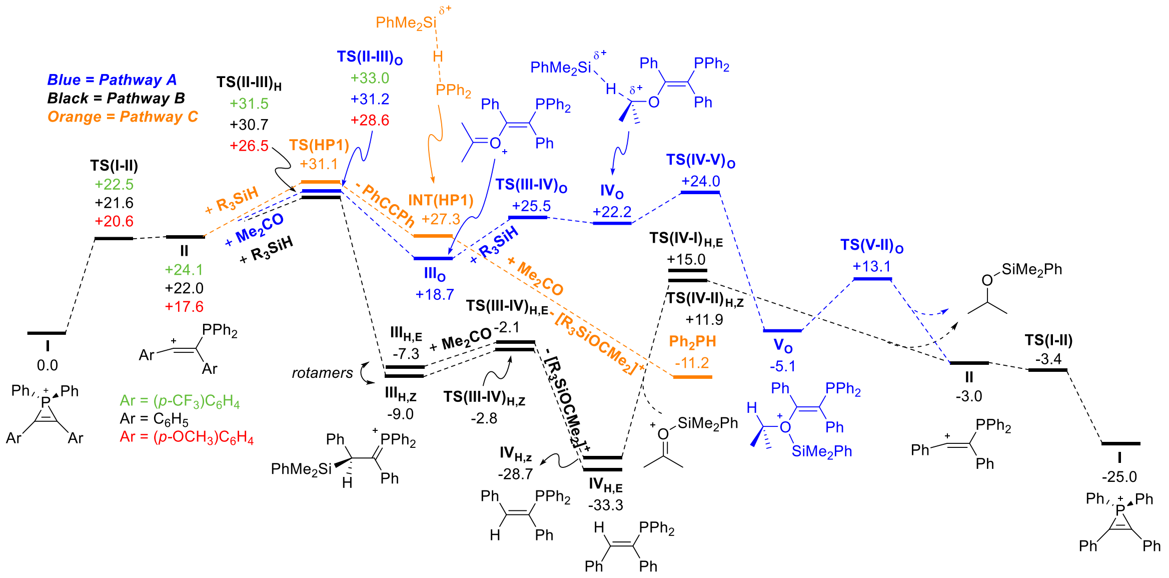

Figure S44. DFT mechanisms of hydrosilylation of acetone computed at the M052X-D3(MeCN)/def2-TZVP//TPSS/def2-SVP level of theory. (A) Initiated by ketone addition to the ring-opened phosphirenium followed by H^- transfer. (B) Initiated by H^- transfer to the phosphirenium, followed by silyl cation transfer and H^- transfer to acetone. Selected species with different para-substituted phosphireniums are also reported (green = *p*-CF_3, red = *p*-OMe). (C) Side process of hydride transfer to P to form Ph_2PH.

Note: Characterization of the product of **TS(II-III)$_{H,OMe}$**, species **III$_{H,OMe}$** (-30.8 kcal/mol) reveals that the Me_2PhSi^+ cation migrates to the phosphorus centre, as opposed to the C1 position seen in **III$_{H,Z}$**. Characterization of the subsequent silyl-transfer process via **TS(III-IV)$_{H,OMe}$** (-24.7 kcal/mol) however reveals this intermediate can also lead to formation of the corresponding vinylphosphine.

10.2 Computed Cartesian coordinates (Å) and energies (au) for all species.

PhCCPh$_{CF3}$

```
SCF (TPSS/def2-SVP) Energy = -1212.81924508
Enthalpy 0K = -1212.603000
Enthalpy 298K = -1212.602056
Free Energy 298K = -1212.674714
Lowest Frequency = 9.8325 cm-1
Second Frequency = 17.0134 cm-1
SCF (M052X-D3,MeCN/def2-TZVP) Energy = -
1213.94968838

C   2.03963  0.00001 -0.03069
C  -0.61430  0.00001 -0.03154
C  -2.03963  0.00001 -0.03069
C   0.61430  0.00001 -0.03154
C   2.76339  1.22010 -0.03272
C   2.76340 -1.22008 -0.03236
C   4.15926  1.21719 -0.03243
C   4.86296  0.00002 -0.02968
C   4.15927 -1.21716 -0.03208
C  -2.76338  1.22010 -0.03238
C  -4.86296  0.00003 -0.02971
C  -2.76340 -1.22007 -0.03272
C  -4.15925  1.21720 -0.03211
C  -4.15927 -1.21715 -0.03246
H   4.70982  2.16252 -0.04164
H   2.21548  2.16676 -0.03757
H   2.21550 -2.16675 -0.03695
H   4.70982 -2.16249 -0.04102
H  -2.21551 -2.16674 -0.03758
H  -4.70982 -2.16248 -0.04170
H  -4.70981  2.16253 -0.04106
H  -2.21548  2.16677 -0.03696
C   6.37196  0.00000  0.02478
F   6.82699 -0.00051  1.30493
F   6.89899 -1.09317 -0.57857
F   6.89902  1.09360 -0.57774
C  -6.37196 -0.00000  0.02479
F  -6.82698 -0.00062  1.30494
F  -6.89904  1.09365 -0.57761
F  -6.89900 -1.09312 -0.57865
```

PhCCPh$_{OMe}$

```
SCF (TPSS/def2-SVP) Energy = -768.103251506
Enthalpy 0K = -767.834050
Enthalpy 298K = -767.833105
Free Energy 298K = -767.897058
Lowest Frequency = 19.3015 cm-1
Second Frequency = 27.7889 cm-1
SCF (M052X-D3,MeCN/def2-TZVP) Energy = -
768.721205610

C   2.03667 -0.11424  0.00001
C  -0.61406  0.03315  0.00000
C  -2.03667  0.11413  0.00000
C   0.61406 -0.03334  0.00001
C   2.70003 -1.37223 -0.00017
C   2.83697  1.05332  0.00019
C   4.08918 -1.45168 -0.00017
C   4.87209 -0.27649  0.00001
C   4.23491  0.98067  0.00019
C  -2.83703 -1.05338  0.00023
C  -4.87208  0.27654 -0.00001
C  -2.69996  1.37216 -0.00023
C  -4.23496 -0.98066  0.00023
C  -4.08911  1.45168 -0.00024
H   4.60239 -2.41816 -0.00032
H   2.10254 -2.28895 -0.00032
H   2.34942  2.03306  0.00033
H   4.81646  1.90641  0.00034
H  -2.10242  2.28885 -0.00042
H  -4.60227  2.41819 -0.00043
H  -4.81656 -1.90637  0.00043
H  -2.34953 -2.03315  0.00043
O  -6.22369  0.46036 -0.00002
C  -7.06698 -0.68412 -0.00013
H  -6.90747 -1.30645 -0.90207
H  -6.90794 -1.30629  0.90201
H  -8.09840 -0.30117 -0.00042
O   6.22371 -0.46025 -0.00000
C   7.06694  0.68428  0.00012
H   6.90760  1.30663 -0.90184
H   6.90767  1.30640  0.90224
H   8.09838  0.30138  0.00003
```

PhCCPh

```
SCF (TPSS/def2-SVP) Energy = -539.190140599
Enthalpy 0K = -538.989983
Enthalpy 298K = -538.989039
Free Energy 298K = -539.040598
Lowest Frequency = 22.7560 cm-1
Second Frequency = 47.0706 cm-1
SCF (M052X-D3,MeCN/def2-TZVP) Energy = -
539.605279413

C  -2.04116  0.00006  0.00000
C   0.61448  0.00013 -0.00000
C   2.04116  0.00006  0.00000
C  -0.61448  0.00013 -0.00000
C  -2.76538  1.22009  0.00008
C  -2.76525 -1.22005 -0.00008
C  -4.16383  1.21414  0.00008
C  -4.86895 -0.00009  0.00000
C  -4.16370 -1.21424 -0.00008
C   2.76538  1.22009 -0.00008
C   4.86895 -0.00009 -0.00000
C   2.76525 -1.22005  0.00008
C   4.16383  1.21413 -0.00008
C   4.16370 -1.21424  0.00008
H  -5.96425 -0.00015  0.00000
H  -4.70891  2.16440  0.00015
H  -2.21459  2.16564  0.00015
H  -2.21436 -2.16554 -0.00015
H  -4.70869 -2.16457 -0.00015
H   2.21436 -2.16554  0.00014
H   4.70869 -2.16457  0.00014
H   5.96425 -0.00015 -0.00000
H   4.70891  2.16440 -0.00015
H   2.21459  2.16564 -0.00015
```

Me$_2$PhSiH

```
SCF (TPSS/def2-SVP) Energy = -601.368451981
Enthalpy 0K = -601.186946
Enthalpy 298K = -601.186002
Free Energy 298K = -601.234310
Lowest Frequency = 19.7474 cm-1
Second Frequency = 86.3151 cm-1
SCF (M052X-D3,MeCN/def2-TZVP) Energy = -
601.675623117

H   1.87993 -1.92172  0.00686
Si  1.65096 -0.44300  0.00155
C   2.43477  0.31477  1.55241
H   3.52835  0.15097  1.56443
H   2.25718  1.40533  1.59725
H   2.01240 -0.13280  2.47014
C  -0.22586 -0.17329  0.00069
C  -0.78004  1.12864 -0.00282
C  -1.12261 -1.26600  0.00346
C  -2.16736  1.33163 -0.00354
C  -2.51236 -1.06989  0.00275
C  -3.03757  0.23036 -0.00075
H  -0.11945  2.00562 -0.00505
H  -0.72862 -2.28986  0.00624
H  -2.57166  2.35035 -0.00627
H  -3.18654 -1.93400  0.00494
H  -4.12221  0.38647 -0.00129
```

```
C   2.43446  0.30357 -1.55489
H   2.01152 -0.15026 -2.46928
H   2.25718  1.39384 -1.60728
H   3.52798  0.13935 -1.56626
```

Me2PhSi

```
SCF (TPSS/def2-SVP) Energy = -600.501347693
Enthalpy 0K = -600.328320
Enthalpy 298K = -600.327375
Free Energy 298K = -600.376909
Lowest Frequency = 21.3677 cm-1
Second Frequency = 57.6274 cm-1
SCF (M052X-D3,MeCN/def2-TZVP) Energy = -
600.880633861

Si  1.59149  0.00000 -0.00002
C   2.56000 -1.57862 -0.00013
H   3.22012 -1.60438 -0.88919
H   3.22474 -1.60199  0.88550
H   1.93165 -2.48307  0.00260
C  -0.22100  0.00003 -0.00003
C  -0.94662  1.22972 -0.00016
C  -0.94659 -1.22969  0.00013
C  -2.34150  1.22366 -0.00012
C  -2.34146 -1.22368  0.00014
C  -3.03649 -0.00002  0.00002
H  -0.41684  2.18811 -0.00029
H  -0.41678 -2.18806  0.00023
H  -2.89355  2.16844 -0.00021
H  -2.89349 -2.16847  0.00025
H  -4.13163 -0.00003  0.00004
C   2.56005  1.57860  0.00016
H   1.93167  2.48304 -0.00290
H   3.21982  1.60450  0.88947
H   3.22510  1.60187 -0.88523
```

Me2PhSiOCHMe2

```
SCF (TPSS/def2-SVP) Energy = -794.459186104
Enthalpy 0K = -794.182818
Enthalpy 298K = -794.181874
Free Energy 298K = -794.242970
Lowest Frequency = 22.7159 cm-1
Second Frequency = 33.7040 cm-1
SCF (M052X-D3,MeCN/def2-TZVP) Energy = -
794.947449464

H   3.32445  1.58076 -0.28362
Si  0.83135 -1.07485 -0.14928
C   1.29499 -1.96229  1.45699
H   1.22543 -1.31436  2.34883
H   0.64011 -2.83810  1.62449
H   2.33586 -2.32944  1.39248
C   0.94818 -2.28320 -1.59029
H   1.98239 -2.65922 -1.69464
H   0.27963 -3.15054 -1.43869
H   0.67041 -1.80227 -2.54544
C   2.24910  1.43050 -0.06332
O   1.95520  0.11451 -0.54773
C   1.44972  2.47180 -0.85523
C   2.05109  1.56529  1.45071
H   1.60864  2.32711 -1.93732
H   2.65649  0.81988  1.99406
H   1.77253  3.49456 -0.58911
H   0.36943  2.38159 -0.64586
H   0.98884  1.42767  1.72456
H   2.36028  2.57012  1.78976
C  -0.93599 -0.38473 -0.01968
C  -1.65178 -0.36946  1.19971
C  -1.59173  0.12809 -1.16490
C  -2.95894  0.13660  1.27668
C  -2.89698  0.63540 -1.09623
C  -3.58439  0.64114  0.12765
H  -1.18488 -0.76202  2.11121
H  -1.07316  0.13396 -2.13188
H  -3.49044  0.13548  2.23522
H  -3.38105  1.02574 -1.99880
H  -4.60502  1.03608  0.18406
```

Me2PhSiOCMe2

```
SCF (TPSS/def2-SVP) Energy = -793.617865309
Enthalpy 0K = -793.353222
Enthalpy 298K = -793.352278
Free Energy 298K = -793.415659
Lowest Frequency = 12.1007 cm-1
Second Frequency = 43.6675 cm-1
SCF (M052X-D3,MeCN/def2-TZVP) Energy = -
794.153181615

Si  0.59052 -1.22688 -0.00879
C   1.02711 -2.15698  1.54908
H   0.91012 -1.54270  2.45892
H   0.35758 -3.03160  1.64726
H   2.06641 -2.52663  1.50977
C   1.02931 -2.09512 -1.60208
H   2.06659 -2.47130 -1.57330
H   0.35671 -2.96255 -1.73712
H   0.91877 -1.44505 -2.48750
C   2.17738  1.28096  0.00118
O   1.95103  0.03804  0.01900
C   3.60093  1.71475  0.08086
C   1.09047  2.29640 -0.10988
H   4.28957  0.87050 -0.06238
H   0.13984  1.93726  0.31197
H   3.76449  2.15679  1.08481
H   3.79785  2.52431 -0.64416
H   0.92598  2.48093 -1.19191
H   1.39869  3.25442  0.33710
C  -1.06536 -0.37468  0.00679
C  -1.70295 -0.04566  1.23050
C  -1.73829 -0.07133 -1.20391
C  -2.95859  0.57632  1.24133
C  -2.99459  0.54888 -1.19127
C  -3.60335  0.87565  0.03069
H  -1.22346 -0.28386  2.18735
H  -1.28653 -0.32951 -2.16894
H  -3.43972  0.81908  2.19435
H  -3.50391  0.77036 -2.13481
H  -4.58707  1.35651  0.03992
```

Me2CO

```
SCF (TPSS/def2-SVP) Energy = -193.043437406
Enthalpy 0K = -192.955757
Enthalpy 298K = -192.954813
Free Energy 298K = -192.989946
Lowest Frequency = 35.5451 cm-1
Second Frequency = 133.4227 cm-1
SCF (M052X-D3,MeCN/def2-TZVP) Energy = -
193.203450913

C   0.00001  0.18492  0.00036
O   0.00008  1.40468 -0.00009
C   1.29699 -0.61619 -0.00001
C  -1.29706 -0.61608 -0.00005
H   2.15639  0.07076  0.00123
H  -2.15637  0.07097 -0.00061
H   1.34436 -1.27248 -0.88876
H   1.34373 -1.27514  0.88679
H  -1.34464 -1.27348  0.88787
H  -1.34374 -1.27392 -0.88768
```

Ph2PH

```
SCF (TPSS/def2-SVP) Energy =) = -
804.930683625
Enthalpy 0K = -804.730834
Enthalpy 298K = -804.729890
Free Energy 298K = -804.782113
Lowest Frequency = 13.3960 cm-1
Second Frequency = 27.3877 cm-1
```

SCF (M052X-D3,MeCN/def2-TZVP) Energy =-
805.379767650

P	-0.05789	1.69287	-0.09208
C	1.42987	0.59826	-0.01409
C	-1.46307	0.46826	-0.08361
C	1.81276	0.06704	1.23760
C	-2.71538	0.92919	0.37213
C	-1.34064	-0.87509	-0.49414
C	2.24084	0.32900	-1.13699
C	-2.44979	-1.73186	-0.46005
C	2.96101	-0.72638	1.35782
C	3.76043	-0.98586	0.23315
C	-3.69595	-1.25919	-0.01876
C	3.39991	-0.45250	-1.01256
C	-3.82741	0.07375	0.39611
H	-4.79503	0.44764	0.74909
H	-2.81728	1.96648	0.71313
H	-0.37165	-1.25257	-0.83911
H	-2.34120	-2.77392	-0.78154
H	-4.56088	-1.93122	0.00686
H	1.20494	0.27815	2.12558
H	3.23903	-1.13587	2.33538
H	4.66286	-1.59944	0.32907
H	4.01887	-0.64962	-1.89504
H	1.96282	0.73243	-2.11751
H	-0.00963	1.91721	-1.50434

Ph_2P^+

SCF (TPSS/def2-SVP) Energy = -804.063067655
Enthalpy 0K = -803.871706
Enthalpy 298K = -803.870762
Free Energy 298K = -803.920656
Lowest Frequency = 42.9256 cm-1
Second Frequency = 50.1092 cm-1
SCF (M052X-D3,MeCN/def2-TZVP) Energy = -
804.560614157

P	0.00000	1.44634	0.00000
C	1.44532	0.42022	0.04147
C	-1.44532	0.42022	-0.04147
C	1.54346	-0.93044	0.50296
C	-2.64033	1.10768	0.35316
C	-1.54346	-0.93044	-0.50296
C	2.64033	1.10768	-0.35316
C	-2.77907	-1.56979	-0.52019
C	2.77907	-1.56979	0.52019
C	3.93462	-0.89226	0.07320
C	-3.93462	-0.89226	-0.07320
C	3.86565	0.44420	-0.36248
C	-3.86565	0.44420	0.36248
H	-4.77090	0.96475	0.68898
H	-2.58107	2.15670	0.66642
H	-0.66010	-1.44487	-0.89101
H	-2.85996	-2.59492	-0.89477
H	-4.90128	-1.40687	-0.09028
H	0.66010	-1.44487	0.89101
H	2.85996	-2.59492	0.89477
H	4.90128	-1.40687	0.09028
H	4.77090	0.96475	-0.68898
H	2.58107	2.15670	-0.66642

I

SCF (TPSS/def2-SVP) Energy = -1343.33050470
Enthalpy 0K = -1342.934988
Enthalpy 298K = -1342.934044
Free Energy 298K = -1343.014478
Lowest Frequency = 21.5262 cm-1
Second Frequency = 22.2105 cm-1
SCF (M052X-D3,MeCN/def2-TZVP) Energy = -
1344.24469472

P	-0.68927	-0.00000	-0.00000
C	1.69470	-1.91055	-0.22177
C	-1.67875	0.22683	-1.49291
C	0.94709	0.67787	0.07990
C	1.69468	1.91057	0.22179
C	0.94710	-0.67785	-0.07988
C	-1.67878	-0.22685	1.49289
C	3.10228	-1.94706	-0.05832
C	1.00314	-3.10977	-0.52383
C	-2.89898	-0.46722	-1.64972
C	-1.18965	-1.05741	2.52410
C	-2.89895	0.46728	1.64973
C	3.79259	-3.15352	-0.20078
C	3.09722	-4.33483	-0.50979
C	-1.18956	1.05730	-2.52416
C	1.70218	-4.31078	-0.67031
C	3.10226	1.94709	0.05837
C	3.09716	4.33486	0.50982
C	1.00310	3.10979	0.52383
C	3.79256	3.15356	0.20084
C	-3.62429	0.32228	2.83926
C	-3.62430	-0.32223	-2.83926
C	-3.14060	0.50606	-3.86400
C	-3.14066	-0.50610	3.86396
C	-1.92664	1.19371	-3.70663
C	-1.92675	-1.19383	3.70655
C	1.70213	4.31080	0.67032
H	3.64425	-5.27632	-0.62308
H	4.87899	-3.17548	-0.06933
H	3.64742	-1.03369	0.19437
H	-0.08483	-3.08658	-0.64961
H	1.16073	-5.23135	-0.90955
H	-1.55349	-1.84158	4.50594
H	-0.24287	-1.59429	2.40207
H	-3.27944	1.11627	0.85390
H	-4.56886	0.86055	2.96616
H	-3.71328	-0.61703	4.79039
H	-3.27953	-1.11614	-0.85386
H	-4.56892	-0.86043	-2.96613
H	-3.71321	0.61699	-4.79044
H	-1.55333	1.84139	-4.50605
H	-0.24274	1.59412	-2.40215
H	-0.08487	3.08658	0.64959
H	1.16066	5.23137	0.90954
H	3.64418	5.27637	0.62312
H	4.87896	3.17552	0.06940
H	3.64741	1.03373	-0.19430

TS(I-II)

SCF (TPSS/def2-SVP) Energy = -1343.29823836
Enthalpy 0K = -1342.904883
Enthalpy 298K = -1342.903939
Free Energy 298K = -1342.983862
Lowest Frequency = -211.1003 cm-1
Second Frequency = 19.9570 cm-1
SCF (M052X-D3,MeCN/def2-TZVP) Energy = -
1344.20866752

P	-0.24060	0.32250	-1.08371
C	0.70657	-0.65761	1.88238
C	0.95234	-0.91803	-1.66341
C	-1.41174	-0.25441	0.25438
C	-2.86799	-0.34895	0.19296
C	-0.35095	-0.39532	0.97741
C	0.49714	1.94796	-0.74670
C	1.23134	0.37581	2.70937
C	1.24751	-1.97301	1.96681
C	0.49502	-1.87046	-2.60167
C	-0.13917	2.84064	0.14456
C	1.61224	2.37778	-1.49870
C	2.26289	0.08728	3.60409
C	2.78337	-1.21611	3.69009
C	2.29529	-0.94054	-1.22357
C	2.27407	-2.24094	2.87386
C	-3.57203	-0.81477	1.32899
C	-5.66464	-0.55022	0.12052
C	-3.57244	0.01422	-0.97285
C	-4.96519	-0.91295	1.28460
C	2.10310	3.67996	-1.32827

```
C   1.37586 -2.84637 -3.08490
C   2.71150 -2.86342 -2.65399
C   1.48453  4.55928 -0.42803
C   3.16950 -1.91130 -1.72904
C   0.36254  4.13832  0.30372
C  -4.96774 -0.08697 -1.00597
H   2.66099  0.87985  4.24532
H   0.81588  1.38495  2.64290
H   0.84437 -2.76403  1.32822
H   2.67983 -3.25473  2.94832
H  -0.13095  4.82558  0.99851
H  -1.02414  2.52812  0.70963
H   2.09322  1.70759 -2.21810
H   2.97161  4.00683 -1.90893
H   1.87032  5.57584 -0.30217
H  -0.54178 -1.84458 -2.95373
H   1.02124 -3.58554 -3.81016
H   3.40055 -3.61953 -3.04409
H   4.21177 -1.92727 -1.39428
H   2.65376 -0.20609 -0.49538
H  -3.01929  0.37600 -1.84740
H  -5.51245  0.19646 -1.91187
H  -5.51034 -1.27483  2.16213
H  -3.02461 -1.09704  2.23388
H  -6.75616 -0.62940  0.09323
H   3.58777 -1.43475  4.39984
```

II

```
SCF (TPSS/def2-SVP) Energy = -1343.29996353
Enthalpy 0K = -1342.905511
Enthalpy 298K = -1342.904567
Free Energy 298K = -1342.985488
Lowest Frequency = 14.5510 cm-1
Second Frequency = 29.3782 cm-1
SCF (M052X-D3,MeCN/def2-TZVP) Energy = -
1344.20813629

P  -0.37691  0.53455 -1.20784
C   0.73779 -1.03035  1.71215
C   1.03018 -0.48415 -1.76804
C  -1.35521 -0.40849  0.15580
C  -2.81539 -0.56401  0.09009
C  -0.38331 -0.71402  0.94469
C   0.25704  2.06819 -0.45471
C   1.19562 -0.13813  2.73325
C   1.42505 -2.26710  1.49411
C   0.75361 -1.40461 -2.80618
C  -0.42506  2.67880  0.62347
C   1.30993  2.76587 -1.09237
C   2.30309 -0.48234  3.50422
C   2.96361 -1.70680  3.28490
C   2.34733 -0.36946 -1.26694
C   2.52435 -2.59436  2.28429
C  -3.48126 -1.23823  1.14212
C  -5.60643 -0.89084  0.01574
C  -3.56065 -0.05748 -0.99365
C  -4.86797 -1.39834  1.09918
C   1.69344  4.03227 -0.63088
C   1.77806 -2.20620 -3.32475
C   3.08549 -2.08202 -2.82876
C   1.02921  4.62155  0.45475
C   3.36821 -1.16434 -1.80422
C  -0.03143  3.94388  1.07720
C  -4.95105 -0.22147 -1.02776
H   2.65490  0.19634  4.28725
H   0.66613  0.80424  2.89776
H   1.07060 -2.94500  0.71276
H   3.04566 -3.54362  2.12714
H  -0.56159  4.40578  1.91652
H  -1.26544  2.16947  1.10757
H   1.82507  2.32841 -1.95383
H   2.51284  4.56129 -1.12808
H   1.33029  5.61222  0.81003
H  -0.26144 -1.48483 -3.21135
H   1.55871 -2.91642 -4.12833
H   3.88860 -2.69770 -3.24693
H   4.38919 -1.06733 -1.42062
H   2.57363  0.34217 -0.46658
H  -3.04643  0.46270 -1.80968
H  -5.52289  0.17475 -1.87266
H  -5.37793 -1.92210  1.91402
H  -2.90579 -1.63326  1.98590
H  -6.69327 -1.01896 -0.01281
H   3.82728 -1.97325  3.90320
```

TS(II-III)$_H$

```
SCF (TPSS/def2-SVP) Energy = -1944.66773104
Enthalpy 0K = -1944.090799
Enthalpy 298K = -1944.089855
Free Energy 298K = -1944.199476
Lowest Frequency = -115.8665 cm-1
Second Frequency = 9.1918 cm-1
SCF (M052X-D3,MeCN/def2-TZVP) Energy = -
1945.88949621

P  -2.34715  0.08094  1.14519
C   0.30670 -0.97752 -0.93358
C  -1.80548 -1.64069  1.46961
C  -0.90302  1.03238  0.32694
C  -0.90497  2.50196  0.48864
C  -0.10302  0.21757 -0.30941
C  -3.61887 -0.02546 -0.16744
C  -0.25018 -1.32940 -2.20112
C   1.25799 -1.84417 -0.31855
C  -1.11320 -1.86039  2.68341
C  -3.57747  0.75907 -1.34093
C  -4.76662 -0.81249  0.09413
C   0.12083 -2.52519 -2.81359
C   1.05660 -3.37374 -2.19225
C  -2.10990 -2.74042  0.63733
C   1.62208 -3.03229 -0.95036
C  -0.46047  3.32295 -0.57469
C  -0.89641  5.30600  0.76184
C  -1.36294  3.10367  1.68022
C  -0.45935  4.71577 -0.43498
C  -5.83126 -0.83224 -0.81502
C  -0.71770 -3.15380  3.04709
C  -1.02945 -4.24230  2.21665
C  -5.77519 -0.06077 -1.98639
C  -1.72653 -4.03418  1.01631
C  -4.64939  0.73519 -2.24423
C  -1.34749  4.49732  1.81640
H  -0.31154 -2.80001 -3.78078
H  -0.97210 -0.65746 -2.67403
H   1.69253 -1.56898  0.64532
H   2.35253 -3.69618 -0.47854
H  -4.60439  1.34500 -3.15266
H  -2.71098  1.39427 -1.55074
H  -4.83070 -1.40844  1.01154
H  -6.71101 -1.44869 -0.60335
H  -6.61085 -0.07537 -2.69348
H  -0.89860 -1.01648  3.34959
H  -0.18568 -3.31563  3.99034
H  -0.73830 -5.25588  2.51178
H  -1.97554 -4.88386  0.37200
H  -2.65378 -2.58600 -0.29979
H  -1.72295  2.47416  2.50083
H  -1.69389  4.95354  2.74919
H  -0.12805  5.34413 -1.26836
H  -0.14224  2.86348 -1.51591
Si  2.90710  1.81646 -0.23800
C   2.89866  2.85511  1.33522
H   2.60431  2.26641  2.22208
H   3.90578  3.27152  1.52246
H   2.19134  3.69766  1.24017
C   3.23700  2.83910 -1.78957
H   4.27043  3.23185 -1.76969
H   3.11837  2.24789 -2.71494
H   2.54892  3.70114 -1.84508
H   1.48329  1.21978 -0.39179
C   4.06104  0.32901 -0.11028
C   4.44656 -0.19404  1.14895
```

```
C   4.57583 -0.29941 -1.27087
C   5.31229 -1.29282  1.24493
C   5.44283 -1.39723 -1.17876
C   5.81181 -1.89665  0.08003
H   4.08029  0.27103  2.07210
H   4.30782  0.07850 -2.26470
H   5.60787 -1.67174  2.22932
H   5.83873 -1.85890 -2.08983
H   6.49548 -2.74931  0.15383
H  -0.89754  6.39573  0.86715
H   1.35060 -4.30717 -2.68388
```

$III_{H,Z}$

SCF (TPSS/def2-SVP) Energy = -1944.71702019
Enthalpy 0K = -1944.134916
Enthalpy 298K = -1944.133972
Free Energy 298K = -1944.238208
Lowest Frequency = 16.4661 cm-1
Second Frequency = 22.8316 cm-1
SCF (M052X-D3,MeCN/def2-TZVP) Energy = -1945.96322655

```
P  -0.85003 -0.56561  0.71536
C   1.00813 -0.20469 -2.08213
C   0.33985 -1.93331  0.62822
C  -0.75532  0.72487 -0.47385
C  -1.86528  1.69727 -0.43110
C   0.39855  0.99129 -1.35336
C  -2.53317 -1.25002  0.83261
C   0.28080 -0.75427 -3.16042
C   2.25547 -0.76926 -1.75968
C   1.52981 -1.86416  1.38480
C  -3.42965 -1.26928 -0.25833
C  -2.91236 -1.82766  2.06463
C   0.79032 -1.83040 -3.89987
C   2.03789 -2.38008 -3.56986
C   0.02125 -3.10739 -0.09117
C   2.76633 -1.84813 -2.49647
C  -2.34578  2.27806 -1.63540
C  -4.03864  3.51248 -0.40486
C  -2.49827  2.06154  0.78778
C  -3.42580  3.16615 -1.61916
C  -4.17404 -2.42101  2.19935
C   2.40959 -2.95504  1.39167
C   2.10399 -4.11192  0.66021
C  -5.06496 -2.43396  1.11548
C   0.91142 -4.18651 -0.07912
C  -4.69130 -1.85899 -0.10959
C  -3.56747  2.95944  0.79795
H   0.21439 -2.23462 -4.73906
H  -0.69081 -0.32560 -3.43503
H   2.83165 -0.37621 -0.91681
H   3.74004 -2.27201 -2.22997
H  -5.38223 -1.87765 -0.95880
H  -3.14252 -0.83108 -1.21894
H  -2.22204 -1.81569  2.91522
H  -4.46311 -2.86852  3.15569
H  -6.05196 -2.89504  1.22413
H   1.76331 -0.96734  1.96655
H   3.33082 -2.90186  1.98062
H   2.79060 -4.96487  0.67375
H   0.67188 -5.09145 -0.64676
H  -0.91153 -3.17283 -0.65966
H  -2.11047  1.66395  1.73193
H  -4.02710  3.24345  1.74990
H  -3.79362  3.58696 -2.56036
H  -1.90262  1.99253 -2.59507
Si  1.68993  2.31733 -0.55150
C   0.71458  3.90631 -0.26491
H  -0.06732  3.81539  0.50784
H   1.42196  4.68871  0.06806
H   0.23400  4.26266 -1.19368
C   2.99318  2.60950 -1.88242
H   3.67578  3.41062 -1.54266
H   3.59758  1.71918 -2.12077
H   2.51488  2.95179 -2.81786
H   0.03757  1.71809 -2.11139
C   2.42689  1.70838  1.07475
C   1.68545  1.79575  2.27983
C   3.75359  1.21996  1.15261
C   2.24215  1.40400  3.50683
C   4.31421  0.82928  2.37748
C   3.55766  0.91745  3.55663
H   0.66447  2.19506  2.26846
H   4.37010  1.15815  0.24794
H   1.65357  1.48866  4.42667
H   5.34718  0.46696  2.41415
H   3.99686  0.61967  4.51471
H  -4.87588  4.21764 -0.39400
H   2.44160 -3.21693 -4.14908
```

$TS(III\text{-}IV)_{H,Z}$

SCF (TPSS/def2-SVP) Energy = -2137.76670751
Enthalpy 0K = -2137.094735
Enthalpy 298K = -2137.093791
Free Energy 298K = -2137.216326
Lowest Frequency = -114.8436 cm-1
Second Frequency = 5.7130 cm-1
SCF (M052X-D3,MeCN/def2-TZVP) Energy = -2139.17496077

```
P  -1.57857 -0.06813 -0.82152
C   0.08478  2.16968  1.11601
C  -2.72524  1.35172 -0.51039
C  -0.72876 -0.29869  0.80156
C  -0.84218 -1.59658  1.51112
C   0.06261  0.70767  1.39337
C  -2.75927 -1.47652 -1.00935
C   0.15523  3.04549  2.22612
C   0.06656  2.73475 -0.17934
C  -3.16978  2.05212 -1.65168
C  -3.84607 -1.71718 -0.14210
C  -2.58907 -2.29644 -2.14496
C   0.17964  4.43358  2.05150
C   0.15782  4.97924  0.75839
C  -3.21509  1.72035  0.75955
C   0.10676  4.12426 -0.35280
C  -1.09259 -1.60341  2.90761
C  -1.09167 -4.03283  2.93913
C  -0.73429 -2.84357  0.84820
C  -1.22484 -2.80814  3.60950
C  -3.47747 -3.35237 -2.39917
C  -4.10027  3.09260 -1.52557
C  -4.58249  3.45492 -0.25916
C  -4.54548 -3.59309 -1.52369
C  -4.13685  2.76926  0.88113
C  -4.72904 -2.77349 -0.39741
C  -0.84219 -4.04410  1.55735
H   0.21806  5.09088  2.92639
H   0.17803  2.62876  3.24026
H   0.03424  2.08528 -1.05921
H   0.09127  4.54153 -1.36511
H  -5.56905 -2.95521  0.28140
H  -4.00736 -1.07556  0.73028
H  -1.76015 -2.09966 -2.83510
H  -3.33946 -3.98084 -3.28541
H  -5.24266 -4.41403 -1.72196
H  -2.78922  1.77794 -2.64233
H  -4.44445  3.62460 -2.41877
H  -5.30516  4.27154 -0.15999
H  -4.51145  3.04940  1.87144
H  -2.87716  1.19168  1.65712
H  -0.54565 -2.87252 -0.22812
H  -0.73780 -4.99599  1.02659
H  -1.43883 -2.78816  4.68311
H  -1.22600 -0.65469  3.43825
Si  2.36295  0.04408  0.84172
C   2.41201 -1.58324  1.77087
H   2.24754 -2.45216  1.11357
H   3.41991 -1.66562  2.21320
H   1.66851 -1.62510  2.58393
C   3.10496  1.51209  1.74243
```

```
H   4.18943  1.33112  1.83185
H   2.93227  2.46657  1.22044
H   2.68297  1.59659  2.75825
H   0.34995  0.46606  2.43176
C   2.26125  0.07221 -1.02016
C   1.82205 -1.06021 -1.74590
C   2.69450  1.20697 -1.74738
C   1.82963 -1.06578 -3.14787
C   2.69942  1.20299 -3.14910
C   2.26944  0.06588 -3.85147
H   1.48729 -1.95772 -1.21353
H   3.04136  2.10102 -1.21782
H   1.49365 -1.95458 -3.69265
H   3.04317  2.08771 -3.69554
H   2.27681  0.06243 -4.94661
H  -1.19067 -4.97473  3.48831
H   0.18075  6.06486  0.61809
C   5.77412 -0.91071 -0.15567
O   4.83167 -0.53157  0.54650
C   7.17149 -0.95826  0.42019
C   5.59696 -1.34674 -1.59132
H   7.14570 -0.82081  1.51097
H   6.41402 -0.95743 -2.22279
H   7.77110 -0.14648 -0.03422
H   7.67432 -1.90627  0.16002
H   4.61996 -1.03858 -1.98809
H   5.66893 -2.45091 -1.62836
```

$IV_{H,Z}$

```
SCF (TPSS/def2-SVP) Energy = -1344.15940288
Enthalpy 0K = -1343.753993
Enthalpy 298K = -1343.753048
Free Energy 298K = -1343.832208
Lowest Frequency = 13.1714 cm-1
Second Frequency = 32.6268 cm-1
SCF (M052X-D3,MeCN/def2-TZVP) Energy = -
1345.04211759

P  -0.49145  0.60704 -1.08270
C   1.24311 -1.81507  0.93191
C   1.33771  0.64463 -1.32220
C  -0.94043 -0.95623 -0.19750
C  -2.38904 -1.29179 -0.35934
C  -0.13829 -1.87269  0.43683
C  -0.85571  1.94620  0.13680
C   1.84472 -0.64092  1.44315
C   1.99013 -3.01869  0.98380
C   1.91468 -0.38017 -2.10561
C  -1.18126  1.70356  1.48767
C  -0.89306  3.27636 -0.34065
C   3.14758 -0.66633  1.95371
C   3.88303 -1.86161  1.96870
C   2.15255  1.71900 -0.91012
C   3.29621 -3.04114  1.48379
C  -3.09511 -1.88576  0.71554
C  -5.13321 -1.98340 -0.60562
C  -3.10127 -1.03902 -1.55617
C  -4.44652 -2.23130  0.59260
C  -1.20987  4.33647  0.51901
C   3.27440 -0.34697 -2.43604
C   4.07858  0.72545 -2.01742
C  -1.52557  4.08313  1.86335
C   3.51056  1.75938 -1.26043
C  -1.51737  2.76497  2.34179
C  -4.45335 -1.38458 -1.67686
H   3.59033  0.25458  2.34839
H   1.27262  0.29027  1.46468
H   1.53248 -3.94436  0.61528
H   3.85675 -3.98238  1.50212
H  -1.77139  2.55872  3.38754
H  -1.17635  0.67715  1.86995
H  -0.67733  3.47880 -1.39655
H  -1.22581  5.36229  0.13425
H  -1.78564  4.91040  2.53272
H   1.29354 -1.21444 -2.45242
H   3.70735 -1.15915 -3.03060
H   5.14062  0.75510 -2.28415
H   4.12844  2.60225 -0.93041
H   1.72696  2.52703 -0.30549
H  -2.57755 -0.57854 -2.40021
H  -4.97795 -1.18867 -2.61869
H  -4.96962 -2.68352  1.44267
H  -2.57583 -2.05416  1.66547
H  -0.61023 -2.85378  0.60465
H  -6.19207 -2.24774 -0.70085
H   4.90298 -1.87648  2.36822
```

$TS(IV\text{-}II)_{H,Z}$

```
SCF (TPSS/def2-SVP) Energy = -2137.75722543
Enthalpy 0K = -2137.087418
Enthalpy 298K = -2137.086474
Free Energy 298K = -2137.207002
Lowest Frequency = -203.7923 cm-1
Second Frequency = 10.2259 cm-1
SCF (M052X-D3,MeCN/def2-TZVP) Energy = -
2139.15129310

P   3.19373  0.56337 -1.06008
C   0.58221 -1.16293  0.56667
C   3.13867 -1.24214 -1.37786
C   1.48757  1.11987 -0.42242
C   1.22677  2.57388 -0.55928
C   0.73734  0.16409  0.08858
C   4.29892  0.76336  0.39076
C   0.99052 -1.48016  1.89682
C   0.03482 -2.18520 -0.26076
C   2.65761 -1.64415 -2.64628
C   3.89480  1.33491  1.61540
C   5.66071  0.42441  0.20610
C   0.88948 -2.78982  2.36271
C   0.37218 -3.79678  1.52497
C   3.63779 -2.21805 -0.48734
C  -0.05159 -3.49351  0.22033
C   0.65390  3.28911  0.51701
C   0.73729  5.34043 -0.78285
C   1.57422  3.26689 -1.73879
C   0.41229  4.66360  0.40335
C   6.58644  0.63066  1.23593
C   2.65824 -2.99746 -3.00725
C   3.15830 -3.96050 -2.11608
C   6.17363  1.19177  2.45486
C   3.64819 -3.56910 -0.86005
C   4.82941  1.54528  2.63989
C   1.31843  4.63943 -1.85068
H   1.21663 -3.03513  3.37802
H   1.39849 -0.69022  2.53404
H  -0.28974 -1.93866 -1.27356
H  -0.44662 -4.28492 -0.42405
H   4.50379  1.98921  3.58658
H   2.85012  1.62118  1.77184
H   5.99866 -0.00077 -0.74611
H   7.63602  0.35940  1.08208
H   6.90061  1.35825  3.25636
H   2.29947 -0.89004 -3.35717
H   2.28998 -3.29872 -3.99356
H   3.17835 -5.01621 -2.40668
H   4.04631 -4.31932 -0.16874
H   4.02804 -1.91822  0.49033
H   2.03683  2.72355 -2.56923
H   1.57941  5.16451 -2.77509
H  -0.01940  5.21091  1.24786
H   0.42561  2.76519  1.45106
H   0.54999  6.41559 -0.86956
H   0.29985 -4.82515  1.89467
H  -0.79915  0.68634  0.07634
Si -3.84595 -1.33963  0.40414
C  -3.60456 -1.80878  2.21249
H  -3.59971 -0.93756  2.89073
H  -4.41573 -2.48419  2.54241
H  -2.64801 -2.34631  2.34322
C  -3.89868 -2.86017 -0.69625
H  -3.00087 -3.48865 -0.56218
```

```
H  -4.78326 -3.47473 -0.44828
H  -3.96882 -2.58540 -1.76365
C  -1.99038  0.80664 -0.04960
O  -2.45375 -0.49193 -0.18319
C  -2.16728  1.59373 -1.34775
C  -2.45723  1.52721  1.21339
H  -1.77639  1.01892 -2.20290
H  -2.16746  0.97225  2.12088
H  -1.65522  2.56778 -1.29931
H  -3.24685  1.76688 -1.51060
H  -3.55926  1.61523  1.19335
H  -2.03363  2.54233  1.26385
C  -5.37197 -0.26125  0.14819
C  -6.14914  0.20479  1.23529
C  -5.79661  0.07919 -1.16006
C  -7.30059  0.97865  1.02654
C  -6.94449  0.85507 -1.37241
C  -7.69839  1.30653 -0.27779
H  -5.86068 -0.04391  2.26354
H  -5.22814 -0.27191 -2.03034
H  -7.89027  1.32284  1.88303
H  -7.25652  1.10308 -2.39273
H  -8.59797  1.90925 -0.44236
```

III$_{H,E}$

SCF (TPSS/def2-SVP) Energy = -1944.71471080
Enthalpy 0K = -1944.132641
Enthalpy 298K = -1944.131697
Free Energy 298K = -1944.236969
Lowest Frequency = 10.7168 cm-1
Second Frequency = 20.6959 cm-1
SCF (M052X-D3,MeCN/def2-TZVP) Energy = -1945.95957388

```
P   1.69453  0.05776 -0.80064
C  -1.63810  1.11347  1.61450
C   2.93291 -1.16958 -0.22871
C   0.24789 -0.07032  0.26168
C  -0.04843 -1.43267  0.74030
C  -0.61138  1.07379  0.49833
C   2.43626  1.72355 -0.57465
C  -1.56418  2.17904  2.53700
C  -2.68496  0.17587  1.74595
C   3.36979 -1.25243  1.11123
C   2.59888  2.31981  0.69712
C   2.94899  2.37554 -1.71599
C  -2.49751  2.29361  3.57716
C  -3.52826  1.35130  3.70452
C   3.51014 -2.00697 -1.20559
C  -3.61836  0.29526  2.78460
C  -0.02189 -2.54639 -0.13578
C  -0.40941 -4.05018  1.73102
C  -0.25053 -1.65648  2.12717
C  -0.22330 -3.83943  0.35574
C   3.60721  3.60746 -1.58928
C   4.36580 -2.17105  1.46324
C   4.93748 -3.00402  0.48736
C   3.75540  4.19854 -0.32664
C   4.51121 -2.92077 -0.84533
C   3.25115  3.55393  0.81451
C  -0.40737 -2.95784  2.61326
H  -2.41871  3.12414  4.28633
H  -0.76636  2.92514  2.44183
H  -2.77270 -0.65185  1.03685
H  -4.42288 -0.44234  2.87348
H   3.37574  4.00902  1.80271
H   2.22338  1.82161  1.59740
H   2.83806  1.91787 -2.70487
H   4.00436  4.10445 -2.48024
H   4.26851  5.16073 -0.22882
H   2.93435 -0.60665  1.88027
H   4.69943 -2.23562  2.50415
H   5.71859 -3.71778  0.76881
H   4.95731 -3.56675 -1.60842
H   3.17785 -1.94327 -2.24754
H  -0.24429 -0.81374  2.82321
H  -0.53514 -3.11915  3.68849
H  -0.23140 -4.68678 -0.33704
H   0.11859 -2.38548 -1.20878
Si -1.72833  1.47447 -1.23381
C  -0.53272  2.05980 -2.56302
H   0.20239  1.29925 -2.87882
H  -1.14212  2.31494 -3.45120
H   0.01279  2.96683 -2.25077
C  -2.82357  2.90297 -0.68647
H  -3.37017  3.27055 -1.57548
H  -3.55965  2.61567  0.08178
H  -2.21938  3.73792 -0.28993
H  -0.03924  2.01895  0.45813
C  -2.69607 -0.02653 -1.80860
C  -2.11823 -0.95184 -2.71284
C  -4.04964 -0.21812 -1.43772
C  -2.85869 -2.02961 -3.21719
C  -4.79169 -1.29505 -1.94220
C  -4.19628 -2.20386 -2.82996
H  -1.08078 -0.82201 -3.04384
H  -4.53678  0.48562 -0.75348
H  -2.39624 -2.72905 -3.92190
H  -5.83915 -1.42097 -1.64851
H  -4.77753 -3.04236 -3.22801
H  -0.55476 -5.06485  2.11562
H  -4.26067  1.44202  4.51312
```

TS(III-IV)$_{H,E}$

SCF (TPSS/def2-SVP) Energy = -2137.76679037
Enthalpy 0K = -2137.094684
Enthalpy 298K = -2137.093740
Free Energy 298K = -2137.216429
Lowest Frequency = -131.0172 cm-1
Second Frequency = 13.4078 cm-1
SCF (M052X-D3,MeCN/def2-TZVP) Energy = -2139.17389196

```
P   1.50321  0.70788 -1.06311
C  -0.39839  0.19076  2.71868
C   3.06680 -0.18915 -1.45336
C   0.84996 -0.07658  0.46986
C   1.21657 -1.50289  0.65760
C   0.03435  0.64029  1.36197
C   2.06036  2.39335 -0.54373
C  -0.35470  1.14106  3.76541
C  -0.86220 -1.11126  3.01740
C   4.20264 -0.18039 -0.61673
C   2.58705  2.70242  0.73036
C   2.01220  3.40491 -1.52693
C  -0.73751  0.79908  5.06935
C  -1.18915 -0.49815  5.35166
C   3.12622 -0.86026 -2.69207
C  -1.25294 -1.44795  4.31930
C   0.97132 -2.47782 -0.33428
C   2.10159 -4.15997  1.00965
C   1.92585 -1.87670  1.82452
C   1.39669 -3.79952 -0.14795
C   2.48331  4.69555 -1.24573
C   5.37002 -0.84612 -1.01030
C   5.41955 -1.51889 -2.24219
C   3.00011  4.99351  0.02312
C   4.29833 -1.52434 -3.08319
C   3.05014  3.99575  1.00937
C   2.37320 -3.19193  1.98941
H  -0.68393  1.54940  5.86507
H  -0.00480  2.15875  3.55425
H  -0.91616 -1.86561  2.22789
H  -1.60855 -2.46208  4.53059
H   3.46014  4.22278  1.99934
H   2.63789  1.93475  1.51000
H   1.61007  3.17830 -2.52119
H   2.44342  5.46915 -2.01968
H   3.36549  6.00167  0.24455
H   4.18197  0.35062  0.34019
H   6.24710 -0.83707 -0.35458
H   6.33660 -2.03367 -2.54746
```

```
H   4.33599 -2.04058 -4.04821
H   2.25232 -0.85812 -3.35316
H   2.13923 -1.12829  2.59399
H   2.93479 -3.46253  2.88960
H   1.18338 -4.54880 -0.91727
H   0.42607 -2.20264 -1.24204
Si -2.10742  0.84148  0.09016
C  -1.57398  2.36441 -0.85584
H  -0.72588  2.16538 -1.53268
H  -2.43366  2.71140 -1.45343
H  -1.28806  3.17177 -0.16020
C  -3.13676  1.12756  1.62783
H  -4.08245  1.59287  1.30110
H  -3.35814  0.19692  2.17426
H  -2.62723  1.81645  2.32082
H   0.10114  1.73604  1.27190
C  -2.20041 -0.78914 -0.80834
C  -1.84162 -0.86243 -2.17685
C  -2.69252 -1.95745 -0.17883
C  -1.97680 -2.06239 -2.88992
C  -2.82170 -3.15810 -0.89148
C  -2.46585 -3.21177 -2.24841
H  -1.46063  0.02449 -2.69420
H  -2.98992 -1.93267  0.87532
H  -1.70254 -2.10060 -3.94972
H  -3.20425 -4.05251 -0.38854
H  -2.57258 -4.14808 -2.80649
H   2.44405 -5.19090  1.14662
H  -1.49286 -0.76772  6.36837
C  -5.36376  1.04086 -1.67968
O  -4.34961  1.39511 -1.07074
C  -6.40078  2.06601 -2.08045
C  -5.62400 -0.39866 -2.05803
H  -6.01906  3.08495 -1.92124
H  -6.68099 -0.66474 -1.88460
H  -7.30783  1.91677 -1.46421
H  -6.70727  1.92611 -3.13216
H  -4.95643 -1.08263 -1.51581
H  -5.44304 -0.51105 -3.14412
```

$IV_{H,E}$

SCF (TPSS/def2-SVP) Energy = -1344.16675215
Enthalpy 0K = -1343.761066
Enthalpy 298K = -1343.760122
Free Energy 298K = -1343.840524
Lowest Frequency = 16.3358 cm-1
Second Frequency = 25.8890 cm-1
SCF (M052X-D3,MeCN/def2-TZVP) Energy = -1345.04843856

```
P   1.30259 -0.08148 -1.10502
C  -2.47340  1.21711  0.36727
C   2.21489 -1.08810  0.15605
C  -0.44348 -0.12847 -0.47393
C  -1.07624 -1.46746 -0.65118
C  -1.05591  0.98068  0.03777
C   1.86024  1.64743 -0.74212
C  -2.77883  2.23635  1.30283
C  -3.55507  0.52992 -0.23673
C   1.76014 -1.29015  1.47570
C   2.56942  2.02710  0.41619
C   1.59458  2.62613 -1.72626
C  -4.10200  2.53209  1.65136
C  -5.15969  1.82923  1.05526
C   3.42634 -1.69020 -0.24553
C  -4.87734  0.83503  0.10394
C  -1.00389 -2.15770 -1.88278
C  -2.21656 -4.04508 -0.94235
C  -1.71660 -2.10572  0.43690
C  -1.57566 -3.42985 -2.02724
C   2.00483  3.95351 -1.54550
C   2.50348 -2.07113  2.37308
C   3.71259 -2.65535  1.96604
C   2.70824  4.32044 -0.38721
C   4.17456 -2.46103  0.65525
C   2.99231  3.35414  0.58882
C  -2.28130 -3.37788  0.29161
H  -4.30853  3.31918  2.38515
H  -1.95687  2.79697  1.76357
H  -3.35575 -0.23485 -0.99216
H  -5.69740  0.29562 -0.38316
H   3.54623  3.63306  1.49232
H   2.79549  1.28066  1.18489
H   1.06262  2.34013 -2.64162
H   1.78519  4.70116 -2.31580
H   3.03943  5.35568 -0.24968
H   0.81961 -0.83075  1.79953
H   2.13701 -2.22340  3.39458
H   4.29138 -3.26597  2.66786
H   5.11508 -2.91965  0.33015
H   3.78033 -1.55415 -1.27433
H  -1.76829 -1.59086  1.40201
H  -2.77054 -3.85458  1.14834
H  -1.51758 -3.94257 -2.99383
H  -0.50176 -1.68273 -2.73239
H  -0.41351  1.85273  0.22114
H  -2.65794 -5.04144 -1.05443
H  -6.19699  2.06173  1.31991
```

$TS(IV\text{-}I)_{H,E}$

SCF (TPSS/def2-SVP) Energy = -2137.74750575
Enthalpy 0K = -2137.076639
Enthalpy 298K = -2137.075695
Free Energy 298K = -2137.197982
Lowest Frequency = -47.5642 cm-1
Second Frequency = 8.1345 cm-1
SCF (M052X-D3,MeCN/def2-TZVP) Energy = -2139.14576668

```
P  -1.79201 -1.31711 -0.16109
C   0.23889  2.40041 -0.00202
C  -3.22237 -1.58487 -1.28236
C  -1.72326  0.59608 -0.11036
C  -2.99996  1.41326 -0.15758
C  -0.63537  1.31187 -0.07187
C  -2.44253 -1.65774  1.53093
C   0.58923  2.96209  1.26558
C   0.74661  2.99384 -1.19968
C  -2.90564 -2.08058 -2.56809
C  -1.86333 -1.02239  2.65374
C  -3.36866 -2.70374  1.74574
C   1.36915  4.11375  1.31989
C   1.82658  4.71061  0.12870
C  -4.57496 -1.33291 -0.95458
C   1.52119  4.14787 -1.12505
C  -3.47030  1.89235 -1.39645
C  -5.39020  2.87854 -0.27675
C  -3.71657  1.68308  1.02545
C  -4.66756  2.61889 -1.45077
C  -3.72092 -3.08211  3.04897
C  -3.91834 -2.32207 -3.50632
C  -5.25709 -2.07249 -3.16889
C  -3.15754 -2.42921  4.15493
C  -5.58301 -1.57890 -1.89473
C  -2.22513 -1.39938  3.95325
C  -4.91365  2.40952  0.95744
H   1.62034  4.55956  2.28719
H   0.21280  2.49521  2.18015
H   0.49718  2.54525 -2.16510
H   1.89335  4.61691 -2.04083
H  -1.77507 -0.89031  4.81219
H  -1.12396 -0.22567  2.51174
H  -3.81057 -3.23210  0.89492
H  -4.44037 -3.89407  3.19813
H  -3.43880 -2.72503  5.17076
H  -1.86058 -2.28724 -2.82701
H  -3.66333 -2.71294 -4.49676
H  -6.05019 -2.26629 -3.89882
H  -6.62836 -1.38414 -1.63321
H  -4.83832 -0.94679  0.03497
H  -3.34545  1.31940  1.98813
H  -5.47191  2.61455  1.87683
```

```
H  -5.03330  2.98498 -2.41563
H  -2.90820  1.68407 -2.31223
H  -6.32142  3.45247 -0.32181
H   2.43114  5.62240  0.17907
H   0.73925 -0.02478 -0.18395
Si  4.30997  0.64541  0.37388
C   4.33922  0.78512  2.25541
H   3.98049 -0.12588  2.76576
H   5.36870  0.98212  2.60818
H   3.70721  1.62896  2.58757
C   4.93342  2.23490 -0.41258
H   4.33766  3.10279 -0.07817
H   5.98662  2.41415 -0.12947
H   4.88267  2.19285 -1.51513
C   1.77287 -0.52479 -0.26452
O   2.68785  0.54012 -0.19213
C   1.81581 -1.18821 -1.64383
C   1.86769 -1.51187  0.90074
H   1.72547 -0.42949 -2.43929
H   1.77011 -0.99492  1.87007
H   0.99598 -1.91855 -1.74684
H   2.78062 -1.71019 -1.77253
H   2.84284 -2.03147  0.87463
H   1.06941 -2.26867  0.82536
C   5.31149 -0.84204 -0.21895
C   5.79887 -1.82051  0.68001
C   5.62986 -0.99376 -1.59122
C   6.56742 -2.90458  0.23026
C   6.39617 -2.07594 -2.04549
C   6.86559 -3.03469 -1.13400
H   5.58559 -1.73565  1.75228
H   5.27967 -0.25248 -2.32029
H   6.93794 -3.64627  0.94613
H   6.63335 -2.16997 -3.11084
H   7.46727 -3.87922 -1.48677
```

TS(II-III)$_O$

```
SCF (TPSS/def2-SVP) Energy = -1536.34961506
Enthalpy 0K = -1535.865454
Enthalpy 298K = -1535.864509
Free Energy 298K = -1535.959004
Lowest Frequency = -113.3195 cm-1
Second Frequency = 10.9215 cm-1
SCF (M052X-D3,MeCN/def2-TZVP) Energy = -
1537.41944806
```

```
P  -0.39381  1.19622  1.18999
C  -0.56475 -1.61577 -0.96200
C  -1.87053  0.12047  1.40122
C   0.93616  0.20093  0.26052
C   2.31214  0.74672  0.37207
C   0.49112 -0.86454 -0.37108
C  -0.89798  2.47028 -0.03407
C  -1.14312 -1.15646 -2.17738
C  -1.04157 -2.81787 -0.37253
C  -1.88427 -0.70993  2.54534
C  -0.29663  2.63495 -1.29904
C  -1.87772  3.39980  0.38773
C  -2.18067 -1.87730 -2.77288
C  -2.64549 -3.06172 -2.17749
C  -3.00508  0.15911  0.56320
C  -2.07659 -3.52941 -0.97873
C   3.17234  0.73874 -0.75187
C   4.91409  1.82027  0.55622
C   2.76840  1.31538  1.58210
C   4.46200  1.27340 -0.65609
C  -2.26285  4.45151 -0.45252
C  -3.00080 -1.50731  2.82913
C  -4.12435 -1.46453  1.98840
C  -1.66466  4.60350 -1.71352
C  -4.12595 -0.62850  0.86095
C  -0.68019  3.69711 -2.13153
C   4.06501  1.83915  1.67258
H  -2.62631 -1.51997 -3.70650
H  -0.77019 -0.23572 -2.63460
H  -0.60203 -3.17228  0.56432
H  -2.44844 -4.45032 -0.51903
H  -0.20508  3.81536 -3.11114
H   0.47823  1.93969 -1.63742
H  -2.34170  3.30119  1.37611
H  -3.02703  5.16010 -0.11664
H  -1.96195  5.43102 -2.36577
H  -1.02381 -0.71461  3.22554
H  -3.00442 -2.14349  3.72056
H  -5.00539 -2.07185  2.22119
H  -5.00665 -0.58555  0.21150
H  -3.01546  0.81361 -0.31420
H   2.10218  1.34383  2.45070
H   4.40886  2.27081  2.61807
H   5.11374  1.27531 -1.53604
H   2.81748  0.32617 -1.70119
H   5.92229  2.24147  0.62580
C   2.79173 -2.69081 -0.19136
O   2.05089 -2.03146 -0.94158
C   3.80482 -3.60898 -0.82132
C   2.74337 -2.59027  1.30762
H   3.82050 -3.48368 -1.91301
H   1.75259 -2.26610  1.65887
H   3.54636 -4.65459 -0.56852
H   4.80471 -3.41599 -0.39260
H   3.47786 -1.81877  1.61168
H   3.04141 -3.53800  1.78352
H  -3.45563 -3.62658 -2.65053
```

III$_O$

```
SCF (TPSS/def2-SVP) Energy = -1536.36084679
Enthalpy 0K = -1535.875164
Enthalpy 298K = -1535.874220
Free Energy 298K = -1535.965691
Lowest Frequency = 14.6513 cm-1
Second Frequency = 26.4599 cm-1
SCF (M052X-D3,MeCN/def2-TZVP) Energy = -
1537.44406187
```

```
P  -0.04952  1.05364  1.04860
C  -1.16010 -1.74059 -0.82664
C  -1.86542  0.83621  1.11054
C   0.78939 -0.33905  0.19280
C   2.27250 -0.17548  0.19789
C   0.18080 -1.47392 -0.29771
C   0.27208  2.52940  0.00750
C  -1.84830 -0.75707 -1.57560
C  -1.76581 -3.00877 -0.64889
C  -2.38972 -0.16285  1.96192
C   0.59532  2.46746 -1.36480
C   0.20556  3.78865  0.64489
C  -3.10968 -1.03116 -2.11239
C  -3.70351 -2.28855 -1.92033
C  -2.75186  1.72566  0.46585
C  -3.02692 -3.27744 -1.18921
C   3.02228 -0.51118 -0.95795
C   5.07929  0.21450  0.12027
C   2.96236  0.36927  1.30935
C   4.40784 -0.31815 -0.99318
C   0.43437  4.96277 -0.08448
C  -3.77274 -0.29686  2.12795
C  -4.64874  0.58590  1.47514
C   0.75404  4.89253 -1.44926
C  -4.13542  1.59980  0.65327
C   0.83837  3.64505 -2.08589
C   4.35102  0.56041  1.26713
H  -3.62760 -0.26294 -2.69505
H  -1.37652  0.21340 -1.75240
H  -1.25175 -3.78869 -0.07752
H  -3.48418 -4.26077 -1.04014
H   1.09091  3.58732 -3.14992
H   0.66320  1.49872 -1.87129
H  -0.02523  3.84886  1.71471
H   0.37457  5.93404  0.41741
H   0.94198  5.81033 -2.01596
H  -1.71221 -0.83230  2.50529
H  -4.16908 -1.07943  2.78337
```

```
H  -5.72996  0.49061  1.61922
H  -4.81473  2.29820  0.15321
H  -2.35954  2.51859 -0.17911
H   2.40070  0.64015  2.20970
H   4.86343  0.98536  2.13641
H   4.96513 -0.56555 -1.90291
H   2.50495 -0.89496 -1.84417
H   6.16252  0.36933  0.08718
C   1.73440 -3.25890  0.36239
O   1.00434 -2.65574 -0.51418
C   2.55813 -4.37203 -0.17767
C   1.76134 -2.88365  1.79459
H   2.34655 -4.54439 -1.24230
H   0.81884 -2.40004  2.09984
H   2.38536 -5.29228  0.40945
H   3.62866 -4.11648 -0.04902
H   2.57021 -2.14006  1.95732
H   1.98518 -3.76654  2.41423
H  -4.68870 -2.50078 -2.34799
```

TS(III-IV)$_O$

```
SCF (TPSS/def2-SVP) Energy = -2137.73285905
Enthalpy 0K = -2137.063665
Enthalpy 298K = -2137.062720
Free Energy 298K = -2137.181925
Lowest Frequency = -112.9142 cm-1
Second Frequency = 12.6959 cm-1
SCF (M052X-D3,MeCN/def2-TZVP) Energy = -
2139.13043780

P  -2.73672  0.16786 -1.09589
C   0.04418  1.75834  0.01587
C  -3.31855  1.67593 -0.18758
C  -1.30596 -0.46048 -0.05597
C  -1.32362 -1.90876  0.31463
C  -0.24367  0.33176  0.26102
C  -4.08831 -1.04269 -0.72413
C   0.64191  2.54760  1.02787
C  -0.26211  2.35864 -1.22599
C  -4.06619  2.59816 -0.95021
C  -4.67216 -1.21834  0.54792
C  -4.59362 -1.77445 -1.81878
C   0.89932  3.90609  0.80739
C   0.58053  4.49566 -0.42663
C  -3.11749  1.92792  1.18502
C   0.00486  3.71631 -1.44192
C  -1.26550 -2.31183  1.66752
C  -1.39964 -4.65036  1.00914
C  -1.43490 -2.90409 -0.68334
C  -1.30473 -3.67102  2.00949
C  -5.65343 -2.67736 -1.64371
C  -4.61576  3.73938 -0.34908
C  -4.41326  3.98016  1.01758
C  -6.21868 -2.85378 -0.37343
C  -3.66131  3.07437  1.78143
C  -5.72744 -2.12247  0.72092
C  -1.46480 -4.26211 -0.33743
H   1.33813  4.51204  1.60735
H   0.87668  2.09721  1.99752
H  -0.71153  1.75778 -2.02436
H  -0.24093  4.16794 -2.40847
H  -6.17488 -2.25278  1.71214
H  -4.31029 -0.64024  1.40460
H  -4.15823 -1.62853 -2.81429
H  -6.04076 -3.23693 -2.50181
H  -7.04858 -3.55484 -0.23470
H  -4.22256  2.41749 -2.02022
H  -5.19908  4.44359 -0.95180
H  -4.83887  4.87312  1.48734
H  -3.50080  3.25888  2.84926
H  -2.53135  1.23016  1.79305
H  -1.49894 -2.61004 -1.73682
H  -1.54975 -5.01895 -1.12425
H  -1.27232 -3.96474  3.06420
H  -1.20665 -1.55321  2.45499
H  -1.43413 -5.71104  1.27840
C   1.82862 -0.90576  0.58469
O   0.79783 -0.26554  1.08877
C   2.59453 -1.67761  1.61908
C   1.83104 -1.35371 -0.84583
H   2.59850 -1.14543  2.58203
H   1.41936 -0.58533 -1.51752
H   3.62009 -1.89325  1.28369
H   2.05930 -2.63762  1.75736
H   1.17672 -2.24585 -0.90660
H   2.84824 -1.63885 -1.15426
H   0.77644  5.55972 -0.59440
Si  4.27139  1.11886  0.06797
H   2.95226  0.31275  0.37944
C   4.60296  2.11144  1.63097
H   4.68886  1.47270  2.52746
H   5.55249  2.66791  1.52096
H   3.79611  2.84406  1.80465
C   3.85533  2.16250 -1.43792
H   3.02105  2.85094 -1.21765
H   4.73722  2.76667 -1.72228
H   3.57319  1.54857 -2.31105
C   5.55318 -0.21407 -0.26382
C   6.34608 -0.74500  0.78471
C   5.75765 -0.72062 -1.57207
C   7.30302 -1.73862  0.53591
C   6.71400 -1.71454 -1.82184
C   7.48659 -2.22564 -0.76752
H   6.22896 -0.36999  1.80848
H   5.17649 -0.32449 -2.41347
H   7.91213 -2.12853  1.35824
H   6.86320 -2.08540 -2.84139
H   8.23694 -2.99898 -0.96284
```

IV$_O$

```
SCF (TPSS/def2-SVP) Energy = -2137.73410595
Enthalpy 0K = -2137.063341
Enthalpy 298K = -2137.062397
Free Energy 298K = -2137.181957
Lowest Frequency = 11.6035 cm-1
Second Frequency = 14.4760 cm-1
SCF (M052X-D3,MeCN/def2-TZVP) Energy = -
2139.13691847

P  -2.70328  0.18813 -1.09370
C   0.07419  1.73649 -0.03736
C  -3.27956  1.67581 -0.14552
C  -1.27533 -0.46705 -0.06645
C  -1.31565 -1.91050  0.31932
C  -0.19388  0.30683  0.23862
C  -4.06559 -1.01954 -0.74715
C   0.62499  2.55491  0.97781
C  -0.20375  2.31202 -1.29722
C  -4.03902  2.61327 -0.87725
C  -4.64749 -1.22122  0.52182
C  -4.57932 -1.72222 -1.85666
C   0.86544  3.91513  0.74366
C   0.57699  4.47841 -0.51011
C  -3.06016  1.89964  1.22910
C   0.04653  3.67107 -1.52869
C  -1.25142 -2.30101  1.67531
C  -1.43308 -4.64365  1.04360
C  -1.45222 -2.91442 -0.66617
C  -1.31162 -3.65538  2.03227
C  -5.64473 -2.62199 -1.69988
C  -4.58220  3.74045 -0.24466
C  -4.36192  3.95250  1.12411
C  -6.20800 -2.82413 -0.43257
C  -3.59807  3.03188  1.85767
C  -5.70871 -2.12168  0.67688
C  -1.50271 -4.26816 -0.30627
H   1.26328  4.54383  1.54786
H   0.82864  2.12197  1.96262
H  -0.62337  1.69107 -2.09633
H  -0.17823  4.10257 -2.50962
H  -6.15392 -2.27230  1.66628
H  -4.27812 -0.66689  1.39092
```

```
H  -4.14488 -1.55631 -2.84949
H  -6.03775 -3.15925 -2.56966
H  -7.04209 -3.52290 -0.30775
H  -4.20968  2.45491 -1.94864
H  -5.17483  4.45630 -0.82429
H  -4.78303  4.83418  1.61871
H  -3.42397  3.19322  2.92724
H  -2.46479  1.18933  1.81328
H  -1.51842 -2.63078 -1.72233
H  -1.60685 -5.03150 -1.08458
H  -1.27260 -3.93868  3.08970
H  -1.16620 -1.53520  2.45296
H  -1.48353 -5.70078  1.32444
C   1.94624 -0.83692  0.52176
O   0.82969 -0.27580  1.05810
C   2.62319 -1.70905  1.56170
C   1.86188 -1.38464 -0.88977
H   2.65996 -1.19206  2.53345
H   1.44153 -0.64511 -1.58919
H   3.63800 -1.99411  1.24422
H   2.01392 -2.62256  1.67950
H   1.19732 -2.26653 -0.87851
H   2.86212 -1.69602 -1.23068
H   0.75760  5.54331 -0.68969
Si  4.20578  1.08540  0.08843
H   2.81496  0.14686  0.36766
C   4.35736  1.97882  1.72642
H   4.41266  1.28654  2.58401
H   5.28864  2.57741  1.71507
H   3.50888  2.66592  1.87826
C   3.68550  2.10909 -1.38540
H   2.80677  2.73273 -1.14929
H   4.52573  2.77858 -1.65418
H   3.45366  1.48978 -2.26845
C   5.50885 -0.20066 -0.24327
C   6.30614 -0.71844  0.81019
C   5.75127 -0.66610 -1.56158
C   7.30819 -1.66232  0.55358
C   6.75306 -1.61106 -1.81560
C   7.53043 -2.11038 -0.75843
H   6.15323 -0.37501  1.83979
H   5.16259 -0.27971 -2.40163
H   7.92056 -2.04623  1.37594
H   6.93279 -1.95503 -2.83934
H   8.31521 -2.84746 -0.95824
```

$TS(IV\text{-}V)_O$

```
SCF (TPSS/def2-SVP) Energy = -2137.71729866
Enthalpy 0K = -2137.045525
Enthalpy 298K = -2137.044581
Free Energy 298K = -2137.164355
Lowest Frequency = -131.0285 cm-1
Second Frequency = 10.1963 cm-1
SCF (M052X-D3,MeCN/def2-TZVP) Energy = -
2139.13479049

P  -2.72954  0.49323 -0.83573
C  -0.00252  1.87895 -0.79728
C  -3.22138  1.47138  0.66287
C  -1.12358 -0.33142 -0.28047
C  -1.09230 -1.73427  0.22120
C  -0.00526  0.43293 -0.40364
C  -3.95978 -0.89204 -0.80963
C   0.05053  2.87985  0.19759
C  -0.02997  2.27270 -2.15309
C  -4.15270  2.50881  0.44047
C  -4.53320 -1.43197  0.36048
C  -4.37643 -1.38473 -2.06518
C   0.10712  4.23661 -0.15429
C   0.10064  4.61376 -1.50548
C  -2.74146  1.26086  1.97189
C   0.02359  3.62939 -2.50297
C  -0.72963 -2.00200  1.56072
C  -1.04636 -4.38771  1.21255
C  -1.43838 -2.82341 -0.60971
C  -0.71225 -3.31437  2.05243
C  -5.32685 -2.41311 -2.14981
C  -4.60544  3.30318  1.50296
C  -4.12246  3.08398  2.80194
C  -5.88205 -2.94997 -0.97919
C  -3.18819  2.06318  3.03304
C  -5.48668 -2.45512  0.27409
C  -1.40691 -4.13699 -0.12010
H   0.13766  5.00088  0.62958
H   0.01472  2.59212  1.25376
H  -0.11936  1.51281 -2.93594
H  -0.00675  3.91887 -3.55870
H  -5.92741 -2.86572  1.18903
H  -4.23951 -1.04630  1.34228
H  -3.95872 -0.94942 -2.98113
H  -5.64377 -2.78479 -3.13030
H  -6.63169 -3.74607 -1.04245
H  -4.52332  2.69488 -0.57441
H  -5.33191  4.10081  1.31382
H  -4.47265  3.70715  3.63167
H  -2.81174  1.88395  4.04646
H  -2.01974  0.46155  2.16719
H  -1.73866 -2.64064 -1.64617
H  -1.67699 -4.96686 -0.78166
H  -0.43786 -3.49951  3.09674
H  -0.45621 -1.17074  2.21938
H  -1.03410 -5.41306  1.59666
C   2.04147 -0.96958 -0.85650
O   1.28260 -0.03441 -0.03969
C   2.23967 -2.30922 -0.15789
C   1.59386 -1.07366 -2.30741
H   2.52126 -2.16603  0.89793
H   1.55221 -0.08648 -2.79276
H   3.04130 -2.87536 -0.66354
H   1.31246 -2.90287 -0.19145
H   0.59519 -1.53828 -2.35711
H   2.29883 -1.71542 -2.86162
H   0.13706  5.67299 -1.78083
Si  3.83645  0.92221  0.35691
H   3.07836 -0.47521 -0.88270
C   3.07772  1.12980  2.04022
H   2.79838  0.16347  2.49213
H   3.83030  1.61171  2.69508
H   2.18275  1.76736  1.98417
C   3.62195  2.25596 -0.91484
H   2.55624  2.49763 -1.06468
H   4.12073  3.16047 -0.51161
H   4.08921  2.01040 -1.88203
C   5.37259 -0.09838  0.30112
C   5.76185 -0.86906  1.42962
C   6.20638 -0.10743 -0.84954
C   6.94852 -1.60960  1.41052
C   7.39215 -0.84819 -0.86290
C   7.76230 -1.59930  0.26575
H   5.13959 -0.88448  2.33132
H   5.93208  0.47291 -1.73778
H   7.24213 -2.19467  2.28781
H   8.03164 -0.84117 -1.75127
H   8.69082 -2.17964  0.25255
```

V_O

```
SCF (TPSS/def2-SVP) Energy = -2137.76597687
Enthalpy 0K = -2137.091074
Enthalpy 298K = -2137.090130
Free Energy 298K = -2137.206048
Lowest Frequency = 10.5237 cm-1
Second Frequency = 18.4025 cm-1
SCF (M052X-D3,MeCN/def2-TZVP) Energy = -
2139.18811397

P  -2.49713  0.63087 -0.42671
C   0.13615  2.29453 -0.83303
C  -3.01179  0.83483  1.34255
C  -0.72257 -0.05706 -0.30894
C  -0.44938 -1.48214  0.03592
C   0.26307  0.83707 -0.61555
C  -3.50372 -0.82219 -0.96669
```

```
C  -0.64834  3.06537  0.05909
C   0.74061  2.95354 -1.93046
C  -4.04716  1.77316  1.55578
C  -4.12059 -1.74576 -0.09698
C  -3.72803 -0.93563 -2.35818
C  -0.84098  4.43618 -0.15353
C  -0.25205  5.06836 -1.25750
C  -2.46659  0.16078  2.45571
C   0.54240  4.32258 -2.14290
C   0.32126 -1.78802  1.18306
C   0.07861 -4.17147  0.77597
C  -0.95350 -2.55899 -0.73011
C   0.57810 -3.11447  1.55169
C  -4.52113 -1.97140 -2.87069
C  -4.53482  2.01876  2.84671
C  -3.98253  1.34565  3.94707
C  -5.12082 -2.89187 -1.99643
C  -2.94728  0.42038  3.74873
C  -4.92532 -2.77218 -0.61171
C  -0.68420 -3.88621 -0.36545
H  -1.45059  5.01065  0.55119
H  -1.08432  2.59003  0.94293
H   1.36189  2.39531 -2.63639
H   1.00559  4.80851 -3.00776
H  -5.40438 -3.48073  0.07248
H  -3.97782 -1.65839  0.98481
H  -3.28653 -0.19753 -3.03912
H  -4.68822 -2.04891 -3.95040
H  -5.75273 -3.69331 -2.39372
H  -4.47208  2.31354  0.70142
H  -5.34310  2.74305  2.99363
H  -4.35794  1.54227  4.95680
H  -2.51589 -0.11128  4.60396
H  -1.67423 -0.58003  2.31750
H  -1.54881 -2.36195 -1.62460
H  -1.08044 -4.70156 -0.97950
H   1.16854 -3.32329  2.45016
H   0.70541 -0.97135  1.80188
H   0.27854 -5.20909  1.06217
C   2.08345 -0.47100 -2.04101
O   1.65103  0.35095 -0.76569
C   2.45143 -1.89272 -1.65496
C   1.01552 -0.39225 -3.11521
H   3.16255 -1.92702 -0.81944
H   0.71943  0.63739 -3.36266
H   2.94335 -2.34571 -2.53486
H   1.56289 -2.49104 -1.40796
H   0.12126 -0.97142 -2.84145
H   1.44858 -0.84631 -4.02389
H  -0.40471  6.13927 -1.42597
Si  3.02204  1.13499  0.25776
H   2.97790  0.09987 -2.34264
C   2.17960  1.76917  1.80190
H   1.52336  1.02738  2.28710
H   2.98224  2.02492  2.51941
H   1.59075  2.67971  1.60737
C   3.80616  2.48661 -0.77831
H   3.14968  3.36641 -0.86891
H   4.73128  2.79797 -0.25690
H   4.09779  2.16411 -1.79321
C   4.26193 -0.21655  0.62494
C   4.11287 -1.05775  1.75486
C   5.42762 -0.36573 -0.16677
C   5.08061 -2.02045  2.07048
C   6.39520 -1.32988  0.14654
C   6.22052 -2.16064  1.26382
H   3.23599 -0.95858  2.40414
H   5.59129  0.27963 -1.03791
H   4.95004 -2.65846  2.95081
H   7.28997 -1.42859 -0.47684
H   6.97803 -2.91156  1.51172
```

TS(V-II)$_O$

```
SCF (TPSS/def2-SVP) Energy = -2137.74727114
Enthalpy 0K = -2137.074647
Enthalpy 298K = -2137.073703
Free Energy 298K = -2137.192789
Lowest Frequency = -102.7112 cm-1
Second Frequency = 12.6625 cm-1
SCF (M052X-D3,MeCN/def2-TZVP) Energy = -
2139.15367225

P  -2.75601  0.94012  0.23273
C   0.13897  2.47136  0.09603
C  -3.34044 -0.09509  1.63535
C  -0.96709  0.09334 -0.09169
C  -0.85540 -1.37788 -0.16366
C  -0.12225  1.08513 -0.10562
C  -3.64022  0.32476 -1.26208
C   0.06299  2.99757  1.41677
C   0.36431  3.35890 -0.99083
C  -3.57713  0.62400  2.83109
C  -4.84623 -0.40674 -1.20500
C  -3.17771  0.78144 -2.51989
C   0.21627  4.36779  1.63623
C   0.45200  5.23090  0.55263
C  -3.56939 -1.49127  1.61808
C   0.51946  4.72538 -0.75845
C  -0.25666 -2.07730  0.90621
C  -0.68698 -4.18944 -0.21989
C  -1.38293 -2.09965 -1.25626
C  -0.17364 -3.47492  0.87347
C  -3.87964  0.47789 -3.69153
C  -4.04250 -0.03216  3.97878
C  -4.26989 -1.41566  3.94753
C  -5.07170 -0.26332 -3.62509
C  -4.03303 -2.14170  2.76827
C  -5.55529 -0.69327 -2.38176
C  -1.28899 -3.49849 -1.28235
H   0.14604  4.76791  2.65248
H  -0.14462  2.32304  2.25205
H   0.40440  2.96407 -2.00812
H   0.68719  5.40516 -1.59950
H  -6.49280 -1.25601 -2.32188
H  -5.24592 -0.73915 -0.24263
H  -2.26113  1.38064 -2.58024
H  -3.50495  0.83069 -4.65819
H  -5.62655 -0.49356 -4.54042
H  -3.40820  1.70713  2.85083
H  -4.23217  0.53746  4.89442
H  -4.63440 -1.93102  4.84244
H  -4.21096 -3.22196  2.74519
H  -3.38828 -2.06995  0.70765
H  -1.85557 -1.56839 -2.08633
H  -1.68982 -4.04898 -2.13967
H   0.29160 -4.00838  1.70905
H   0.13004 -1.52156  1.76435
H  -0.62006 -5.28200 -0.24277
C   2.28349 -0.21112 -1.85940
O   1.90159  0.26477 -0.51031
C   2.25266 -1.73620 -1.91354
C   1.41400  0.44232 -2.92926
H   2.90785 -2.16898 -1.14205
H   1.54481  1.53612 -2.94104
H   2.61908 -2.07295 -2.89933
H   1.23079 -2.12113 -1.77235
H   0.34730  0.20214 -2.78198
H   1.71392  0.06067 -3.92004
H   0.57501  6.30460  0.72872
Si  3.19469  0.64995  0.61736
H   3.32610  0.12712 -2.01583
C   2.50239  0.38706  2.34511
H   2.26734 -0.67478  2.53345
H   3.26794  0.69193  3.08251
H   1.59492  0.97891  2.54541
C   3.73239  2.43091  0.32054
H   2.93666  3.14842  0.58460
H   4.61737  2.66443  0.94077
H   4.00861  2.61017 -0.73433
C   4.65089 -0.51484  0.32915
C   4.70021 -1.79521  0.93258
C   5.75877 -0.11659 -0.45722
C   5.80298 -2.64184  0.75378
```

```
C   6.86311 -0.96135 -0.64092
C   6.88608 -2.22640 -0.03584
H   3.86765 -2.14208  1.55630
H   5.77057  0.87265 -0.93043
H   5.82024 -3.62598  1.23426
H   7.70933 -0.62924 -1.25179
H   7.74911 -2.88621 -0.17488
```

TS(II-III)$_P$

```
SCF (TPSS/def2-SVP) Energy = -1536.35103448
Enthalpy 0K = -1535.866798
Enthalpy 298K = -1535.865854
Free Energy 298K = -1535.963682
Lowest Frequency = -79.1649 cm-1
Second Frequency = 12.1473 cm-1
SCF (M052X-D3,MeCN/def2-TZVP) Energy = -
1537.42048332

P   0.75895  0.54709  0.10236
C  -2.26787 -1.64000 -0.47296
C  -0.13471  0.53119  1.69387
C   0.38802 -1.84170 -0.54568
C   1.68902 -2.46250 -0.61321
C  -0.85410 -1.73695 -0.51909
C  -0.28213  1.45301 -1.10678
C  -2.96355 -2.07117  0.69072
C  -3.00749 -1.15714 -1.58649
C   0.65142  0.16736  2.81395
C  -0.20821  1.04922 -2.46109
C  -1.00389  2.62565 -0.78143
C  -4.35865 -2.02078  0.72950
C  -5.07962 -1.53602 -0.37505
C  -1.50761  0.80992  1.87701
C  -4.40202 -1.10460 -1.52818
C   1.95544 -3.58457  0.20898
C   4.18421 -3.76530 -0.74496
C   2.69372 -1.99761 -1.49418
C   3.19650 -4.22733  0.13834
C  -1.65610  3.35417 -1.78542
C   0.08396  0.11256  4.09174
C  -1.27788  0.40840  4.26580
C  -1.59564  2.93048 -3.12180
C  -2.07043  0.75008  3.15923
C  -0.86760  1.77843 -3.45875
C   3.92838 -2.65159 -1.56135
H  -4.88962 -2.36380  1.62327
H  -2.39526 -2.44826  1.54567
H  -2.47655 -0.83612 -2.48641
H  -4.96736 -0.73489 -2.38957
H  -0.80752  1.45039 -4.50174
H   0.35841  0.15180 -2.73379
H  -1.03266  2.98415  0.25150
H  -2.21038  4.26103 -1.52177
H  -2.10770  3.50305 -3.90214
H   1.71353 -0.07046  2.68006
H   0.70202 -0.16234  4.95263
H  -1.72299  0.36673  5.26544
H  -3.13494  0.96880  3.29330
H  -2.13992  1.06245  1.02138
H   2.49522 -1.13098 -2.13286
H   4.69486 -2.29432 -2.25688
H   3.39136 -5.09693  0.77432
H   1.18124 -3.94672  0.89190
H   5.15228 -4.27367 -0.79951
H  -6.17344 -1.50072 -0.33987
C   2.72609  2.95478  0.47335
O   1.59008  2.51609  0.73214
C   3.68119  2.24122 -0.44367
C   3.14516  4.26308  1.08431
H   3.49438  1.15566 -0.44185
H   2.30222  4.74168  1.60226
H   4.72933  2.46413 -0.18891
H   3.50300  2.61775 -1.47026
H   3.55800  4.93116  0.30729
H   3.96405  4.07176  1.80393
```

III$_P$

```
SCF (TPSS/def2-SVP) Energy = -997.151636782
Enthalpy 0K = -996.869242
Enthalpy 298K = -996.868297
Free Energy 298K = -996.935322
Lowest Frequency = 13.8109 cm-1
Second Frequency = 24.7134 cm-1
SCF (M052X-D3,MeCN/def2-TZVP) Energy = -
997.812018559

P   0.04375  0.32393 -0.98548
C   1.50120 -0.55401 -0.35702
C  -1.41758 -0.54732 -0.34970
C   2.75346 -0.01018 -0.74166
C  -2.38310 -0.89853 -1.32275
C  -1.65190 -0.85574  1.01286
C   1.46584 -1.76662  0.37111
C  -2.82715 -1.51803  1.38300
C   3.94312 -0.63915 -0.36234
C   3.89640 -1.83459  0.37401
C  -3.76759 -1.89050  0.40613
C   2.66121 -2.40004  0.73017
C  -3.54521 -1.58418 -0.94427
H  -4.28142 -1.86809 -1.70283
H  -2.21951 -0.64249 -2.37615
H  -0.92513 -0.56450  1.77796
H  -3.01224 -1.74882  2.43716
H  -4.68161 -2.41507  0.70310
H   2.79195  0.90527 -1.34465
H   4.90654 -0.20952 -0.65445
H   4.82733 -2.33628  0.65784
H   2.62973 -3.34267  1.28581
H   0.50859 -2.22395  0.63718
C  -0.13754  2.87454  0.36288
O   0.04244  1.62877  0.46413
C  -0.37207  3.57148 -0.93631
C  -0.10702  3.65764  1.63512
H  -0.60288  2.87497 -1.75483
H   0.33766  3.07097  2.45139
H   0.55172  4.13004 -1.18848
H  -1.16753  4.32768 -0.82029
H  -1.15357  3.90938  1.90027
H   0.42228  4.61526  1.49277
```

TS(III-IV)$_P$

```
SCF (TPSS/def2-SVP) Energy = -1598.52547055
Enthalpy 0K = -1598.059974
Enthalpy 298K = -1598.059029
Free Energy 298K = -1598.152815
Lowest Frequency = -117.5322 cm-1
Second Frequency = 10.1122 cm-1
SCF (M052X-D3,MeCN/def2-TZVP) Energy = -
1599.49262479

P  -1.76615  0.00138  0.71780
C  -2.71168  1.45817  0.15826
C  -2.81996 -1.44684  0.33259
C  -2.20219  2.71328  0.56756
C  -3.34055 -2.15348  1.43881
C  -3.11779 -1.88642 -0.97786
C  -3.91391  1.40723 -0.58074
C  -3.93054 -3.01133 -1.17001
C  -2.86033  3.89605  0.21010
C  -4.04629  3.83528 -0.53824
C  -4.46109 -3.69734 -0.06486
C  -4.57358  2.59320 -0.92665
C  -4.16888 -3.26681  1.23752
H  -4.57713 -3.80380  2.09977
H  -3.10039 -1.83106  2.45894
H  -2.71360 -1.35106 -1.84376
H  -4.15673 -3.35114 -2.18620
H  -5.09991 -4.57270 -0.22113
H  -1.29108  2.75953  1.17649
H  -2.45837  4.86377  0.52712
H  -4.56919  4.75861 -0.80851
```

```
H  -5.50673  2.54842 -1.49752
H  -4.33963  0.44335 -0.87572
C   0.43222 -0.75742 -0.79261
O  -0.68577 -0.08192 -0.74199
C   0.63809 -1.95419  0.09650
C   1.11561 -0.69742 -2.13221
H   0.29760 -1.75462  1.12621
H   0.95928  0.28453 -2.60300
H   1.69185 -2.27161  0.09550
H   0.01777 -2.77739 -0.30956
H   0.64953 -1.46873 -2.77603
H   2.18932 -0.92588 -2.04672
Si  2.64244  1.06681  0.65677
H   1.48141  0.21853  0.01286
C   2.28123  1.03816  2.50279
H   2.28687  0.01504  2.91766
H   3.05111  1.62310  3.04002
H   1.29637  1.48796  2.71963
C   2.45005  2.76483 -0.12962
H   1.46066  3.19597  0.10464
H   3.22223  3.45088  0.26578
H   2.55617  2.73477 -1.22814
C   4.20398  0.15478  0.16164
C   4.70469 -0.91231  0.94935
C   4.91322  0.50378 -1.01491
C   5.86789 -1.59981  0.57774
C   6.07733 -0.18286 -1.38630
C   6.55474 -1.23609 -0.59112
H   4.19147 -1.20445  1.87344
H   4.56459  1.33070 -1.64483
H   6.24412 -2.41557  1.20400
H   6.61698  0.10804 -2.29367
H   7.46613 -1.77041 -0.87938
```

IV_P

SCF (TPSS/def2-SVP) Energy = -1598.52719236
Enthalpy 0K = -1598.059951
Enthalpy 298K = -1598.059007
Free Energy 298K = -1598.153787
Lowest Frequency = 7.2374 cm-1
Second Frequency = 14.5773 cm-1
SCF (M052X-D3,MeCN/def2-TZVP) Energy = -1599.50042789

```
P  -1.62203 -0.03706  0.64855
C  -2.46389  1.49903  0.10786
C  -2.84998 -1.36294  0.31058
C  -2.18960  2.66295  0.85999
C  -3.62371 -1.80145  1.40637
C  -3.04417 -1.95315 -0.95688
C  -3.36221  1.57359 -0.97869
C  -4.00745 -2.95859 -1.12200
C  -2.79003  3.88469  0.52304
C  -3.66856  3.95122 -0.56784
C  -4.78578 -3.37782 -0.03177
C  -3.95337  2.79661 -1.31668
C  -4.59443 -2.79740  1.23107
H  -5.19526 -3.12716  2.08494
H  -3.46893 -1.36278  2.39958
H  -2.44158 -1.62704 -1.81134
H  -4.15374 -3.41393 -2.10732
H  -5.53835 -4.16167 -0.16592
H  -1.51464  2.60632  1.72278
H  -2.57881  4.78081  1.11574
H  -4.14213  4.90285 -0.83110
H  -4.64799  2.84987 -2.16148
H  -3.60640  0.67468 -1.55364
C   0.56610 -0.93012 -0.74123
O  -0.59763 -0.22749 -0.77470
C   0.57367 -2.23538  0.04090
C   1.20274 -0.96012 -2.12039
H   0.18698 -2.09219  1.06412
H   1.16509  0.03896 -2.58188
H   1.59141 -2.65537  0.08744
H  -0.08477 -2.95591 -0.47628
H   0.62496 -1.65530 -2.75487
H   2.24435 -1.31523 -2.07038
Si  2.48843  0.82478  0.73345
H   1.40194 -0.22303 -0.05981
C   2.01252  0.55988  2.52318
H   2.16728 -0.48191  2.85214
H   2.64550  1.21141  3.15663
H   0.95548  0.82567  2.69228
C   1.93798  2.43497 -0.04130
H   0.85153  2.58179  0.08620
H   2.45890  3.27242  0.46161
H   2.17245  2.48741 -1.11800
C   4.12917  0.14185  0.20256
C   4.77749 -0.85944  0.97178
C   4.77050  0.61278 -0.97266
C   6.02378 -1.36433  0.58283
C   6.01727  0.10629 -1.35811
C   6.64317 -0.88236 -0.58167
H   4.31272 -1.24192  1.88774
H   4.30026  1.38893 -1.58704
H   6.51607 -2.13129  1.18939
H   6.50483  0.48470 -2.26232
H   7.61902 -1.27675 -0.88377
```

TS(IV-V)_P

SCF (TPSS/def2-SVP) Energy = -1598.51767159
Enthalpy 0K = -1598.049559
Enthalpy 298K = -1598.048615
Free Energy 298K = -1598.140812
Lowest Frequency = -106.2159 cm-1
Second Frequency = 8.5411 cm-1
SCF (M052X-D3,MeCN/def2-TZVP) Energy = -1599.50523167

```
P   1.88039 -0.01331 -1.23201
C   2.25911  1.53032 -0.29552
C   2.81350 -1.28944 -0.28703
C   2.15478  2.74702 -1.00628
C   4.12090 -1.56408 -0.74425
C   2.30561 -2.00984  0.81274
C   2.65889  1.55770  1.05819
C   3.09626 -2.97620  1.45207
C   2.41746  3.96858 -0.36904
C   2.79805  3.98535  0.98164
C   4.40132 -3.22923  1.00330
C   2.92129  2.78024  1.69238
C   4.91287 -2.52048 -0.09458
H   5.92746 -2.72112 -0.45423
H   4.52162 -1.02818 -1.61353
H   1.28794 -1.81500  1.16409
H   2.69318 -3.53335  2.30482
H   5.01671 -3.98392  1.50388
H   1.87937  2.73331 -2.06785
H   2.34104  4.90571 -0.93058
H   3.01535  4.93743  1.47729
H   3.23756  2.79294  2.74099
H   2.78418  0.61930  1.60959
C  -0.72687 -0.85496 -1.46959
O   0.26102 -0.24438 -0.61592
C  -0.88472 -0.18248 -2.83156
C  -0.59051 -2.37255 -1.53149
H  -0.97019  0.91182 -2.73100
H  -0.54784 -2.81312 -0.52279
H  -1.78150 -0.56907 -3.34467
H  -0.00488 -0.40027 -3.46022
H   0.34654 -2.62868 -2.05827
H  -1.43120 -2.82031 -2.08778
Si -2.01682  0.53001  0.66189
H  -1.70458 -0.64736 -0.89396
C  -1.55878  2.22330  0.05681
H  -1.96667  2.44565 -0.94323
H  -2.00771  2.94708  0.76707
H  -0.46655  2.36356  0.04923
C  -1.11650 -0.21695  2.10495
H  -0.08392  0.15965  2.15732
H  -1.66017  0.07525  3.02524
H  -1.09939 -1.31917  2.06412
```

C -3.77910 0.01859 0.45621
C -4.65321 0.74256 -0.39874
C -4.29491 -1.09515 1.17245
C -5.99693 0.37383 -0.51884
C -5.63938 -1.45984 1.04821
C -6.48907 -0.72644 0.20318
H -4.28571 1.60448 -0.96690
H -3.64579 -1.67580 1.83763
H -6.66395 0.94350 -1.17361
H -6.02844 -2.31471 1.61033
H -7.54125 -1.01398 0.10676

V_P

SCF (TPSS/def2-SVP) Energy = -1598.56662858
Enthalpy 0K = -1598.095411
Enthalpy 298K = -1598.094467
Free Energy 298K = -1598.184900
Lowest Frequency = 3.2788 cm-1
Second Frequency = 16.4966 cm-1
SCF (M052X-D3,MeCN/def2-TZVP) Energy = -1599.558483

P   1.43249 0.17184 1.22019
C   2.45327 -0.95834 0.20152
C   1.55395 1.80994 0.41230
C   2.82934 -2.16042 0.85061
C   2.83792 2.30636 0.08328
C   0.44848 2.68761 0.35454
C   2.93907 -0.70637 -1.10314
C   0.61434 4.01168 -0.07165
C   3.63684 -3.10115 0.19934
C   4.09734 -2.84493 -1.10136
C   1.88776 4.48918 -0.41798
C   3.75485 -1.64489 -1.74671
C   2.99718 3.63659 -0.33062
H   3.99630 4.00485 -0.58505
H   3.72103 1.66343 0.16135
H -0.54713 2.33561 0.64045
H -0.25412 4.67657 -0.12413
H   2.01570 5.52603 -0.74475
H   2.49433 -2.35222 1.87618
H   3.91959 -4.02615 0.71189
H   4.73637 -3.57478 -1.60922
H   4.12985 -1.43815 -2.75441
H   2.69832 0.23352 -1.60848
C -1.14817 -0.71542 1.97682
O -0.30205 -0.31175 0.74620
C -0.50157 -1.87919 2.71264
C -1.40322 0.52186 2.81913
H -0.27330 -2.71527 2.03207
H -1.94737 1.29046 2.24750
H -1.21995 -2.24405 3.46778
H   0.41662 -1.57261 3.24017
H -0.46254 0.94594 3.21163
H -2.02941 0.23435 3.68159
Si -1.04706 -0.73935 -0.89979
H -2.08553 -1.04246 1.50157
C -0.66529 -2.55650 -1.14350
H -1.09274 -3.19565 -0.35149
H -1.09081 -2.89036 -2.10817
H   0.42419 -2.72962 -1.17695
C -0.29237 0.36362 -2.20471
H   0.77448 0.16497 -2.38390
H -0.83769 0.12922 -3.13962
H -0.42432 1.43828 -2.00059
C -2.87666 -0.38471 -0.70125
C -3.81908 -1.43761 -0.59659
C -3.36403 0.94607 -0.71566
C -5.19246 -1.17083 -0.50455
C -4.73594 1.21361 -0.61816
C -5.65140 0.15476 -0.51167
H -3.48350 -2.48137 -0.59555
H -2.66823 1.78798 -0.81537
H -5.90547 -1.99858 -0.43154
H -5.09374 2.24842 -0.63361
H -6.72397 0.36354 -0.44047

$TS(V\text{-}II)_P$

SCF (TPSS/def2-SVP) Energy = -2137.75743597
Enthalpy 0K = -2137.084655
Enthalpy 298K = -2137.083711
Free Energy 298K = -2137.206700
Lowest Frequency = -112.9936 cm-1
Second Frequency = 9.3379 cm-1
SCF (M052X-D3,MeCN/def2-TZVP) Energy = -2139.16559561

P -0.55875 -0.42533 0.12311
C -3.97156 1.02145 -0.39165
C -0.84411 0.55290 1.63837
C -2.78017 -1.35924 -0.28885
C -2.64838 -2.79598 -0.36846
C -3.32213 -0.23609 -0.34772
C -0.42719 0.73609 -1.28481
C -4.70042 1.48418 0.73968
C -3.93652 1.81383 -1.57209
C -0.50218 -0.11563 2.84079
C -0.63026 0.15858 -2.56385
C -0.02998 2.09048 -1.20982
C -5.37642 2.70441 0.68106
C -5.33317 3.48166 -0.48955
C -1.43140 1.83757 1.71528
C -4.61297 3.03525 -1.61090
C -2.39467 -3.56212 0.79322
C -2.45388 -5.59818 -0.53367
C -2.79941 -3.44871 -1.61493
C -2.30312 -4.95538 0.70577
C   0.13911 2.84703 -2.37834
C -0.70958 0.49692 4.08153
C -1.27561 1.78103 4.14304
C -0.08503 2.26765 -3.63600
C -1.64165 2.44378 2.96133
C -0.47116 0.91957 -3.72667
C -2.70274 -4.84310 -1.69015
H -5.94622 3.05174 1.54884
H -4.73318 0.86989 1.64391
H -3.38144 1.45535 -2.44343
H -4.58724 3.64122 -2.52212
H -0.63726 0.45928 -4.70614
H -0.92003 -0.89525 -2.64478
H   0.16847 2.55341 -0.24111
H   0.45101 3.89409 -2.30382
H   0.04899 2.86199 -4.54587
H -0.05856 -1.11610 2.79496
H -0.43236 -0.02668 5.00227
H -1.43836 2.26173 5.11335
H -2.09702 3.43844 3.00855
H -1.73860 2.36158 0.80700
H -3.00679 -2.85856 -2.51293
H -2.82869 -5.34277 -2.65610
H -2.11837 -5.54437 1.60999
H -2.28589 -3.05920 1.75918
H -2.38223 -6.68879 -0.59689
H -5.86852 4.43595 -0.53018
C   2.33003 -1.91250 0.11998
O   1.86410 -0.56379 0.50709
C   1.71707 -2.97054 1.03656
C   2.05936 -2.15537 -1.36205
H   1.94482 -2.76056 2.09497
H   2.48927 -1.34820 -1.97661
H   2.13342 -3.96282 0.78933
H   0.62230 -3.01944 0.90774
H   2.52118 -3.10971 -1.66967
Si 3.10097 0.44206 1.23509
H   3.42838 -1.90905 0.26587
C   3.62382 -0.34139 2.86834
H   4.03659 -1.35891 2.75083
H   4.40118 0.27812 3.35300
H   2.76661 -0.39834 3.56311
C   2.39599 2.15584 1.53715
H   1.46500 2.14946 2.12870
H   3.15457 2.72180 2.11025

H   2.22550 2.70898 0.59851
C   4.53823 0.52870 0.01746
C   5.83465 0.07681 0.35806
C   4.34984 1.08264 -1.27255
C   6.90058 0.17633 -0.54917
C   5.41134 1.18155 -2.18225
C   6.68992 0.72832 -1.82071
H   6.02384 -0.35642 1.34737
H   3.35850 1.44374 -1.57489
H   7.89689 -0.17678 -0.26217
H   5.24431 1.61509 -3.17441
H   7.52100 0.80724 -2.52974
H   0.97641 -2.22569 -1.56217

TS(V-III)$_P$

SCF (TPSS/def2-SVP) Energy = -1791.62035328
Enthalpy 0K = -1791.059303
Enthalpy 298K = -1791.058359
Free Energy 298K = -1791.166669
Lowest Frequency = -3.5635 cm-1
Second Frequency = 9.7583 cm-1
SCF (M052X-D3,MeCN/def2-TZVP) Energy = -1792.76767199

P -0.43358 0.59868 1.12731
C -1.24148 1.85855 0.07322
C -0.75726 -1.01232 0.32308
C -0.63943 3.11468 -0.16472
C -0.54021 -2.14142 1.15177
C -1.28174 -1.20760 -0.97499
C -2.59288 1.66077 -0.30010
C -1.55638 -2.49984 -1.43701
C -1.35117 4.13364 -0.81056
C -2.68262 3.92472 -1.20188
C -1.30730 -3.61232 -0.61739
C -3.29813 2.69371 -0.93624
C -0.80131 -3.43299 0.67974
H -0.62661 -4.29738 1.32849
H -0.17399 -2.00317 2.17508
H -1.49737 -0.34959 -1.61664
H -1.97462 -2.63885 -2.43902
H -1.52357 -4.62104 -0.98503
H   0.39009 3.30036 0.15427
H -0.86611 5.09679 -1.00091
H -3.23919 4.72307 -1.70337
H -4.34014 2.52350 -1.22657
H -3.11646 0.71837 -0.09750
C   2.24207 1.10973 1.94589
O   1.35939 0.92031 0.69963
C   1.84329 2.39683 2.64916
C   2.21181 -0.14021 2.81056
H   1.94366 3.27124 1.98623
H   2.45704 -1.03731 2.22019
H   2.51292 2.54674 3.51387
H   0.80815 2.34222 3.02751
H   2.97648 -0.02988 3.59936
Si 2.34413 0.89577 -0.86263
H   3.24531 1.21166 1.50012
C   3.18638 2.57379 -0.93886
H   3.81374 2.81584 -0.06423
H   3.84491 2.58347 -1.82802
H   2.44778 3.38324 -1.07139
C   1.18837 0.66084 -2.30869
H   0.34043 1.36540 -2.30623
H   1.79729 0.86740 -3.21031
H   0.81008 -0.36935 -2.39500
C   3.54796 -0.51949 -0.64041
C   4.93170 -0.28447 -0.45395
C   3.09866 -1.86248 -0.69202
C   5.83336 -1.35046 -0.32209
C   3.99883 -2.92756 -0.55746
C   5.36709 -2.67228 -0.37227
H   5.31880 0.74092 -0.41991
H   2.03455 -2.08365 -0.83936
H   6.90082 -1.14911 -0.18428
H   3.63481 -3.95944 -0.60234
H   6.07077 -3.50532 -0.27153
H   1.23363 -0.26875 3.30282
C -5.73274 -1.29013 0.19096
O -4.59678 -0.97203 -0.14144
C -6.30126 -2.65531 -0.15651
C -6.63828 -0.34422 0.96181
H -5.57364 -3.22478 -0.75329
H -6.09776 0.57807 1.22215
H -7.24806 -2.54975 -0.71685
H -6.54103 -3.21204 0.76829
H -7.01773 -0.82916 1.87941
H -7.52392 -0.09204 0.34924

INT(V-III)$_P$

SCF (TPSS/def2-SVP) Energy = -1791.62665376
Enthalpy 0K = -1791.064563
Enthalpy 298K = -1791.063618
Free Energy 298K = -1791.170074
Lowest Frequency = 10.4271 cm-1
Second Frequency = 16.9389 cm-1
SCF (M052X-D3,MeCN/def2-TZVP) Energy = -1792.77213360

P   1.23206 0.16452 0.61275
C   1.87444 -1.11671 -0.54363
C   0.73261 1.61266 -0.40386
C   1.72257 -2.47164 -0.16921
C   0.59711 2.82125 0.31759
C   0.49929 1.62259 -1.79538
C   2.64800 -0.80948 -1.68630
C   0.12805 2.80906 -2.44374
C   2.28415 -3.49447 -0.94476
C   3.02435 -3.17953 -2.09484
C -0.00955 4.00057 -1.71509
C   3.21277 -1.83685 -2.45557
C   0.22938 4.00594 -0.33233
H   0.13172 4.93415 0.24007
H   0.78979 2.83650 1.39631
H   0.61137 0.70701 -2.38001
H -0.04914 2.80311 -3.52442
H -0.29720 4.92541 -2.22595
H   1.15929 -2.72807 0.73325
H   2.15035 -4.53940 -0.64571
H   3.46435 -3.97827 -2.70081
H   3.80633 -1.58446 -3.34056
H   2.82905 0.23264 -1.96317
C -1.16396 -0.66380 2.16829
O -0.66217 -0.68021 0.74729
C -0.32972 -1.60425 3.03109
C -1.25251 0.76343 2.69458
H -0.33801 -2.63167 2.63173
H -1.82171 1.40471 2.00320
H -0.75616 -1.63299 4.04886
H   0.71401 -1.25608 3.10824
H -1.77948 0.74964 3.66459
Si -1.86740 -1.40512 -0.37825
H -2.19220 -1.06006 2.08934
C -2.10232 -3.19193 0.16660
H -2.43765 -3.30518 1.21194
H -2.86828 -3.66223 -0.47812
H -1.16883 -3.76761 0.03834
C -1.24933 -1.34777 -2.14472
H -0.22922 -1.74351 -2.27756
H -1.93948 -1.99540 -2.71938
H -1.31426 -0.33978 -2.58333
C -3.43244 -0.38588 -0.17119
C -4.62405 -0.95387 0.33868
C -3.45819 0.97630 -0.55994
C -5.79696 -0.19291 0.45443
C -4.62804 1.73873 -0.44203
C -5.79955 1.15420 0.06506
H -4.64642 -2.00649 0.64503
H -2.55438 1.45144 -0.96136
H -6.71005 -0.65309 0.84666
H -4.62892 2.78987 -0.74994
H -6.71495 1.74887 0.15357

```
H  -0.25226  1.19690  2.86024
C   4.28558  1.01980  1.25084
O   3.34882  1.12605  0.44499
C   4.23263  0.08307  2.43060
C   5.53526  1.83296  1.03498
H   3.19054 -0.12185  2.72013
H   5.49972  2.35001  0.06559
H   4.81029  0.47189  3.28467
H   4.70133 -0.87258  2.12477
H   6.42947  1.18743  1.09994
H   5.62324  2.57740  1.84903
```

TS2(V-III)P

```
SCF (TPSS/def2-SVP) Energy = -1791.62391411
Enthalpy 0K = -1791.063405
Enthalpy 298K = -1791.062460
Free Energy 298K = -1791.171596
Lowest Frequency = -16.0539 cm-1
Second Frequency = 8.9228 cm-1
SCF (M052X-D3,MeCN/def2-TZVP) Energy = -
1792.77476077
```

```
P   1.80406  0.32370  0.55047
C   2.43377 -0.84128 -0.70702
C   1.59111  1.91775 -0.29032
C   1.65746 -2.00502 -0.91469
C   1.11437  2.97858  0.51958
C   1.77944  2.12981 -1.67546
C   3.63422 -0.65581 -1.43504
C   1.52256  3.38966 -2.22935
C   2.07365 -2.96095 -1.85335
C   3.26675 -2.77666 -2.56687
C   1.08058  4.44379 -1.41334
C   4.04760 -1.62709 -2.35451
C   0.87700  4.23914 -0.03878
H   0.51985  5.05854  0.59311
H   0.92979  2.81204  1.58755
H   2.11070  1.30952 -2.31905
H   1.66421  3.54977 -3.30298
H   0.88137  5.42650 -1.85332
H   0.71818 -2.13575 -0.36317
H   1.46509 -3.85541 -2.02171
H   3.59311 -3.52990 -3.29150
H   4.97836 -1.48513 -2.91361
H   4.24377  0.24137 -1.28129
C  -1.80499 -1.78270  1.72531
O  -1.40351 -1.30441  0.41615
C  -1.04392 -3.07249  2.03992
C  -1.56723 -0.68092  2.75958
H  -1.23546 -3.83902  1.26999
H  -2.15092  0.21926  2.50550
H  -1.35317 -3.48036  3.01826
H   0.04465 -2.88131  2.07611
H  -1.87055 -1.01877  3.76627
Si -2.56656 -1.09259 -0.82427
H  -2.89074 -2.01208  1.70895
C  -3.23880 -2.77516 -1.35500
H  -3.73029 -3.31413 -0.52453
H  -3.97974 -2.67155 -2.16912
H  -2.41918 -3.41560 -1.72941
C  -1.62120 -0.27094 -2.23061
H  -0.78655 -0.90353 -2.58366
H  -2.29742 -0.09753 -3.08774
H  -1.20799  0.70864 -1.93025
C  -3.96203  0.02212 -0.20403
C  -5.28901 -0.44590 -0.06156
C  -3.70476  1.37398  0.13134
C  -6.31479  0.39556  0.39622
C  -4.72502  2.21912  0.58903
C  -6.03406  1.72973  0.72267
H  -5.53400 -1.48416 -0.31493
H  -2.68881  1.77773  0.03148
H  -7.33498  0.00923  0.49618
H  -4.50295  3.26268  0.83899
H  -6.83349  2.38891  1.07807
H  -0.49684 -0.40821  2.78716
```

```
C   4.16054  0.09130  2.12135
O   3.52565  0.77546  1.26441
C   3.68777 -1.25401  2.55430
C   5.41243  0.68655  2.66840
H   2.59228 -1.23459  2.71117
H   5.64079  1.65014  2.19248
H   4.21113 -1.60689  3.45442
H   3.87049 -1.96892  1.72689
H   6.24785 -0.02572  2.53344
H   5.29449  0.81760  3.76178
```

TS(PH1)

```
SCF (TPSS/def2-SVP) Energy = -1944.66321340
Enthalpy 0K = -1944.086491
Enthalpy 298K = -1944.085547
Free Energy 298K = -1944.199081
Lowest Frequency = -79.7504 cm-1
Second Frequency = 5.9071 cm-1
SCF (M052X-D3,MeCN/def2-TZVP) Energy = -
1945.88470815
```

```
P   0.08176 -0.03465 -0.44768
C   3.69404  0.71278  0.48270
C   0.07524 -0.38599  1.34655
C   1.55912  2.02678 -0.41982
C   0.70996  3.13132 -0.78711
C   2.54981  1.39382 -0.00550
C   1.13160 -1.30367 -1.25576
C   4.01285  0.77741  1.86812
C   4.55601  0.00190 -0.39584
C  -0.99991  0.19399  2.06460
C   1.70055 -0.93979 -2.50024
C   1.26411 -2.63943 -0.80955
C   5.16228  0.14830  2.35100
C   6.00383 -0.55734  1.47393
C   1.07416 -1.09426  2.05391
C   5.69739 -0.62890  0.10433
C   0.59798  4.23003  0.10080
C  -0.86715  5.36148 -1.47390
C   0.01132  3.16148 -2.01714
C  -0.18588  5.33530 -0.24717
C   1.96863 -3.57540 -1.57906
C  -1.08765  0.04227  3.45308
C  -0.09637 -0.67235  4.14499
C   2.54679 -3.19617 -2.80042
C   0.98408 -1.23256  3.44552
C   2.41006 -1.87699 -3.26131
C  -0.76531  4.27363 -2.35633
H   5.40871  0.21059  3.41585
H   3.35459  1.33111  2.54370
H   4.32105 -0.03857 -1.46258
H   6.35973 -1.17193 -0.57737
H   2.84972 -1.57998 -4.21911
H   1.59194  0.08656 -2.86930
H   0.79942 -2.95651  0.12894
H   2.06153 -4.60750 -1.22512
H   3.09560 -3.93135 -3.39815
H  -1.77239  0.75931  1.53037
H  -1.92878  0.48515  3.99611
H  -0.16309 -0.78797  5.23188
H   1.76382 -1.77925  3.98616
H   1.92967 -1.52257  1.52452
H   0.08694  2.31283 -2.70384
H  -1.29307  4.29436 -3.31538
H  -0.26226  6.18228  0.44233
H   1.13547  4.20856  1.05344
H  -1.47694  6.22991 -1.74352
H   6.90510 -1.04651  1.85775
Si -2.76213 -1.86271 -0.98123
C  -2.49076 -3.61119 -0.34251
H  -2.22699 -3.62589  0.72954
H  -3.41446 -4.20549 -0.47454
H  -1.68423 -4.11245 -0.90564
C  -2.90986 -1.74949 -2.85314
H  -3.82289 -2.27568 -3.18888
H  -2.97653 -0.70347 -3.20010
```

```
H  -2.04187 -2.22200 -3.34635
H  -1.41314 -1.07895 -0.67984
C  -4.07109 -0.90380 -0.04282
C  -4.61322 -1.39970  1.16805
C  -4.54463  0.34171 -0.52508
C  -5.59819 -0.68463  1.86370
C  -5.52533  1.05930  0.17210
C  -6.05415  0.54590  1.36725
H  -4.27319 -2.36048  1.57138
H  -4.14947  0.75799 -1.45960
H  -6.01396 -1.09033  2.79215
H  -5.88343  2.01742 -0.21929
H  -6.82506  1.10401  1.90922
```

INT(PH1)

```
SCF (TPSS/def2-SVP) Energy = -1405.46480865
Enthalpy 0K = -1405.089936
Enthalpy 298K = -1405.088992
Free Energy 298K = -1405.170627
Lowest Frequency = 9.9932 cm-1
Second Frequency = 17.1313 cm-1
SCF (M052X-D3,MeCN/def2-TZVP) Energy = -
1406.27222346

P   0.94519  0.13633 -1.21697
C   0.75221  1.80173 -0.49558
C   2.49769 -0.54248 -0.54237
C  -0.53273  2.39482 -0.58271
C   3.25705 -1.31915 -1.45095
C   2.96701 -0.37092  0.78349
C   1.84211  2.57920 -0.03797
C   4.17235 -0.95874  1.18133
C  -0.72757  3.71806 -0.17553
C   0.35941  4.47936  0.28802
C   4.92671 -1.71406  0.26517
C   1.64092  3.91230  0.34516
C   4.47139 -1.89206 -1.04922
H   5.05931 -2.47917 -1.76179
H   2.89775 -1.46357 -2.47646
H   2.38944  0.22208  1.50049
H   4.53014 -0.82750  2.20781
H   5.87220 -2.16664  0.58162
H  -1.38067  1.81850 -0.97152
H  -1.72564  4.16408 -0.23451
H   0.20760  5.52010  0.59210
H   2.49178  4.51028  0.68717
H   2.84969  2.15440 -0.00303
Si -1.05509 -1.55743  0.76783
C  -0.52673 -1.02084  2.48069
H  -0.62453  0.06847  2.62814
H  -1.17336 -1.52414  3.22533
H   0.51646 -1.31588  2.68427
C  -0.47182 -3.24496  0.21248
H  -1.01046 -4.01760  0.79430
H  -0.66993 -3.42732 -0.85751
H   0.60892 -3.37130  0.39831
H  -0.06555 -0.59032 -0.22350
C  -2.74026 -1.01756  0.21784
C  -3.45820 -0.03463  0.94808
C  -3.34525 -1.58263 -0.93479
C  -4.74093  0.35670  0.54639
C  -4.62625 -1.18615 -1.33493
C  -5.32402 -0.21739 -0.59484
H  -3.01963  0.42077  1.84319
H  -2.81757 -2.34255 -1.52230
H  -5.28943  1.10713  1.12487
H  -5.08439 -1.63415 -2.22251
H  -6.32731  0.08985 -0.90812
```

I$_{CF3}$

```
SCF (TPSS/def2-SVP) Energy = -2016.94717988
Enthalpy 0K = -2016.535722
Enthalpy 298K = -2016.534778
Free Energy 298K = -2016.636445
Lowest Frequency = 8.7376 cm-1
Second Frequency = 12.0685 cm-1
SCF (M052X-D3,MeCN/def2-TZVP) Energy = -
2018.58433483

P   0.00217  1.72828 -0.00029
C  -1.92294 -0.66146  0.00304
C   0.08523  2.69084 -1.52318
C   0.67954  0.08805  0.03319
C   1.91471 -0.67056  0.05462
C  -0.68412  0.09141  0.00868
C  -0.07544  2.73072  1.49701
C  -1.94185 -2.06035  0.22767
C  -3.14596  0.01732 -0.21730
C  -0.61380  3.91412 -1.62935
C  -0.79499  2.24916  2.61220
C   0.62583  3.95518  1.56926
C  -3.15115 -2.75689  0.22052
C  -4.35671 -2.07244 -0.01641
C   0.80673  2.17893 -2.62348
C  -4.35302 -0.68360 -0.23110
C   1.92645 -2.07456 -0.13542
C   4.34218 -2.09172  0.09905
C   3.14073  0.00658  0.26357
C   3.13257 -2.77649 -0.11435
C   0.59889  4.69212  2.76010
C  -0.58259  4.61946 -2.83907
C   0.13721  4.11338 -3.93273
C  -0.11893  4.21620  3.86848
C   0.82982  2.89664 -3.82509
C  -0.81382  2.99823  3.79456
C   4.34437 -0.69957  0.29185
H  -3.16577 -3.83444  0.40735
H  -1.01108 -2.59732  0.42797
H  -3.14156  1.09967 -0.38322
H  -5.29619 -0.15665 -0.40025
H  -1.37531  2.63149  4.65946
H  -1.33763  1.29948  2.55595
H   1.18883  4.32982  0.70791
H   1.14278  5.63991  2.82210
H  -0.13775  4.79817  4.79549
H  -1.17838  4.31223 -0.77963
H  -1.12472  5.56616 -2.92738
H   0.15934  4.67076 -4.87466
H   1.39284  2.50633 -4.67861
H   1.34762  1.23017 -2.54094
H   3.14062  1.09112  0.41469
H   5.28821 -0.17604  0.46805
H   3.14000 -3.86063 -0.25853
H   0.99213 -2.61275 -0.31466
C   5.65530 -2.85310  0.07227
F   5.49650 -4.12256  0.50310
F   6.14954 -2.90742 -1.18604
F   6.58486 -2.25805  0.84989
C  -5.66214 -2.84497 -0.07878
F  -6.70965 -2.07099  0.27765
F  -5.89271 -3.29252 -1.33401
F  -5.63624 -3.91928  0.73880
```

II$_{CF3}$

```
SCF (TPSS/def2-SVP) Energy = -2016.91508304
Enthalpy 0K = -2016.504766
Enthalpy 298K = -2016.503822
Free Energy 298K = -2016.606098
Lowest Frequency = 8.9477 cm-1
Second Frequency = 11.6028 cm-1
SCF (M052X-D3,MeCN/def2-TZVP) Energy = -
2018.54418609

P   0.46159  1.88138  0.33529
C  -1.40871 -1.00117 -0.12592
C  -0.75686  1.97427  1.68590
C   1.04393  0.06420  0.08765
C   2.46596 -0.30469  0.06069
C  -0.11157 -0.48893 -0.03929
C  -0.29040  2.44385 -1.22244
C  -2.08049 -1.07218 -1.38544
```

C -2.06954 -1.47084 1.05204
C -0.22676 2.10177 2.99181
C 0.13093 1.90017 -2.45914
C -1.13619 3.57844 -1.21442
C -3.36752 -1.59789 -1.45537
C -4.00056 -2.06787 -0.28788
C -2.15837 1.96939 1.49440
C -3.35270 -2.00211 0.96138
C 2.82021 -1.65946 -0.14620
C 5.16886 -1.06408 0.00128
C 3.47807 0.65963 0.23124
C 4.16341 -2.03323 -0.17523
C -1.58052 4.12893 -2.42408
C -1.08871 2.21010 4.08986
C -2.47884 2.21223 3.89336
C -1.18054 3.56943 -3.64641
C -3.01074 2.09412 2.59889
C -0.32245 2.45761 -3.66068
C 4.82462 0.28129 0.20037
H -3.89086 -1.64935 -2.41422
H -1.57653 -0.71186 -2.28610
H -1.55644 -1.41331 2.01570
H -3.86375 -2.36363 1.85823
H 0.00307 2.02672 -4.61308
H 0.81451 1.04451 -2.48582
H -1.43963 4.04020 -0.26934
H -2.23851 5.00362 -2.40794
H -1.52805 4.00500 -4.58858
H 0.85813 2.12440 3.14438
H -0.67454 2.30908 5.09822
H -3.15091 2.31285 4.75184
H -4.09530 2.09820 2.44869
H -2.57898 1.87509 0.48825
H 3.20852 1.70996 0.38788
H 5.60991 1.03179 0.32534
H 4.43762 -3.07908 -0.34165
H 2.03923 -2.41384 -0.28500
C -5.38330 -2.69486 -0.38071
F -6.07643 -2.19860 -1.42563
F -5.28293 -4.03083 -0.54538
F -6.09459 -2.46882 0.74353
C 6.62516 -1.49057 0.01631
F 7.44582 -0.46491 -0.29510
F 6.98013 -1.94158 1.24149
F 6.85025 -2.49041 -0.86425

TS(I-II)$_{CF3}$

SCF (TPSS/def2-SVP) Energy = -2016.91412199
Enthalpy 0K = -2016.504860
Enthalpy 298K = -2016.503915
Free Energy 298K = -2016.605199
Lowest Frequency = -189.1008 cm-1
Second Frequency = 8.9234 cm-1
SCF (M052X-D3,MeCN/def2-TZVP) Energy = -2018.54660553

P 0.34062 1.79583 0.18375
C -1.44953 -0.93709 -0.10658
C -0.69446 2.06950 1.64921
C 1.05297 0.06821 0.03216
C 2.46457 -0.31262 0.01672
C -0.16438 -0.35001 -0.05295
C -0.40510 2.38966 -1.36072
C -2.14601 -1.05549 -1.34261
C -2.05611 -1.41253 1.09173
C -0.02973 2.32530 2.87019
C -0.00653 1.83378 -2.59769
C -1.25100 3.52075 -1.33564
C -3.40882 -1.64524 -1.37270
C -3.99281 -2.12460 -0.18564
C -2.10726 2.05606 1.59698
C -3.31635 -2.00618 1.04240
C 2.80545 -1.68093 -0.09951
C 5.15831 -1.08762 -0.01176
C 3.48074 0.65822 0.11218
C 4.14771 -2.06166 -0.11328
C -1.72029 4.06194 -2.54068
C -0.77752 2.55208 4.03240
C -2.18011 2.54543 3.97852
C -1.34296 3.49623 -3.76707
C -2.84181 2.30133 2.76405
C -0.48405 2.38546 -3.79273
C 4.82419 0.27155 0.09663
H -3.94915 -1.73661 -2.31915
H -1.68528 -0.68708 -2.26284
H -1.52519 -1.31630 2.04266
H -3.78441 -2.37586 1.95924
H -0.17692 1.94925 -4.74871
H 0.67592 0.97739 -2.63107
H -1.53752 3.98425 -0.38643
H -2.38043 4.93476 -2.51644
H -1.70991 3.92578 -4.70453
H 1.06456 2.35167 2.90881
H -0.26320 2.74860 4.97837
H -2.76129 2.73634 4.88648
H -3.93593 2.29710 2.72547
H -2.62695 1.85885 0.65399
H 3.21306 1.71766 0.19506
H 5.61545 1.02334 0.16221
H 4.41665 -3.11760 -0.20936
H 2.01915 -2.43784 -0.17922
C -5.34517 -2.81539 -0.23493
F -6.09478 -2.35266 -1.25746
F -5.19332 -4.14680 -0.40484
F -6.03260 -2.62334 0.91129
C 6.61344 -1.51877 0.02267
F 7.43277 -0.52874 -0.39078
F 6.98214 -1.85762 1.27934
F 6.82333 -2.59476 -0.76630

TS(II-III)$_{H,CF3}$

SCF (TPSS/def2-SVP) Energy = -2618.28572298
Enthalpy 0K = -2617.692896
Enthalpy 298K = -2617.691951
Free Energy 298K = -2617.821986
Lowest Frequency = -94.0001 cm-1
Second Frequency = 5.8237 cm-1
SCF (M052X-D3,MeCN/def2-TZVP) Energy = -2620.22861608

P 1.05305 2.43278 1.01799
C -1.25280 0.12068 -0.28601
C -0.60063 2.55358 1.79435
C 1.28630 0.66448 0.30906
C 2.67532 0.16633 0.21505
C 0.13319 0.16035 -0.03398
C 1.01797 3.51543 -0.45426
C -1.76187 0.57102 -1.54030
C -2.15581 -0.36865 0.70170
C -0.69404 2.11608 3.13680
C 1.45397 3.08769 -1.72807
C 0.69815 4.88121 -0.25753
C -3.13245 0.54389 -1.78422
C -4.01149 0.05405 -0.79746
C -1.73258 3.12446 1.17014
C -3.52465 -0.39273 0.44368
C 3.05755 -0.65399 -0.87162
C 5.31018 -0.80347 0.02185
C 3.63464 0.50718 1.19112
C 4.36641 -1.13439 -0.96581
C 0.78651 5.78612 -1.32223
C -1.90318 2.23178 3.83401
C -3.02176 2.80555 3.20817
C 1.20530 5.34933 -2.58875
C -2.93397 3.25265 1.88024
C 1.54049 4.00155 -2.78722
C 4.94126 0.01623 1.10022
H -3.53006 0.90054 -2.73871
H -1.06944 0.94487 -2.29969
H -1.76802 -0.71217 1.66338
H -4.22091 -0.76195 1.20073
H 1.87520 3.65821 -3.77162

```
H   1.72952  2.04173 -1.89642
H   0.38470  5.23974  0.72930
H   0.53252  6.83845 -1.15892
H   1.27662  6.06025 -3.41813
H   0.18744  1.69975  3.63845
H  -1.96646  1.89538  4.87404
H  -3.96089  2.91459  3.76065
H  -3.80494  3.70568  1.39530
H  -1.67080  3.47601  0.13547
H   3.35131  1.15127  2.02984
H   5.67645  0.26680  1.87009
H   4.66038 -1.76499 -1.81022
H   2.33029 -0.89546 -1.65231
Si  0.41304 -3.26611 -0.02383
C   1.33936 -3.74282  1.54687
H   0.93378 -3.23897  2.44216
H   1.28143 -4.83419  1.71436
H   2.40646 -3.47069  1.46084
C   1.19397 -3.99458 -1.57971
H   1.09299 -5.09558 -1.56650
H   0.71470 -3.62521 -2.50369
H   2.27092 -3.75523 -1.63117
H   0.51193 -1.72476 -0.14745
C  -1.43770 -3.61418  0.07335
C  -2.07284 -3.91986  1.30296
C  -2.24251 -3.57010 -1.09270
C  -3.45036 -4.17606  1.36396
C  -3.62005 -3.81803 -1.03457
C  -4.22663 -4.12109  0.19565
H  -1.48338 -3.97591  2.22595
H  -1.78759 -3.34743 -2.06565
H  -3.91787 -4.42762  2.32215
H  -4.22350 -3.77995 -1.94728
H  -5.30249 -4.32149  0.24065
C  -5.49643 -0.03847 -1.10198
F  -5.78216 -1.20767 -1.72378
F  -6.23749  0.01646  0.02416
F  -5.89380  0.96202 -1.91595
C   6.74015 -1.28964 -0.11312
F   7.35309 -1.36815  1.08771
F   6.78901 -2.51343 -0.68774
F   7.46662 -0.45115 -0.88771
```

$III_{H,CF3}$

```
SCF (TPSS/def2-SVP) Energy = -2618.33747780
Enthalpy 0K = -2617.739381
Enthalpy 298K = -2617.738437
Free Energy 298K = -2617.863018
Lowest Frequency = 10.0377 cm-1
Second Frequency = 15.4496 cm-1
SCF (M052X-D3,MeCN/def2-TZVP) Energy = -
2620.30668451
```

```
P   0.24260  0.44675  1.29532
C  -1.48282 -0.13354 -1.48995
C  -1.51963  0.55746  1.69008
C   0.76028 -0.10453 -0.27540
C   2.21753 -0.02972 -0.52608
C  -0.10911 -0.76497 -1.27619
C   1.11357  1.92607  1.88273
C  -1.54446  1.10539 -2.16351
C  -2.68615 -0.71621 -1.05332
C  -2.17424 -0.54806  2.27639
C   1.67907  2.88192  1.00987
C   1.16805  2.12666  3.28086
C  -2.76670  1.75219 -2.37811
C  -3.95551  1.16521 -1.91637
C  -2.20706  1.77907  1.50178
C  -3.91362 -0.07643 -1.26423
C   2.69827  0.33046 -1.81122
C   4.99198  0.19407 -1.02929
C   3.16752 -0.29250  0.49455
C   4.06877  0.44869 -2.05639
C   1.78059  3.27564  3.79628
C  -3.52184 -0.43452  2.64379
C  -4.21237  0.76772  2.43088
C   2.34686  4.22124  2.92790
C  -3.55621  1.87161  1.85949
C   2.29386  4.02320  1.53865
C   4.53610 -0.18541  0.24535
H  -2.80011  2.70936 -2.90683
H  -0.62322  1.57208 -2.53160
H  -2.67095 -1.67147 -0.52138
H  -4.84136 -0.54141 -0.91877
H   2.72469  4.76715  0.86077
H   1.63328  2.74046 -0.07393
H   0.73035  1.38973  3.96306
H   1.82016  3.42861  4.87942
H   2.82815  5.11690  3.33354
H  -1.63454 -1.48463  2.44600
H  -4.02964 -1.28851  3.10322
H  -5.26583  0.85140  2.71755
H  -4.09997  2.80512  1.68607
H  -1.69429  2.64301  1.06770
H   2.82138 -0.62227  1.47971
H   5.25702 -0.41616  1.03491
H   4.42468  0.73715 -3.04960
H   1.99401  0.56143 -2.61687
Si -0.03955 -2.77752 -1.17652
C   1.75710 -3.26737 -1.47567
H   2.44453 -2.95593 -0.67118
H   1.80493 -4.37031 -1.54344
H   2.13907 -2.85785 -2.42808
C  -1.10480 -3.38611 -2.60805
H  -1.02620 -4.48761 -2.66802
H  -2.17156 -3.12280 -2.52022
H  -0.73374 -2.97331 -3.56363
H   0.45197 -0.71773 -2.23288
C  -0.62030 -3.42021  0.49934
C   0.23989 -3.37743  1.62546
C  -1.89084 -4.02287  0.66502
C  -0.15562 -3.90482  2.86418
C  -2.28929 -4.55258  1.90118
C  -1.42326 -4.49100  3.00418
H   1.24459 -2.94815  1.53278
H  -2.57751 -4.10089 -0.18644
H   0.52979 -3.87044  3.71784
H  -3.27172 -5.02638  2.00072
H  -1.72994 -4.91190  3.96760
C  -5.27112  1.89861 -2.05810
F  -6.31072  1.04556 -2.18559
F  -5.51677  2.66253 -0.95710
F  -5.27514  2.72731 -3.12364
C   6.47847  0.36178 -1.28330
F   7.20474 -0.49269 -0.53117
F   6.78540  0.14612 -2.58007
F   6.87682  1.61750 -0.97280
```

I_{OMe}

```
SCF (TPSS/def2-SVP) Energy = -1572.25427293
Enthalpy 0K = -1571.789602
Enthalpy 298K = -1571.788658
Free Energy 298K = -1571.881051
Lowest Frequency = 17.9000 cm-1
Second Frequency = 22.5815 cm-1
SCF (M052X-D3,MeCN/def2-TZVP) Energy = -
1573.36422425
```

```
P   0.12088  1.24746 -0.00436
C  -2.09095 -0.85193 -0.00284
C   0.27873  2.22189 -1.51916
C   0.59307 -0.45709 -0.00127
C   1.72504 -1.34858  0.03060
C  -0.76723 -0.28223  0.01536
C   0.21609  2.25198  1.49591
C  -2.30856 -2.24724 -0.11941
C  -3.22816 -0.00196  0.08533
C  -0.26577  3.52267 -1.59341
C  -0.52502  1.86130  2.63164
C   1.07167  3.37396  1.55275
C  -3.59777 -2.77943 -0.13937
C  -4.71689 -1.91884 -0.04163
```

```
C   0.89044  1.63864 -2.64940
C  -4.51441 -0.52067  0.06924
C   1.57188 -2.75728  0.16040
C   3.98715 -3.05524  0.10099
C   3.03868 -0.82574 -0.05433
C   2.67861 -3.59269  0.19464
C   1.17543  4.10176  2.74531
C  -0.18884  4.23418 -2.79781
C   0.42401  3.65660 -3.92052
C   0.43470  3.71668  3.87348
C   0.96184  2.36181 -3.84614
C  -0.41297  2.59892  3.81643
C   4.15862 -1.65671 -0.02301
H  -3.72865 -3.86004 -0.23351
H  -1.45588 -2.92606 -0.20674
H  -3.08399  1.08109  0.16624
H  -5.39276  0.12719  0.13770
H  -0.99145  2.30148  4.69675
H  -1.18494  0.98818  2.58752
H   1.65369  3.67722  0.67605
H   1.83928  4.97081  2.79315
H   0.51806  4.29066  4.80200
H  -0.74826  3.97674 -0.72145
H  -0.61234  5.24167 -2.85928
H   0.48256  4.21809 -4.85853
H   1.44054  1.91370 -4.72258
H   1.30733  0.62734 -2.59175
H   3.17979  0.25707 -0.14503
H   5.15647 -1.21712 -0.09128
H   2.57033 -4.67614  0.29709
H   0.57219 -3.19148  0.24549
O   4.99182 -3.95054  0.14258
C   6.34844 -3.49852  0.05957
H   6.53463 -2.97991 -0.89840
H   6.59458 -2.82930  0.90386
H   6.96625 -4.40528  0.11479
O  -5.99975 -2.32700 -0.04819
C  -6.30023 -3.72329 -0.15996
H  -5.91937 -4.13482 -1.11221
H  -5.87699 -4.28808  0.69040
H  -7.39649 -3.79219 -0.14013
```

TS(I-II)$_{OMe}$

SCF (TPSS/def2-SVP) Energy = -1572.22284293
Enthalpy 0K = -1571.760249
Enthalpy 298K = -1571.759304
Free Energy 298K = -1571.851822
Lowest Frequency = -262.6543 cm-1
Second Frequency = 17.4739 cm-1
SCF (M052X-D3,MeCN/def2-TZVP) Energy = -1573.32911829

```
P  -0.25157 -1.47620  0.34432
C   1.23498  1.28764 -0.42480
C   0.83765 -1.51201  1.79601
C  -1.15512  0.10983  0.02499
C  -2.58249  0.37574  0.04831
C   0.04496  0.54950 -0.20778
C   0.43046 -2.29121 -1.12997
C   1.92464  1.23814 -1.67401
C   1.77300  2.09016  0.61986
C   0.24682 -1.70396  3.06426
C  -0.06288 -1.95858 -2.41051
C   1.35002 -3.35102 -0.98076
C   3.08254  1.97446 -1.86614
C   3.60085  2.78606 -0.82377
C   2.23766 -1.36194  1.67854
C   2.93334  2.83636  0.42392
C  -3.05582  1.67359 -0.25649
C  -5.34841  0.93776  0.09098
C  -3.51714 -0.63662  0.37234
C  -4.42160  1.95703 -0.23578
C   1.79405 -4.04753 -2.11391
C   1.05462 -1.73280  4.20841
C   2.44610 -1.59282  4.09098
C   1.31859 -3.70665 -3.38822
C   3.03506 -1.41174  2.82931
C   0.38834 -2.66498 -3.53260
C  -4.87854 -0.36332  0.39441
H   3.61548  1.95579 -2.82097
H   1.52835  0.62104 -2.48513
H   1.26048  2.12904  1.58553
H   3.31416  3.45561  1.23962
H   0.00579 -2.40231 -4.52426
H  -0.79749 -1.15507 -2.53247
H   1.71639 -3.63719  0.01027
H   2.51261 -4.86478 -1.99448
H   1.66603 -4.25675 -4.26842
H  -0.83690 -1.83363  3.15375
H   0.59669 -1.87951  5.19180
H   3.07532 -1.62967  4.98625
H   4.12087 -1.30282  2.74013
H   2.69844 -1.21014  0.69722
H  -3.15775 -1.64589  0.60447
H  -5.61165 -1.13612  0.64158
H  -4.76096  2.96804 -0.47346
H  -2.34516  2.46679 -0.51003
O   4.72189  3.46568 -1.11792
C   5.31505  4.32664 -0.13610
H   4.61614  5.13087  0.15565
H   5.62479  3.75277  0.75574
H   6.19896  4.76185 -0.62172
O  -6.68377  1.10545  0.13838
C  -7.24914  2.38781 -0.15718
H  -6.89229  3.15201  0.55700
H  -7.00948  2.69750 -1.19064
H  -8.33561  2.26326 -0.05192
```

II$_{OMe}$

SCF (TPSS/def2-SVP) Energy = -1572.22914097
Enthalpy 0K = -1571.765311
Enthalpy 298K = -1571.764367
Free Energy 298K = -1571.857722
Lowest Frequency = 9.8735 cm-1
Second Frequency = 20.9128 cm-1
SCF (M052X-D3,MeCN/def2-TZVP) Energy = -1573.33442317

```
P   0.48914  1.60683  0.75707
C  -1.19074 -1.31272 -0.49100
C  -1.01100  1.34883  1.78124
C   1.10662 -0.12424  0.19023
C   2.53995 -0.43840  0.20920
C   0.02778 -0.74499 -0.17275
C  -0.03997  2.45390 -0.77627
C  -1.73158 -1.21913 -1.82265
C  -1.93932 -2.03262  0.50057
C  -0.79029  1.13613  3.16163
C   0.48784  2.09752 -2.03767
C  -0.83692  3.61784 -0.66742
C  -2.93675 -1.81292 -2.13260
C  -3.65796 -2.53452 -1.13733
C  -2.33455  1.40803  1.29110
C  -3.14562 -2.63649  0.18561
C   2.99272 -1.68311 -0.28846
C   5.29487 -1.09146  0.22318
C   3.49767  0.47336  0.71338
C   4.34778 -2.01322 -0.28451
C  -1.12667  4.38330 -1.80470
C  -1.87501  0.97023  4.03242
C  -3.18769  1.03881  3.53948
C  -0.61599  4.01123 -3.05726
C  -3.41487  1.26010  2.17200
C   0.19395  2.87075 -3.16903
C   4.85134  0.15565  0.72127
H  -3.36416 -1.75542 -3.13756
H  -1.17241 -0.66982 -2.58510
H  -1.53926 -2.10285  1.51599
H  -3.69299 -3.18504  0.95567
H   0.60566  2.58267 -4.14205
H   1.13304  1.21816 -2.13810
H  -1.22869  3.93180  0.30624
```

```
H  -1.75017  5.27813 -1.70808
H  -0.84122  4.61375 -3.94310
H   0.23310  1.11154  3.55342
H  -1.69528  0.80778  5.10015
H  -4.03507  0.93015  4.22459
H  -4.43895  1.32073  1.78847
H  -2.51905  1.58098  0.22595
H   3.16850  1.44115  1.10766
H   5.59611  0.85549  1.11039
H   4.66294 -2.98372 -0.67544
H   2.26868 -2.40313 -0.68544
O  -4.80402 -3.08026 -1.54495
C  -5.61813 -3.84741 -0.63725
H  -5.06073 -4.72370 -0.26420
H  -5.95699 -3.21917  0.20445
H  -6.48141 -4.17677 -1.23019
O   6.62454 -1.30911  0.27344
C   7.15724 -2.54705 -0.20788
H   6.75637 -3.40204  0.36674
H   6.93867 -2.68232 -1.28299
H   8.24378 -2.48026 -0.05874
```

TS(II-III)$_{H,OMe}$

SCF (TPSS/def2-SVP) Energy = -2173.59351295
Enthalpy 0K = -2172.947594
Enthalpy 298K = -2172.946650
Free Energy 298K = -2173.067388
Lowest Frequency = -189.7182 cm-1
Second Frequency = 8.8414 cm-1
SCF (M052X-D3,MeCN/def2-TZVP) Energy = -
2175.01636501

```
P   1.29798  2.01941  1.23579
C  -1.20587  0.31314 -0.63029
C  -0.43844  2.48430  1.62321
C   1.25789  0.33768  0.35123
C   2.53818 -0.39494  0.33365
C   0.08383  0.01199 -0.15735
C   1.81799  3.18885 -0.08311
C  -1.37257  1.00537 -1.87446
C  -2.37376 -0.06441  0.09762
C  -0.96996  1.98492  2.83451
C   2.20935  2.79074 -1.37832
C   1.93471  4.55120  0.28083
C  -2.63451  1.32370 -2.34063
C  -3.78596  0.94946 -1.59530
C  -1.21363  3.36976  0.84422
C  -3.64297  0.25061 -0.36923
C   2.92345 -1.12738 -0.81200
C   5.00133 -1.79983  0.25843
C   3.42718 -0.36311  1.43735
C   4.13723 -1.82261 -0.85927
C   2.40549  5.49288 -0.64233
C  -2.25863  2.34836  3.24673
C  -3.02149  3.23210  2.46667
C   2.78415  5.08901 -1.93249
C  -2.49664  3.74274  1.26923
C   2.68874  3.73809 -2.29534
C   4.62941 -1.06135  1.40709
H  -2.77943  1.86136 -3.28191
H  -0.48656  1.29217 -2.44802
H  -2.26394 -0.60386  1.04147
H  -4.51814 -0.04635  0.21269
H   2.99013  3.41638 -3.29794
H   2.14765  1.73823 -1.67260
H   1.65686  4.87719  1.28999
H   2.48508  6.54493 -0.34929
H   3.15879  5.82580 -2.65048
H  -0.36264  1.32299  3.46333
H  -2.65845  1.96117  4.18996
H  -4.01945  3.53517  2.80120
H  -3.08580  4.44102  0.66509
H  -0.80830  3.77687 -0.08750
H   3.15760  0.20996  2.33059
H   5.31278 -1.05050  2.26101
H   4.41218 -2.35870 -1.77120
H   2.27527 -1.12608 -1.69467
Si  0.06928 -3.25385 -0.15470
C   1.11214 -3.75759  1.33046
H   0.80854 -3.22778  2.25049
H   1.01262 -4.84373  1.51363
H   2.17794 -3.53411  1.14906
C   0.67027 -4.00518 -1.77779
H   0.48963 -5.09626 -1.77280
H   0.15011 -3.58087 -2.65480
H   1.75387 -3.84055 -1.90882
H   0.19808 -1.69565 -0.29461
C  -1.77341 -3.55027  0.11694
C  -2.31493 -3.65658  1.42188
C  -2.66175 -3.67799 -0.97867
C  -3.68354 -3.88532  1.62376
C  -4.03045 -3.90807 -0.78080
C  -4.54396 -4.01107  0.52154
H  -1.65894 -3.57519  2.29686
H  -2.28188 -3.60889 -2.00490
H  -4.07800 -3.97831  2.64147
H  -4.69603 -4.01856 -1.64394
H  -5.61139 -4.20145  0.67800
O  -4.96148  1.29820 -2.13515
C  -6.18956  0.95880 -1.47186
H  -6.28878 -0.13626 -1.36996
H  -6.24306  1.43914 -0.47896
H  -6.98838  1.34756 -2.11755
O   6.19437 -2.42935  0.32192
C   6.65307 -3.18128 -0.80411
H   5.96005 -4.01043 -1.03905
H   6.77503 -2.53413 -1.69221
H   7.62974 -3.59079 -0.51096
```

III$_{H,OMe}$

SCF (TPSS/def2-SVP) Energy = -2173.34754984
A.U
Enthalpy 0K = -2172.687192
Enthalpy 298K = -2172.686248
Free Energy 298K = -2172.803056
Lowest Frequency = 8.4888 cm-1
Second Frequency = 17.1964 cm-1
SCF (M052X-D3,MeCN/def2-TZVP) Energy = -
2175.12600199

```
P   1.38981 -0.20255  0.63618
C  -2.75821 -0.71446  0.49011
C   1.81562  0.23684  2.36899
C  -0.36477  0.22987  0.32909
C  -0.61767  1.62621 -0.13243
C  -1.30121 -0.73083  0.56763
C   2.42423  0.81897 -0.46983
C  -3.55406  0.40919  0.14806
C  -3.43555 -1.91607  0.79170
C   3.15704  0.43972  2.74510
C   2.74537  0.33890 -1.75216
C   2.83349  2.10983 -0.09074
C  -4.93410  0.32060  0.10977
C  -5.59138 -0.89261  0.41048
C   0.81009  0.28269  3.34966
C  -4.82359 -2.01979  0.75553
C  -0.72035  2.69538  0.78190
C  -1.09701  4.26889 -1.02945
C  -0.74799  1.91487 -1.49956
C  -0.95915  3.99271  0.34490
C   3.56848  2.89825 -0.97848
C   3.47971  0.69413  4.07994
C   2.47395  0.74273  5.05093
C   3.89670  2.41158 -2.24762
C   1.14145  0.53645  4.68404
C   3.48190  1.13395 -2.63381
C  -0.98943  3.21448 -1.95291
H  -5.54751  1.18494 -0.15132
H  -3.08402  1.36199 -0.08684
H  -2.85511 -2.80142  1.06753
H  -5.29679 -2.97106  0.99743
H   3.73053  0.75131 -3.62636
```

```
H   2.41798 -0.65094 -2.07461
H   2.57714  2.50722  0.89241
H   3.88285  3.89982 -0.67617
H   4.47478  3.03081 -2.93771
H   3.95334  0.41383  1.99856
H   4.52252  0.85828  4.36135
H   2.73000  0.94385  6.09377
H   0.35181  0.57633  5.43792
H  -0.23510  0.12745  3.07457
H  -0.67025  1.10559 -2.22976
H  -1.09140  3.39358 -3.02318
H  -1.04994  4.82092  1.05034
H  -0.62602  2.50507  1.85356
Si  1.97979 -2.49295  0.40083
C   1.08488 -3.41698  1.77338
H   0.01644 -3.57743  1.56789
H   1.55156 -4.41318  1.85858
H   1.18612 -2.91400  2.74747
C   3.83576 -2.49610  0.69442
H   4.07520 -2.17931  1.72047
H   4.20696 -3.52776  0.57124
H   4.38493 -1.85136 -0.00807
H  -0.92218 -1.70412  0.89482
C   1.52792 -3.10880 -1.31597
C   0.18867 -3.32735 -1.70134
C   2.54208 -3.42083 -2.24644
C  -0.12510 -3.83070 -2.96572
C   2.22999 -3.92813 -3.51096
C   0.89552 -4.13175 -3.87327
H  -0.63014 -3.10700 -1.01223
H   3.59397 -3.27623 -1.98663
H  -1.16970 -3.99246 -3.24252
H   3.03194 -4.16990 -4.21273
H   0.65048 -4.52944 -4.86114
O  -6.93069 -0.87116  0.34335
C  -7.67721 -2.04057  0.63467
H  -7.43163 -2.86047 -0.06187
H  -7.50844 -2.37950  1.67115
H  -8.73342 -1.77010  0.51279
O  -1.32658  5.55535 -1.35670
C  -1.49619  5.91824 -2.71395
H  -0.59351  5.69791 -3.31040
H  -2.36243  5.40519 -3.16704
H  -1.67617  7.00071 -2.72498
```

TS(III-IV)$_{H,OMe}$

```
SCF (TPSS/def2-SVP) Energy = -2366.70933403
Enthalpy 0K = -2365.968174
Enthalpy 298K = -2365.967230
Free Energy 298K = -2366.105498
Lowest Frequency = -7.2840 cm-1
Second Frequency = 6.2809 cm-1
SCF (M052X-D3,MeCN/def2-TZVP) Energy = -
2368.32558187
```

```
P  -0.02924 -0.68953  0.22753
C   2.67171  2.48480 -0.05252
C  -0.06782 -1.42873  1.92961
C   1.64460  0.12184  0.13933
C   2.87747 -0.71132  0.11337
C   1.60221  1.48370 -0.00116
C   0.10602 -2.15319 -0.89629
C   4.03434  2.24412  0.27734
C   2.32323  3.80461 -0.42733
C  -0.59864 -2.71143  2.18848
C  -0.67998 -2.12278 -2.06849
C   0.95650 -3.26005 -0.67768
C   4.98040  3.26030  0.21601
C   4.61195  4.56645 -0.18336
C   0.29082 -0.61526  3.02930
C   3.26509  4.83561 -0.50431
C   3.36419 -1.38960  1.25893
C   5.21365 -2.32680 -0.02070
C   3.58663 -0.87595 -1.09628
C   4.51373 -2.17695  1.19753
C   1.01289 -4.30572 -1.60954
C  -0.74769 -3.17150  3.50693
C  -0.37507 -2.36063  4.58834
C   0.22682 -4.26276 -2.77202
C   0.14480 -1.07912  4.34352
C  -0.61993 -3.16887 -3.00128
C   4.73879 -1.66936 -1.17426
H   6.02653  3.07552  0.47776
H   4.34875  1.24885  0.59873
H   1.27801  4.02724 -0.67377
H   2.94654  5.83625 -0.80750
H  -1.23659 -3.13005 -3.90603
H  -1.34337 -1.26875 -2.24910
H   1.57745 -3.30487  0.22239
H   1.67763 -5.15733 -1.42819
H   0.27474 -5.08235 -3.49726
H  -0.88594 -3.36307  1.35611
H  -1.14883 -4.17528  3.68632
H  -0.48390 -2.72516  5.61527
H   0.44355 -0.43879  5.18101
H   0.70216  0.38586  2.85114
H   3.22582 -0.37142 -1.99874
H   5.25447 -1.77036 -2.13282
H   4.89916 -2.68806  2.08498
H   2.84096 -1.28394  2.21382
Si -4.27404  0.43555  0.67825
C  -3.06161  1.75876  1.18958
H  -3.20747  2.72067  0.66832
H  -3.09920  1.93238  2.27926
H  -2.05017  1.37879  0.93902
C  -4.20183 -1.13334  1.67629
H  -4.98587 -1.85150  1.37964
H  -3.21835 -1.61491  1.52724
H  -4.31108 -0.91974  2.75419
H   0.59542  1.92066 -0.08560
C  -4.48716  0.21246 -1.16645
C  -3.72024  0.96517 -2.08954
C  -5.40440 -0.74002 -1.67583
C  -3.86889  0.77520 -3.47097
C  -5.55671 -0.92530 -3.05626
C  -4.78918 -0.16718 -3.95471
H  -2.98613  1.69555 -1.73048
H  -6.00422 -1.35233 -0.99122
H  -3.26249  1.35993 -4.17062
H  -6.26867 -1.66723 -3.43276
H  -4.90504 -0.31549 -5.03355
O   5.61272  5.48092 -0.21709
C   5.31500  6.82034 -0.60567
H   4.91601  6.86001 -1.63680
H   4.59075  7.28808  0.08768
H   6.26879  7.36528 -0.56087
O   6.31915 -3.11627  0.02131
C   7.07995 -3.30070 -1.16980
H   6.47683 -3.78222 -1.96292
H   7.47884 -2.33952 -1.54578
H   7.91519 -3.96062 -0.89431
C  -6.71672  2.00163  0.94248
O  -5.90854  1.07421  1.26577
C  -7.98968  2.09635  1.71013
C  -6.40388  2.97445 -0.14517
H  -8.00106  1.39452  2.55557
H  -7.30188  3.51560 -0.47811
H  -8.83403  1.87380  1.02719
H  -8.14663  3.13453  2.05740
H  -5.91138  2.46977 -0.99459
H  -5.68484  3.71863  0.25422
```

TS(II-III)$_{O,CF3}$

```
SCF (TPSS/def2-SVP) Energy = -2209.96767465
Enthalpy 0K = -2209.467603
Enthalpy 298K = -2209.466659
Free Energy 298K = -2209.581339
Lowest Frequency = -89.7921 cm-1
Second Frequency = 12.4141 cm-1
SCF (M052X-D3,MeCN/def2-TZVP) Energy = -
2211.75737839
```

P   0.24448 1.66532 1.17546
C -1.47290 -0.96481 -0.37535
C -1.45031 1.35224 1.81115
C   0.86504 0.08165 0.30066
C   2.33670 -0.03846 0.16718
C -0.10302 -0.71717 -0.08167
C   0.04349 2.86329 -0.19832
C -2.04202 -0.40090 -1.54926
C -2.28730 -1.74016 0.49251
C -1.53986 0.76809 3.09590
C   0.50073 2.63159 -1.51316
C -0.47926 4.13593 0.13455
C -3.39993 -0.57407 -1.82002
C -4.20026 -1.31566 -0.93388
C -2.63323 1.73365 1.14116
C -3.64127 -1.91029 0.21319
C   2.89965 -0.58512 -1.01042
C   5.13124 -0.25752 -0.10965
C   3.19536 0.42004 1.18916
C   4.28602 -0.69085 -1.14662
C -0.56522 5.14004 -0.83740
C -2.79107 0.54545 3.68604
C -3.96245 0.92012 3.00876
C -0.11785 4.89766 -2.14581
C -3.88187 1.51576 1.73983
C   0.41705 3.64507 -2.47913
C   4.58357 0.30295 1.05466
H -3.84364 -0.13636 -2.71874
H -1.41233 0.17958 -2.22888
H -1.85338 -2.18380 1.39272
H -4.27496 -2.49663 0.88427
H   0.77400 3.45329 -3.49644
H   0.92867 1.66229 -1.78824
H -0.81941 4.34370 1.15561
H -0.97699 6.11826 -0.56864
H -0.18112 5.68649 -2.90221
H -0.62515 0.50575 3.64112
H -2.85133 0.09938 4.68432
H -4.93969 0.75975 3.47615
H -4.79600 1.80748 1.21248
H -2.57634 2.20694 0.15587
H   2.77119 0.86970 2.09274
H   5.24377 0.65734 1.85136
H   4.71762 -1.09573 -2.06686
H   2.24517 -0.91121 -1.82385
C   1.16413 -3.42690 0.11199
O   0.65710 -2.57100 -0.63262
C   1.52374 -4.76525 -0.47800
C   1.45977 -3.18062 1.56614
H   1.39233 -4.75896 -1.56911
H   0.85539 -2.35514 1.96943
H   0.87249 -5.53916 -0.02949
H   2.56257 -5.03028 -0.21141
H   2.52726 -2.89684 1.64648
H   1.32230 -4.09789 2.16154
C   6.63136 -0.43427 -0.23932
F   7.30664 0.45935 0.51319
F   7.00339 -1.67310 0.16906
F   7.03629 -0.29599 -1.52081
C -5.69508 -1.41893 -1.17757
F -5.99107 -1.35249 -2.49081
F -6.19906 -2.57003 -0.68756
F -6.33798 -0.39605 -0.56029

$III_{O,CF3}$

SCF (TPSS/def2-SVP) Energy = -2209.98040065
Enthalpy 0K = -2209.478769
Enthalpy 298K = -2209.477825
Free Energy 298K = -2209.589611
Lowest Frequency = 13.8732 cm-1
Second Frequency = 14.7109 cm-1
SCF (M052X-D3,MeCN/def2-TZVP) Energy = -
2211.78763103

P   0.20556 1.38490 1.17705
C -1.66679 -1.23592 -0.26834
C -1.60029 1.59507 1.37260
C   0.63742 -0.20947 0.36699
C   2.11246 -0.36354 0.20324
C -0.23738 -1.22789 0.05711
C   0.76728 2.68232 0.00989
C -2.23456 -0.18932 -1.03148
C -2.49890 -2.30195 0.14932
C -2.27448 0.78733 2.31689
C   0.88888 2.48528 -1.38255
C   1.09829 3.93980 0.56213
C -3.59880 -0.18585 -1.32522
C -4.42012 -1.22961 -0.86659
C -2.31093 2.61795 0.70733
C -3.86466 -2.29745 -0.14319
C   2.63261 -0.92473 -0.99022
C   4.90533 -0.59550 -0.19749
C   3.02794 0.09617 1.18058
C   4.01086 -1.03883 -1.18815
C   1.52310 4.98536 -0.26838
C -3.64460 0.95989 2.54323
C -4.35094 1.96374 1.85983
C   1.64124 4.78155 -1.65181
C -3.67861 2.79942 0.95578
C   1.32764 3.53114 -2.20633
C   4.40940 -0.01826 0.98102
H -4.02940 0.62457 -1.91985
H -1.59537 0.61421 -1.40624
H -2.08281 -3.13551 0.72402
H -4.50403 -3.11901 0.19154
H   1.42150 3.36971 -3.28534
H   0.64555 1.51325 -1.82523
H   1.02398 4.09974 1.64388
H   1.77293 5.95794 0.16790
H   1.98190 5.59678 -2.29838
H -1.72513 0.02212 2.87809
H -4.16191 0.32055 3.26606
H -5.42164 2.10013 2.04216
H -4.22171 3.59582 0.43622
H -1.79537 3.26897 -0.00607
H   2.64958 0.54413 2.10535
H   5.10457 0.34691 1.74257
H   4.39796 -1.45215 -2.12448
H   1.94692 -1.24308 -1.78267
C   0.99547 -3.25008 0.71097
O   0.29468 -2.56876 -0.13252
C   1.51035 -4.54199 0.18872
C   1.26702 -2.79713 2.09507
H   1.15478 -4.73063 -0.83389
H   0.49951 -2.08875 2.44649
H   1.21572 -5.36689 0.86310
H   2.61813 -4.51452 0.19948
H   2.23874 -2.26096 2.11644
H   1.35411 -3.66747 2.76533
C   6.39688 -0.78163 -0.39106
F   7.10898 0.11526 0.32215
F   6.77979 -2.01902 0.01384
F   6.74559 -0.65943 -1.69024
C -5.91392 -1.16842 -1.11249
F -6.19706 -0.61197 -2.31029
F -6.47947 -2.39369 -1.07481
F -6.52144 -0.41160 -0.16360

$TS(II\text{-}III)_{O,OMe}$

SCF (TPSS/def2-SVP) Energy = -1765.27311482
Enthalpy 0K = -1764.719735
Enthalpy 298K = -1764.718791
Free Energy 298K = -1764.824410
Lowest Frequency = -168.3650 cm-1
Second Frequency = 10.3095 cm-1
SCF (M052X-D3,MeCN/def2-TZVP) Energy = -
1766.54407807

P   0.24986 1.48659 1.22874
C -1.40773 -1.09440 -0.56938
C -1.53813 1.24976 1.60360
C   0.85052 -0.06473 0.33552

```
C   2.32363 -0.19552  0.26366
C  -0.07416 -0.91479 -0.10331
C   0.29316  2.77782 -0.08198
C  -1.83281 -0.43441 -1.76127
C  -2.33643 -1.93520  0.09949
C  -1.84465  0.59120  2.81589
C   0.71335  2.55149 -1.40858
C  -0.03548  4.09383  0.31792
C  -3.12268 -0.59577 -2.24196
C  -4.03905 -1.43327 -1.55742
C  -2.58868  1.77179  0.82105
C  -3.63214 -2.10625 -0.37794
C   2.94372 -0.71393 -0.89720
C   5.15094 -0.39535  0.07632
C   3.15904  0.23760  1.32599
C   4.33461 -0.81543 -0.99871
C   0.02706  5.14999 -0.59944
C  -3.17631  0.43145  3.22162
C  -4.21562  0.95157  2.43416
C   0.44046  4.91421 -1.92024
C  -3.91922  1.62457  1.23879
C   0.78765  3.61604 -2.31978
C   4.54413  0.13030  1.24054
H  -3.46043 -0.09092 -3.15143
H  -1.12911  0.21043 -2.29497
H  -2.03562 -2.44868  1.01794
H  -4.32636 -2.75067  0.16633
H   1.11845  3.42760 -3.34677
H   0.99381  1.54412 -1.73286
H  -0.34277  4.29322  1.35133
H  -0.23773  6.16277 -0.27810
H   0.49778  5.74267 -2.63378
H  -1.03246  0.22204  3.45431
H  -3.40208 -0.07542  4.16599
H  -5.25555  0.84788  2.76193
H  -4.72758  2.04319  0.62969
H  -2.36492  2.30342 -0.10934
H   2.70580  0.65982  2.22908
H   5.18928  0.45726  2.06112
H   4.77594 -1.20155 -1.92104
H   2.32502 -1.02446 -1.74521
C   1.12310 -3.41132  0.37517
O   0.69902 -2.60979 -0.48382
C   1.53555 -4.77817 -0.09194
C   1.26712 -3.05711  1.82475
H   1.46741 -4.85749 -1.18587
H   0.47466 -2.36271  2.14530
H   0.88277 -5.53440  0.38348
H   2.56511 -4.99057  0.24959
H   2.23095 -2.52191  1.94041
H   1.29169 -3.95426  2.46167
O   6.50047 -0.44773  0.08390
C   7.19180 -0.93819 -1.06761
H   6.92238 -1.99007 -1.27715
H   6.97858 -0.31496 -1.95547
H   8.26105 -0.87494 -0.82220
O  -5.26342 -1.52653 -2.10181
C  -6.26047 -2.35407 -1.48710
H  -5.93470 -3.40930 -1.46023
H  -6.48834 -2.00240 -0.46489
H  -7.15340 -2.25828 -2.11964
```

$III_{O,OMe}$

```
SCF (TPSS/def2-SVP) Energy = -1765.28102164
Enthalpy 0K = -1764.726313
Enthalpy 298K = -1764.725369
Free Energy 298K = -1764.829096
Lowest Frequency = 10.9662 cm-1
Second Frequency = 20.1155 cm-1
SCF (M052X-D3,MeCN/def2-TZVP) Energy = -
1766.56061537

P   0.23488  1.33849  1.13694
C  -1.56633 -1.26385 -0.45439
C  -1.57621  1.47196  1.38143
C   0.69589 -0.26247  0.36372
```

```
C   2.16719 -0.42984  0.25806
C  -0.18414 -1.26895  0.01036
C   0.69987  2.63357 -0.07883
C  -2.06970 -0.19405 -1.24260
C  -2.43247 -2.35241 -0.19047
C  -2.17647  0.64921  2.36126
C   0.93665  2.38034 -1.44649
C   0.85953  3.94780  0.41552
C  -3.37157 -0.20842 -1.72248
C  -4.22890 -1.29890 -1.43909
C  -2.35549  2.46276  0.74740
C  -3.74416 -2.37940 -0.66846
C   2.73506 -1.05255 -0.88117
C   4.99157 -0.67124 -0.04993
C   3.06488  0.07863  1.23805
C   4.11832 -1.17667 -1.04133
C   1.21947  4.99031 -0.44839
C  -3.53530  0.78584  2.66704
C  -4.30565  1.76930  2.02524
C   1.44913  4.73021 -1.80838
C  -3.71103  2.61029  1.07364
C   1.31248  3.42477 -2.30349
C   4.44432 -0.04203  1.09126
H  -3.75864  0.60982 -2.33603
H  -1.41366  0.64394 -1.49372
H  -2.08181 -3.19899  0.40873
H  -4.38146 -3.23658 -0.43856
H   1.49524  3.21737 -3.36324
H   0.83519  1.36460 -1.84304
H   0.70160  4.15431  1.48043
H   1.33325  6.00604 -0.05554
H   1.73985  5.54399 -2.48080
H  -1.57407 -0.09472  2.89639
H  -3.99087  0.13768  3.42318
H  -5.36457  1.88759  2.27756
H  -4.30501  3.38582  0.57860
H  -1.90109  3.12185  0.00057
H   2.66157  0.57078  2.12951
H   5.12965  0.34941  1.84868
H   4.51067 -1.63928 -1.95068
H   2.07807 -1.41632 -1.67898
C   0.94109 -3.29661  0.82736
O   0.33509 -2.62700 -0.09737
C   1.39820 -4.64989  0.41282
C   1.18915 -2.76127  2.18369
H   1.05258 -4.89231 -0.60212
H   0.40161 -2.04581  2.47589
H   1.04792 -5.40897  1.13575
H   2.50555 -4.67926  0.44186
H   2.14169 -2.18792  2.17711
H   1.28569 -3.58189  2.91149
O   6.33899 -0.73495 -0.10556
C   6.97007 -1.33314 -1.24114
H   6.68441 -2.39663 -1.34234
H   6.71707 -0.78885 -2.16945
H   8.05036 -1.25924 -1.05432
O  -5.47352 -1.21848 -1.95312
C  -6.39887 -2.28632 -1.72894
H  -6.02369 -3.23364 -2.15768
H  -6.59999 -2.41787 -0.64988
H  -7.32413 -1.99068 -2.24263
```

$TS(II\text{-}III)_{P,CF3}$

```
SCF (TPSS/def2-SVP) Energy = -2209.97029448
Enthalpy 0K = -2209.470108
Enthalpy 298K = -2209.469164
Free Energy 298K = -2209.588008
Lowest Frequency = -97.0658 cm-1
Second Frequency = 8.5013 cm-1
SCF (M052X-D3,MeCN/def2-TZVP) Energy = -
2211.76073996

P   0.53906  1.44486  0.09113
C  -1.78922 -1.48452 -0.19476
C  -0.48946  1.43639  1.60059
C   0.82290 -0.95339 -0.19004
```

```
C   2.23336 -1.27800 -0.16814
C  -0.39879 -1.21522 -0.19949
C  -0.54367  1.84898 -1.33369
C  -2.41372 -1.94316  0.99789
C  -2.56722 -1.33784 -1.37489
C   0.23799  1.42857  2.81520
C  -0.22850  1.26201 -2.58254
C  -1.55302  2.83894 -1.27876
C  -3.77597 -2.24145  1.00473
C  -4.53787 -2.07669 -0.16615
C  -1.90135  1.41775  1.64254
C  -3.93131 -1.62938 -1.35363
C   2.66774 -2.36432  0.62912
C   4.94973 -2.01882 -0.12458
C   3.18306 -0.56419 -0.93395
C   4.01691 -2.72831  0.64930
C  -2.24716  3.20393 -2.44046
C  -0.43233  1.42839  4.04360
C  -1.83640  1.42762  4.07469
C  -1.94349  2.59693 -3.66838
C  -2.56658  1.41709  2.87629
C  -0.92977  1.62802 -3.73837
C   4.52905 -0.93716 -0.91706
H  -4.25416 -2.60878  1.91728
H  -1.81645 -2.06650  1.90538
H  -2.08951 -1.00068 -2.29824
H  -4.53015 -1.52450 -2.26276
H  -0.67915  1.16118 -4.69659
H   0.56182  0.50600 -2.65016
H  -1.77704  3.34437 -0.33534
H  -3.02618  3.97143 -2.38530
H  -2.48904  2.88576 -4.57270
H   1.33450  1.42488  2.79498
H   0.13911  1.42852  4.97745
H  -2.36196  1.43029  5.03532
H  -3.66122  1.40564  2.90054
H  -2.48275  1.39514  0.71668
H   2.86012  0.28103 -1.54915
H   5.26038 -0.38539 -1.51477
H   4.34938 -3.56342  1.27252
H   1.94090 -2.91817  1.23001
C   1.79466  4.32434  0.30112
O   0.76010  3.64153  0.41544
C   3.09478  3.75670 -0.19785
C   1.73254  5.78336  0.66002
H   3.13131  2.66477 -0.06477
H   0.69472  6.10001  0.83481
H   3.95555  4.24372  0.28855
H   3.16561  3.98086 -1.28045
H   2.20173  6.39407 -0.13159
H   2.32753  5.94489  1.57940
C   6.40506 -2.44166 -0.14087
F   6.65032 -3.30566 -1.15283
F   7.22387 -1.37747 -0.31348
F   6.75737 -3.05450  1.01045
C  -6.03167 -2.33905 -0.13417
F  -6.34665 -3.30126  0.75858
F  -6.70334 -1.21722  0.22457
F  -6.49530 -2.71895 -1.34345
```

TS(II-III)$_{P,OMe}$

```
SCF (TPSS/def2-SVP) Energy = -1765.27204670
Enthalpy 0K = -1764.718693
Enthalpy 298K = -1764.717749
Free Energy 298K = -1764.829024
Lowest Frequency = -13.9848 cm-1
Second Frequency = 6.5335 cm-1
SCF (M052X-D3,MeCN/def2-TZVP) Energy = -
1766.53887893

P   0.60673  1.05334  0.11596
C  -1.89309 -1.75720 -0.05678
C  -0.32122  1.17800  1.68011
C   0.75722 -1.55051 -0.07493
C   2.15921 -1.83337 -0.05037
C  -0.48766 -1.59624 -0.08131
```

```
C  -0.55153  1.51796 -1.22861
C  -2.56478 -2.02121  1.17439
C  -2.66447 -1.71117 -1.24783
C   0.47880  1.21957  2.84868
C  -0.35772  0.90243 -2.48821
C  -1.50079  2.56162 -1.11802
C  -3.93580 -2.22972  1.20281
C  -4.69360 -2.17990  0.00653
C  -1.72854  1.18506  1.81066
C  -4.04389 -1.91692 -1.22218
C   2.82153 -2.06673  1.18593
C   4.92710 -2.43359  0.02096
C   2.91841 -1.90106 -1.24218
C   4.17903 -2.36150  1.22012
C  -2.25187  2.95449 -2.23399
C  -0.11396  1.29737  4.11344
C  -1.51360  1.32000  4.23043
C  -2.06803  2.31908 -3.47184
C  -2.31615  1.25797  3.08102
C  -1.11704  1.29410 -3.59817
C   4.28378 -2.19961 -1.21502
H  -4.45891 -2.44484  2.13902
H  -1.98497 -2.07024  2.10026
H  -2.16650 -1.51956 -2.20215
H  -4.60721 -1.88136 -2.15776
H  -0.96136  0.80406 -4.56511
H   0.38744  0.10609 -2.59394
H  -1.63277  3.08467 -0.16622
H  -2.98126  3.76559 -2.13670
H  -2.65816  2.62966 -4.34043
H   1.57157  1.19784  2.76000
H   0.51378  1.33933  5.00956
H  -1.97867  1.38049  5.22000
H  -3.40729  1.26279  3.17276
H  -2.36557  1.12184  0.92431
H   2.42637 -1.73480 -2.20581
H   4.83688 -2.25867 -2.15569
H   4.69458 -2.55417  2.16546
H   2.24976 -2.02478  2.11799
C   2.05952  3.82215 -0.14770
O   1.09113  3.23981  0.37244
C   2.94045  3.15338 -1.16891
C   2.33847  5.24777  0.24022
H   3.08622  2.09101 -0.91009
H   1.56160  5.62876  0.91791
H   3.90662  3.66773 -1.28322
H   2.41434  3.18525 -2.14280
H   2.40583  5.87647 -0.66646
H   3.32798  5.29952  0.73276
O   6.23791 -2.73237  0.16122
C   7.05627 -2.86192 -1.00336
H   6.68570 -3.66801 -1.66308
H   7.10269 -1.91157 -1.56717
H   8.05930 -3.11970 -0.63563
O  -6.01725 -2.39954  0.14162
C  -6.85648 -2.39641 -1.01768
H  -6.83978 -1.41064 -1.51772
H  -6.55026 -3.18323 -1.73084
H  -7.87056 -2.60610 -0.65027
```

HP1

```
SCF (TPSS/def2-SVP) Energy = -1344.08499993
Enthalpy 0K = -1343.682050
Enthalpy 298K = -1343.681106
Free Energy 298K = -1343.763374
Lowest Frequency = 13.6693 cm-1
Second Frequency = 19.4359 cm-1
SCF (M052X-D3,MeCN/def2-TZVP) Energy = -
1344.95802700

P   0.86771 -0.85152  0.15109
C  -2.10038 -1.56444  0.14652
C   1.74070 -0.20265  1.64407
C  -0.50945  0.56181 -0.14804
C  -1.24042  1.82136 -0.31480
C  -0.88994 -0.73387  0.07798
```

```
C   1.84697 -0.71118 -1.40383
C  -3.35714 -1.06337 -0.26956
C  -2.02632 -2.89564  0.61792
C   2.64486 -1.04389  2.32141
C   2.28343 -1.87651 -2.06095
C   2.17548  0.55025 -1.93457
C  -4.49923 -1.86932 -0.20734
C  -4.41348 -3.18827  0.26891
C   1.54475  1.11285  2.10240
C  -3.17273 -3.69796  0.68043
C  -2.12466  2.28453  0.69476
C  -2.57002  4.31974 -0.56421
C  -1.03703  2.64755 -1.44870
C  -2.76978  3.52151  0.57325
C   2.93078  0.64350 -3.11149
C   3.34108 -0.57626  3.44508
C   3.13743  0.73487  3.90095
C   3.34529 -0.52219 -3.77618
C   2.23787  1.57724  3.22897
C   3.01762 -1.78178 -3.25275
C  -1.70609  3.87088 -1.57592
H  -5.31015 -3.81588  0.31635
H  -5.46474 -1.46732 -0.53440
H  -3.42772 -0.03817 -0.64604
H  -1.05951 -3.29698  0.94021
H  -3.09750 -4.72619  1.05148
H   3.33729 -2.69266 -3.77099
H   2.04334 -2.85949 -1.63840
H   1.83736  1.45386 -1.41578
H   3.19670  1.62772 -3.51287
H   3.92709 -0.44784 -4.70162
H   2.79928 -2.06962  1.96594
H   4.04128 -1.23782  3.96712
H   3.67944  1.10040  4.78023
H   2.07618  2.60154  3.58235
H   0.84401  1.76578  1.57163
H  -0.36049  2.30909 -2.24049
H  -1.54584  4.48343 -2.47056
H  -3.08171  5.28342 -0.66074
H  -3.44140  3.86103  1.37026
H  -2.29266  1.66322  1.58139
H   1.30658 -2.23734  0.39260
```

HP2

```
SCF (TPSS/def2-SVP) Energy = -1344.08055805
Enthalpy 0K = -1343.677765
Enthalpy 298K = -1343.676820
Free Energy 298K = -1343.757790
Lowest Frequency = 21.3441 cm-1
Second Frequency = 22.9453 cm-1
SCF (M052X-D3,MeCN/def2-TZVP) Energy = -
1344.95456791
```

```
P   0.54220 -0.57060 -0.69861
C  -2.76329 -0.46654 -0.65669
C   2.34534  0.06455 -0.83759
C  -0.48845  0.73528 -0.07538
C  -0.52014  2.09608  0.48018
C  -1.32468 -0.28691 -0.46020
C   0.86509 -2.04173  0.37233
C  -3.64985  0.62652 -0.83820
C  -3.29483 -1.77787 -0.73363
C   2.96699  0.15285 -2.09556
C  -0.03711 -2.38476  1.39530
C   1.99609 -2.85140  0.14860
C  -5.01126  0.41140 -1.07739
C  -5.52411 -0.89611 -1.12690
C   3.04110  0.53760  0.29443
C  -4.66134 -1.98956 -0.95206
C  -1.51280  2.46895  1.41879
C  -0.55864  4.69942  1.59022
C   0.45845  3.05134  0.11796
C  -1.52900  3.75651  1.96723
C   2.20837 -3.99824  0.92809
C   4.25882  0.68991 -2.22837
C   4.94179  1.15024 -1.09336
C   1.31162 -4.32754  1.95551
C   4.33043  1.07334  0.17000
C   0.19223 -3.51489  2.19303
C   0.43268  4.34104  0.66401
H  -6.59198 -1.06096 -1.30821
H  -5.68008  1.26710 -1.22399
H  -3.25518  1.64715 -0.80323
H  -2.61464 -2.62807 -0.61402
H  -5.05511 -3.01110 -0.99747
H  -0.50754 -3.76405  2.99835
H  -0.92183 -1.76015  1.56334
H   2.71384 -2.58393 -0.63452
H   3.08179 -4.63137  0.73673
H   1.48541 -5.21654  2.57181
H   2.43246 -0.20436 -2.98792
H   4.73122  0.74968 -3.21580
H   5.94886  1.57102 -1.19098
H   4.86326  1.43267  1.05807
H   2.57111  0.48325  1.28515
H   1.23577  2.77615 -0.60169
H   1.19373  5.07058  0.36542
H  -0.57394  5.70714  2.01961
H  -2.30206  4.02661  2.69551
H  -2.26627  1.73412  1.72186
H   0.53720 -0.96270 -2.08104
```

11. References

(1) Yakelis, N. A.; Bergman, R. G., Safe Preparation and Purification of Sodium Tetrakis[(3,5-trifluoromethyl)phenyl]borate ($NaBArF_{24}$): Reliable and Sensitive Analysis of Water in Solutions of Fluorinated Tetraarylborates. *Organometallics* **2005**, *24*, 3579-3581.

(2) Mio, M. J.; Kopel, L. C.; Braun, J. B.; Gadzikwa, T. L.; Hull, K. L.; Brisbois, R. G.; Markworth, C. J.; Grieco, P. A., One-Pot Synthesis of Symmetrical and Unsymmetrical Bisarylethynes by a Modification of the Sonogashira Coupling Reaction. *Org. Lett.* **2002**, *4*, 3199-3202.

(3) Ushijima, S.; Dohi, S.; Moriyama, K.; Togo, H., Facile preparation of aromatic ketones from aromatic bromides and arenes with aldehydes. *Tetrahedron* **2012**, *68*, 1436-1442.

(4) Huang, W.; Byun, J.; Rörich, I.; Ramanan, C.; Blom, P. W. M.; Lu, H.; Wang, D.; Caire da Silva, L.; Li, R.; Wang, L.; Landfester, K.; Zhang, K. A. I., Asymmetric Covalent Triazine Framework for Enhanced Visible-Light Photoredox Catalysis via Energy Transfer Cascade. *Angew. Chem. Int. Ed.* **2018**, *57*, 8316-8320.

(5) Kortmann, F. A.; Chang, M.-C.; Otten, E.; Couzijn, E. P. A.; Lutz, M.; Minnaard, A. J., Consecutive dynamic resolutions of phosphine oxides. *Chem Sci.* **2014**, *5*, 1322-1327.

(6) Petit, C.; Favre-Réguillon, A.; Mignani, G.; Lemaire, M., A straightforward synthesis of unsymmetrical secondary phosphine boranes. *Green Chem.* **2010**, *12*, 326-330.

(7) Gudat, D., Cationic low coordinated phosphorus compounds as ligands: Recent developments. *Coord. Chem. Rev.* **1997**, *163*, 71-106.

(8) Ivanov, B. E.; Krokhina, S. S.; Chichkanova, T. V.; Ageeva, A. B., Dual reactivity of diphenylphosphinous and phenylphosphonous acid amides in reactions with N-acetoxymethyl-substituted diethylamine, benzamide, and acetamide. *Bull. Acad. Sci. USSR, Div. Chem. Sci.* **1986**, *35*, 2535-2539.

(9) Fluck, E.; Binder, H., Darstellung von verbindungen MIT P-P - und P-P-P - gerüsten. *Inorg. Nucl. Chem. Lett.* **1967**, *3*, 307-313.

(10) Dinh, L. V.; Gladysz, J. A., "Catalyst-on-a-Tape"—Teflon: A New Delivery and Recovery Method for Homogeneous Fluorous Catalysts. *Angew. Chem. Int. Ed.* **2005**, *44*, 4095-4097.

(11) Yabushita, K.; Yuasa, A.; Nagao, K.; Ohmiya, H., Asymmetric Catalysis Using Aromatic Aldehydes as Chiral α-Alkoxyalkyl Anions. *J. Am. Chem. Soc.* **2019**, *141*, 113-117.

(12) Takeda, M.; Yabushita, K.; Yasuda, S.; Ohmiya, H., Synergistic palladium/copper-catalyzed Csp^3–Csp^2 cross-couplings using aldehydes as latent α-alkoxyalkyl anion equivalents. *Chem. Commun.* **2018**, *54*, 6776-6779.

(13) Garcés, K.; Lalrempuia, R.; Polo, V.; Fernández-Alvarez, F. J.; García-Orduña, P.; Lahoz, F. J.; Pérez-Torrente, J. J.; Oro, L. A., Rhodium-Catalyzed Dehydrogenative Silylation of Acetophenone Derivatives: Formation of Silyl Enol Ethers versus Silyl Ethers. *Chem. Eur. J.* **2016**, *22*, 14717-14729.

(14) Ye, F.; Zheng, Z.-J.; Li, L.; Yang, K.-F.; Xia, C.-G.; Xu, L.-W., Development of a Novel Multifunctional N,P Ligand for Highly Enantioselective Palladium-Catalyzed Asymmetric Allylic Etherification of Alcohols and Silanols. *Chem. Eur. J.* **2013**, *19*, 15452-15457.

(15) Mitsudome, T.; Yamamoto, Y.; Noujima, A.; Mizugaki, T.; Jitsukawa, K.; Kaneda, K., Highly Efficient Etherification of Silanes by Using a Gold Nanoparticle Catalyst: Remarkable Effect of O_2. *Chem. Eur. J.* **2013**, *19*, 14398-14402.

(16) Mohr, J.; Durmaz, M.; Irran, E.; Oestreich, M., Tris(5,6,7,8-tetrafluoronaphthalen-2-yl)borane, a Partially Fluorinated Boron Lewis Acid with Fluorination Distal to the Boron Atom. *Organometallics* **2014**, *33*, 1108-1111.

(17) Rawat, S.; Bhandari, M.; Porwal, V. K.; Singh, S., Hydrosilylation of Carbonyls Catalyzed by Hydridoborenium Borate Salts: Lewis Acid Activation and Anion Mediated Pathways. *Inorg. Chem.* **2020**, *59*, 7195-7203.

(18) Toutov, A. A.; Betz, K. N.; Haibach, M. C.; Romine, A. M.; Grubbs, R. H., Sodium Hydroxide Catalyzed Dehydrocoupling of Alcohols with Hydrosilanes. *Org. Lett.* **2016**, *18*, 5776-5779.
(19) Fernandes, A. C.; Fernandes, R.; Romão, C. C.; Royo, B., [MoO_2Cl_2] as catalyst for hydrosilylation of aldehydes and ketones. *Chem. Commun.* **2005**, 213-214.
(20) Reis, P. M.; Romão, C. C.; Royo, B., Dioxomolybdenum(vi) complexes as catalysts for the hydrosilylation of aldehydes and ketones. *Dalton Trans.* **2006**, 1842-1846.
(21) Fujita, M.; Hiyama, T., Fluoride ion-catalyzed reduction of aldehydes and ketones with hydrosilanes. Synthetic and mechanistic aspects and an application to the threo-directed reduction of .alpha.-substituted alkanones. *J. Org. Chem.* **1988**, *53*, 5405-5415.
(22) Vasilikogiannaki, E.; Titilas, I.; Gryparis, C.; Louka, A.; Lykakis, I. N.; Stratakis, M., Efficient hydrosilylation of carbonyl compounds by 1,1,3,3-tetramethyldisiloxane catalyzed by Au/TiO_2. *Tetrahedron* **2014**, *70*, 6106-6113.
(23) Hudrlik, P. F.; Hudrlik, A. M.; Jeilani, Y. A., Intramolecular nucleophilic attack at silicon in o-silylbenzyl alcohols. Generation of allyl and benzyl anion equivalents. *Tetrahedron* **2011**, *67*, 10089-10096.
(24) Petitjean, L.; Gagne, R.; Beach, E. S.; Xiao, D.; Anastas, P. T., Highly selective hydrogenation and hydrogenolysis using a copper-doped porous metal oxide catalyst. *Green Chem.* **2016**, *18*, 150-156.
(25) Unoh, Y.; Hirano, K.; Miura, M., Metal-Free Electrophilic Phosphination/Cyclization of Alkynes. *J. Am. Chem. Soc.* **2017**, *139*, 6106-6109.
(26) Laali, K. K.; Geissler, B.; Wagner, O.; Hoffmann, J.; Armbrust, R.; Eisfeld, W.; Regitz, M., Generation of the First Persistent Phosphirenylium Cation. *J. Am. Chem. Soc.* **1994**, *116*, 9407-9408.
(27) Krishnamurti, V.; Barrett, C.; Prakash, G. K. S., Siladifluoromethylation and Deoxo-trifluoromethylation of P^V–H Compounds with $TMSCF_3$: Route to P^V–CF_2^- Transfer Reagents and P–CF_3 Compounds. *Org. Lett.* **2019**, *21*, 1526-1529.
(28) Pearce, K. G.; Borys, A. M.; Clark, E. R.; Shepherd, H. J., Exploring the Reactivity of Donor-Stabilized Phosphenium Cations: Lewis Acid-Catalyzed Reduction of Chlorophosphanes by Silanes. *Inorg. Chem.* **2018**, *57*, 11530-11536.
(29) Li, D. R.; Murugan, A.; Falck, J. R., Enantioselective, Organocatalytic Oxy-Michael Addition to γ/δ-Hydroxy-α,β-enones: Boronate-Amine Complexes as Chiral Hydroxide Synthons. *J. Am. Chem. Soc.* **2008**, *130*, 46-48.
(30) Bassindale, A. R.; Stout, T., The synthesis of functionalised silyltriflates. *J. Organomet. Chem.* **1984**, *271*, C1-C3.
(31) Barrett, A. N.; Sanderson, H. J.; Mahon, M. F.; Webster, R. L., Hydrophosphination using [$GeCl\{N(SiMe_3)_2\}_3$] as a pre-catalyst. *Chem. Commun.* **2020**, *56*, 13623-13626.
(32) Qian, Y.; Dai, Q.; Li, Z.; Liu, Y.; Zhang, J., O-Phosphination of Aldehydes/Ketones toward Phosphoric Esters: Experimental and Mechanistic Studies. *Org. Lett.* **2020**, *22*, 4742-4748.
(33) Dolomanov, O. V.; Bourhis, L. J.; Gildea, R. J.; Howard, J. A. K.; Puschmann, H., OLEX2: a complete structure solution, refinement and analysis program. *J. Appl. Crystallogr.* **2009**, *42*, 339-341.
(34) Bourhis, L. J.; Dolomanov, O. V.; Gildea, R. J.; Howard, J. A. K.; Puschmann, H., The anatomy of a comprehensive constrained, restrained refinement program for the modern computing environment - Olex2 dissected. *Acta Cryst. A* **2015**, *71*, 59-75.
(35) Sheldrick, G., Crystal structure refinement with SHELXL. *Acta Crystallogr., Sect. C: Cryst. Struct. Commun.* **2015**, *71*, 3-8.
(36) Sheldrick, G., SHELXT - Integrated space-group and crystal-structure determination. *Acta Crystallographica Section A* **2015**, *71*, 3-8.
(37) Gaussian 09, Revision D.01, M. J. Frisch, G. W. Trucks, H. B. Schlegel, G. E. Scuseria, M. A. Robb, J. R. Cheeseman, G. Scalmani, V. Barone, B. Mennucci, G. A. Petersson, H. Nakatsuji, M. Caricato, X. Li, H. P. Hratchian, A. F. Izmaylov, J. Bloino, G. Zheng, J. L. Sonnenberg, M. Hada, M. Ehara, K. Toyota, R. Fukuda, J. Hasegawa, M. Ishida, T. Nakajima, Y. Honda, O. Kitao, H. Nakai, T. Vreven, J. A. Montgomery, Jr., J. E. Peralta, F. Ogliaro, M. Bearpark, J. J. Heyd, E. Brothers, K. N. Kudin, V. N.

Staroverov, T. Keith, R. Kobayashi, J. Normand, K. Raghavachari, A. Rendell, J. C. Burant, S. S. Iyengar, J. Tomasi, M. Cossi, N. Rega, J. M. Millam, M. Klene, J. E. Knox, J. B. Cross, V. Bakken, C. Adamo, J. Jaramillo, R. Gomperts, R. E. Stratmann, O. Yazyev, A. J. Austin, R. Cammi, C. Pomelli, J. W. Ochterski, R. L. Martin, K. Morokuma, V. G. Zakrzewski, G. A. Voth, P. Salvador, J. J. Dannenberg, S. Dapprich, A. D. Daniels, O. Farkas, J. B. Foresman, J. V. Ortiz, J. Cioslowski, and D. J. Fox, Gaussian, Inc., Wallingford CT, **2013**.

(38) Tao, J.; Perdew, J. P.; Staroverov, V. N.; Scuseria, G. E., Climbing the Density Functional Ladder: Nonempirical Meta--Generalized Gradient Approximation Designed for Molecules and Solids. Phy*s. Rev. Lett.* **2003**, *91*, *1*46401.

(39) Weigend, F.; Ahlrichs, R., Balanced basis sets of split valence, triple zeta valence and quadruple zeta valence quality for H to Rn: Design and assessment of accuracy. PCC*P 200***5, 7**, *3*297-3305.

(40) Weigend, F., Accurate Coulomb-fitting basis sets for H to Rn. PCC*P* **2006**, *8*, *1*057-1065.

(41) Zhao, Y.; Schultz, N. E.; Truhlar, D. G., Design of Density Functionals by Combining the Method of Constraint Satisfaction with Parametrization for Thermochemistry, Thermochemical Kinetics, and Noncovalent Interactions. J. *Chem. Theory Comput.* **2006**, *2*, *3*64-382.

(42) Goerigk, L.; Hansen, A.; Bauer, C.; Ehrlich, S.; Najibi, A.; Grimme, S., A look at the density functional theory zoo with the advanced GMTKN55 database for general main group thermochemistry, kinetics and noncovalent interactions. PCC*P* **2017**, *19*, *3*2184-32215.

(43) Tomasi, J.; Mennucci, B.; Cammi, R., Quantum Mechanical Continuum Solvation Models. Che*m. Rev.* **2005**, *105, 2*999-3094.

(44) Grimme, S.; Antony, J.; Ehrlich, S.; Krieg, H., A consistent and accurate ab initio parametrization of density functional dispersion correction (DFT-D) for the 94 elements H-Pu. *J. Chem. Phys.* **2010**, *132, 1*54104.
